# Anode-Free Batteries: Pioneering Energy Storage Revolution

**DOI:** 10.1021/acs.chemrev.5c00533

**Published:** 2026-02-05

**Authors:** Yosef Nikodimos, Kassie Nigus Shitaw, Teklay Mezgebe Hagos, Tsung-I Yeh, Yu-Chun Huang, Chia-Lung Hsieh, Hsuan-Hsuan Su, Wei-Nien Su, Bing Joe Hwang

**Affiliations:** † Nano-electrochemistry Laboratory, Department of Chemical Engineering, 34878 National Taiwan University of Science and Technology, Taipei City 106, Taiwan; ‡ Sustainable Electrochemical Energy Development (SEED) Center, 34878 National Taiwan University of Science and Technology, Taipei City 106, Taiwan; § Nano-electrochemistry Laboratory, Graduate Institute of Applied Science and Technology, National Taiwan University of Science and Technology, Taipei City 106, Taiwan; ∥ National Synchrotron Radiation Research Center (NSRRC), Hsinchu 30076, Taiwan

## Abstract

Anode-free batteries (AFBs) represent a transformative approach in energy storage technologies, eliminating the anode to achieve remarkable improvements in energy density, safety, and manufacturing simplicity. This review provides a comprehensive analysis of the fundamental phenomena governing the operation of AFBs during metal plating and stripping, including nucleation and growth, dendrite formation, dead lithium formation and regeneration mechanisms, morphological evolution, and electrolyte decomposition. The challenges associated with AFBs are critically examined, alongside various designed innovative strategies. Expanding the discussion beyond lithium, this review explores advancements in anode-free sulfur, sodium, potassium, zinc, magnesium, aluminum, and all-solid-state batteries, highlighting the unique challenges and solutions tailored to each system. Critical parameters influencing performance, including Coulombic efficiency, electrolyte-to-cathode ratio, and cycling protocols, are analyzed to provide insights into optimizing these systems. Furthermore, the potential of AFBs as a platform for probing interfacial phenomena is emphasized, with applications in visualizing metal deposition, quantifying dead metal formation, and rapid screening of electrolytes. The review concludes with a perspective on future research directions aimed at addressing current limitations and accelerating the practical deployment of AFBs. By bridging fundamental understanding and innovative design, this work positions AFBs as a pivotal technology for the next generation of high-performance energy storage systems.

## Graphical abstract

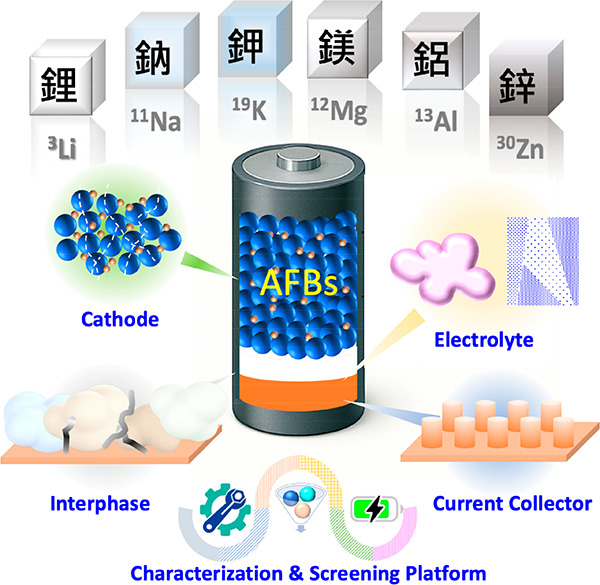

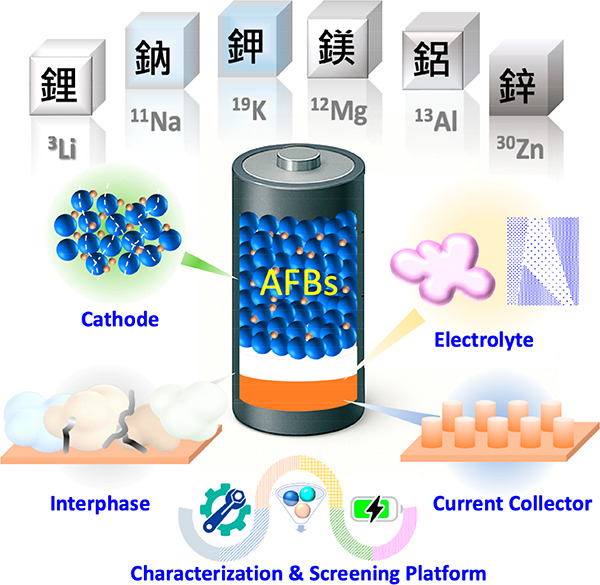

## 1 Introduction

The landscape of energy storage is undergoing a remarkable transformation, driven by the ever-increasing demand for efficient, reliable, and sustainable power sources. Rechargeable batteries, often referred to as the linchpin of this transformation, have evolved significantly over the years. The emergence of commercial lithium-ion batteries (LIBs) has had a profound impact on society since their introduction in the 1990s.^1,2^ These batteries have found application in diverse fields, ranging from small-scale electronics to powering electric vehicles. The fusion of solid-state physics and electrochemistry has propelled innovations in LIB configuration, facilitating their integration into unmanned aerial vehicles and grid-scale energy storage systems.^3^ Despite their increasing energy density, the journey toward achieving these milestones has been marked by numerous challenges. Conventional LIBs primarily utilize graphite as the anode, boasting a theoretical capacity of 372 mAh g^–1^. Paired with compatible cathodes, these batteries typically exhibit cell-level energy densities of approximately 250 Wh kg^–1^ (approximately 700 Wh L^–1^),^4^ and pouch cell models less than 300 Wh kg^–1^.^5,6^ While the integration of silicon into graphite has shown promise in boosting energy density,^7,8^ current limitations cap it at around 300 Wh kg^–1^.

As conventional LIB technology nears its limits, particularly with mature production techniques for graphitic anodes and lithium-containing oxide cathodes, exploring alternative electrode formats becomes imperative for enhancing overall performance metrics. The adoption of lithium-metal anodes holds promise for significant enhancements in energy density. Lithium metal possesses a low redox potential (−3.04 V versus standard hydrogen electrolyte [SHE]) and boasts a high theoretical capacity of 3860 mAh g^–1^ (2061 mAh cm^–3^),^9–12^ surpassing other feasible anode materials. Lithium transition metal oxide (Li-LMO) batteries, for instance, offer a specific energy of approximately 440 Wh kg^–1^. Although lithium-metal anodes offer numerous benefits, their practical application has been impeded by the inadequate reversibility of lithium plating and stripping.^13,14^ The development of extensive, high surface area lithium plates, known as “mossy” plating, contributes to lithium depletion through unwanted side reactions.^15^ While excess lithium can counteract this issue, it compromises the energy density benefit inherent in lithium foil anode. In recent years, researchers have dedicated significant efforts to stabilize lithium plating and enhance the cycling performance of lithium-metal anodes.^16–18^ Moreover, the conventional lithium-metal battery (LMB) requires excess lithium as anodes, impeding additional energy density improvements. The use of lithium foil as an anode in LMBs faces significant challenges, including poor Coulombic efficiency (CE) and the uncontrolled growth of dendrites. These issues not only hinder performance but also pose serious safety risks.^19^

Recent advancements in alkali metal-based batteries, including (Na, K, and even multivalent systems like Mg, Zn, and Al have emerged as promising candidates for next-generation energy storage.^20–22^ These elements, particularly Na and K, offer improved earth abundance and reduced material costs compared to lithium, aligning well with long-term sustainability goals.^23,24^ Sodium metal, for instance, shares similar electrochemical properties with lithium but is more plentiful and cheaper to extract. Despite its slightly higher redox potential (−2.71 V vs SHE) and lower theoretical capacity (1166 mAh g^–1^), its adoption is gaining traction, especially for large-scale stationary storage. All solid-state batteries, which replace flammable organic electrolytes with solid electrolytes, are another frontier garnering intense research focus. Solid electrolytes promise enhanced safety, wider electrochemical windows, and compatibility with high-capacity metal anodes.^25,26^ However, challenges such as poor interfacial contact, limited room-temperature ionic conductivity, and dendrite formation through grain boundaries continue to hinder commercialization. As the field evolves, a holistic approach that encompasses performance, safety, sustainability, and scalability will be essential in guiding the development of future rechargeable battery technologies.

In this context, AFBs emerge as a groundbreaking innovation in the world of energy storage aiming to enable lithiated cathode systems to achieve maximum energy density without the hindrance of excess metal.^27–29^ Although “anode-free” indicates that no active anode metal is present at assembly, the anode forms in situ during the first charge as the active metal electrochemically plates onto the current collector (CC).^30,31^ Therefore, the term “anode-free” refers more accurately to the absence of active anode material in the as-fabricated cell, rather than to the absence of anode during operation. This design stands in contrast to traditional metal-ion and metal batteries, which utilize preinstalled anode materials such as graphite, silicon, or the metal itself in foil form. Figure 1 schematically compares traditional metal-ion/metal batteries and anode-free configurations, highlighting key structural differences. In conventional configurations, the anode participates in both initial and subsequent cycles. Anode-free architectures, however, rely entirely on metal sourced from the cathode.

**Figure 1:**
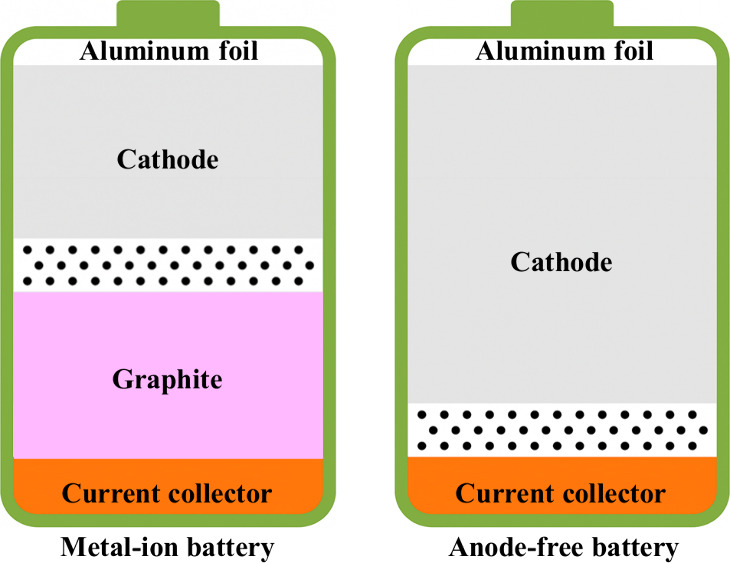
Schematic comparison between traditional metal-ion and AFBs. The traditional design includes an anode, while the anode-free design uses a bare CC on which the metal plates during the first charge.

AFBs hold great significance for several reasons.^27,32–34^ First, they hold the potential for dramatically increased energy density, making them an attractive option for applications requiring high-capacity energy storage in a compact footprint. Second, the elimination of the anode at the initial fabrication simplifies the battery architecture, potentially reducing weight and cost, and enhancing overall energy density. Furthermore, AFBs present a compelling path toward enhanced cell safety by eliminating the need for ultrathin alkali metal, better safety and shelf life due to the omission of alkali metal during its fabrication, and a more accurate evaluation of battery performance without excess alkali metal to compensate for irreversible losses.

The motivation for this review is rooted in the need to comprehensively understand AFBs, their underlying principles, and their potential to transform the future of energy storage. As presented in Figure 2, the aim is to explore the fundamental electrochemical mechanisms governing AFBs, the materials and electrolytes used, innovations, challenges and strategies. Additionally, critical parameters in AFBs and the applicability of AFB systems as powerful tools to study interfacial phenomena are extensively discussed. The review concludes by offering insights into future research directions and areas of exploration in the realm of AFBs. It identifies the main challenges, open research questions, and potential future developments that will shape the trajectory of energy storage technology. As this journey through the intricate world of AFBs unfolds, the goal is to provide a comprehensive overview of this technology, enabling readers to grasp its potential for revolutionizing energy storage and addressing the pressing challenges of our energy-dependent society.

**Figure 2:**
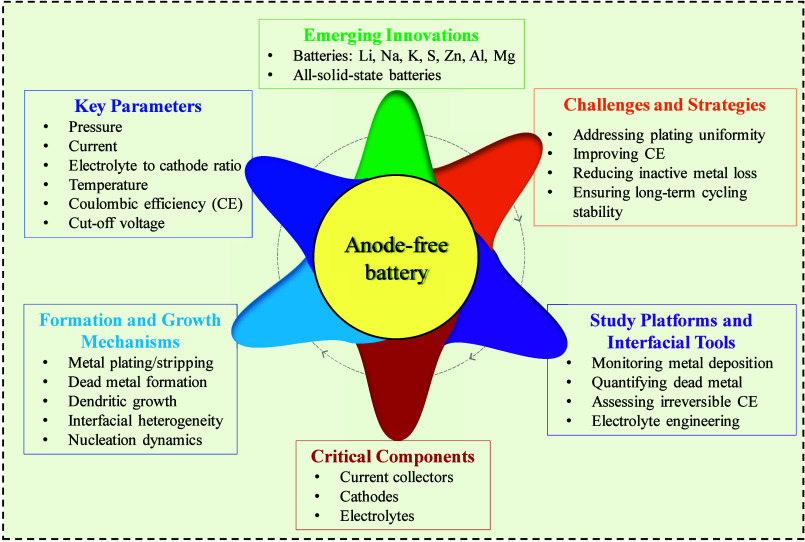
Schematic illustration providing a comprehensive overview of the key aspects, challenges, and opportunities in the development of AFBs covered in this review.

## 2 Fundamentals of Anode-Free Batteries

Unlike conventional metal-anode systems, which utilize a preplated metal layer, the anode-free configuration relies entirely on reversible in situ metal plating onto a bare CC, such as Cu, Ni, or stainless steel (SS) foil. As such, the entire metal inventory originates from the cathode, where metal-ions are hosted in the crystal structure of an intercalation or conversion-type material.^35,36^ During the initial charging cycle, metal-ions are extracted from the cathode and migrate through the electrolyte to the anode side as shown in Figure 3(a). Upon reaching the CC, these ions are electrochemically reduced, forming a nascent metal film directly on the substrate, based on the reaction:

$$M^{n+}+{\mathrm{ne}}^{-}\to M$$

**Figure 3:**
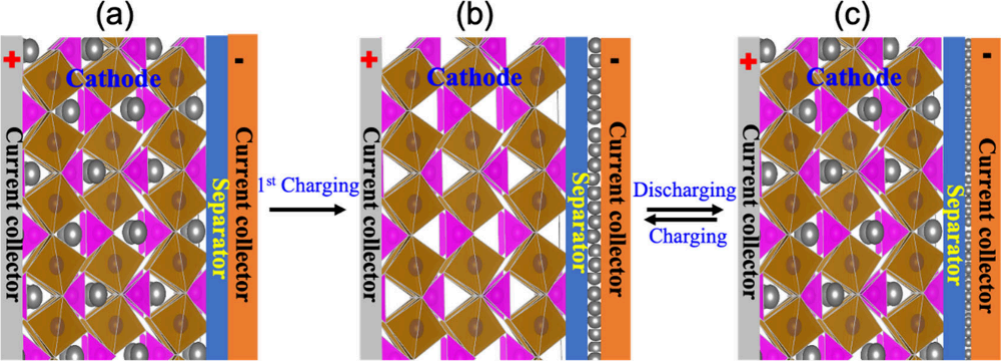
Schematic representation illustrating metal plating/stripping processes. (a) As-assembled AF cell: cathode||CC (no active metal on the negative side). (b) First charge: ions are deintercalated from the cathode and plate as a conformal thin M° film on the negative CC, establishing the working anode. (c) Subsequent cycling: the preformed thin metal layer reversibly strips/plates. The attainable thickness is limited by the cathode inventory and first-cycle irreversibility (SEI/CEI formation), i.e., the cell is no longer strictly “anode-free” after formation.

This reaction results in the formation of a thin metal layer on the CC, effectively generating the anode in situ. However, the newly plated metal is highly reactive and spontaneously interacts with the surrounding electrolyte, leading to the SEI formation.^37,38^ During discharge, the plated metal is stripped back into the electrolyte as ions and returns to the cathode.

During the discharge cycle, the previously plated metal is oxidized, returning to the cathode material as metal-ions (Figure 3(b)). This electrochemical stripping reaction can be represented as

$$M\to M^{n+}+{\mathrm{ne}}^{-}$$

Ideally, the entire quantity of plated metal is reversibly oxidized and reintegrated into the cathode host. However, due to morphological irregularities, SEI fracturing, and interfacial loss of contact, this process is often incomplete (Figure 3(c)). Therefore, although “anode-free” indicates that no active anode metal is present at assembly, the anode forms in situ during the first charge as the active metal electrochemically plates onto the current collector (CC).^30,31^ Therefore, the term “anode-free” refers more accurately to the absence of active anode material in the as-fabricated cell, rather than to the absence of anode during operation. The interplay between electrochemical kinetics, interfacial stability, and morphology evolution governs the reversibility and efficiency of metal plating and stripping in AFBs.^39–41^ A detailed understanding of these fundamental processes is essential for diagnosing performance degradation and enabling practical implementation. In the following sections, the key phenomena governing metal plating and stripping are discussed in detail, with emphasis on the dynamic interfacial evolution and its implications for cell reversibility and safety.

### 2.1 Plating Phenomena

The initial metal plating process in AFBs is critical in establishing a stable and reversible interface. If plating in AFB systems occurs on a metallophobic CC that lacks uniform nucleation sites, it results in the formation of a new, highly reactive metal surface. This leads to spatially nonuniform plating and complex interfacial evolution.^42,43^ Furthermore, the high reactivity of the freshly plated metal with the electrolyte triggers continuous SEI formation, which can fracture, reform, and evolve throughout cycling.^44,45^ These events collectively govern the morphology, interfacial stability, and electrochemical reversibility of the plated metal layer. Understanding the fundamental mechanisms underlying nucleation, growth, SEI evolution, and associated degradation processes is essential for improving the long-term performance and safety of anode-free cells. The following subsections explore these interconnected phenomena in detail.

#### 2.1.1 Nucleation and Growth Dynamics

Nucleation is the initial formation of a new phase, in this case, the formation of metallic Li, Na, or other metal deposits, on the CC surface during the first charging cycle of AFBs. Since there is no pre-existing metal anode, metal atoms must nucleate directly onto a bare CC (usually Cu or other conductive substrate). The characteristics of this initial metal nucleation are crucial as they determine the resulted plated metal morphology, which in turn affects the cycling stability of the entire battery. This nucleation process is governed by the thermodynamics of Gibbs free energy changes, as metal-ions transition to a saturated state from a supersaturated solution at the interface between the CC and the electrolyte.^46,47^ This process is crucial for the reversible operation of the battery and determines its cycling stability and safety.^48^

Briefly, the nucleation and growth dynamics of metal plating in AFBs typically involves two fundamental steps. The first is the nucleation stage, which occurs during the initial charging cycle. In this phase, M^n+^ ions originating from the cathode migrate through the electrolyte and are reduced at the surface of the CC. This reduction leads to the formation of initial metal nuclei. The uniformity and distribution of these nuclei are critical, as they dictate the morphology and stability of the subsequently plated metal layer.^49^

After the initial nucleation phase, the next step is the growth stage wherein the initial nuclei evolve into larger plated metal through continued ion reduction and metal accumulation. The growth process occurs, following nucleation, at the surface of these nuclei, with additional metal-ion preferentially plated on the original nucleus, causing it to grow into larger metal structures.^50^ This growth process is influenced by various factors, including local charge transfer, metal-ion diffusion properties, and surface energy, which will determine the local current density and morphology of metal growth. The morphology of the initial nuclei evolves into different forms, such as spheres, needles, columns, or mixtures of these, depending on factors like the electrolyte type, surface energy and plating conditions.^15,51^

To create nuclei of a specific size on a substrate, the reduced metal-ions must overcome a nucleation barrier. This barrier can be controlled by adjusting the electrochemical supersaturation at the electrified interface. A key physical parameter representing this supersaturation is the overpotential.^52^ For reactive metals like, an extra challenge arises from the spontaneous formation of the SEI or passivation film at the metal–electrolyte interface, creating a barrier to ionic transport. In this context, overpotential can be determined as follows:^53,54^

$$\eta =\frac{RT}{\alpha F}\ln\frac{i}{i_{0}}+\frac{2\gamma V}{Fr}+\frac{irRT}{D_{B}C_{B}F^{2}}+\frac{ir^{2}RT}{D_{S}C_{S}F^{2}}$$

where, η represents the total overpotential including the charge transfer overpotential (η_ct_), interfacial (surface) energy overpotential (η_s_), bulk diffusion overpotential, and ionic transport overpotential through the SEI. The last two terms in total represent mass transport overpotential (η_D_) including bulk diffusion and SEI transport of metal-ions. The terms *i* and *i*_0_ denote the current density and the exchange current density, respectively, while *r* is the radius of the nuclei. The parameters *D*_B_ and *C*_B_ refer to the diffusivity and concentration of metal-ions in the bulk electrolyte, whereas *D*_S_ and *C*_S_ represent the diffusivity and concentration of metal-ions within the SEI. Additionally, γ signifies the interfacial energy, and *F*, *T*, *R*, *V*, and α correspond to the Faraday constant, the temperature, the universal gas constant, the molar volume of the metal, and the charge transfer coefficient, respectively. η_ct_ arises from electron transfer resistance at the interface (Ω_ct_), while η_D_ reflects ionic transport limitations through the bulk electrolyte and SEI (Ω_D_). The relative magnitudes of these impedance components determine the dominant limiting process during metal plating and stripping, thereby influencing the local ion concentration, nucleation behavior, and resulting metal morphology as discussed in Sections 2.1.2 and 2.2.

Based on these concepts, Pei et al. studied the processes of lithium nucleation and growth on copper CCs using a galvanostatic technique combined with ex situ scanning electron microscopy (SEM) analysis in a Li||Cu cell system.^52^ As illustrated in Figure 4(a, b), their findings revealed that the size of lithium nuclei decreases as the overpotential increases. Smaller nuclei and uneven lithium morphologies were observed to significantly contribute to faster electrolyte depletion and SEI formation, driven by their higher specific surface area. However, galvanostatic methods have limitations. Our research group proposed a potentiostatic approach and quantified nucleation kinetics considering Li nucleation and growth associated with SEI fracture.^55^ Notably, SEI fracture intensifies over time, highlighting a progressive increase in SEI fracture during plating Figure 4(c, d). Moreover, rate constant for the decomposition of electrolyte rises with overpotential but diminishes with Fluoroethylene Carbonate (FEC) additive, suggesting its role in mitigating SEI fracture. Yan et al. also investigated the Li-nucleation overpotential across various CCs, including Ni, Cu, Sn, Si, Pt, Al, Mg, Zn, Ag, and Au.^56^ Their findings revealed that Au, Ag, Zn, and Mg exhibited lower nucleation overpotential values (Figure 4(e–g)). This behavior can be attributed to the solubility of these metals in Li. When Li interacts with these metal substrates, it forms a solid solution through alloying before the metal-Li phase fully develops. This solid solution serves as a buffer layer, effectively lowering the barriers for Li nucleation and enhancing overall interfacial stability.

**Figure 4:**
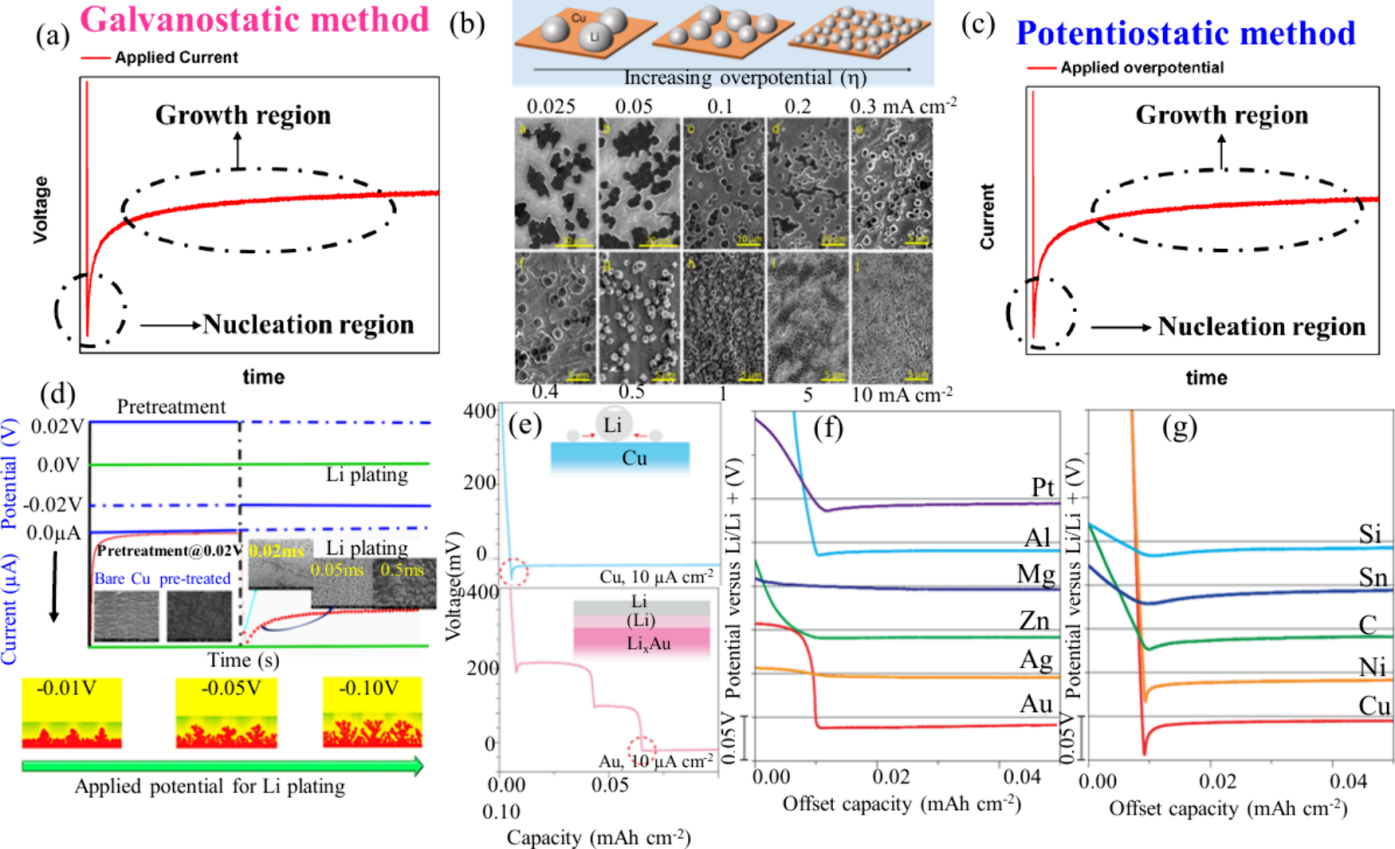
Li nucleation and growth, focusing on the impact of current density and electrolyte properties on plated lithium morphology. (a) Voltage trends during lithium deposition at a constant current. (b) Illustration of lithium nuclei size and density on copper at different overpotentials, accompanied by SEM images of nuclei formed at current densities ranging from 0.025 to 10 mA cm^–2^. (a, b) Reproduced with permission,^52^ Copyright 2017 American Chemical Society. (c) Current response during lithium plating under constant voltage. (d) Experimental setup for analyzing lithium nucleation and growth, including SEM images showing progression over time and a schematic depicting lithium growth and SEI layer fracturing at various voltages on copper foil. (c, d) Reproduced with permission,^55^ Copyright 2019 American Chemical Society. (e) Voltage profiles of Li plating at 10 μA cm^–2^ on copper (top) and gold (bottom) substrates. (f) Voltage profiles for materials partially soluble in Li during plating at 10 μA cm^–2^. (g) Modified voltage profiles for materials with minimal lithium solubility under similar conditions. (e–g) Reproduced with permission,^56^ Copyright 2016 Springer Nature.

#### 2.1.2 Morphology of Plated Metal

The morphology of plated alkali metal (e.g., Li, Na) in AFBs plays a pivotal role in determining the overall electrochemical performance, cycling stability, and safety of the system. Because anode-free configurations lack an excess metal reservoir and rely entirely on the reversible plating and stripping of metal onto a CC (typically Cu), the quality and uniformity of the plated metal directly impact CE, interfacial stability, and the propensity for short-circuiting. The morphology that emerges during metal plating is governed by a complex interplay of interfacial kinetics, substrate characteristics, electrolyte composition, current density, temperature, and pressure. Common morphological classes include uniform (granular), dendritic, and columnar structures, each associated with distinct implications for battery operation. High current density leads to dendrites, while lower current density favors uniform growth. Metallophilic surfaces and coatings promote controlled plating. Electrolyte additives stabilize the SEI and prevent dendrite formation. Elevated temperatures and applied pressure can enhance uniformity but may also trigger side reactions or mechanical issues.

The morphology of metal plating and stripping is critically influenced by the interplay between charge transfer impedance (Ω_ct_) at the electrode–electrolyte interface and the diffusional impedance (Ω_D_) of metal-ions through the electrolyte and SEI as demonstrated in (Figure 5(a, b)). The relative magnitudes of these impedances determine the dominant limiting mechanism during plating and subsequently dictate the uniformity and stability of the plated metal. Two limiting cases, diffusion-limited (Ω_ct_ ≪ Ω_D_) and charge-transfer-limited (Ω_ct_ ≫ Ω_D_) regimes, result in distinct plating behaviors.

**Figure 5:**
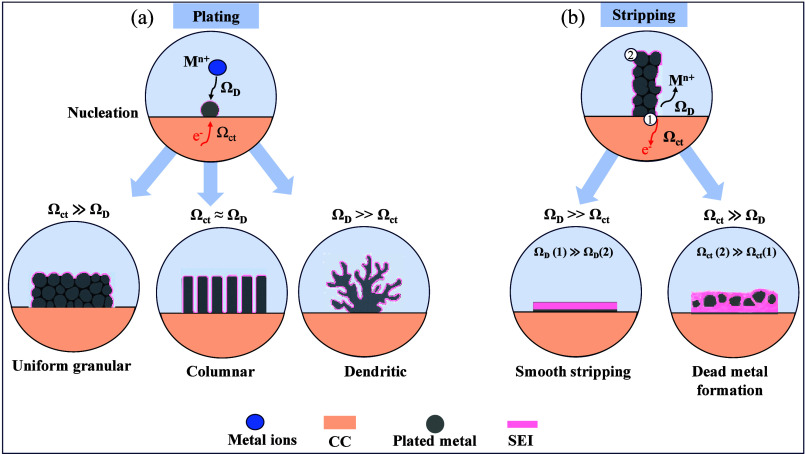
Schematic illustration of typical metal plating and stripping morphologies in AFBs. (a) plating phenomena (b) stripping phenomena.

When the charge transfer resistance is much lower than ionic diffusion resistance (i.e., Ω_ct_ ≪ Ω_D_), the plating process becomes diffusion-limited (Figure 5(a)). Under such conditions, metal-ions are rapidly reduced at the electrode surface, while their replenishment from the bulk electrolyte is slow. This leads to the formation of concentration gradients near the electrode interface and the development of M^n+^ depletion zones, particularly during extended or high-rate cycling.^52,57^ These nonuniform ion distributions give rise to preferential metal growth at localized, M^n+^-rich regions. The electric field becomes concentrated at protrusions, accelerating further plating in these regions.^58,59^ As a result, metal plating tends to proceed in a nonuniform, filamentary, or dendritic manner.^60,61^ This morphology not only deteriorates CE due to increased SEI formation and “dead metal”, but also poses serious safety risks through potential short-circuiting in liquid or solid electrolytes.

Conversely, when charge transfer is the rate-limiting step (i.e., Ω_ct_ ≫ Ω_D_), the process is said to be charge-transfer-limited (Figure 5(a)). In this regime, ionic transport through the electrolyte and SEI is relatively efficient, allowing metal-ions to arrive uniformly across the electrode surface. However, the slow kinetics of electron transfer at the interface leads to a relatively uniform distribution of electrochemical potential and reaction rates.^62^ This uniform electrochemical environment promotes uniform nucleation and uniform growth of metal across the CC. The metal is plated in a dense, compact morphology, minimizing the surface area exposed to the electrolyte and thus suppressing excessive SEI formation. This morphology is highly desirable, as it improves cycling reversibility, reduces dead metal formation, and enhances long-term cell stability.^15,63^

#### 2.1.3 Electrolyte Decomposition Associated with SEI Fracture

The mechanical integrity of the SEI is continuously challenged during metal plating in AFBs. The volumetric expansion of the metal layer during plating, coupled with the inherently uneven growth of morphologies such as dendrites, filaments, or mossy structures, generates significant local mechanical stress.^64–66^ These stresses frequently exceed the brittle SEI’s strain tolerance, leading to fracture and rupture. Once fractured, fresh reactive metal surfaces become exposed to the electrolyte, initiating renewed parasitic reactions and promoting the formation of a secondary SEI. This repeated cycle of breakdown and repair, commonly termed “SEI breathing,” results in continuous electrolyte consumption, progressive metal loss, and the evolution of a thick, resistive, and heterogeneous interphase.^67,68^

Crucially, this process has been quantitatively described using a side-reaction, *J*_SEI_, which captures the electrolyte decomposition arising from SEI fracture. In the metal-SEI model, proposed by our research group, the total current observed during metal plating is expressed as a combination of two processes: the diffusion-controlled nucleation and growth metal (*J*_3D‑DC_), and the electrolyte decomposition induced by SEI fracture (*J*_SEI_):^55^

$$\begin{array}{ll} J_{\mathrm{total}}\left(t\right) & =J_{3\mathrm{D‐DC}}\left(t\right)+J_{\mathrm{SEI}}\left(t\right)\\ & =\frac{a}{\sqrt{t}}\left[1-\exp^{-ct}\right]+d\left(1-\exp^{-c\left(t-\frac{1-\exp^{-Nt}}{N}\right)}\right) \end{array}$$

where,

$$a=\frac{nFD^{1/2}C^{\infty }}{\sqrt{\pi }}\text{,}\;c=N_{0}\pi D\sqrt{\frac{8\pi MC^{\infty }}{\rho }}\text{,}\;d=\left(n_{\mathrm{SEI}}Fk_{\mathrm{SEI}}\right)\left(\sqrt{\frac{2C^{\infty }M}{\pi \rho }}\right),$$

g=N where *nF* (C mol^–1^) is the molar charge transferred during the Li metal deposition; *D* (cm^2^ s^–1^) is the diffusion coefficient of metal-ion through SEI layer; *C* ^∞^ (mol cm^–3^) is the bulk concentration of metal-ions in the solution; *M* (g mol^–1^) is the molar mass; ρ (g cm^–3^) is the density of the metal; *N*_0_ (cm^–2^) is the number of nucleation sites toward the nucleation on the electrode surface; *N* (s^–1^-) is the nucleation rate-; *n*_SEI_ *F* (C mol^–1^) is the molar charge transferred during the electrolyte decomposition; and *k*_SEI_ (mol cm^2^ s^–1^) is the rate constant of the electrolyte decomposition due to SEI fracture. The model provides a powerful framework for quantitatively assessing SEI degradation, enabling the extraction of key kinetic parameters, such as *D*, *N*_0_, and *k*_SEI_, from chronoamperometry measurements. Importantly, experimental results indicate that *J*_SEI_ increases with time, suggesting an autocatalytic loop in which freshly exposed metal sites accelerate continuous SEI fracture and reformation.

From a mechanistic standpoint, the origin of SEI fracture lies in the morphological evolution of the depositing metal. Irregular features introduce sharp curvatures and localized strain fields that the SEI, composed of brittle inorganic compounds such as Li_2_CO_3_, LiF, and Li_2_O (for instance, in the cases of lithium-based AFB) cannot accommodate.^69,70^ Additionally, repeated metal plating/stripping cycles lead to dynamic volumetric expansion and contraction at the interface, exacerbating stress concentrations and promoting interfacial rupture. Following fracture, electrolyte solvents (e.g., ethylene carbonate (EC), dimethyl carbonate (DMC)), diethyl carbonate (DEC), ethyl methyl carbonate (EMC), undergo reductive decomposition to form inorganic carbonates and gaseous byproducts like ethylene. At the same time, LiPF_6_ salt degradation produces PF_5_ and HF. These byproducts are chemically aggressive and further attack the interphase. Additive molecules, such as fluoroethylene carbonate (FEC), vinylene carbonate (VC), and propylene carbonate (PC), initially contribute to SEI stabilization. However, they are also subject to reduction at newly exposed metal surfaces, leading to SEI thickening and compositional heterogeneity. This degradation manifests as a larger *k*_SEI_ at higher overpotentials, indicating accelerated electrolyte decomposition and interphase failure.^71–73^

The progressive accumulation of decomposition products results in increased interfacial resistance, loss of cyclable metal and electrolyte, greater localized current densities, more severe dendritic growth, potential gas evolution and pressure buildup in sealed cells. Suppressing *J*_SEI_, through interface engineering, electrolyte additive optimization, or surface modification, is therefore critical to enhancing AFB longevity and safety. The metal-SEI model offers a foundational tool to evaluate and compare interfacial stability across different substrates and electrolytes under various operating conditions.

#### 2.1.4 Metal Inventory Formation Mechanism

In the context of AFBs, “metal inventory formation” refers to the process by which the metal (such as Li) forms and accumulates on the CC during the initial and subsequent charge/discharge cycles. In AFBs, the anode is not prelithiated during the battery assembly. Instead, the anode is created in situ during the first charge cycle by plating Li metal onto the CC during the initial charge cycle. The mechanism of metal inventory formation in AFBs primarily involves metal plating on a CC during the charge cycles.^74^ At the first cycle of charging, metal-ions from the positive electrode move through the electrolyte and are reduced at the CC (typically copper) on the anode side, forming a plated metal layer. The process begins with the nucleation of the metal atoms on the CC. The quality and uniformity of this nucleation process are crucial for forming a stable and dense plated metal layer.^27,75,76^ After nucleation, the plated metal layer grows as more metal-ions are reduced and plated. However, the irreversibility of the first cycle leads to the formation of an active metal reservoir at the anode.^74^ Furthermore, it was reported that the first-cycle irreversibility could be recovered by discharging at a low potential, confirming the presence of residual active lithium at the anode.^74,77^ Overall, the metal inventory is established during the initial plating process upon the first charge, as metal-ions are deposited directly onto the bare CC. This inventory is typically evaluated during the subsequent stripping process, where the reversible capacity provides insight into the amount of active metal available and the extent of irreversible losses.

Several factors can influence the formation of metal inventory. The surface properties of the CC, such as roughness and chemical composition, influence the nucleation and growth of lithium metal.^78–80^ A smooth and chemically compatible surface can promote uniform lithium plating and excellent reversibility. The choice of electrolyte can also impact the lithium plating and stripping process.^81^ Some electrolytes and additives can form a stable SEI layer, which can enhance the uniformity of lithium plating and protect against dendrite formation. Gervillié-Mouravieff et al. investigated the lithium inventory in pouch-based AFBs configured as NMC622||Cu.^77^ The cells were charged to 4.3 V and fully discharged to 1 V, while testing several electrolyte formulations. These included high-concentration electrolytes (HCE; 4M-HCE with LiFSI-2.4DME and 8M-HCE with LiFSI-1.2DME in molar ratios), a commercial carbonate-based electrolyte (CCE; 1.2 M LiPF_6_ in EC/DEC with 10 wt % FEC), and a localized highly concentrated electrolyte (LHCE; LiFSI-1.2DME-3TTE in molar ratios). The voltage profiles, as shown in Figure 6(a), displayed similar patterns across all formulations, featuring a reversible charge and discharge slope between 4.3 and 3 V, along with a brief voltage plateau at 1.6 V during discharge. This plateau indicates that the lithium reservoir stored at the anode can be retrieved at low voltages through cathode overlithiation. Although the first-cycle performance of cells with LHCE and CCE electrolytes was comparable, LHCE-based cells demonstrated significantly improved cycle life Figure 6(b)). The lithium reservoir was completely depleted after 12 cycles for CCE and 30 cycles for LHCE.

**Figure 6:**
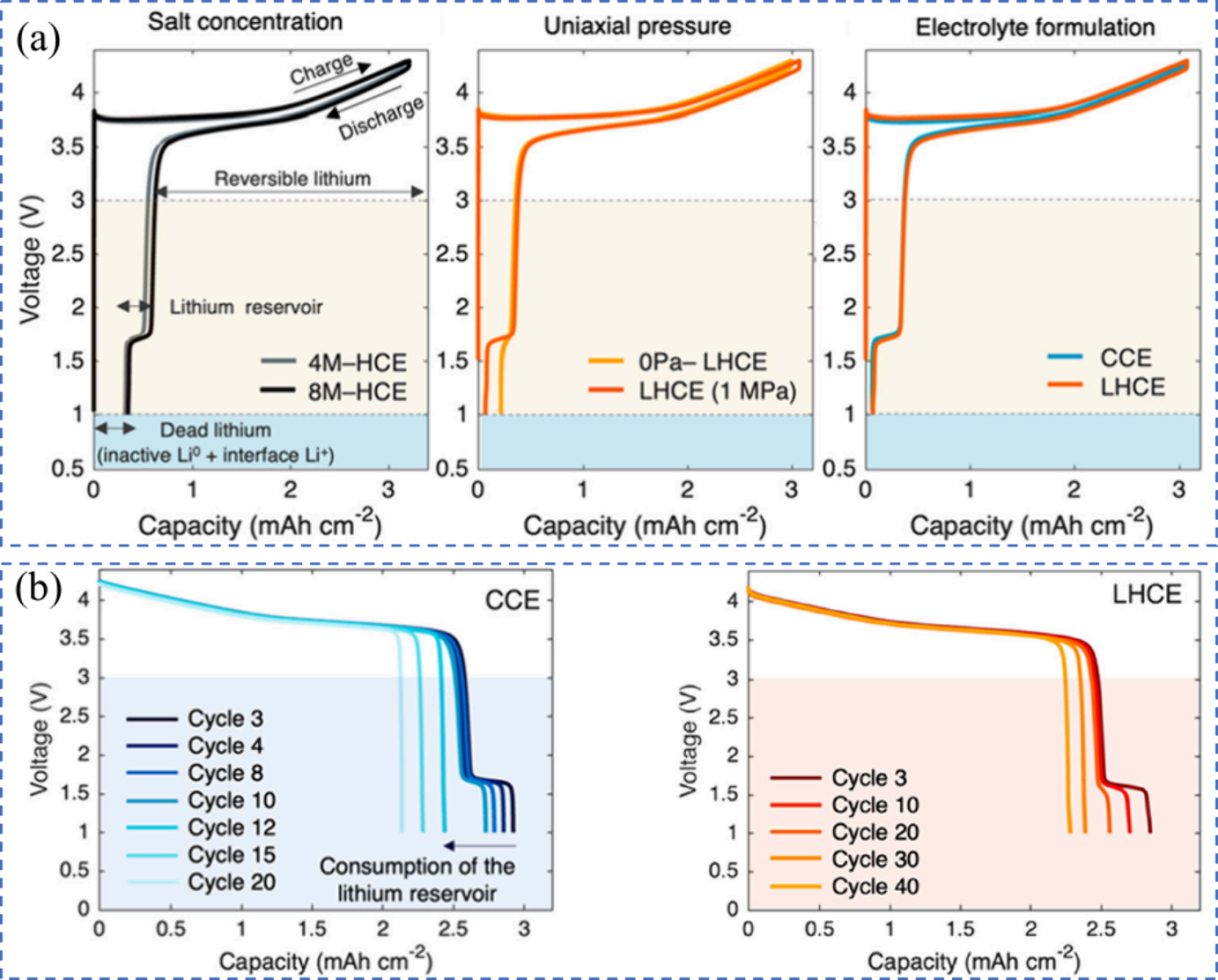
Lithium inventory in AFBs. (a) First cycle lithium inventory in NMC622||Cu pouch-based AFB. (b) Lithium reservoir consumption. (a, b) Reproduced with permission,^77^ Copyright 2024 American Chemical Society.

The formation mechanism of the metal inventory in AFBs are critical to their performance and longevity. Understanding the mechanisms of lithium plating, stripping, and replating, as well as the factors that influence these processes, is essential for advancing AFB technology. Recent advancements in surface engineering,^82–86^ electrolyte optimization,^87,88^ and innovative materials,^89–92^ offer promising pathways to overcome the challenges associated with metal inventory formation. Continued research and development in these areas are crucial for realizing of high-performance AFBs.

#### 2.1.5 Heterogeneity Formation Mechanism

In the early phase of metal plating, the interaction between the CC and the liquid electrolyte is key in shaping the alkali nucleation morphology. CCs like Cu exhibit low wettability with the electrolyte due to their hydrophobic nature. This poor wettability results in heterogeneous alkali metal nucleation on the Cu CC surface, ultimately contributing to the uncontrolled formation of sharp metal dendrites.^93,94^ Figure 7(a, b) illustrates how the Cu CC surface influence the metal nucleation and growth as well as the formation of metal dendrites in AFBs. In an AFB configuration, the relationship between the metallophilicity of the CC and its electrochemical stability is crucial.^93^ The metallophobic nature of the Cu CC leads to a lattice mismatch with the platting metal during the initial stage of plating, resulting in small metal nuclei with a large contact angle.^75^ In contrast, the CC with a metallophilic matrix lowers the nucleation energy barrier at the surface, enabling uniform metal plating with a lower contact angle.^95^ This uniform plating not only reduces the likelihood of a battery short circuit but also enhances the reversibility of the metal plating and stripping processes^96^

**Figure 7:**
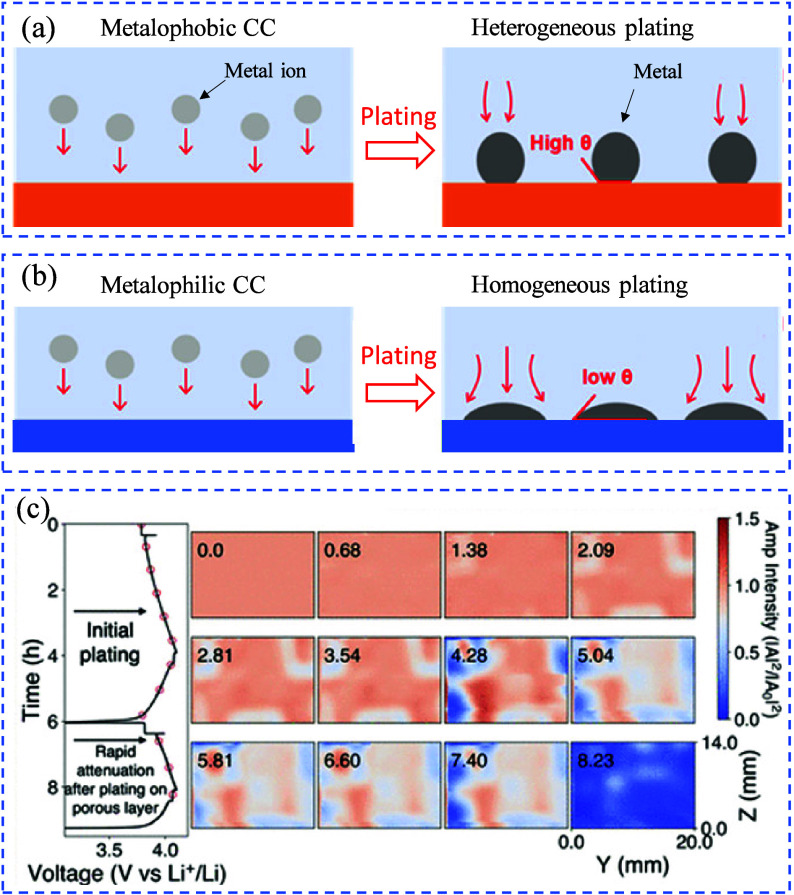
Schematic demonstrating (a) heterogeneous and (b) uniform plating formation mechanisms in anode-free lithium-metal batteries (AF-LMBs), depending on the lithiophilicity behavior of the CC. (c) The time-domain amplitude operando scanning of AF-LMBs reveals complete amplitude attenuation after plating on the initial porous layer. (c) Reproduced with permission,^97^ Copyright 2021 American Chemical Society.

In AFBs, the main phase change occurs due to the plating of a metal on the negative CC (creating a new phase) and the consumption of the electrolyte (transitioning from liquid to gas). Chang and Steingart employed a spatially resolved operando scanning to detect local variations in phase behavior across the entire cell area during charging, irrespective of the applied practical rates.^97^ Operando acoustic scanning provides a map of electrolyte consumption rates during cycling, as well as spatial variations in interfacial roughening, which contributes to acoustic scattering. Using a 1 M LiPF_6_ EC (3:7 w/w%) + 2% VC electrolyte, characterized by poor CE and capacity retention, acoustic amplitude attenuation is observed starting in the second charge cycle Figure 7(c)). This attenuation first occurs at the edges of the electrode stack, showing limited effects in the initial plating step. After the first cycle, the attenuated areas rapidly spread inward during the second plating step.

Overall, the formation of interfacial heterogeneity in AFBs primarily stems from the poor wettability and metallophobic nature of the CCs, which promote nonuniform metal nucleation and dendrite growth. In contrast, metallophilic surfaces facilitate uniform plating and improved reversibility. Operando imaging reveals that electrolyte consumption and interfacial roughening, particularly initiating at electrode edges, exacerbate spatial heterogeneity during cycling. To mitigate these issues, rational design of metallophilic interfaces, advanced electrolyte formulations, and edge-focused structural strategies are essential. Incorporating operando diagnostics can further guide the development of more stable and uniform plated metal interfaces.

### 2.2 Stripping Phenomena

The stripping process in AFBs is the electrochemical oxidation of the previously plated metal back into ionic form during discharge. Unlike conventional metal anodes, which often exhibit some degree of structural integrity and abundance, the freshly plated metal in anode-free configurations is typically thin, morphologically heterogeneous, and mechanically fragile. This makes the stripping process highly sensitive to interfacial instabilities, mechanical detachment, and electrochemical inhomogeneity. As the metal is stripped, any nonuniformity in current density, SEI coverage, or substrate contact can result in partial stripping, leaving behind isolated metallic clusters that become electronically disconnected. This not only causes irreversible capacity loss but also induces void formation, increases interfacial impedance, and compromises subsequent plating cycles. Furthermore, the continuous evolution of the SEI and the intrinsic instability of the stripped interface exacerbate these challenges. Understanding the stripping mechanisms is crucial for improving the reversibility and lifetime of anode-free systems.

Metal stripping is governed by the availability of charge carriers and the ability to transfer electrons at the Metal|electrolyte interface. The uniformity of this process is dictated by the interplay between Ω_ct_ and Ω_D_, with their spatial distribution playing a critical role (Figure 5(b)). In the diffusion-limited regime (Ω_ct_ ≪ Ω_D_), charge transfer is relatively facile, but sluggish ion transport leads to nonuniform Li^+^ concentration gradients near the interface. As the stripping current preferentially follows regions of lower concentration overpotential, plated metal is oxidized from the top layer rather than from the bottom layer, potentially resulting in leveling effect, uniform stripping and less dead metal formation.

However, in the charge-transfer-limited regime (Ω_ct_ ≫ Ω_D_), ionic mobility is sufficient to homogenize the metal-ion concentration, but the interfacial electron transfer becomes rate-limiting. More importantly, Ω_ct_ is spatially nonuniform across the thickness of the plated metal layer. As illustrated in (Figure 5(b)), the plated metal closest to the CC (i.e., the bottom layer (site 1)) exhibits a much lower Ω_ct_ due to direct contact with the electron-conducting CC, enabling preferential oxidation. In contrast, the plated metal located further from the CC (top layer (site 2)) suffers from higher Ω_ct_ and poor electronic connectivity, severely limiting its ability to undergo oxidation. This leads to bottom-up stripping, where the metal near the CC is stripped first, eventually isolating the top plated metal and rendering it electrochemically inactive. The consequence is the formation of dead metal, resulting in capacity loss, lowered CE, and an unstable interface for subsequent plating. Therefore, while moderate charge-transfer impedance may help distribute stripping current more uniformly, excessively high or spatially heterogeneous Ω_ct_ accelerates the formation of dead metal.

#### 2.2.1 Dead Metal Formation Phenomena

As mentioned above, stripping involves the oxidation and removal of plated metal back into the cathode through the electrolyte as ions. However, nonuniform current distribution and weak adhesion of the plated metal to the CC often result in partial detachment of metallic regions.^45,98^ These electrically disconnected regions become “dead metal” as demonstrated in Figure 8(a). This dead metal is electrically inactive and cannot participate in subsequent charge–discharge cycles.^69^ As a result, it exhibits no electrochemical activity in the batteries, leading to a rapid decline in capacity and significantly reducing the cycle life of AFBs. Their formation is strongly tied to initial plating uniformity, SEI integrity, and substrate–metal affinity. The transition from active to dead metal is not only a loss of cyclable ions but also creates a porous, fragile interface that is prone to further degradation. In AFBs, where the reversibility of metal-ions is crucial for practical deployment, dead metal formation remains a dominant factor limiting cycle life.

**Figure 8:**
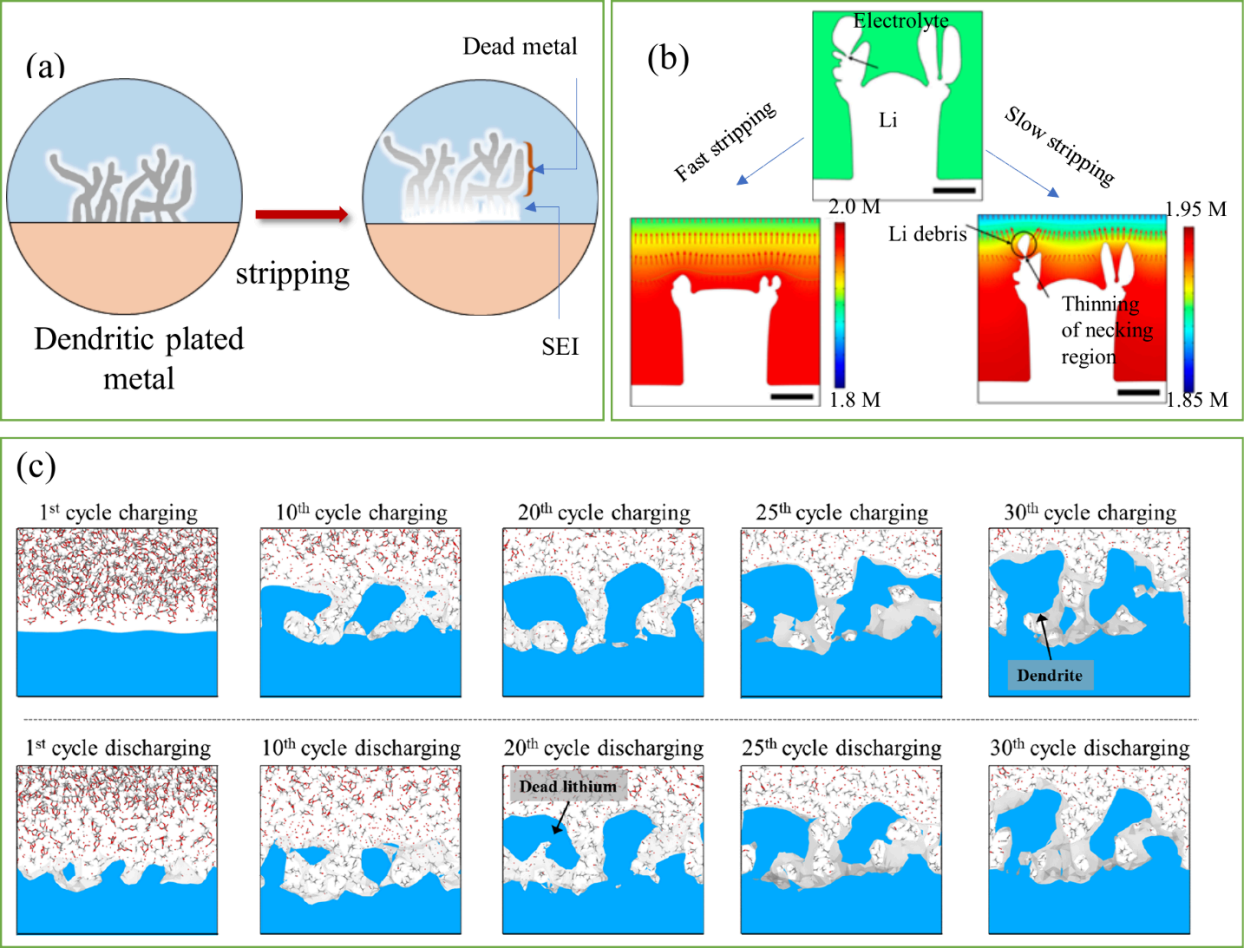
(a) Illustration of the process leading to dead metal formation in AFBs. (b) Accumulation of lithium at a constricted region (indicated by the black arrow) during gradual stripping. (b) Reproduced with permission,^99^ Copyright 2018 American Chemical Society. (c) Development and growth of dead lithium and dendrites in an electrolyte containing only ethylene carbonate. (c) Reproduced with permission,^103^ Copyright 2022 Nature Publishing Group. Stripping morphology phenomena under impedance-limited regimes.

The dead metal formation has been studied using various computational techniques, including models such as the continuum mechanics model,^99^ the mechano-electrochemical phase-field model,^100,101^ and mesoscale computational model.^102^ For example, in lithium-based AFBs, Yoon et al. used a continuum mechanics model to show that dead lithium can form easily when plated lithium is stripped slowly, especially when the plating has irregular shapes, which are commonly seen after rapid plating (Figure 8b)).^99^ This suggests that varying the charge/discharge rates during battery cycles increases the likelihood of dead lithium formation. Additionally, Lee et al. conducted reactive molecular dynamics (MD) simulations to investigate the formation of dead lithium.^103^ Their results, shown in Figure 8(c), reveal that during repeated charge–discharge cycles, dead lithium and dendrite growth develop with a sharp, fiber-like structure between the 20th and 30th cycles. Therefore, to minimize the formation of dead lithium, it is essential to ensure the moderate reactivity of the SEI and maintain a uniform pattern of metal plating.^104,105^ In general, heterogeneous metal plating, caused by nonuniform current distribution, results in uneven dead metal formation. Additionally, side reactions between the metal and the electrolyte can form an SEI layer that traps metal-ions. Mechanical stress, which results from volume changes during cycling, can lead to the formation of cracks and the separation of lithium into isolated particles. These issues collectively contribute to the mechanism of dead lithium formation.

#### 2.2.2 Contact Loss from Current Collector

The contact between the electroplated metal and the CC is essential for efficient charge transport during stripping. However, as cycling proceeds, morphological and interfacial instabilities often lead to localized or widespread loss of electrical contact between the plated metal and the underlying substrate. This phenomenon, referred to as contact loss, significantly compromises the reversibility and utilization efficiency of the plated metal.

During stripping, nonuniform metal removal commonly results in the formation of voids at the interface between the metal layer and the CC. These voids emerge due to preferential stripping from areas of higher local current density or thinner plating, while other regions retain unreacted metal. Once voids form and expand, they disrupt the electronic percolation network, isolating portions of the metallic layer and rendering them electrochemically inactive. This isolated material, although still metallic and chemically active, becomes electrically disconnected and effectively behaves as “dead metal.”

The situation is further exacerbated by the absence of a prehosted or structured anode scaffold, which in conventional systems can buffer against volume changes and maintain structural integrity. In anode-free configurations, the plated metal is typically soft, porous, and loosely adhered, making it highly susceptible to delamination under the influence of interfacial stress, SEI growth, or mechanical deformation during cycling. Moreover, repeated plating and stripping cycles exacerbate the interfacial mismatch due to continuous expansion and contraction, which promotes mechanical fatigue and further compromises adhesion.

Contact loss not only reduces the effective active area for subsequent plating but also increases local current density at remaining contact sites. This can further accelerate nonuniform plating, SEI fracture, and dendritic growth during replating cycles. The resulting feedback loop severely limits CE and cycle life. Engineering strategies aimed at mitigating contact loss include the use of metallophilic coatings, 3D porous CCs, alloy interlayers, and electrolyte formulations that promote uniform stripping. A mechanistic understanding of how stripping dynamics interact with interface morphology is essential for guiding such design principles. Ultimately, improving interfacial contact retention across cycles remains a central challenge to achieving practical and durable AFBs.

#### 2.2.3 Dead Metal Regeneration Mechanism

Reviving dead metal by breaking down the SEI layer and converting it back into active metal is an effective method to extend the lifespan of AFBs. For instance, Tao et al. developed a strategy involving redox reactions that decompose SEI components to restore the electronic pathway of inactive lithium, as shown in Figure 9(a).^106^ Their cryo-TEM analysis revealed that Li_2_O is a significant component of the SEI in both ether and ester-based electrolytes. To tackle this, they introduced an iodine mediator, which facilitates the breakdown of Li_2_O into soluble compounds. This process gradually disintegrates the SEI layer around the inactive lithium, exposing fresh lithium and enabling the formation of a new SEI. Meanwhile, any residual lithium is converted back into active lithium ions, which are reintegrated into the cathode during charging. This enhancement improves lithium plating and stripping efficiency, resulting in enhanced cycle stability and extending the battery’s lifespan to up to 1000 cycles (Figure 9(b, c)). This approach is also adaptable to other AF-LMB systems. For instance, Li et al. added lithium iodide to a commercial ether-based electrolyte, effectively suppressing dendrite formation and reactivating lithium, thereby extending battery life.^107^

**Figure 9:**
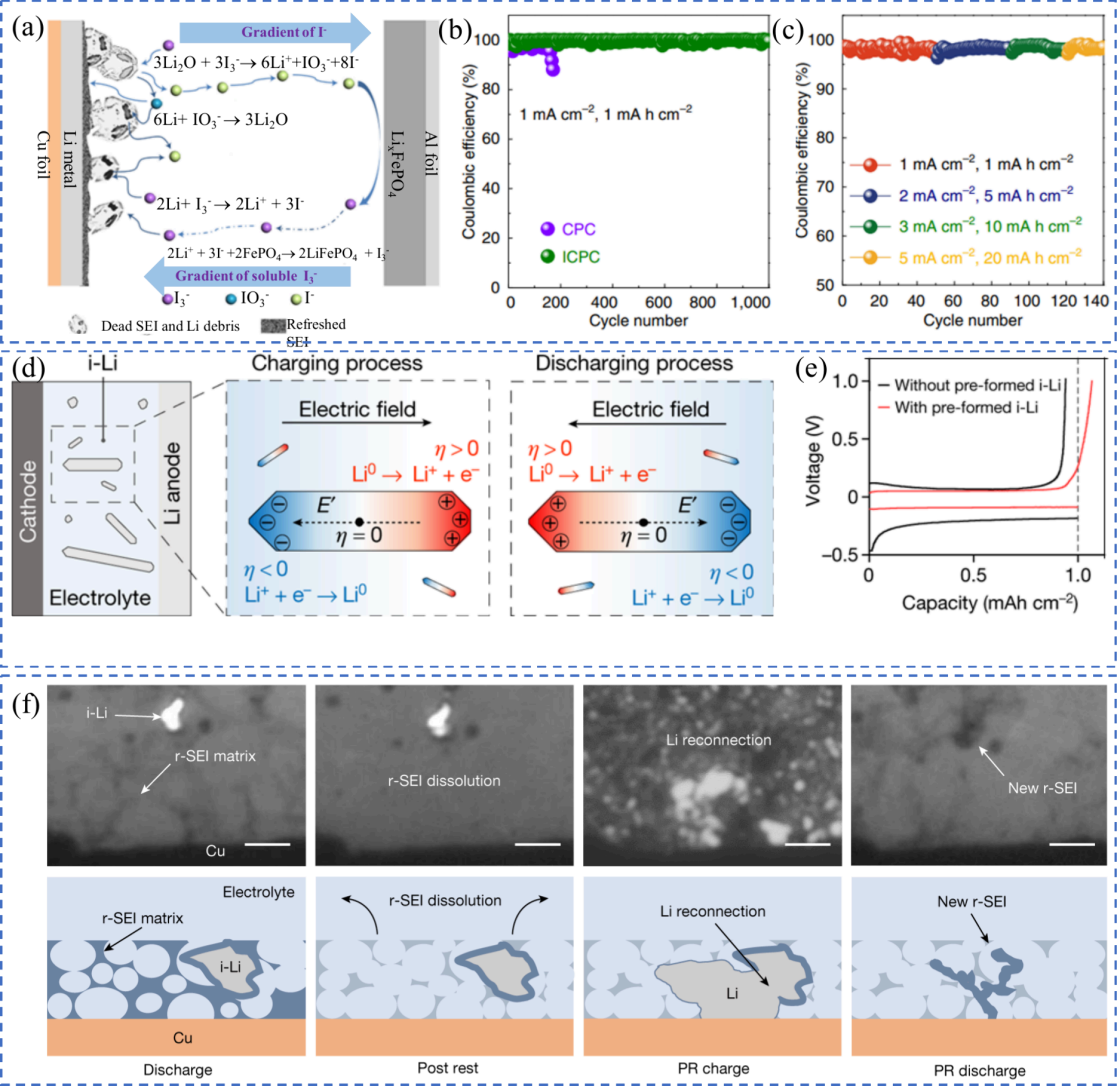
(a) Diagram illustrating the process of dead lithium recovery through repeated iodine redox reactions. (b, c) CE during extended cycling of half-cells at varying current densities. (a–c) Reproduced with permission,^106^ Copyright 2021 Springer Nature. (d) Dynamic overpotential of dead lithium under the influence of an electric field during charge and discharge cycles. (e) Voltage-capacity curves of Cu||Li half-cells, comparing cells with and without preformed dead lithium. (d, e) Reproduced with permission,^108^ Copyright 2021 Springer Nature. (f) Optical images and a diagram showing the mechanism for r-SEI dissolution and i-Li regeneration via calendar aging in the discharged state. (f) Reproduced with permission,^109^ Copyright 2024 Springer Nature.

Cui et al. took a different approach by demonstrating how isolated lithium can respond to electric fields within the battery.^108^ When exposed to an electrolyte under an electric field, the charge distribution of the inactive lithium shifts, causing high overpotential Figure 9(d)). This overpotential, driven by the local voltage difference across the lithium/electrolyte interface, leads to lithium plating where the electric field is weaker and stripping where it is stronger. This movement enables inactive lithium to migrate and reattach to the anode. In experiments with Cu||Li half-cells, rapid discharge encouraged lithium growth toward the copper electrode, resulting in a CE above 100% Figure 9(e)). The study also found that higher stripping currents improved this reconnection process, ultimately extending battery life. These findings offer new insights into enhancing cycling performance and fast-charging capabilities for AF-LMBs. Zhang et al. recently discovered that calendar aging in the discharged state can enhance capacity retention by recovering isolated lithium from previous cycles (r-SEI), as illustrated in Figure 9(f).^109^ This finding contrasts with the common phenomenon of capacity degradation typically observed during calendar aging in the charged state. The recovery of inactive lithium was confirmed by CE exceeding 100% in both Li||Cu half-cells and anode-free cells. This was achieved using a hybrid cycling protocol that combines continuous cycling with resting periods and was verified through titration gas chromatography, providing a clear indication of lithium reintegration and capacity restoration.

### 2.3 Properties of Various Metals (Activity, Migration, Diffusivities)

The design of next-generation batteries without an anode, utilizing various single- or multivalent metals (such as Ca, Zn, Al, Mg, K, Li, and Na), as listed in Table 1, is attracting considerable interest.^110–112^ The formation of the SEI on the anode surface has long been recognized as a critical factor in battery performance. Various strategies have been explored to develop SEIs for different metals. While the physicochemical properties of Na and K are similar to those of Li, they exhibit higher electronegativity, leading to differences in the modulus and solubility of their SEI formulations, as seen in Figure 10(a).^113^ Based on Mohs hardness and bulk/shear modulus, K and Na are softer than Li, suggesting a greater ability to suppress dendrite formation. In contrast, the SEI formation process for multivalent metals remains underexplored. The high modulus of Al and Zn suggests that they are more prone to severe dendrite growth. Additionally, the higher charge density of multivalent ions results in slower ion mobility within the SEI, hindering the stable development of the SEI. Theoretical calculations in Figure 10(b) reveal that Mg has the lowest diffusion barriers compared to Al, Zn, Na and Li, which supports the formation of a smoother structure. The hexagonal closest-packed structures of Mg and ZnS/ZnF_2_-Zn­(101) promote high-coordination configurations, unlike the face-centered and body-centered cubic structures seen in Li and Na.^114^

**Figure 10:**
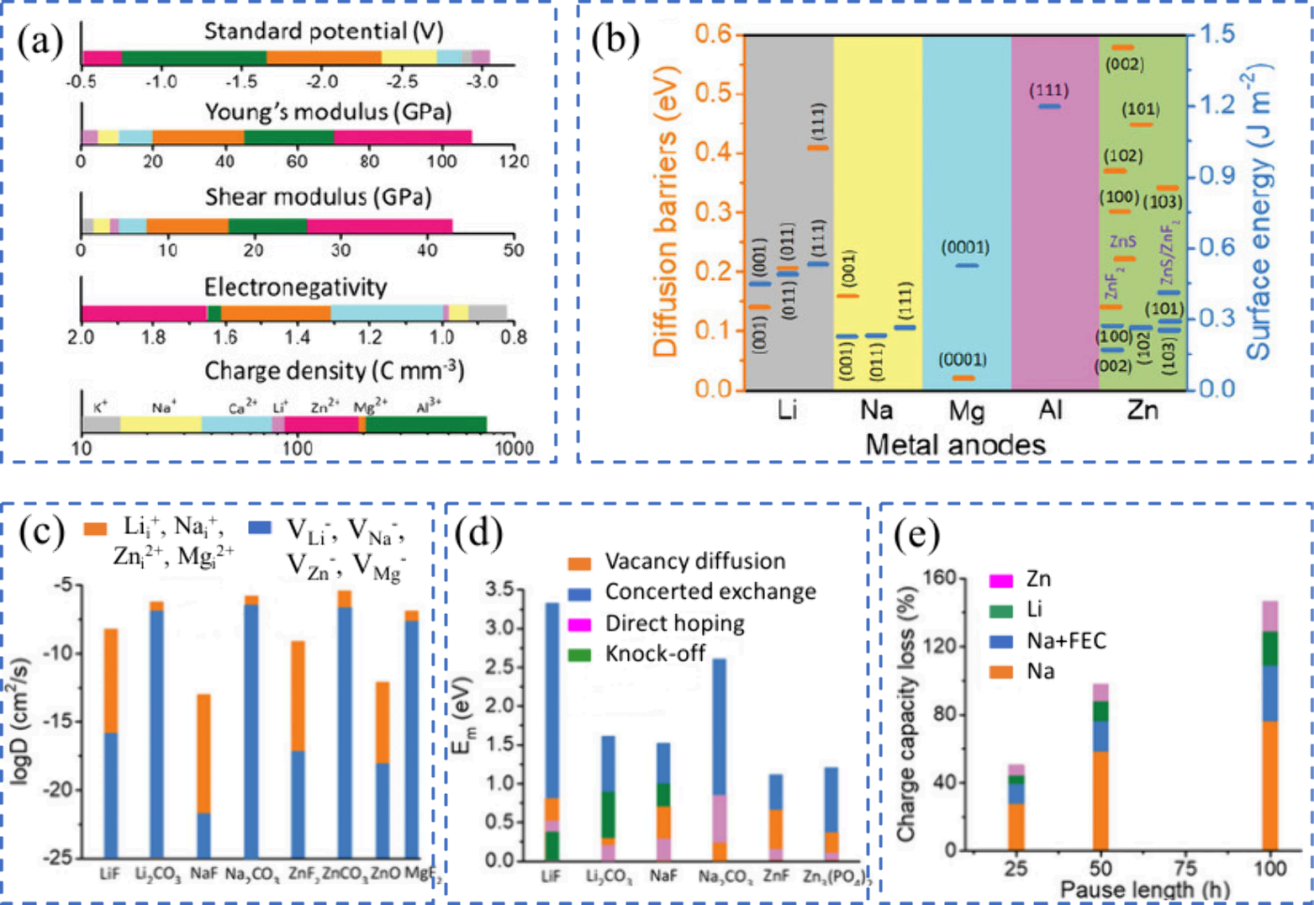
(a) Physicochemical characteristics of various metals. (b) Diffusion barriers and surface energies of different metals (Li, Na, Mg, Al, Zn) for various crystal orientations. (c) Self-diffusion coefficients for the solid-electrolyte interphase (SEI) of Li, Na, Zn, and Mg. The terms Li_i_^+^, Na_i_^+^, Zn_i_^2+^, and Mg_i_^2+^ refer to excess interstitial ions of Li, Na, Zn, and Mg, respectively. V_Li_^–^, V_Na_^–^, V_Zn_^–^, and V_Mg_^–^ represent the negatively charged vacancies of Li, Na, Zn, and Mg. (d) Migration energy barriers for different heterodiffusion processes. (e) Charge capacity loss in Li, Na, and Zn metals due to SEI dissolution. (a-e) Reproduced with permission,^136^ Copyright 2023 The Authors published by Wiley-VCH GmbH.

**Table 1:** Comparative Summary of Metal Candidates for AFB Technologies

Aspect	Li	Na	K	Mg	Al	Zn	Ca
Valency	+1	+1	+1	+2	+3	+2	+2
Gravimetric capacity (mAh g^–1^)	∼3860	∼1165	∼685	∼2205	∼2980	∼820	∼1337
Volumetric capacity (mAh cm^–3^)	∼2062	∼1130	∼487	∼3833	∼8046	∼5855	∼2073
Redox potential (vs SHE)	–3.04 V	–2.71 V	–2.93 V	–2.37 V	–1.66 V	–0.76 V	–2.87 V
Dendrite formation	Yes	Yes	Yes	Yes (limited data, under investigation)	Yes (limited data, under investigation)	Yes (more manageable in aqueous electrolytes)	Yes (limited data, under investigation)
Air/moisture stability	Poor	Poor	Poor	Moderate to good	Good	Good	Poor
Electrolyte compatibility	SEI-forming liquid/solid electrolytes	Similar to Li	Similar to Li, limited options	Requires reactive, specialized electrolytes	Requires chloroaluminate-based ionic liquids	Compatible with aqueous and some nonaqueous electrolytes	Still limited; requires tailored nonaqueous electrolytes
Plating behavior	Inhomogeneous, dendritic	Inhomogeneous, dendritic	Highly dendritic; low CE	Sluggish kinetics	High overpotential; limited reversibility	Reversible but dendritic in some conditions	Incomplete data; SEI and plating issues persist
Motivation for anode-free	Energy density, simplification	Cost, abundance, ease of sourcing	Cost, abundance, ease of sourcing	Simplification, cost (not safety-driven)	Volumetric density, cost	Simplification, aqueous system integration	Energy density, abundance, potential for multivalent use
Commercialization maturity	Advanced R&D, commercial prototyping	Early commercial interest	Early stage research	Early stage research	Fundamental research stage	Some commercial usage (Zn-ion aqueous)	Early stage research
Key challenges	Dendrites, low CE, SEI instability	Same as Li	Severe dendrites, unstable SEI, limited electrolyte stability	Electrolyte stability, kinetics	Electrolyte cost, cathode compatibility	Dendrites, shelf life, and hydrogen evolution	Interfacial instability, reversible cycling
Potential applications	Aviation vehicle, grid storage	Grid storage	Grid storage	Stationary storage	Grid storage	Grid storage, backup power	Stationary storage

Theoretical studies indicate that ZnF, NaF, and LiF promote effective ionic transport through vacancy defects Figure 10(c)). Li^+^ exhibits faster transport in LiF due to higher diffusion coefficients compared to Na^+^ in NaF and Zn^2+^ in ZnF_2_.^115,116^ Both Na^+^ and Li^+^ can migrate through their respective SEIs, Na-SEI (NaF and Na_2_CO_3_) and Li-SEI (LiF and Li_2_CO_3_), via vacancy mechanisms, knock-off, or direct hopping Figure 10(d)).^115^ The plating/stripping process is less efficient for Na, resulting in higher capacity loss, especially in carbonate-based electrolytes Figure 10(e)).^117^ Inorganic NaF and Na_2_O play a key role in achieving a high CE of 99.9% for Na plating/stripping in NaPF_6_-based diglyme electrolytes.^118^ A stable Li-SEI on the Na-metal anode (via heterostructural design) has been proposed as an approach to protect the Na-metal. Fluoroethylene or vinylene carbonate (FEC/VC), and dioxolane (DOL) exhibit similar chemical properties for both Na and Li. At the same time, NaNO_3_ has a detrimental effect on Na compared to the more favorable LiNO_3_ for Li, due to extensive electrolyte decomposition. In Li/Na hybrids, the more negative redox potential of Li/Li^+^ provides a protective effect for Na^+^, helping to manage the interphase dynamics for denser electroplating.^119^ K, the softest metal among the anodes, shares properties with Na and Li, but its high reactivity with solvents leads to an unstable SEI even at low current densities (0.2 mA cm^–2^). However, highly concentrated electrolytes can effectively stabilize the SEI for K.^120^

High overpotential arises due to the combination of a small ionic radius and a high charge number. Aluminum can undergo reversible plating and stripping in ionic liquids, such as AlCl_3_ mixed with 1-ethyl-3-methylimidazolium chloride, which are highly corrosive.^121^ The use of artificial SEI allows the operation of Al and Mg in both aqueous and nonaqueous electrolytes, demonstrating the crucial role of SEI in multivalent metals.^122–124^ Zinc’s compatibility with water enhances its intrinsic safety, although issues such as dendrite formation and volume change hinder its commercial application for larger-scale use.^125^ Zinc typically forms a Zn_5_(CO_3_)_2_(OH)_6_ layer under ambient conditions, which is easily oxidized in mild acids and converts to a movable interphase of Zn_4_SO_4_(OH)_6_·*x*H_2_O, leading to low CE and severe dendritic growth.^126^ In alkaline media, the formation of soluble ZnO_2_^2–^ and irreversible ZnO on the anode causes structural distortions. To protect the zinc anode, approaches such as cation-selective membranes, polymer-based additives, epitaxial electroplating with reduced lattice mismatch, and artificial ZnS/ZnF2-based SEI have been employed.^125,127^

The SEI, or passivation film, plays an essential role in allowing the diffusion of metal ions (such as Al^3+^, Ca^2+^, K^+^, Zn^2+^, Mg^2+^, Na^+^, and Li^+^) through a layer under a uniform electric field, thereby reducing overpotential. This helps prevent the aggregation of electrochemically active species, maintaining uniform chemical compositions in the electrodes. However, as the SEI or passivation layer thickens, it can increase the internal resistance of the cell by consuming metal ions from the cathode, which reduces both capacity and power. Under carbonate-based electrolytes, the SEI forms at around 1 V versus Li/Li^+^ or Na/Na^+^ for metals, oxides, or carbons. Anode materials used in K-storage typically have voltage ranges of around 0–1 V.^128^ For instance, K metal (0 V), graphite (≈0.1 V), alloys (≈0.8 V), and red phosphorus (≈0.7 V) exhibit a higher Fermi energy than the LUMO.^129–131^ The mixed ionic and electronic conduction behavior of the SEI and CEI layers can negatively impact the overall performance of the cell. Using inert materials to protect the anode and cathode has shown promise in reducing chemical reactions. Interphase reactions are crucial for maximizing the potential of batteries, where factors such as electronegativity, contact conditions, and interphase structures strongly affect the overall energy output of the cells.^132^

Cation–anion or cation–solvent interactions play a crucial role in determining the reduction stability of solvents and anions, as they affect the solvation structures and the coordination numbers of solvents around cation centers.^120,133,134^ When cations bind to solvents or anions, their LUMO levels decrease because electron pairs are donated to the cations. This interaction leads to the formation of ion pairs and solvation, which can promote the decomposition of electrolytes. Density functional theory (DFT) calculations show that ion–solvent complexes, such as those with FEC, propylene carbonate (PC), diethyl carbonate (DEC), ethylene carbonate (EC), 1,2-dimethoxyethane (DME), and 1,3-dioxolane (DOL), have lower LUMO levels compared to the pure solvents. The reduction in LUMO level for carbonate solvents follows the trend: Li^+^ > Na^+^ > K^+^, which correlates linearly with binding energy. In contrast, for ether solvents, the LUMO level shifts are more pronounced in the order of Na^+^ > K^+^ > Li^+^.^134^ The formation of the SEI and the reduction stability of electrolytes are influenced by the interaction of cations with electrolytes, emphasizing the need to understand the differences in the SEI layer formation across different battery chemistries, such as those involving Na, Li, K, Zn, Mg, Al, and Ca.

From an electronic structure viewpoint for aqueous batteries, water behaves as an oxide with an *E*_g_ of 8.7–8.9 eV. If the difference between the LUMO and HOMO reflects the electrochemical window, water could serve as a solvent in Na, K, or Zn-ion batteries. The thermodynamic potential of water is 1.23 V, which sets limits for hydrogen evolution at −4.02 eV and oxygen evolution at −5.25 eV (equivalent to 0 V vs SHE at pH 7), corresponding to an energy level of −4.44 eV on the absolute scale (where electron energy is 0 eV in a vacuum). However, no direct correlation exists between the LUMO–HOMO energy gaps of water and the Fermi levels of electrons during water oxidation and reduction in solutions.^135^ The actual chemical potentials are influenced by surface potentials.

From a commercialization perspective, Li and Na anode-free systems are furthest along in development. The battery industry already possesses robust manufacturing infrastructure for Li-ion and Na-ion technologies, enabling smoother integration of anode-free designs. Moreover, companies such as Sion Power, QuantumScape, and others are actively pursuing lithium metal and AFB platforms. In contrast, Mg, Al, Ca, and Zn systems are generally less mature. Mg and Al face major obstacles in electrolyte chemistry and cathode development, which limit their viability for anode-free designs despite their intrinsic advantages. Ca and Zn offer specific performance and safety benefits, but both require further progress in interfacial control and plating/stripping efficiency to be considered practical for anode-free cells. Zn, however, is unique in its proximity to commercial application, particularly in nonflammable, aqueous-based systems.

In summary, while anode-free configurations offer a path to higher energy density and cost-effective battery systems, the benefits and feasibility of this approach vary significantly across different metal chemistries. For Li and Na, the primary motivations are improving energy density and safety by eliminating dendrite-prone excess metal. For Mg, Al, and Ca, the motivation lies more in simplifying cell architecture and reducing material costs, though significant electrolyte and interfacial challenges must first be addressed. Zn, although facing its own issues, represents the most commercially mature of the alternative metal systems. A deeper understanding of the electrochemical behavior of each metal, along with targeted materials and interface design strategies, will be essential in advancing anode-free technologies across this broader range of chemistries. Future work should also incorporate comparative techno-economic and lifecycle assessments to guide research priorities and identify the most promising directions for industrial-scale implementation.

## 3 Parameters and Performance in Anode-Free Batteries

In AFBs, optimizing electrochemical performance goes beyond the choice of materials and electrolytes. Measurement protocols also play a crucial role.^137,138^ Parameters such as current density, cutoff voltage, pressure, temperature, and mass loading significantly impact key processes, including SEI formation, electrolyte stability, and the quality of metal deposition. High current densities can lead to nonuniform metal plating and the formation of dendrites. In contrast, low current densities promote uniform growth of the SEI. The cutoff voltage affects electrolyte decomposition and SEI stability, with improper limits causing adverse side reactions. Pressure is crucial in maintaining interfacial contact, influencing deposition uniformity and dendrite suppression; however, excessive pressure can cause structural damage. Temperature affects ion transport and reaction kinetics, where high temperatures enhance performance but can also accelerate degradation. Mass loading influences ion diffusion, impacting the uniformity of deposition and the quality of the SEI. Balancing these protocols is essential for optimizing the electrochemical performance and longevity of AFBs.

### 3.1 Critical Parameters

#### 3.1.1 Coulombic Efficiency

The main factor affecting AFBs’ performance so their CE reflects is the CE of metal plating, which measures how well a metal is reversible during each cycle. CE is the ratio of stripped metal to plated metal. Ideally, without side reactions, this process would be fully reversible with no ″dead metal″ formed. However, in reality, the reactive plated metal anode forms an SEI layer as it reacts with the electrolyte, leading to a loss of active metal.^139^ The anode’s volume expansion and dendrite growth during cycling further damage the SEI, allowing it to come into contact with fresh metal and react with the electrolyte, consuming even more metal. Improving the CE of plated metal is critical to overcoming these challenges.

Xiao et al. studied the relationship between metal plating CE and cycle life in lithium-metal batteries.^140^ Figure 11(a) shows four common cell configurations used for performance evaluation: Li||Cu, Li||Li, Cu||NCM811, and Li||NCM811. In Li||NCM811 cells, lithium is supplied from both the anode and cathode, ensuring that CE is always 100% if there’s excess lithium, thereby preventing lithium loss. Cu||NCM811 cells, however, only receive lithium from the cathode, so their CE reflects the lithium loss during cycling. Li||Cu half-cells assess lithium loss by comparing stripped and deposited lithium, while Li||Li symmetric cells, with excess lithium on both sides, always show 100% CE and are more useful for studying dendrite growth prevention in separators or electrolytes. Figure 11(b, c) compares the cyclability of Li||NCM811 cells with two electrolytes: 1.2 M LiFSI in TEP: BTFE (1:3 mol/mol, TEP = triethyl phosphate; BTFE = bis­(2,2,2-trifluoroethyl) ether) and 1.0 M LiPF_6_ in EMC/EC (7:3 wt/wt +2% VC). Both showed high initial CE over 99%, but the cell with 1.0 M LiPF_6_ degraded after 55 cycles, while the one with 1.2 M LiFSI TEP remained stable for over 200 cycles. This suggests that when excess metal is present, CE does not accurately represent electrolyte performance. However, with limited metal, performance drops quickly in low-CE electrolytes. Figure 11(d, e) shows that in Cu||NCM811 cells without excess lithium, the cycle life directly depends on CE. With a CE of 97.79%, the cells cycled over 100 times; however, at a CE of 92.31%, their capacity dropped to zero after 38 cycles. In general, because AFBs lack excess metal inventory, their cycle life is directly correlated with the CE of metal plating/stripping. To enhance the cyclability of these batteries, a CE greater than 99% must be achieved.

**Figure 11:**
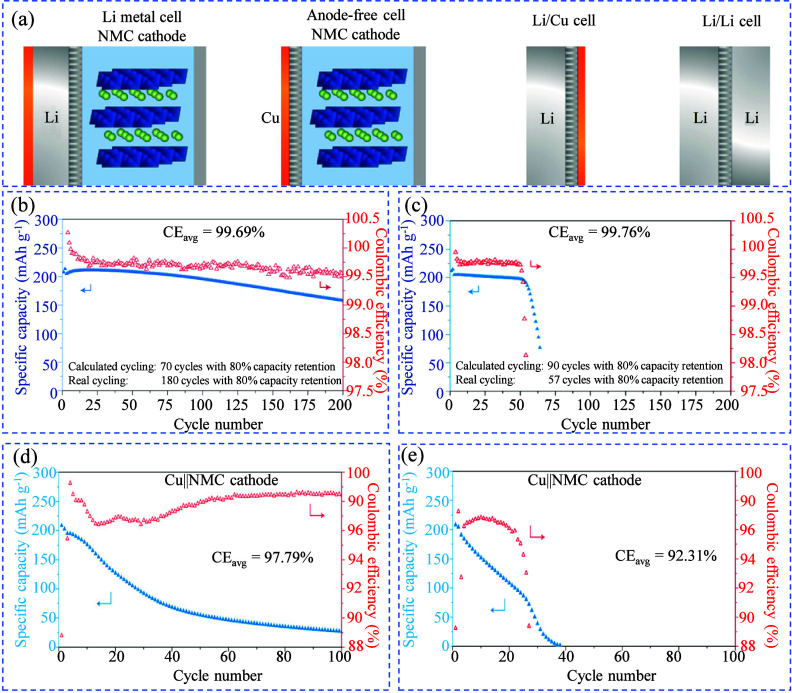
Effects of CE on AF-LMBs: (a) Configurations of various lithium-metal battery types. (b) Cyclability and CE of Li||NMC811 coin cells using 1.2 M LiFSI in TEP: BTFE (1:3 mol/mol). (c) Cyclability and CE of Li||NMC811 coin cells with 1.0 M LiPF_6_ in EMC/EC (7:3 wt/wt) + 2 wt % VC. (d) Cyclability of Cu||NCM811 cells with 1.2 M LiFSI in TEP: BTFE (1:3 mol/mol). (e) Cyclability of Cu||NCM811 cells with 1.0 M LiPF_6_ in EMC/EC (7:3 wt/wt). (a–e) Reproduced with permission,^140^ Copyright 2020 Nature Publishing Group.

While the studies effectively demonstrate the dependence of cycle life on CE in AFB configurations, future research must address the persistent challenges of dendrite formation, SEI fracture, and continuous metal loss. Additionally, the use of standardized testing protocols for comparing CE across different cell configurations and electrolyte systems would be vital to establishing meaningful benchmarks. Finally, achieving such high CE in real-world conditions, not just in controlled laboratory settings, should be a primary focus, as this directly impacts the commercial viability of AFBs.

#### 3.1.2 Electrolyte-to-Cathode (E:C) Ratio

The E:C ratio is a critical parameter influencing the electrochemical performance, cycling stability, and practical energy density of AFBs. Unlike conventional battery systems, AFBs are particularly sensitive to electrolyte depletion due to their reliance on in situ metal plating and the continuous formation and repair of the SEI. The reductive decomposition of electrolyte components forms the SEI, and its repeated regeneration consumes both solvent and metal salt. Consequently, the electrolyte plays not only a transport medium role but also serves as a finite reactant reservoir that is gradually exhausted during cycling.

Usually, excess electrolyte is used in metal-based batteries to mitigate rapid degradation and extend cycle life. However, a high E:C ratio reduces the effective energy density, counteracting one of the key motivations for AFBs, which is to achieve high gravimetric and volumetric energy densities through the elimination of excess metal and minimal inactive materials. Zhang et al. systematically studied the impact of E:C ratio on the cycling stability of both Cu||Li half-cells and Li||NMC811 full cells by varying the electrolyte volume at a given cathode capacity.^140^ In the Cu||Li system, a high E:C ratio (∼75 μL) enabled stable cycling for over 800 h, whereas reducing the electrolyte to 15 μL (significantly lower E:C ratio) curtailed the lifespan to approximately 180 h Figure 12(a)). In full-cell configurations (Li||NMC811), a high E:C ratio allowed the cell to cycle for over 50 cycles with a high CE of 99.92%. Conversely, reducing the electrolyte volume resulted in early capacity decay and a sharp decline in CE after only 30 cycles, indicating rapid electrolyte depletion Figure 12(b)).

**Figure 12:**
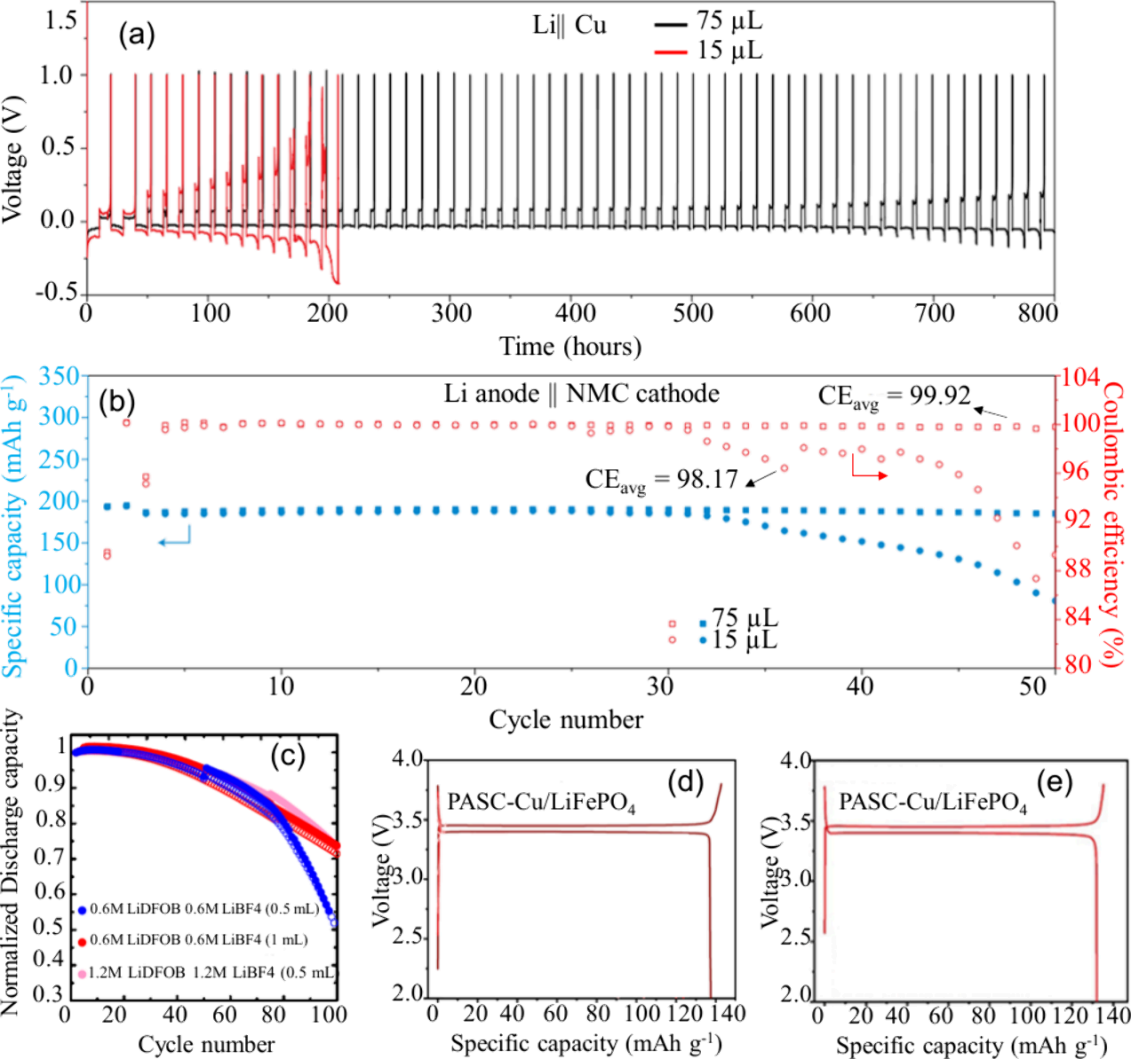
Impact of electrolyte quantity on the electrochemical performance of cells: (a) Cu||Li cell; (b) NMC811||Li cell. (a, b) Reproduced with permission,^140^ Copyright 2020 Nature Publishing Group. (c) Relationship between normalized discharge capacity and cycling performance for cells with varying LiDFOB amount, adjusted by increasing salt concentration or volume of electrolyte. (c) Reproduced with permission,^141^ Copyright 2019, Nature Publishing Group. Typical charge/discharge profiles of PASC-Cu||LiFePO4 AFBs under different conditions: (d) flooded electrolyte and (e) lean electrolyte, (d, e) Reproduced with permission,^142^ Copyright 2024, Wiley-VCH GmbH.

Similarly, Weber et al. explored pouch cell configurations using a dual-salt electrolyte (0.6 M LiBF_4_ + 0.6 M LiDFOB in FEC, 2:1 v/v).^141^ As shown in Figure 12(c), increasing the electrolyte volume from 0.5 to 1 mL at a given cathode capacity, thus increasing the E:C ratio, led to enhanced capacity retention, suggesting that a higher E:C ratio can delay the onset of electrolyte exhaustion and maintain SEI integrity over prolonged cycling. However, this improvement came at the cost of reduced energy density.^15^ Ouyang et al. further demonstrated the trade-off between E:C ratio and cell performance using a PASC-Cu||LiFePO_4_ AFB cell.^142^ The cells were assembled with both flooded and lean electrolyte conditions, the latter corresponding to an E:C ratio of 2 gA h^–1^. Under flooded conditions, the cell achieved a discharge capacity of 138 mAh g^–1^, while the lean condition still delivered a comparable 132 mAh g^–1^ at a C/10 rate, as shown in Figure 12(d, e). This result underscores that with appropriate interfacial engineering, high performance can be retained even at lower E:C ratios.

Overall, these findings highlight the delicate balance between E:C ratio, SEI stability, electrolyte consumption, and energy density. While lowering the E:C ratio improves the cell’s gravimetric and volumetric energy metrics, it also increases the risk of premature failure due to accelerated electrolyte degradation. A low E:C ratio amplifies the importance of minimizing parasitic reactions and designing robust SEI layers that do not rely on continuous electrolyte decomposition for self-repair. Therefore, achieving practical AFBs necessitates strategic electrolyte management: ensuring sufficient ionic transport and interphase stability at low E:C ratios without compromising the energy density. Optimizing the E:C ratio is not merely a volumetric consideration but a core challenge in balancing electrochemical longevity and high energy output in next-generation metal battery systems.

#### 3.1.3 External Pressure

Recent studies have highlighted the critical role of external pressure in enhancing the performance of metal batteries. High external pressure effectively suppresses both volume expansion and metal dendrite growth, resulting in significant improvements in metal plating efficiency and cycle life of AFBs.^143–145^ In lithium-based systems, external pressure helps maintain intimate contact between the deposited lithium and the CC, promoting uniform Li nucleation and mitigating the formation of porous or dendritic structures. This mechanical constraint also stabilizes the SEI, reducing interfacial degradation and enhancing CE. For instance, the research by Dahn’s group demonstrated the influence of external pressure on the capacity retention of Cu||NMC532 AFBs Figure 13(a)).^79^ Their findings showed that batteries subjected to 1200 kPa, a higher pressure, exhibited much better capability in retaining capacity compared to those under lower pressure (75 kPa). To further optimize performance, Dahn’s group introduced a “mechanical pressure” protocol Figure 13(b)).^146^ They investigated the electrochemical performance of pouch AFBs configured as Cu||NMC532 subjected to different amounts of stack pressure, ranging from 75 to 2205 kPa, using two electrolyte systems: 1 M LiPF_6_-TFEC/FEC and 1 M LiPF_6_-DEC/FEC. The results revealed a remarkable improvement in performance as the pressure used rose from 75 to 1200 kPa. However, applying a moderately high pressure was found to be most effective for enhancing the performance of AFBs. These findings underscore the importance of carefully tuning external pressure to optimize the electrochemical performance of AFBs. While increased pressure can significantly enhance metal plating efficiency, suppress dendrite formation, and improve cycle life, excessively high pressures may pose mechanical challenges or diminishing returns. Therefore, identifying an optimal pressure window is essential for achieving stable and high-performance AFB operation. Future work should focus on balancing mechanical design with electrochemical requirements to develop practical pressure management strategies for scalable AFB technologies.

**Figure 13:**
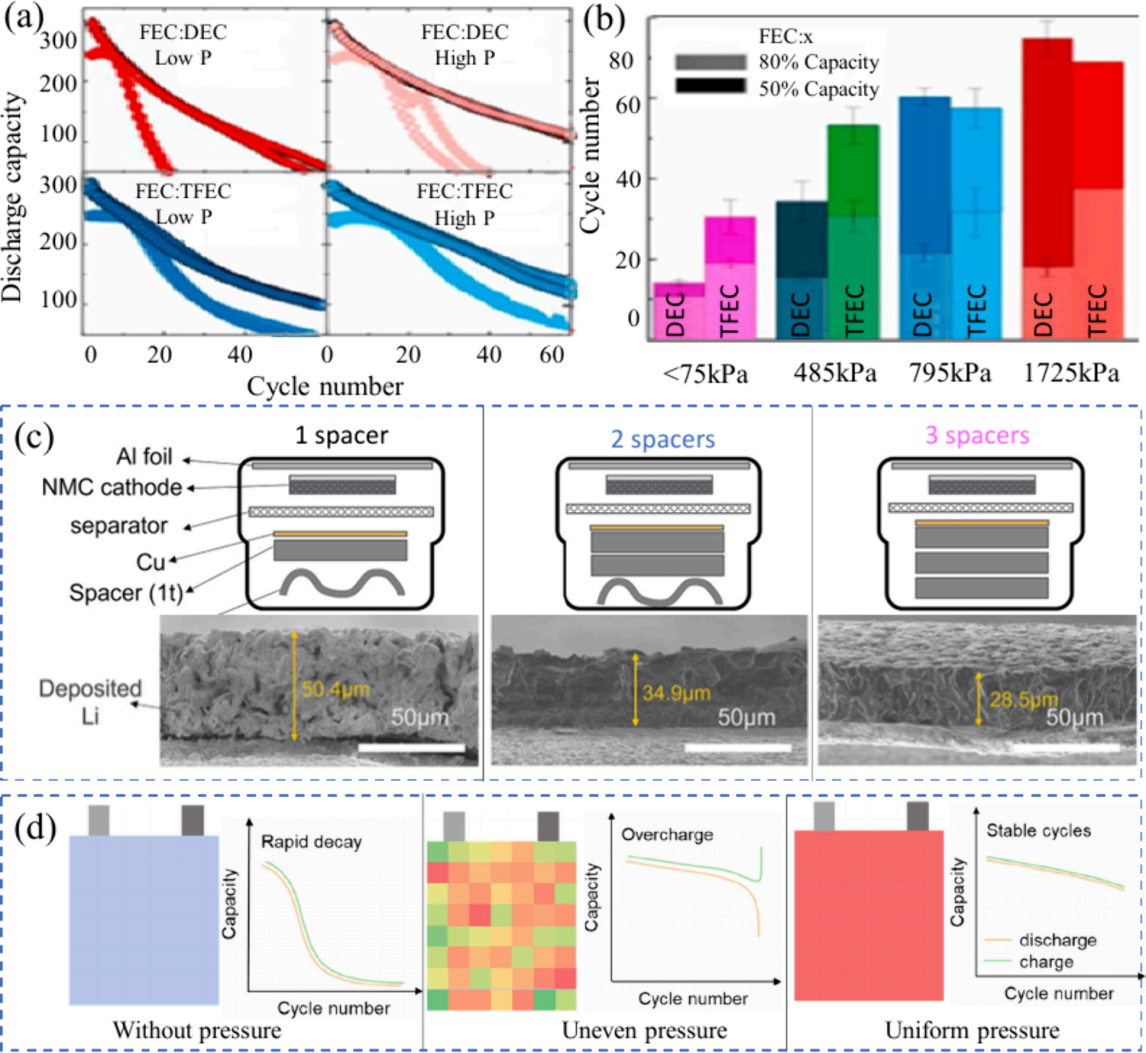
(a) Capacity retention comparison of Cu||NMC532 AFBs under different pressures and electrolytes. Reproduced with permission,^79^ Copyright 2018 The Electrochemical Society. (b) Illustration of pressure impact on capacity. Reproduced with permission,^146^ Copyright 2019 The Authors published by The Electrochemical Society. (c) A schematic diagram showing the assembly of coin-cell-based AFBs with varying numbers of stainless-steel spacers, along with their corresponding cross-sectional SEM images of the initial lithium plating. Reproduced with permission,^147^ Copyright 2023 American Chemical Society. (d) A schematic representation of the cell capacity model under different pressure conditions. Reproduced with permission,^148^ Copyright 2024 American Chemical Society.

Recent studies have investigated the effect of internal pressure on the efficiency of AF-LMBs, aiming to optimize cycling stability and capacity retention. Lim et al. investigated the application of optimized internal pressures on AF-LMB coin cells.^147^ By using a different number of spacers to increase the internal pressure Figure 13(c)) and employing a refined cycling protocol, they demonstrated that the formation of a thin, uniform layer of nucleation sites in the first cycle promotes smooth lithium plating/stripping in the cycles that follow. This process, combined with an optimized pressure distribution, results in a stable SEI layer, thereby enhancing long-term cycling performance. Their work demonstrated that AF-LMBs (Cu||NMC811) retained 72% of their capacity after 100 cycles under these conditions. Similarly, Yang et al. developed a pouch AFB with a higher energy density (415 Wh/kg) by integrating a LiCoO_2_ cathode and a Cu foil anode.^148^ Their findings indicated that applying high pressure significantly increases lithium deposition density, nearly 3-fold, but can result in overcharge if the pressure is unevenly distributed, as demonstrated in Figure 13(d). By incorporating a soft layer to disperse pressure, they were able to densify lithium deposition further and mitigate overcharge, achieving 70% capacity retention after 100 cycles for the 2.22 Ah anode-free pouch cell. This approach offers promising insights for scaling the performance improvements to practical lithium-metal batteries. These findings highlight the essential role of pressure optimization in enhancing the cyclability and capacity retention of AFBs, underscoring its potential for advancing high-energy-density battery technologies.

Sodium and potassium-based AFBs, which suffer from even more pronounced dendrite formation and interfacial instability due to their higher reactivity and larger ionic radii, likewise benefit from external pressure, though care must be taken to avoid excessive stress that may crush soft metal deposits or crack brittle interfaces.^149–151^ In divalent and trivalent systems such as magnesium and aluminum, which exhibit sluggish kinetics and strong surface passivation rather than dendrite growth, external pressure serves to improve interfacial contact and facilitate more uniform metal deposition, particularly when using solid or gel polymer electrolytes.^152,153^ Similarly, zinc metal batteries, especially those operating in aqueous or semisolid environments, respond favorably to moderate applied pressure, which suppresses dendritic protrusions and enhances reversibility. In the case of anode-free ASSBs, external pressure is indispensable for maintaining consistent physical contact between the solid electrolyte and the electrodes, thereby minimizing interfacial voids and contact resistance during cycling.^154–156^ As many solid electrolytes are brittle and prone to delamination or cracking under volume change, a sustained stack pressure is often necessary to enable stable interfacial dynamics.

Overall, the electrochemical performance of AFBs is greatly affected by external pressure. The external pressure impacts the morphology of metal deposition, which, in turn, can enhance capacity retention in two key ways: (i) by preventing dendrite formation, thus reducing the amount of electrically isolated metallic lithium or sodium or other metal that cannot be used, and (ii) by lowering the surface area of deposited metal, which limits unwanted interactions with the electrolyte that consume active metal and contribute to the formation of an SEI. This understanding highlights that optimizing external conditions is a promising strategy for improving the cycle life and CE of AFBs.

#### 3.1.4 Formation Protocols

The formation stage is a critical determinant of metal morphology, SEI structure, and long-term performance in AFBs. Unlike conventional LIBs that benefit from slow formation protocols optimized for intercalation anodes, recent studies have shown that AFBs require distinct strategies tailored to their metal plating/stripping dynamics.^157,158^ Kim et al. systematically investigated the effect of formation current density (CD) on CE and capacity retention in Cu∥NMC811 AFBs (Figure 14(a, b)).^159^ Their results revealed that increasing the formation CD up to C/2 enhanced cycling stability, with CE improving significantly compared to the traditional slow formation at C/20. Specifically, formation at C/2 resulted in more uniform metal plating as illustrated in Figure 14(c), minimized exposed CC areas after stripping, and formed a mechanically robust, inorganic-rich SEI. Despite lower initial CE at higher CDs due to the increased surface area for SEI formation, subsequent cycles demonstrated improved retention and less metal loss, indicating that the dominant role of SEI and morphology formed during the initial cycle in dictating long-term behavior.

**Figure 14:**
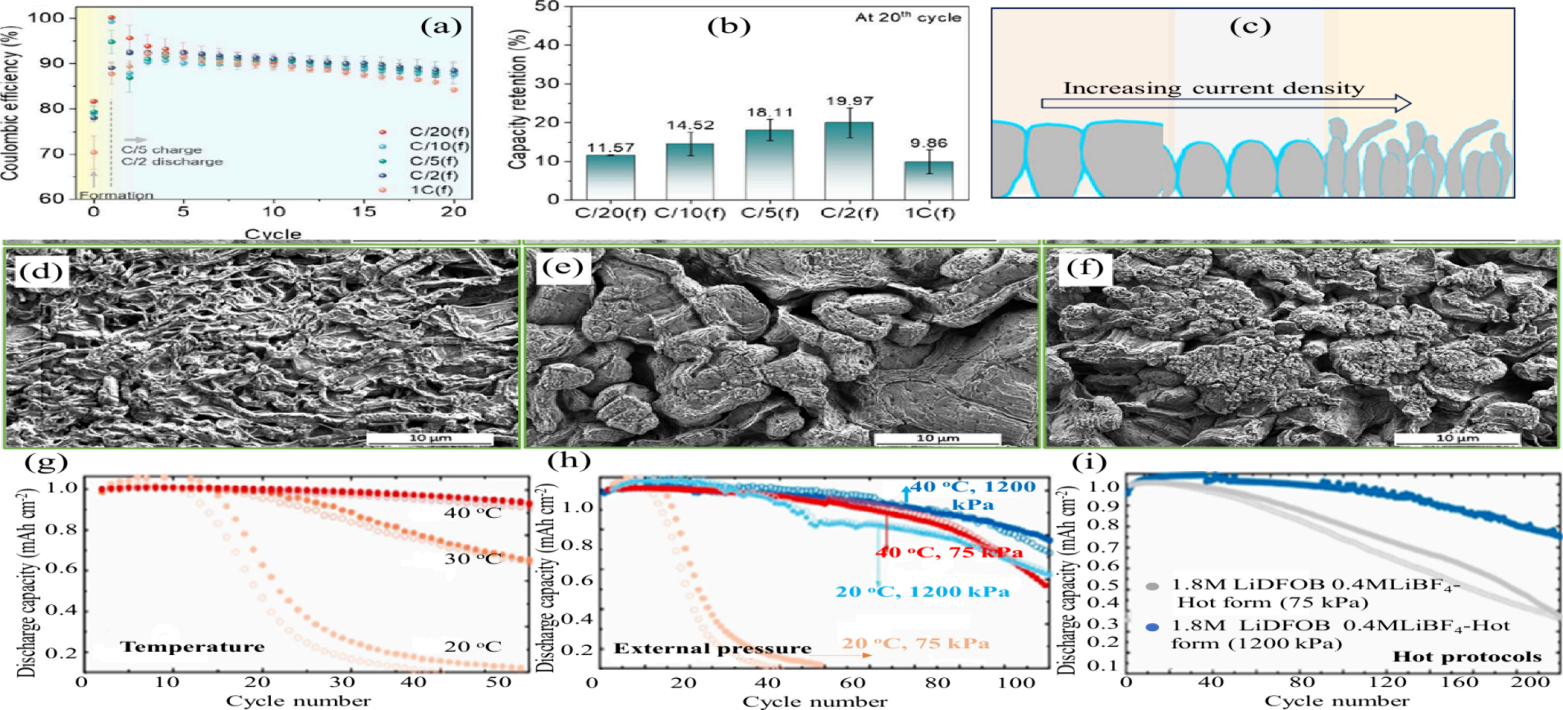
Effect of formation current in AFBs. (a) CE of AFBs and (b) Capacity retention after the 20th cycle of AFBs, (c) illustration of the relationship between the initial charging current density and the morphology characteristic of the plated metal. (a–c) Reproduced with permission,^159^ Copyright 2024 The Authors, published by American Chemical Society. Morphology of metallic Li after pulsed current charging at C/8 (d) with *t*_on_ = 1 s, *t*_off_ = 2 s (e) C/5 with *t*_on_ = 0.25 s, *t*_off_ = 0.75 s and (f) C/2 with *t*_on_ = 0.25 s, *t*_off_ = 0.75 s. (d–f) Reproduced with permission,^160^ Copyright 2024 The Authors, published by The Royal Society of Chemistry. (g) Cyclability of Cu||NMC532 AFBs at varying temperatures, (h) under different pressures, and (i) using “hot protocols”. (g–i) Reproduced with permission,^161^ Copyright 2019, The Author(s) Published by The Electrochemical Society.

Complementing this, Cicvarić et al. demonstrated that pulsed current formation protocols can further enhance the quality of metal plating in AFBs.^160^ Their SEM images in Figure 14(d-f) compared lithium morphology after formation using both constant-current and pulsed-current protocols. Pulsed protocols (e.g., C/2, 0.25 s on/0.75 s off) yielded dense, compact, “rock-like” lithium deposits with reduced surface roughness and fewer needle-like structures, which are typically precursors to dendritic growth. In contrast, constant-current protocols, particularly at higher rates (C/5), led to rough, porous, and irregular morphologies. These morphological improvements translated into reduced interfacial resistance and higher CE, as smoother deposits presented less reactive surface area to the electrolyte, thereby reducing parasitic side reactions.

Temperature also significantly influences several key properties of electrolytes in AFBs, including their conductivity, viscosity, and diffusion coefficient. It also affects the formation and stability of the SEI layer, which plays a crucial role in overall battery performance. For example, Dahn’s research team introduced a “hot formation” protocol, which involves two initial cycles, at 40 °C, charging at C/10 and discharging at C/2.^161^ Before this, they systematically investigated the effects of temperature and external pressure (Figure 14(g, h)). This strategy led to notable improvements in capacity retention. Moreover, the combination of high external pressure with the hot formation protocol further enhanced the performance of the AFBs (Figure 14(i)).

Overall, the formation protocol is a pivotal factor influencing the initial interfacial chemistry and long-term cycling performance across a wide spectrum of AFB chemistries, including those based on Li, Na, K, Mg, Al, and Zn. Each metal presents unique challenges, such as differing redox potentials, ion mobility, and SEI-forming tendencies, all of which necessitate metal-specific formation strategies. For instance, the more reactive nature of alkali metals like K and Na may require milder conditions to suppress dendritic growth, whereas multivalent systems like Mg, Zn, and Al demand protocols that mitigate sluggish plating kinetics and promote compact metal deposition. Furthermore, in the context of ASSBs, where the formation of stable solid–solid interfaces is paramount, thermal and mechanical parameters during the initial cycles are even more critical. Recent advancements highlight the importance of optimized current densities, pulsed formation conditions, and elevated temperatures to facilitate uniform metal nucleation and robust SEI formation. These tailored approaches not only improve CE and capacity retention but also suppress adverse morphologies across diverse battery chemistries. As AFB technology evolves, the rational and chemistry-specific design of formation protocols will be indispensable for enabling safe, high-energy-density, and commercially viable energy storage systems.

#### 3.1.5 Cycling Protocols

The charging and discharging protocols of LMBs greatly impact the overall performance. One critical factor is current density, which impacts the quality of lithium plating by affecting the stability of the SEI layer, the bulk ion concentration, and the Li^+^ ion mass transfer rate.^162,163^ Studies have consistently demonstrated the pivotal role of current density in shaping the electrochemical performance of AFBs. For example, Seong et al. developed a strategy that illustrates the correlation between current density, discharge capacity, and lithium dendrite growth (Figure 15(a)).^164^ The findings suggest that dendrite formation can be mitigated by using a smaller current density and extending deposition times. Louli et al. extended the understanding of how charging/discharging rates influence cyclability through both asymmetric and symmetric cell designs Figure 15(b)).^78^ Their study revealed that asymmetric protocols, where charging is slower than discharging, are particularly effective in reducing lithium inventory loss during the plating/stripping process. Specifically, using a charging rate 2.5 times slower than the discharging rate allows for faster charging without significantly degrading performance, as long as the discharge rate remains higher (Figure 15(c, d)).

**Figure 15:**
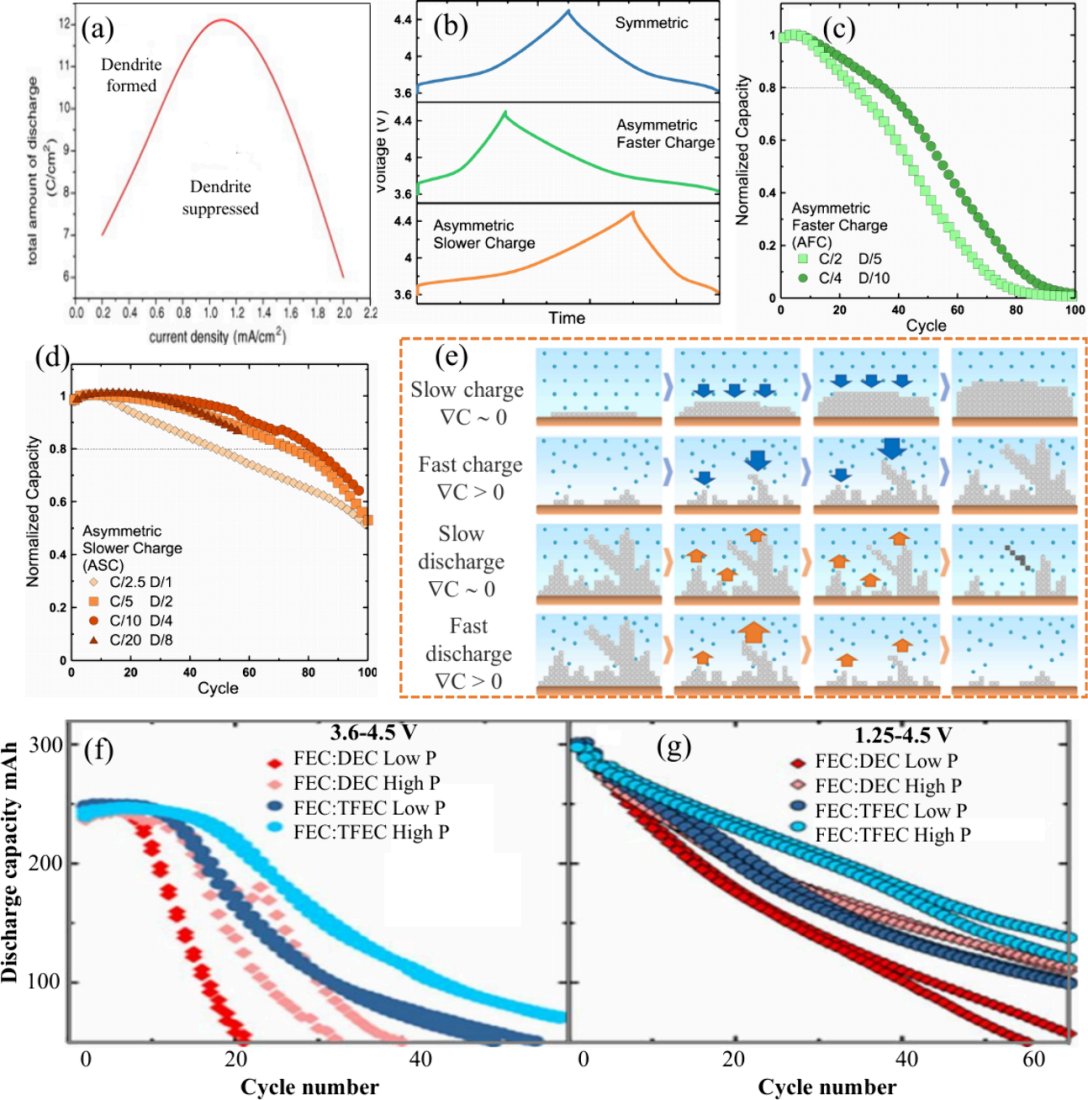
(a) Correlation between current density, discharge capacity, and Li dendrite growth. Reproduced with permission,^164^ Copyright 2007 Elsevier. (b) Time vs voltage profile of three charging and discharging protocols: symmetric charging and discharging, asymmetric faster charging, and asymmetric slower charging. (c) Performance of an asymmetric protocol with a faster charge. (d) Performance of an asymmetric setup using slower charging, (e) schematic of metal plating and stripping mechanisms under fast and slow charge/discharge conditions. (b–e) Reproduced with permission,^78^ Copyright 2021 The Author(s) published by IOP Publishing Limited. Capacity retention of Cu||NMC532 pouch AFBs with a voltage range of (f) 3.6–4.5 V and (g) 1.25–4.5 V. (f, g) Reproduced with permission,^79^ Copyright 2018 The Electrochemical Society.

The rates at which a battery is charged and discharged critically influence the metal plating and stripping processes, as illustrated in Figure 15(e). During fast charging, the application of high current induces localized current densities and steep metal-ion concentration gradients (∇*C*), which promote uneven metal plating and the growth of porous, mossy structures. As discussed in Sections 2.1.2 and 2.2, this behavior is further exacerbated by low Ω_ct_ in certain regions of the electrode–electrolyte interface. These areas facilitate faster local metal plating. Simultaneously, the diffusion resistance Ω_D_ increases due to the tortuous, high-surface-area morphology of the SEI, which worsens mass transport and amplifies nonuniform deposition. In contrast, slow charging results in lower ∇*C* and higher Ω_ct_, which collectively promote uniform metal plating and the formation of large, stable metal grains or columnar structures.

During slow discharge, the concentration gradient approaches zero (∇*C* ≈ 0), resulting in a high Ω_ct_. This results in relatively uniform stripping across the electrode surface but does not preferentially remove metal protrusions, leaving behind isolated “dead” metal and contributing to increased inventory loss. Conversely, fast discharge generates a steep ∇*C* and a condition where Ω_ct_ ≪ Ω_D_, favoring tip stripping. This preferential removal of protruding metal effectively smooths the metal surface and reduces residual metal accumulation. Overall, the interplay between current density and electrochemical resistances governs the evolution of metal morphology. A slow charge-fast discharge protocol optimally balances these effects by promoting uniform metal plating and efficient stripping, thereby minimizing metal loss and enhancing overall battery performance.

The voltage window is another critical factor that directly impacts capacity retention.^79^ On one hand, it influences the oxidative stability of electrolytes. Extremely high cutoff voltages accelerate electrolyte decomposition, producing gas through parasitic reactions, which reduces capacity retention and shortens the battery’s lifespan. On the other hand, the potential window affects the reversibility of metal plating/stripping on the CC. Dahn’s group conducted a detailed analysis using Cu||NMC532 AFB configuration to investigate the effects of discharge cutoff voltage on capacity fading (Figure 15(f, g)).^79^ Their findings show that extending the voltage window from 3.6 to 4.5 V to 1.25–4.5 V improved capacity retention. Batteries operating in the narrower voltage range initially exhibited a high CE exceeding 99.8%. However, this efficiency dropped significantly after just 10 cycles due to issues like oxygen evolution and phase transformation in NMC532 at high voltages.^79,141^ While both voltage windows share the same high upper cutoff voltage (4.5 V), extending the lower cutoff to 1.25 V improves capacity retention by enabling more complete lithium utilization, promoting uniform metal plating, and facilitating the formation of a stable SEI. This broader voltage range helps establish a more robust lithium inventory and interfacial stability early in the cycling process, which ultimately mitigates the degradation effects associated with high-voltage stress.

### 3.2 Cycling Performance and Energy Density

#### 3.2.1 High-rate Performance and Long-term Cycling

One of the key issues in AFBs is the efficiency of plating and stripping during fast charging. At higher charging rates, metal ions may not deposit uniformly on the CC, leading to dendrite formation. These needle-like structures can cause short circuits.^163^ Unlike in conventional metal-ion batteries, where the SEI layer forms on the anode and protects it, the SEI in AFBs forms directly on the plated metal. Since the plating and stripping processes constantly disrupt this layer, the SEI’s stability under high charging rate conditions becomes a challenge, accelerating battery degradation. Electrolyte decomposition is another concern when cycling at high rates. The faster the charging and discharging process, the more stress is placed on the electrolyte, causing it to break down and reducing the overall cycle life.^165,166^ Furthermore, temperature management plays a crucial role in high-rate performance, as rapid cycling generates heat that can destabilize the lithium plating process. The cathode materials also impact the rate performance of AFBs.^104^ High-rate cycling requires not only fast lithium-ion movement at the anode but also rapid intercalation and deintercalation at the cathode.

To address these issues, researchers are exploring new approaches to design AFBs with improved high-rate performance. For example, Ouyang et al. have developed ultralight composite CCs by engineering polyacylsemicarbazide (PASC) with copper and used them in high-energy AF-LMBs.^142^ The PASC-based Cu CC enhances the stability and flexibility of the CC, and the safety of the AFB. As a result, the PASC-based AFB achieved high cyclability. It reached a high-rate capability of 5 C (10 mA/cm^2^) as shown in Figure 16(a, b), due to the consistent Li-ion flow and smooth Li deposition on the nanostructured copper layer. Shi et al. demonstrated a high-performance anode-free zinc metal battery (AF-ZMB) by simply adding LiI to a 2 M ZnSO_4_ electrolyte dissolved in deionized water as an additive.^167^ Using this electrolyte and a zinc activated-carbon (AC) cathode, a Cu∥AC AF-ZMB exhibited excellent cycling stability and high-rate performance, as shown in Figure 16(c), maintaining 88.2% of its original capacity after 10,000 cycles at 50 mA cm^–2^. It is indicated that I^–^ regulates the early stage Zn nucleation and growth process in the underlying mechanism.

**Figure 16:**
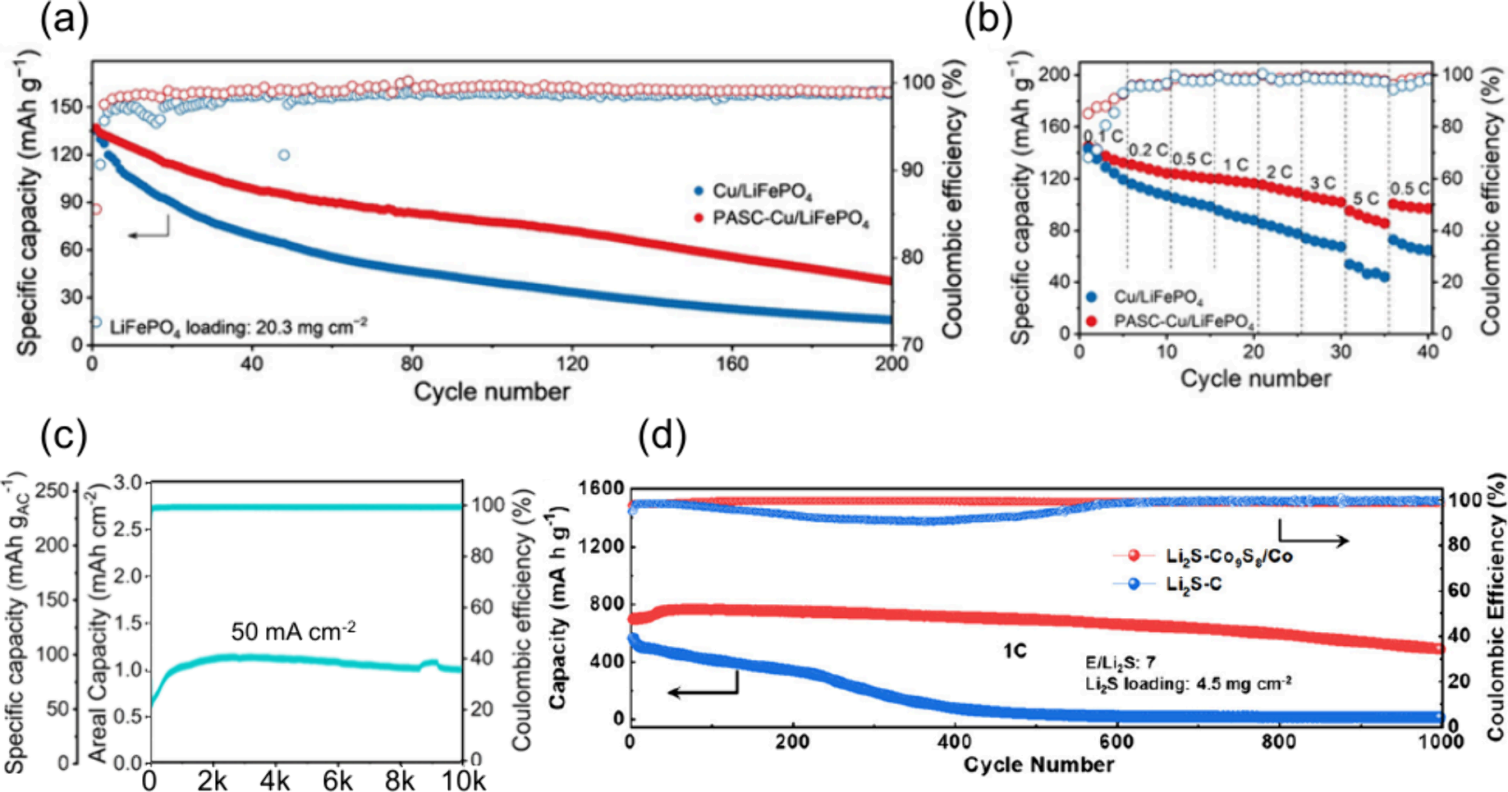
(a) Cyclability of and LiFePO_4_||Cu and LiFePO_4_||PASC-Cu AFBs at charge and discharge rates of 0.3/0.5 C, respectively. (b) Rate capability evaluation of PASC- LiFePO_4_||Cu and LiFePO_4_|| PASC-Cu AFBs under varying cycling rates varying from 0.1 to 5 C. (a, b) Reproduced with permission,^142^ Copyright 2024 Wiley-VCH GmbH. (c) cycling performance of the Cu∥AC AF-ZMB at 50 mA cm^–2^. Reproduced with permission,^167^ Copyright 2024 The Royal Society of Chemistry. (d) Cycling stability of the Li_2_S-Co_9_S_8_/Co cathode at a 1C rate. Reproduced with permission,^168^ Copyright 2022 American Chemical Society.

He et al. demonstrated that AF-LSB exhibits highly stable cycling performance at a 1C rate (1C = 1166 mA g^–1^), achieving a remarkable lifespan of over 1000 cycles.^168^ This performance was achieved under challenging conditions, including a high Li_2_S loading of 4.5 mg cm^–2^ and a low electrolyte-to-Li_2_S ratio of 7 μL mg^–1^, as illustrated in Figure 16(d). Their method involved synthesizing Li_2_S nanoparticles decorated with Co_9_S_8_ and Co embedded within a carbon matrix (Li_2_S-Co_9_S_8_/Co) using a straightforward carbothermal process. This approach enhanced Li_2_S utilization, minimized the lithium polysulfide shuttle effect, and prevented the accumulation of inactive Li_2_S on the lithium anode, thereby ensuring efficient lithium usage.^169^ Thanks to this advanced design, the AF-LSB, equipped with the Li_2_S-Co_9_S_8_/Co cathode, achieved an impressive capacity of 969 mAh g^–1^, showcasing its capability for high-performance energy storage. To enhance the high-rate performance of AFBs, it is essential to focus on strategies that promote uniform metal plating and stabilize the SEI.

To enhance the high-rate performance of AFBs across different chemistries, it is imperative to tailor strategies that reflect the unique electrochemical and physical behaviors of each metal system. In lithium-based AFBs, rapid cycling often leads to inhomogeneous lithium deposition, unstable SEI formation, and severe dendritic growth, especially under lean electrolyte conditions.^23^ Improving ion transport through optimized electrolytes, surface-engineered CCs, and robust SEI-forming additives is essential to ensure uniform lithium plating and mitigate failure.^71^ Sodium and potassium metal AFBs face similar issues. However, the larger ionic radii and higher reactivity of Na^+^ and K^+^ ions exacerbate SEI instability and dendritic tendencies during high-rate cycling.^170,171^ Their relatively low melting points also make them thermally sensitive, necessitating strict temperature regulation and electrolyte designs with enhanced thermal and electrochemical stability.^172^

In contrast, multivalent systems, such as Mg and Al AFBs, are less prone to dendrite formation but suffer from sluggish ion kinetics and severe interfacial passivation, which limits their high-rate capability.^21,173^ For these systems, high-rate performance relies on developing highly conductive electrolytes that can facilitate the transport of divalent or trivalent ions and designing interfacial chemistries that prevent the formation of blocking layers during metal deposition.^174,175^ In aqueous and hybrid Zn-based AFBs, which exhibit inherently high ionic conductivity, rate performance is often constrained by the irregular Zn plating behavior and hydrogen evolution. Strategies such as electrolyte modification, interfacial regulation through additives like halides, and optimized CC textures have shown significant promise in promoting uniform Zn nucleation and achieving stable high-rate cycling.^176,177^

In AF-ASSBs, the mechanical rigidity and limited ionic conductivity of solid electrolytes present critical barriers to rapid ion transport and interface dynamics.^178^ External pressure can mitigate contact loss, but further improvements require the design of highly conductive, mechanically robust solid electrolytes, as well as adaptive interfaces that accommodate volume changes without delamination or resistance buildup. Advanced cathode designs also play a central role in all systems; since the cathode is the only source of metal-ions in AFBs, its ability to support rapid intercalation/deintercalation and maintain structural integrity under fast cycling conditions is essential.^156,179,180^ Overall, while notable progress has been demonstrated in some studies, further advancements are needed. These include developing tailored electrolyte systems for each metal type, engineering multifunctional CCs, integrating protective interfacial layers, and refining cathode architectures that sustain rapid ion transport without compromising stability. Continued efforts in these directions will be vital to realize high-rate, high-energy-density AFBs for diverse metal chemistries.

#### 3.2.2 Energy Density

The transition from conventional metal-ion batteries to anode-free configurations represents a significant leap in the pursuit of high-energy-density energy storage systems. In a AF-LMB, removing the graphite anode, which makes up 27.8% of the total stack weight and 46.1% of its thickness, would increase gravimetric energy density (GED) by 38.5% and volumetric energy density (VED) by 85.5% at the stack level.^181–184^ To provide a holistic perspective on this evolution, we compare four key architectures: (1) metal-ion batteries (MIBs, e.g., Li-ion, Na-ion), (2) metal batteries (MBs) with excess metal foil anodes, (3) AFBs with liquid electrolytes (LEs), and (4) anode-free ASSBs (AF-ASSBs). These systems span a range of active metal chemistries, including monovalent (Li, Na, K) and multivalent (Mg, Al, Zn) platforms. Figure 17 (a) illustrates how these changes in cell configuration lead to improvements in both GED and VED.

**Figure 17:**
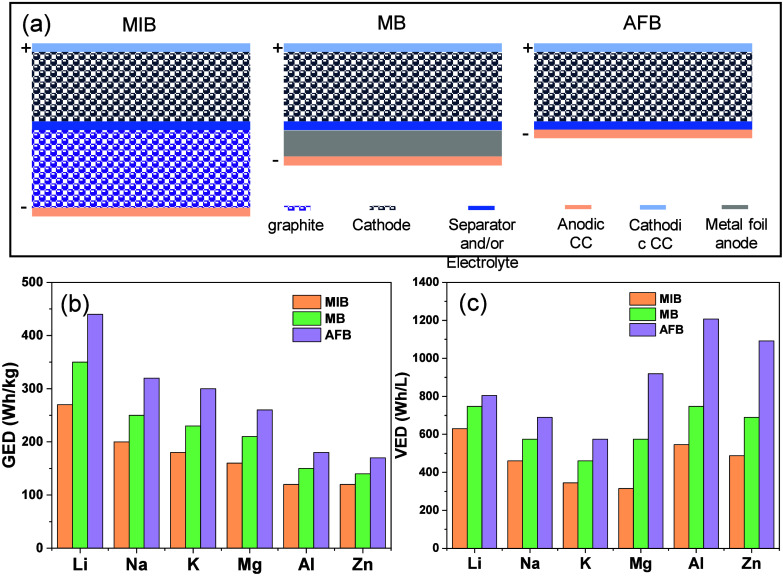
(a) Diagrammatic representation showing the configurations of various battery architectures. Comparison of energy density between the different battery architectures: (b) Gravimetric energy density (GED), (c) Volumetric energy density (VED).

The energy densities of these various architectures are compared, as presented in Figure 17(b, c). They are compiled from experimental benchmarks, theoretical projections, and modeling studies reported in the literature.^91,140,182,185–197^ For emerging systems, such as anode-free and multivalent metal batteries, where full-cell data are limited, values are estimated based on representative cathode pairings, assumed N/P ratios, and idealized stack configurations.^198–200^ These figures serve as comparative indicators rather than exact performance values.

As indicated in Figure 17(b, c), anode-free designs offer remarkable theoretical and practical energy density advantages. For instance, the gravimetric energy density of Li-based systems increases from ∼270 Wh/kg for Li-ion batteries to ∼460 Wh/kg in AF-ASSBs. In contrast, the volumetric energy density rises from ∼650 Wh/L to ∼950 Wh/L. This boost is primarily attributed to the elimination of the excess metal anode.

Na and K chemistries show similar trends, though with modest absolute energy densities due to their lower operating voltages and heavier atomic weights. For example, the gravimetric energy density of Na-based systems improves from ∼160 Wh/kg (Na-ion battery) to ∼390 Wh/kg in anode-free solid-state designs. Potassium systems, although lagging slightly due to lower redox potentials and cathode performance, still benefit significantly from architecture optimization, increasing from 120 Wh/kg to over 320 Wh/kg across the same evolution.

Interestingly, multivalent systems such as Mg, Al, and Zn exhibit exceptionally high volumetric energy densities in their anode-free forms, particularly with solid electrolytes, reaching up to 1330 Wh/L for Al-based batteries and 1210 Wh/L for Zn systems. These values exceed those of Li- and Na-based counterparts despite generally lower gravimetric metrics. This phenomenon stems from the high volumetric capacity of multivalent metals (e.g., 8046 mAh/cm^3^ for Al) and their dense electron-storage capability due to multiple redox-active electrons per atom. The trade-off, however, lies in the sluggish ion transport kinetics and complex interfacial chemistry, which remain critical challenges for practical implementation.

The MB configuration, where an excess metal foil serves as the anode, presents a compromise between energy density and operational stability. For most chemistries, this format yields moderate gains in energy density compared to ion-based cells, while maintaining relatively stable cycling performance. However, the added mass and volume of excess metal inherently cap the achievable gains, especially in terms of gravimetric efficiency. For example, Li-metal batteries achieve ∼350 Wh/kg, compared to 270 Wh/kg for Li-ion batteries, but still fall short of the 420–460 Wh/kg range observed in anode-free cells.

Moreover, anode-free designs with liquid electrolytes achieve significant density gains over foil-based systems while retaining some degree of manufacturability using current liquid electrolyte systems. These systems (e.g., Li or Na) achieve gravimetric energy densities of 420 and 345 Wh/kg, respectively, and volumetric densities of 800 and 735 Wh/L. These designs consistently yield the highest theoretical and practical energy densities across all chemistries examined.

## 4 Anode-Free Battery: A Powerful Tool for Exploring Interfacial Phenomena

Extensive work has been done to tackle the challenges of electrolyte decomposition, formation of inactive metal, and dendrite growth in metal anodes like Li, Na, and K.^201,202^ However, the irreversible processes that occur often go unnoticed or are difficult to identify, partly because of the excess metal typically used in these batteries and the simultaneous events happening at both electrodes and their interfaces. Investigating irreversible CE (irr-CE), which arises from the formation of the SEI, electrolyte breakdown, and inactive metal, is challenging in conventional metal batteries. This difficulty stems from the presence of excess metal on the negative electrode.^30,203^ To address this, a more comprehensive approach is recommended. For instance, in Li metal batteries, employing cathode||Cu, Li||Cu, and cathode||Li cell setups can be used to thoroughly evaluate and pinpoint the sources of internal charge evolution (irr-CE). This multifaceted strategy is crucial for identifying the factors that lead to irr-CE and capacity loss in both AF-LMBs and traditional LMBs. One particularly valuable configuration involves utilizing a cell without an anode in conjunction with various techniques, enabling a more precise distinction and quantification of these irreversible interfacial processes.^74,203^

Figure 18 illustrates how different setups, such as cathode||Cu, cathode||Li, Li||Cu, and Li||Li cells without an anode, offer unique insights into their chemical reactions, aiding in the understanding and diagnosis of battery failure mechanisms.^74,203^ The metal||metal symmetric such as the Li||Li cell is particularly useful for studying SEI polarization on the lithium surface, critical current density and internal short circuits. However, it is not suitable for measuring irr-CE because the presence of excess lithium at both electrodes results in a CE of 100%. In this case, the irreversible losses from SEI formation, electrolyte degradation, and inactive lithium are offset by the additional lithium. By switching the lithium working electrode with copper in a Li||Cu cell, it is easier to measure the irreversible processes occurring on the bare copper surface. This setup provides precise insights into irr-CE sources, including inactive lithium deposition, electrolyte degradation, and SEI formation, making it a valuable tool for understanding battery performance.

**Figure 18:**
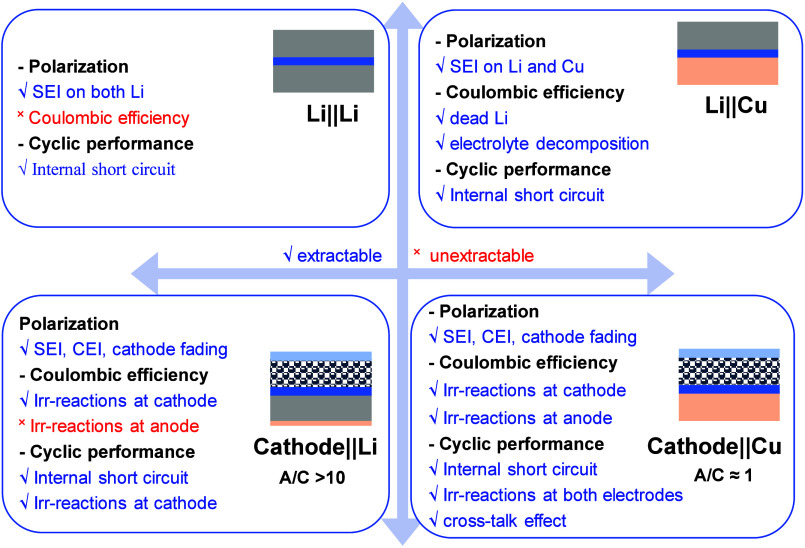
Interfacial chemistries that can be analyzed or remain inaccessible using different protocols for LMBs and AF-LMBs.

To better understand the irreversible reactions occurring at the cathode side, the cathode||Li type setup is an effective tool.^56,189^ This setup provides precise information on electrolyte oxidation breakdown at the positive electrode surface, cathode deterioration, CEI formation, and initial irreversible capacity. However, this method does not account for any information at the anode regarding the sources of the irr-CE. In an anode-free cell (Cu||cathode), an additional irr-CE source emerges due to cross-talk effects, beyond the sources of irr-CE identified in Cu||Li and cathode||Li cells. Cross-talk involves chemical or electrochemical reactions at one electrode that cause similar reactions at the other electrode. This phenomenon notably affects the behavior of plating and stripping, as well as the formation of CEI or SEI, resulting in capacity loss due to the depletion of active lithium and material. In brief, irr-CE is influenced by several factors: cross-talk effects, oxidative and reductive electrolyte breakdown, inactive lithium, and SEI formation. These mechanisms have a significant impact on the performance of AF-LMBs. Employing an integrated protocol with diverse cell setups allows for a rapid and comprehensive evaluation of critical irr-CE sources in AF-LMBs, providing valuable insights into improving their performance.

### 4.1 Visualizing Metal Plating and Dendrite Formation Dynamics

A thorough understanding of how plated metal morphology and its evolution impact battery performance is essential for advancing the practical use of AFBs. Advanced imaging techniques, such as optical microscopy (OM), transmission electron microscopy (TEM), and scanning electron microscopy (SEM), have been widely employed to observe the formation of dead metal and dendrites in real-time.^204,205^ However, these methods have limitations. For instance, SEM and TEM require high-vacuum or cryogenic environments, and the electron beams they use can damage the metal, complicating in situ observations. Additionally, performing quantitative analyses of lithium dendrites with these techniques poses significant challenges. In contrast, transmission X-ray microscopy (TXM), a nondestructive technique, offers a unique advantage.^206^ It enables high-resolution visualization of lithium’s morphological changes in an AFB setup. It has been extensively used to study microstructural changes in electrode materials during cycling. Our research team has successfully leveraged in situ TXM to observe lithium growth and stripping, using a specially designed cell that delivers high spatial resolution in standard environmental conditions. The designed cell setup offers excellent adaptability and provides a high-contrast view of the interaction between lithium and the electrolyte. This design enables detailed observation of lithium plating/stripping behaviors. At a current density of 1 mA cm^–2^, mossy lithium structures were observed to grow and shrink during the respective plating and stripping processes (Figure 19(a)). Interestingly, lithium was found to grow vertically from its base and strip faster from the top, indicating a directional preference in its behavior. Furthermore, under higher current densities across multiple cycles, mossy or dendritic lithium structures became more pronounced, suggesting that uneven current distribution leads to severe dendrite formation (Figure 19(b)). This highlights the critical role of current distribution in the morphological evolution of lithium during battery operation.

**Figure 19:**
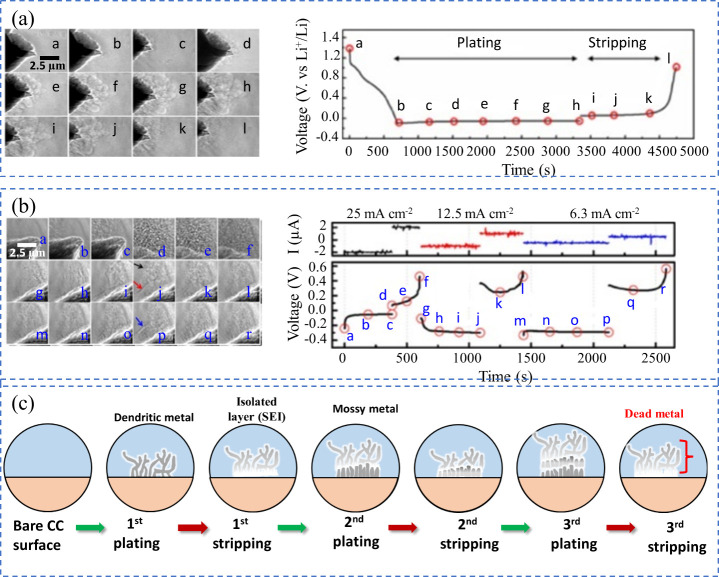
Real-time observation of lithium dendrite formation. (a) Images of in situ TXM showing lithium deposition and stripping at different stages, and the corresponding voltage profile at one mA cm^–2^ current density. (b) Images of in situ TXM illustrating lithium deposition and stripping during the 1st, 2nd, and 3rd cycles at 25, 12.5, and 6.3 mA cm^–2^ current densities, respectively, along with their corresponding voltage profiles. (a, b) Reproduced with permission,^207^ Copyright 2017 American Chemical Society. (c) Schematic representation of the mechanism leading to dead lithium formation.

The process of dead lithium formation during cycling is illustrated schematically in Figure 19(c).^207^ After the initial cycle, a dendritic lithium structure develops and becomes detached from the electrode surface. During the second cycle, as lithium plating begins, fresh lithium grows directly from the electrode, forming a denser layer that pushes the previously formed dendritic dead lithium away from the copper electrode. When lithium is stripped in the second cycle, the dendritic structure remains isolated from the electrode due to the newly formed SEI, leading to the creation of dead lithium. While the second cycle shows improved reversibility compared to the first, it still results in some irreversible SEI and dead lithium formation. This residual structure is less noticeable during the second cycle than the first, which is why TXM imaging shows lower contrast. A similar trend is observed during the third cycle.

Dendritic lithium growth and the mechanisms of short-circuiting during cycling, a Li||Cu cell was monitored using in situ OM.^74^ Initially, smooth lithium plating and stripping were observed on both the copper and lithium electrodes. However, with continued cycling, dendritic lithium began to form, eventually causing a short-circuit and the generation of dead lithium, as depicted in Figure 20(a). Interestingly, when a short circuit occurred during Li plating/stripping on the CC, the cell voltage did not drop to zero. Instead, it exhibited a slight positive (+22 mV) or negative (−22 mV) value, depending on the process. This indicates that the SEI acts as an insulating barrier, preventing direct contact between the lithium deposits on both sides. These findings highlight the complex interplay between SEI properties and dendrite formation in determining cell stability during cycling.

**Figure 20:**
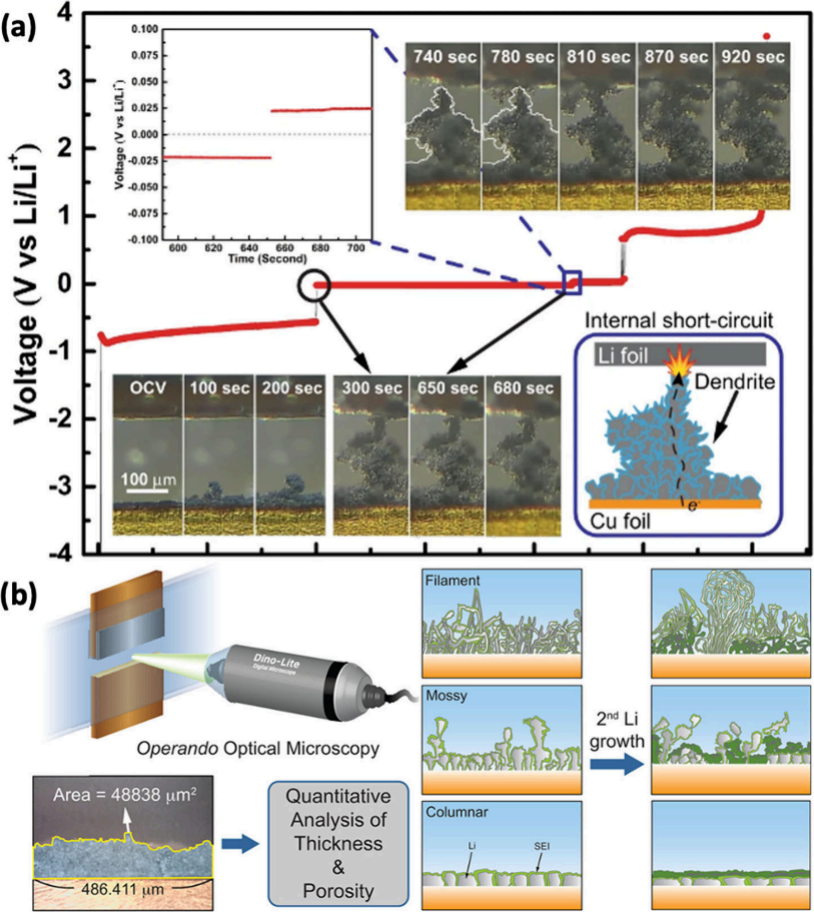
(a) Copper surface images of in situ OM of lithium deposition and stripping at 500 mA cm^–2^, an extremely high current density, resulting in an internal short circuit. Reproduced with permission,^74^ Copyright 2021 the Authors. Published by Springer Nature. (b) Schematic of operando OM measurement setup and the Li growth and capping layers based on the observations. Reproduced with permission,^208^ Copyright 2023 American Chemical Society.

Furthermore, our research group used operando optical microscopy, combined with a specially developed analytical method, to observe the behavior of Li plating and stripping in various electrolytes.^208^ This approach enabled the visualization of freshly plated lithium and the formation and evolution of surface capping layers, as schematically illustrated in Figure 20(b). This technique enabled the precise control of the current density and the accurate measurement of key properties of the lithium layer, such as its thickness and porosity, to better understand the mechanisms behind lithium growth. The observations indicated that the robustness and porosity of the remaining capping layer after lithium stripping play a crucial role in determining how dendrites grow during subsequent cycles. Specifically, fragile capping layers tend to fracture easily, allowing dendrites to form rapidly. In contrast, a dense and mechanically stable capping layer can support smooth and uniform lithium plating and stripping, even when operating at high current densities. These insights help explain the distinct capping and stacking behaviors that influence lithium growth over repeated cycling. These findings strengthen the fundamental understanding of metal plating and stripping during consecutive cycles and the influence of dead metal formation in AFBs.

### 4.2 Quantifying Dead Metal

Quantifying the amount of dead metal after cycling is crucial for diagnosing failure in metal batteries. Titration gas chromatography (TGC) gives an absolute measure of dead Li. It consistently shows that capacity fade in Cu||Li and broader LMBs is driven mainly by dead-Li formation rather than SEI growth.^209^ Still, TGC alone cannot capture all sources of irr-CE loss in AF-LMBs. To follow interphase dynamics, liquid secondary ion mass spectrometry (liquid-SIMS) has been used to track SEI formation on Cu in real time (e.g., in Cu||LiCoO_2_ cells).^210^ Complementing this, in situ NMR tailored for Cu||LiFePO_4_ anode-free cells directly quantifies metallic-Li loss.^211,212^ The spectrum resolves pristine Li at ∼245 ppm and newly deposited Li near ∼263 ppm (Figure 21(a, b)).^212^ Combining in situ and ex situ NMR separates inactive Li^0^ from SEI-bound Li^+^. It shows that inactive lithium can accumulate on both the Cu and Li sides of the cell. Moreover, in situ NMR spectroscopy was also used to study the effects of FEC as an electrolyte additive. The results indicated that the fraction of dead lithium decreased greatly when 5% FEC was added to 1 M LiPF_6_ in EC/DEC (3:7 wt/wt), dropping to 3.3%, compared to 9.4% without the FEC additive. This highlights the importance of electrolyte additives in reducing lithium loss and improving battery efficiency.

**Figure 21:**
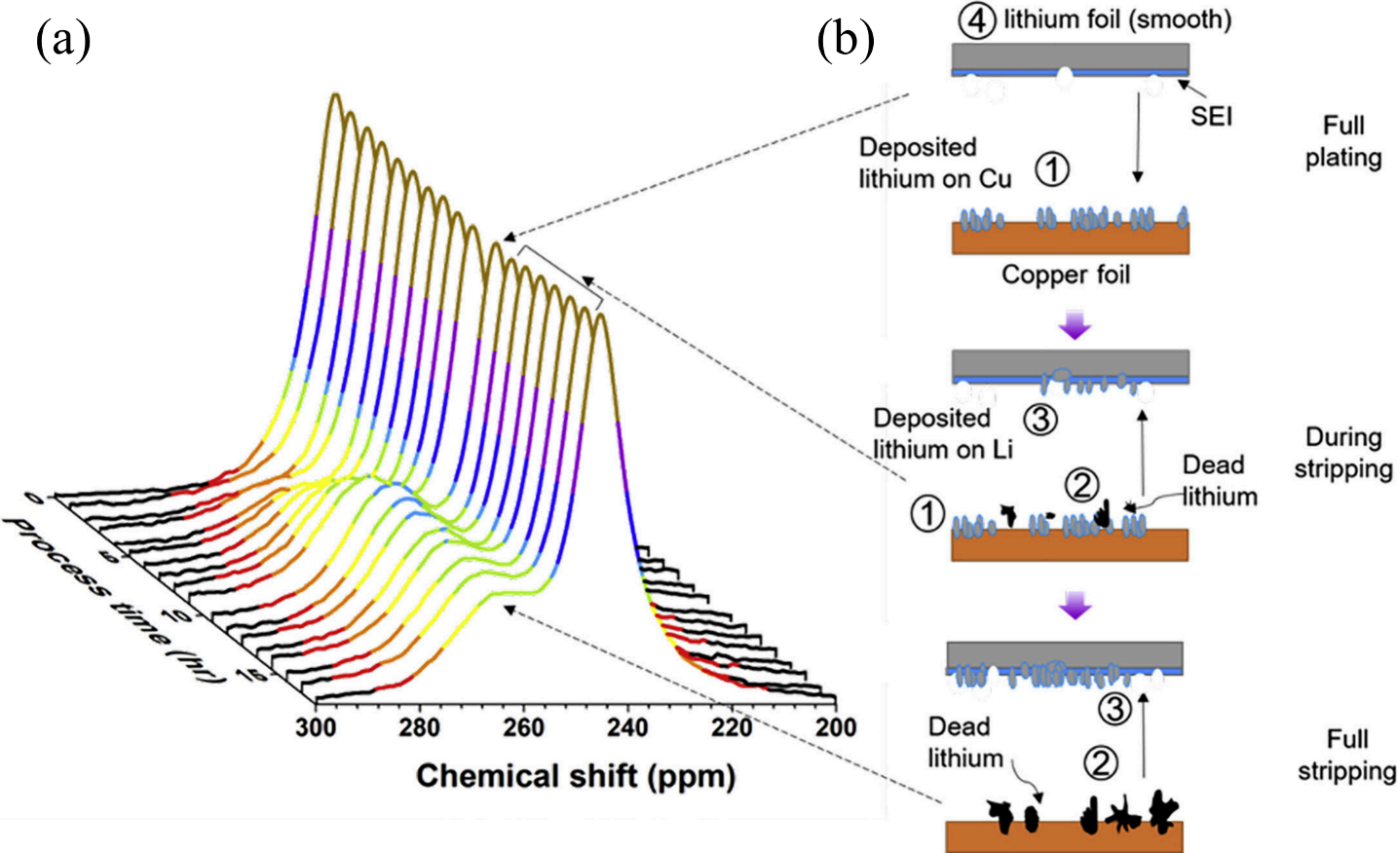
Analysis of dead lithium and irr-CE using an AFB approach. (a) Spectra of in situ ^7^Li NMR showing Li deposition and stripping throughout the cycling process. (b) Diagram depicting the stages of Li plating and the presence of dead Li. (a, b) Reproduced with permission,^212^ Copyright 2020 The Author(s) published by Cell Press.

Furthermore, using an AFB as a methodological tool, ex situ NMR obtained after a complete stripping step further enables the differentiation between dead metal (such as dead Li) and SEI formation, helping in separating their contributions to irr-CE. Because the anode-free architecture contains no reservoir of Li, any Li detected after stripping is, by definition, irreversibly lost as either inactive metallic Li^0^ or SEI-bound Li^+^. As illustrated in Figure 22, spectral deconvolution separates the Knight-shifted Li^0^ resonance (∼260 ppm) and the SEI-Li^+^ envelope (0–10 ppm).^213^ Using the plated charge *Q*_plated_ and Faraday’s constant *F*, the moles of Li deposited are

$$n_{\mathrm{plated}}=Q_{\mathrm{plated}}/E$$

**Figure 22:**
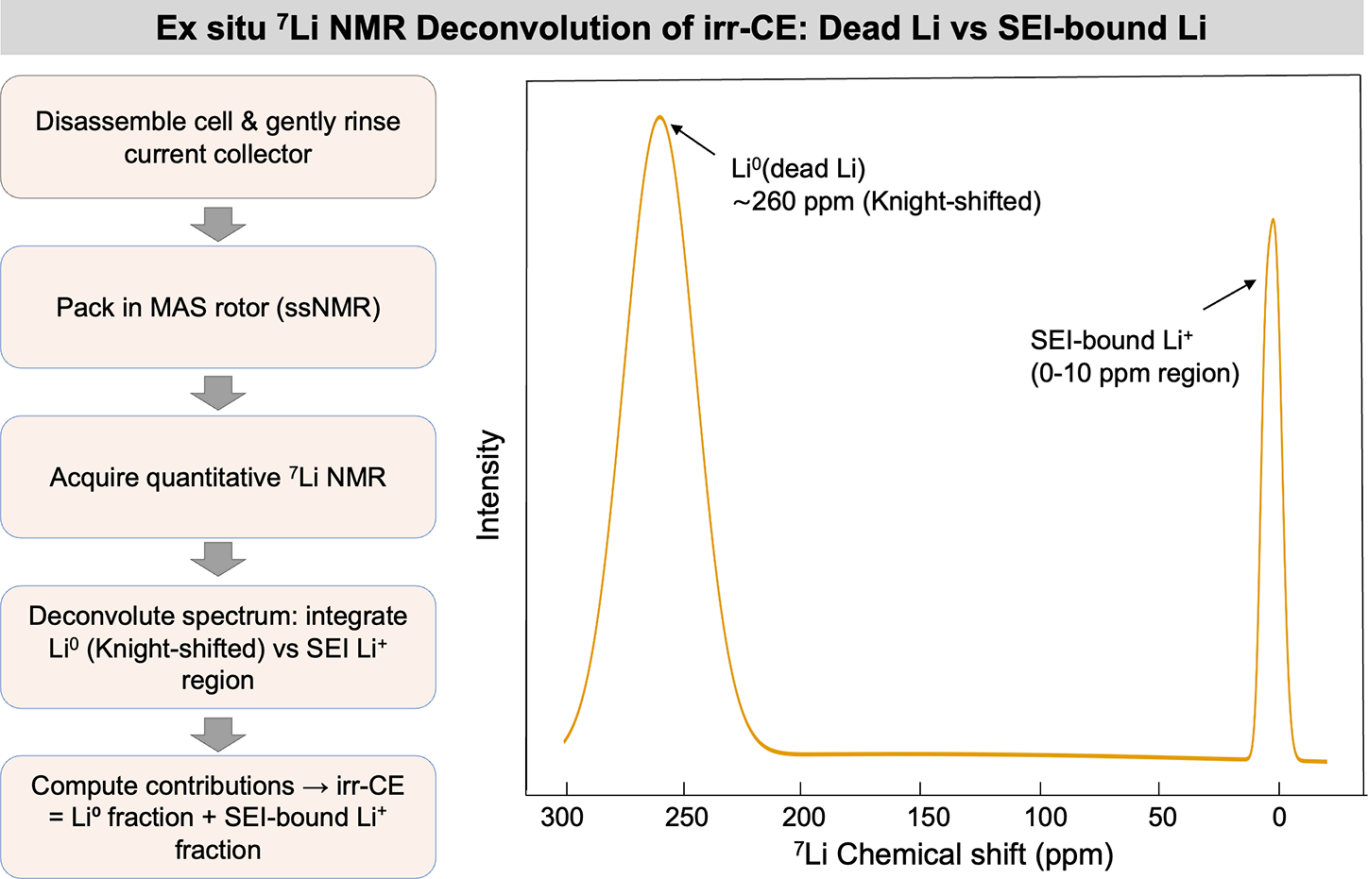
Ex situ ^7^Li NMR schematic used to separate the contributions to irr-CE: Li^0^ (dead Li, ∼260 ppm) vs SEI-bound Li^+^ (0–10 ppm). Actual positions and widths depend on reference and experimental conditions.

The fractional (*f*) losses are then:

$$\begin{array}{l} f_{\mathrm{dead}}={n\mathrm{Li}}^{0}/n_{\mathrm{plated}}\;\mathrm{and}\\ f_{\mathrm{SEI}}=\left({{n_{\mathrm{SEI‐bound Li}}}^{+}}_{\mathrm{after stripping}}-{{n_{\mathrm{SEI‐bound Li}}}^{+}}_{\mathrm{before cycling}}\right)/n_{\mathrm{plated}}\\ \mathrm{giving}:\mathrm{irr‐CE}\approx f_{\mathrm{dead}}+f_{\mathrm{SEI}} \end{array}$$

giving: irr-CE ≈ *f*_dead_ + *f*_SEI_.

### 4.3 Determining Irreversible Coulombic Efficiency

AFBs are ideal for quantifying irr-CE because they start with zero active metal on the anode. During the first charge, the metal plates from the cathode onto the CC, creating a clear baseline: any loss per cycle results from dendrite formation, side reactions, or SEI growth. Advanced imaging and post-mortem methods can directly estimate “dead metal,” enhancing the material balance. In practice, irr-CE for the first cycle is calculated from the ratio of metal recovered after stripping to the metal plated during the first charge; with no pre-existing metal, inefficiencies directly reflect cycling processes.^214^ Our research group used an integrated protocol with Li||Cu, cathode||Li, and cathode||Cu (anode-free) configurations to identify the sources of irr-CE in AF-LMBs and conventional LMBs.^74^ First-cycle irr-CE in AF-LMBs primarily stems from three factors: (i) SEI formation on Cu, (ii) dead Li trapped beneath or within the SEI, and (iii) cross-talk when the anode-to-cathode capacity ratio drops below one. The Cu||Li cell isolates (i) and (ii): it measures the charge lost to SEI and the metallic Li that becomes electrically disconnected. Subtracting this Cu||Li loss from the total irr-CE observed in the AF-LMB (Cu||cathode) isolates the cross-talk component (purple bar in Figure 23(a)).^74^ The cathode||Li cell shows the cathode’s inherent irreversible capacity on the first cycle, due to electrolyte oxidation at high voltage and degradation of the cathode. These losses are important in anode-free operation because they provide an additional Li source to the anode early on, allowing the initial anode-to-cathode capacity ratio to exceed one. As cycling continues and Li is consumed by SEI growth and dead-Li formation, this ratio decreases and eventually falls below one, indicating a shift to Li-starved conditions. Together (Figure 23(a, b)), the Li||Cu and cathode||Li baselines help break down the Cu||cathode anode-free data into segments for SEI, dead metal, and cross-talk, offering a clear and quantitative view of where charge is lost and how to target it.

**Figure 23:**
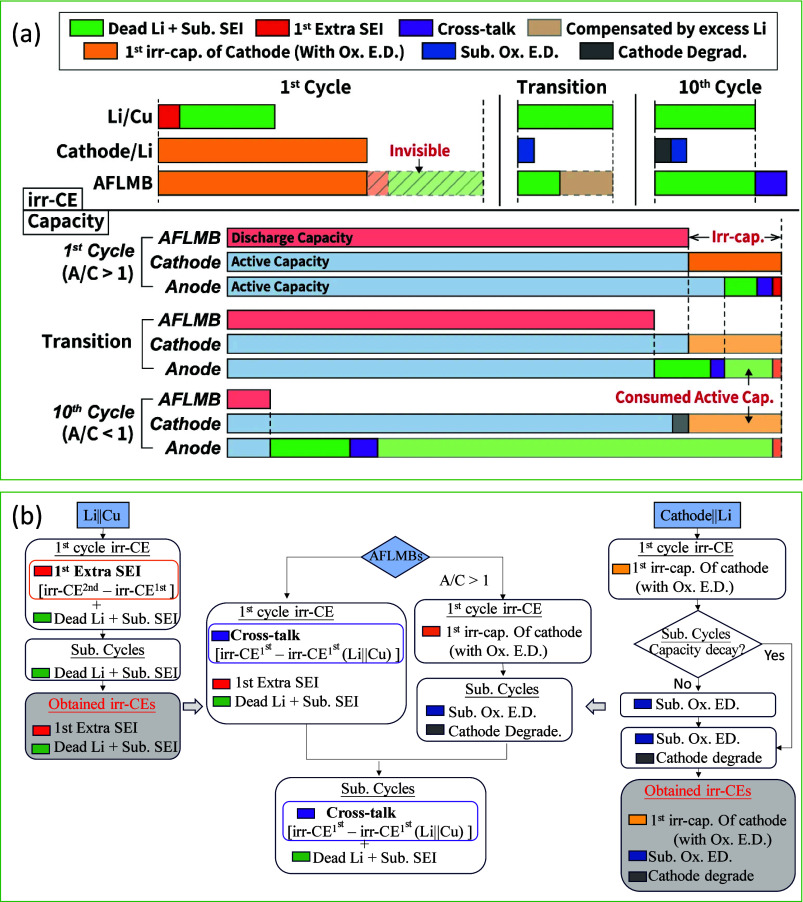
(a) Illustration of proposed integrated method combining Cu||Li and Li||cathode cells to identify the irr-CE source in AF-LMBs. (b) A diagram of the protocols to determine the source IR-CE in various cell setups. (a, b) Reproduced with permission,^74^ Copyright 2021 The authors published by Springer Nature.

### 4.4 Screening Electrolytes Using Anode-Free Configurations

AFBs provide a unique platform for screening electrolytes, since the absence of an excess metal reservoir makes cell performance highly sensitive to electrolyte composition and interfacial chemistry. By directly revealing how solvent choice, salt combinations, and additive packages influence plating/stripping reversibility, the anode-free configuration enables rapid evaluation of candidate systems.^215,216^ This approach has been widely applied to compare carbonate, ether, fluorinated, and dual-salt formulations, providing mechanistic insight into their ability to stabilize the interface and extend cycle life. For instance, Weber et al. developed several dual-salt carbonate-based electrolyte formulations. They used them in AFBs to identify and optimize those that provide a long cycle life.^141^ Among all the electrolytes tested, the dual-salt combination of LiDFOB and LiBF_4_ achieved the best result, with 80% retention of the initial capacity after 90 cycles, as shown in Figure 24(a). This electrolyte was tested in a cell pairing a LiNi_0.5_Mn_0.3_Co_0.2_O_2_ (NMC532) cathode electrode with copper CC. The excellent performance of these AF-LMBs in the dual-salt electrolyte was attributed to the formation of a robust SEI with an optimal balance of inorganic and organic components. Rodriguez et al. explored various ether-based electrolyte formulations in LFP||Cu AFB, as depicted in Figure 24(b).^217^ By utilizing anode-free cells, they could directly measure lithium loss in cells with a limited lithium supply. Among the tested electrolytes, the 4 M LiFSI in DME achieved the best performance in retaining the initial capacity. However, the morphology of the lithium electrodeposited from this electrolyte was similar to that observed with other electrolytes that showed poor capacity retention. Our research group also evaluated several electrolyte formulations by examining key performance indicators such as average CE and cycle life with 50% capacity retention.^216,218,219^ As shown in Figure 24(c), these metrics were assessed using a LiNi_0.3_Mn_0.3_Co_0.3_O_2_ (NMC111) cathode and a copper CC. Among the electrolytes tested were locally concentrated formulations, various blends with dual additives (KPF_6_ and TMSP), and KNO_3_, among others. The findings revealed that the electrolyte prepared from 1 M LiPF_6_ in a 3:7 v/v mixture of FEC/TTE was the most effective in terms of performance, while the commercially available electrolyte (EC/DEC in a 1:1 v/v ratio) demonstrated the poorest results.

**Figure 24:**
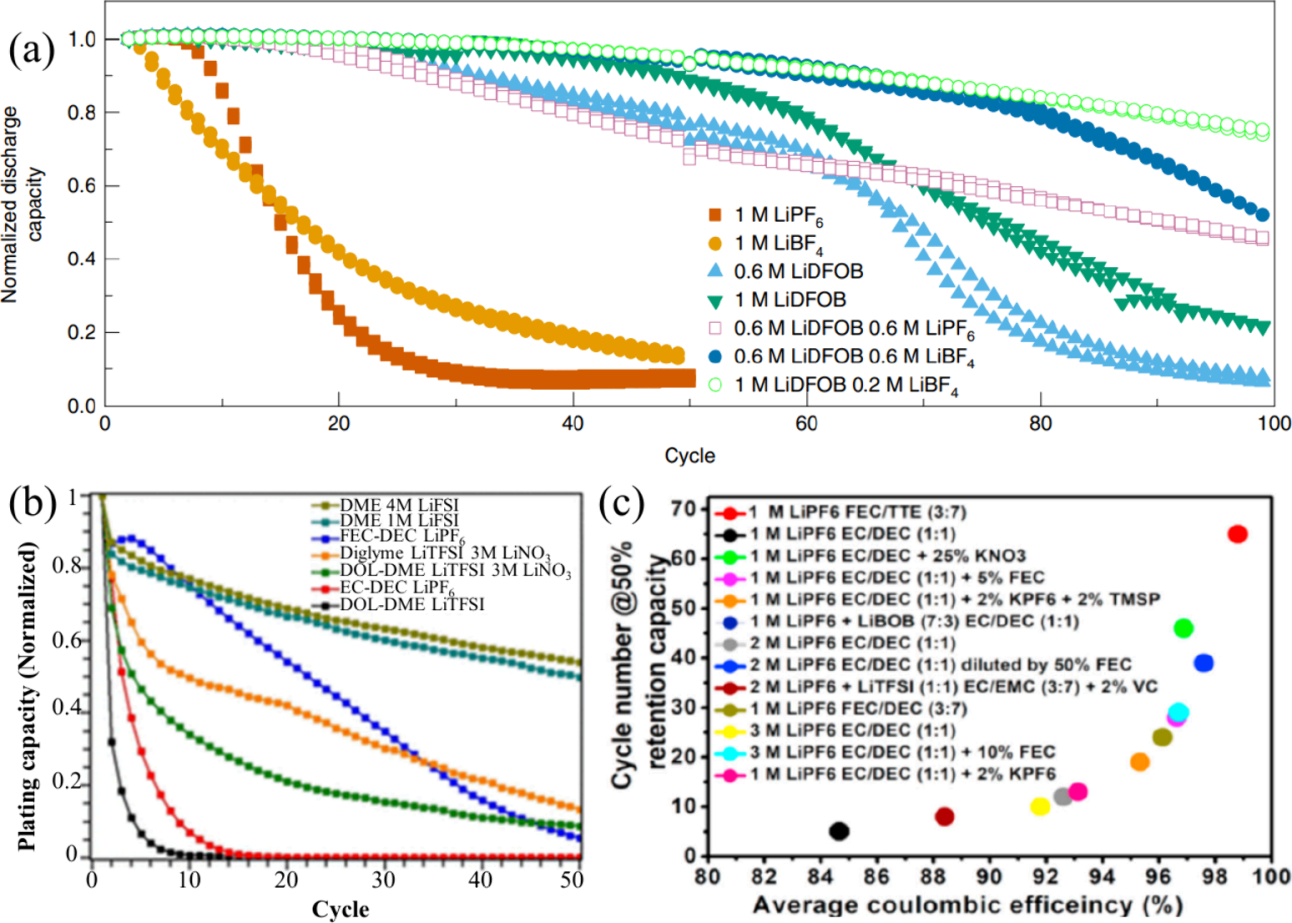
Screening of electrolytes in AFB system. (a) Comparison of capacity retention over multiple cycles for Cu||NMC532 pouch AFB with various Li salt-based electrolytes. Reproduced with permission,^141^ Copyright 2019 Nature Springer. (b) Retention of the Coulombic capacity of LFP|Cu cells for different electrolytic solutions normalized to the first cycle. Reproduced with permission,^217^ Copyright 2018 American Chemical Society. (c) Average CE at 50% capacity retention for Cu||NMC532 AFBs. Reproduced with permission,^216^ Copyright 2020 Elsevier.

## 5 Challenges in Anode-Free Batteries

AFBs present significant challenges stemming from their dependence on the cathode as the only source of active ions, the fragile nature of metal plating and stripping processes, and the sensitive interaction between electrode interfaces and electrolytes. These main challenges can be grouped into six interconnected categories as presented in Figure 25.

**Figure 25:**
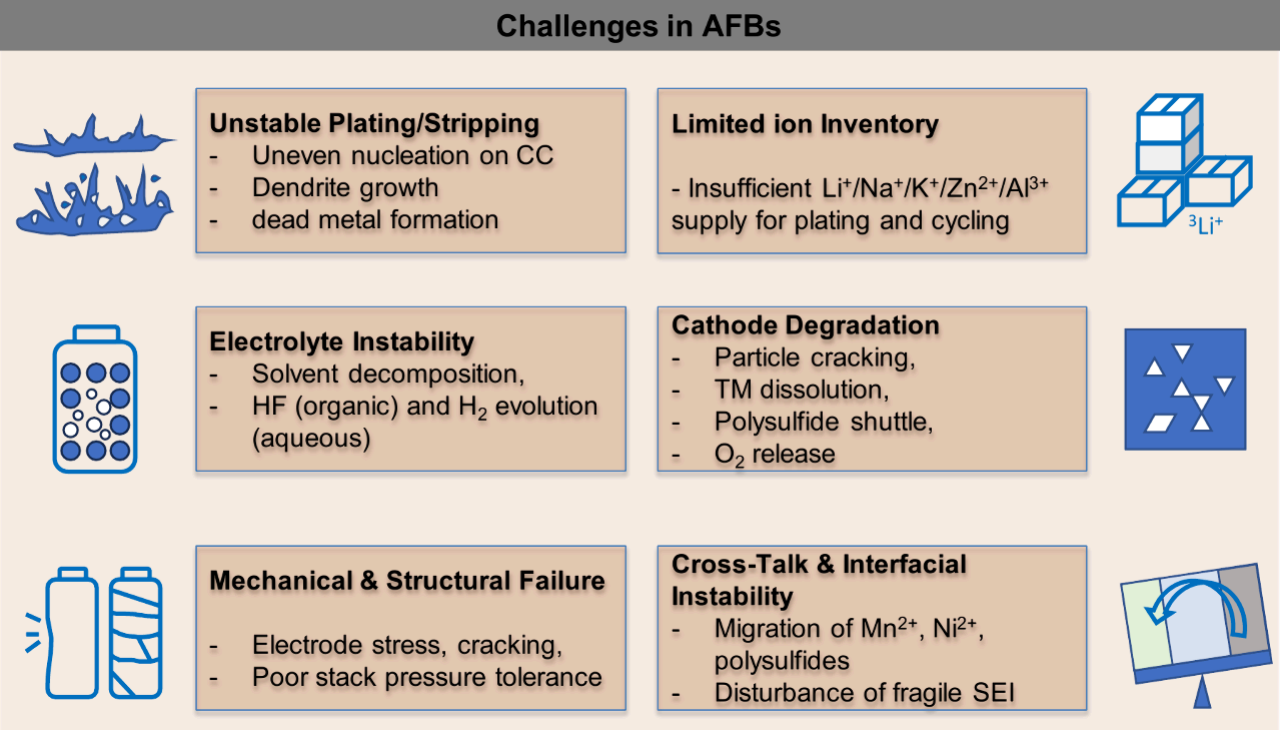
Key challenges in AFBs, including ion inventory loss, unstable plating/stripping, cathode degradation, electrolyte breakdown, cross-talk, and mechanical failure. These coupled issues collectively limit CE, cycling stability, and energy density.

### 5.1 Limited Metal Inventory

Since no active metal is supplied at the anode, all ions needed for reversible plating must originate from the cathode. A significant portion of these ions is irreversibly consumed during the initial cycles to form interphases such as the SEI on the anode. Interphase formation at both electrodes traps a portion of the limited amount, thereby decreasing the cyclable quantity available for future cycles.^36,75,137,220^ In ion-starved AFBs, this irreversible consumption cannot be compensated for, unlike in traditional systems where excess metallic anode material serves as a buffer. Mechanistically, Sand’s time analysis highlights that ion depletion at the interface accelerates with a smaller initial inventory, resulting in early instability and a limited cycle life.^23,221,222^

### 5.2 Unstable Plating and Stripping

The CCs in AFBs are not naturally metallophilic or ionophilic, which results in high nucleation overpotentials and uneven current distribution during initial plating. When the nucleation barrier is high, only a few nuclei form, causing the overall current to pass through a limited number of nucleation sites.^41,223,224^ Ions are consumed faster at these sites than the electrolyte can replenish them. In other words, diffusion constrains the process and dominates the overpotential (Ω_D_ ≫ Ω_ct_), as demonstrated in Figure 5(a). This local ion deficiency creates strong concentration gradients near the tips, so those raised spots attract more current and grow faster. The deposit does not form a uniform film; instead, it extends outward at the tips, resulting in nodular, porous, or dendritic shapes.^39,225^ Once the surface becomes rough due to diffusion control, the uneven deposit is stripped unevenly; the top of the tips loses electrical contact, and dead metal accumulates, as clearly shown in Figure 19(c). Over multiple cycles, diffusion-controlled plating and uneven stripping increase roughness, elevate local stress, and raise the risk of soft shorting and early failure.^150,226^

### 5.3 Cathode Degradation

As the only source of active ions, the cathode experiences more intense extraction and harsher redox conditions than in conventional cells.^29,227,228^ Layered oxides face issues such as transition-metal dissolution, oxygen release, and structural collapse under such extreme conditions. Sulfur cathodes in anode-free sulfur batteries (AF-SBs) suffer from polysulfide dissolution and shuttle effects, which destabilize the SEI on the anode side.^168,229,230^ Oxygen cathodes produce highly reactive intermediates that can migrate easily and cause parasitic reactions. Conversion-type transition metal compounds undergo significant volume changes and exhibit slow kinetics, resulting in the formation of soluble or inactive byproducts.^231,232^ Across different chemistries, these degradation pathways decrease ion availability, leading to SEI fracture at the anode interface.^233,234^

### 5.4 Electrolyte Instability

The electrolyte in AFBs must simultaneously stabilize both the cathode and the plating interface, a requirement rarely met under real-world operating conditions.^38,166,235^ Such degradation accelerates the accumulation of electronically isolated deposits, directly increasing the fraction of dead metal as illustrated in Figure 19(c). Once solvent breakdown products integrate into the SEI/CEI, ion transport is hindered and instability propagates across cycles, further amplifying capacity fade.^236,237^ In organic systems, solvent cointercalation, decomposition into gaseous or radical species, and HF formation lead to irreversible losses. In aqueous systems, hydrogen and oxygen evolution predominate, consuming active ions and altering the pH.^238^ In multivalent systems, strong solvation and slow desolvation processes hinder plating, resulting in high polarization and poor reversibility.^239,240^ Therefore, electrolyte instability results not only from parasitic ion consumption but also from structural and interfacial breakdown across both electrodes.

### 5.5 Cross-Talk and Interfacial Instability

Cross-talk is one of the most insidious failure modes of AFBs, caused by the migration of degradation products from the cathode to the anode side. Dissolved transition-metal ions catalyze side reactions or deposit parasitically at the anode.^31,221^ Polysulfides migrate across the separator, disrupt SEI uniformity, and cause dendritic growth.^241,242^ Electrolyte decomposition products such as HF, CO_2_, or reactive oxygen species diffuse freely, destabilizing both electrodes.^243,244^ In aqueous systems, anion crossover leads to passivation and corrosion.^245,246^ Cross-talk thus directly links cathode degradation to plating failure, amplifying damage through a feedback loop that shortens cycle life.

### 5.6 Mechanical and Structural Failure

AFBs are also susceptible to mechanical instabilities caused by repeated plating and stripping, as well as extensive cathode use.^23,227,247^ Conversion-type cathodes undergo significant volume expansion and contraction, which generates internal stress, cracks, and new reactive surfaces. These defects promote dissolution and side reactions.^248,249^ At the cell level, insufficient stack pressure can create interfacial voids, while excessive pressure can cause particle fracture.^250,251^ Stress-electrochemistry coupling further aggravates side reactions by forming reactive defect sites and uneven current pathways. Over time, these mechanical instabilities significantly contribute to irreversible ion loss and interfacial failure.

The challenges in AFBs are highly interconnected. A limited ion supply exacerbates unstable plating, while cathode degradation generates soluble intermediates that cause cross-talk and destabilize the SEI. Electrolyte instability accelerates each of these processes, and mechanical stress worsens them by exposing new reactive surfaces. Therefore, these challenges cannot be solved separately; effective solutions must address multiple degradation pathways simultaneously. Identifying and understanding these linked mechanisms is the first step toward designing stable, high-performance AFB systems.

## 6 Strategies to Overcome Challenges in Anode-Free Batteries

The unique challenges of AFBs, including ion deficiency, unstable metal plating, cathode degradation, and interfacial instability, necessitate the development of integrated solutions. To address these, strategies are generally organized into four main areas: electrolyte engineering, which modifies solvation and helps build robust interphases; CC and interface design, aimed at guiding uniform nucleation and reducing mechanical stress; cathode optimization and prelithiation, to ensure sustainable ion reservoirs; and separator modification, to control ion transport and block harmful species. Collectively, these strategies establish a framework for overcoming the inherent limitations of AFBs and enabling their practical use.

### 6.1 Liquid Electrolyte Engineering

Electrolytes serve as the chemical and physical medium through which ions traverse between electrodes. In AFBs, the electrolyte assumes an even greater responsibility than in conventional battery chemistries. The electrolyte must not only provide high ionic conductivity and stability but also guide the nucleation, plating, and stripping of reactive metals (Li, Na, K, Zn, Mg, Al) with minimal losses.^14,252–255^ In contrast to conventional cells, where the excess anode can buffer interfacial instability, the CEs in AFBs directly translate to irreversible capacity loss and shortened lifetime. Thus, electrolyte engineering is the most decisive factor in enabling long-lived, high-efficiency AFBs.^254,256,257^

The field of liquid electrolyte design for AFBs encompasses two broad domains: nonaqueous organic electrolytes (carbonates, ethers, nitriles, fluorinated solvents, ionic liquids) that dominate Li, Na, and K chemistries, and aqueous electrolytes (Zn, Al, Mg, Ca salts in water-rich or hybrid solvents) where safety, cost, and abundance are central drivers.^255,258–260^ Although these systems differ in solvent polarity, solvation energetics, and redox stability, they share key principles for performance enhancement, particularly in terms of concentration, coordination environment, and controlled interfacial decomposition, which serve as common levers across various chemistries.

#### 6.1.1 Functional Solvents and Cosolvents

In liquid electrolytes, the solvent simultaneously controls bulk ion transport and provides the molecular precursors to interfacial films; therefore, its donor number, dielectric constant, viscosity, and frontier orbital energies (HOMO/LUMO) collectively determine both solvation thermodynamics and resistance to reductive or oxidative process decomposition.^261–264^ Fluorinated ethers and carbonates lower solvent HOMO energies and favor reduction toward inorganic fluoride formation.^265–267^ Nitriles (e.g., acetonitrile, adiponitrile) offer high permittivity and excellent oxidative stability;^268,269^ but many nitriles are flammable and toxic, requiring additive-assisted protection at highly reducing anodes.^270,271^ Sulfone and sulfoxide solvents (e.g., tetramethylene sulfone, ethyl methyl sulfone) offer wide liquidus ranges and stability above 5 V;^272^ however, their high viscosity impedes ion mobility.^273^ Effective functionalized solvents are essential for stable, high-performance AF-LMBs. Figure 26 illustrates how functional solvents fundamentally alter the composition and stability of the SEI in AFBs. Such solvents decompose preferentially to form inorganic-rich SEI layers, which provide mechanical robustness, chemical stability, and good ionic conductivity. Such inorganic-dominant SEIs suppress dendrite nucleation and growth, reduce interfacial overpotential, and expand the electrochemical stability window, thereby improving cycling reversibility.^274,275^ In contrast, nonfunctional solvents tend to produce organic-rich SEIs, which are mechanically weaker and more permeable to electrolyte decomposition products. This leads to unstable interfaces, higher overpotential, and accelerated dendritic deposition.^274,276^ Thus, functional solvent design offers a direct and powerful route to stabilize metal plating/stripping in anode-free configurations. Several studies, as summarized in Table 2, that employed this approach are reported to have improved the performance of AFBs.

**Figure 26:**
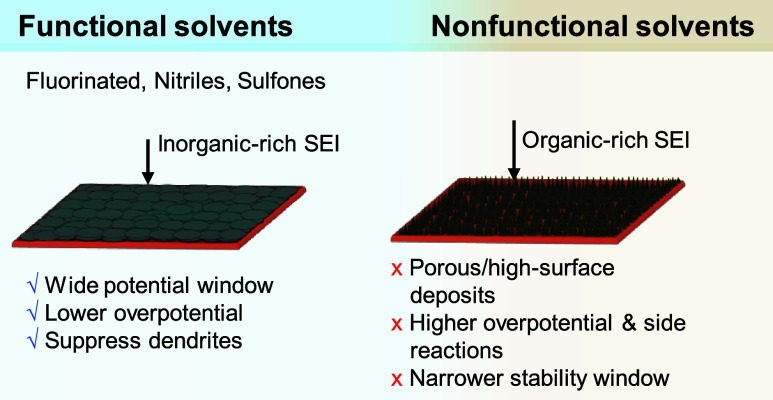
Functional solvents (such as fluorinated, nitriles, sulfones) yield stable inorganic-rich SEI, suppressing dendrites and lowering overpotential, unlike nonfunctional solvents that form unstable organic-rich SEI.

**Table 2:** Performance of AFBs Enabled by Functional Solvent and/or Cosolvent Designs

Battery type	Cell configuration	Electrolyte	Potential window (V)	Capacity (mAh cm^–2^)	Current	Cycle	CR (%)	CE (%)	Ref.
AF-LMB	Cu||NMC532	1 M LiFSI in FDMB	2.7–4.3	2.7	0.3C	100	80	-	^290^
	Cu||NMC111	1 M LiPF_6_ in FEC/TTE/EMC (3:5:2 v/v)	2.5–4.5	2.0	0.2/0.5 mA cm^–2^	80	40	98.30	^307^
	Cu||NMC811	1 M LiPF_6_ in FEC/FEMC/1,1,2,2- tetrafluoroethyl 2,2,3,3-tetrafluoropropyl ether (HFE) (2:6:2 wt %)	2.8–4.4	2.0	0.5 mA cm^–2^	50	30	-	^308^
	Cu||NMC811	LiFSI-1.2DME-3TTE (in molar ratio)	2.8–4.4	4.2	0.1/0.33C	70	77	-	^254^
	Cu||NMC811	1 M LiBF_4_ + 1 M LiDFOB in tFEP/FEC (tFEP: ethyl 3,3,3-trifluoropropanoate)	3–4.6	4.64	0.5C	100	80	98.7	^294^
	Cu||LiNi_0.8_Co_0.15_Al_0.05_O_2_	1 mL 1 M LiPF_6_ in EC/DEC, 0.07 g LiNO_3_, and 0.6 mL G2 G2: Diglyme	3–4.2	2.0	0.2C	50	73	98.6	^309^
AF-SMB	Al-C||P2-NaNMT	1 M NaPF_6_ in G2/G4 = 9:1, v/v%	2–4.1	1.0	0.1C	80	97.0	-	^166^
	Al/C||Na(Ni_0.3_Fe_0.4_Mn_0.3_)O_2_	1.2 M NaPF_6_ + 0.6 mM NaBF_4_ in Diethylene glycol dibutyl ether	2–4	-	0.2C	100	83.1	-	^291^
	Cu||Na[Ni_1/3_Fe_1/3_Mn_1/3_]O_2_	2 M NaPF_6_ in DEE	2–4	1.0	0.5 C	100	82.3	-	^295^
AF-ZMB	Cu||LiFePO_4_/C nanoplates	1.5 M Zn(CF_3_SO_3_)_2_ and 1.5 M LiCF_3_SO_3_ in H_2_O/EG (1:1 v/v) EG = ethylene glycol	0.6∼1.6	0.35	1 mA cm^–2^	100	75.2	99.7	^310^
	Cu||ZnMn_2_O_4_	1 M Zn(OTf)_2_) in H_2_O/PC (1:1 v/v) PC: propylene carbonate	0.8–2.1	1.0	0.5C	275	80	99.66	^239^
	Cu||zincificated NaV_3_O_8_·1·5H_2_O (ZnNVO)	3 M Zn(OTf)_2_ in H_2_O/IDE (6:4 v/v) IDE: isosorbide dimethyl ether	0.2–1.6	3.0	0.5C	300	60	-	^311^

Cosolvent strategies intentionally combine molecules with complementary donor strengths, permittivities, and viscosities to separate bulk transport from interphase formation.^277^ The change in solvation structure is summarized in Figure 27: a solvent-rich regime dominated by solvent-separated ion pairs (SSIPs) tends to form an organic-rich SEI, with a low transference number (*t* ^+^ ≈ 0.2–0.3) and a lower desolvation barrier (Δ*G*_desolv_), which together promote dendritic growth.^278^ In contrast, an anion-participating regime with more contact-ion pairs (CIPs) and aggregates (AGG) favors an anion-derived SEI, a higher *t* ^+^ (→ 0.5), higher Δ*G*_desolv_, and results in smoother, denser metal deposition.^279,280^ This approach adjusts solvation thermodynamics and desolvation kinetics in organic electrolytes. Since no single solvent can optimize conductivity, interphase chemistry, and stability simultaneously, cosolvent mixtures are often used to separate these functions. Typical design logic includes: (i) carbonate/ether blends, where carbonates provide oxidative stability while ethers reduce viscosity and enhance ion transport;^281,282^ (ii) fluorinated/nonfluorinated blends that combine chemically inert, weakly or nonsolvating highly fluorinated electrolytes with coordinating ethers to create LHCEs;^283–285^ and (iii) mixed-dielectric systems that combine high-permittivity components (nitriles, carbonates) with low-permittivity ethers, tuning solvation free energy and ion pairing.^270,286,287^ Such blending reshapes cation coordination-number distributions and desolvation barriers, thereby altering nucleation kinetics.

**Figure 27:**
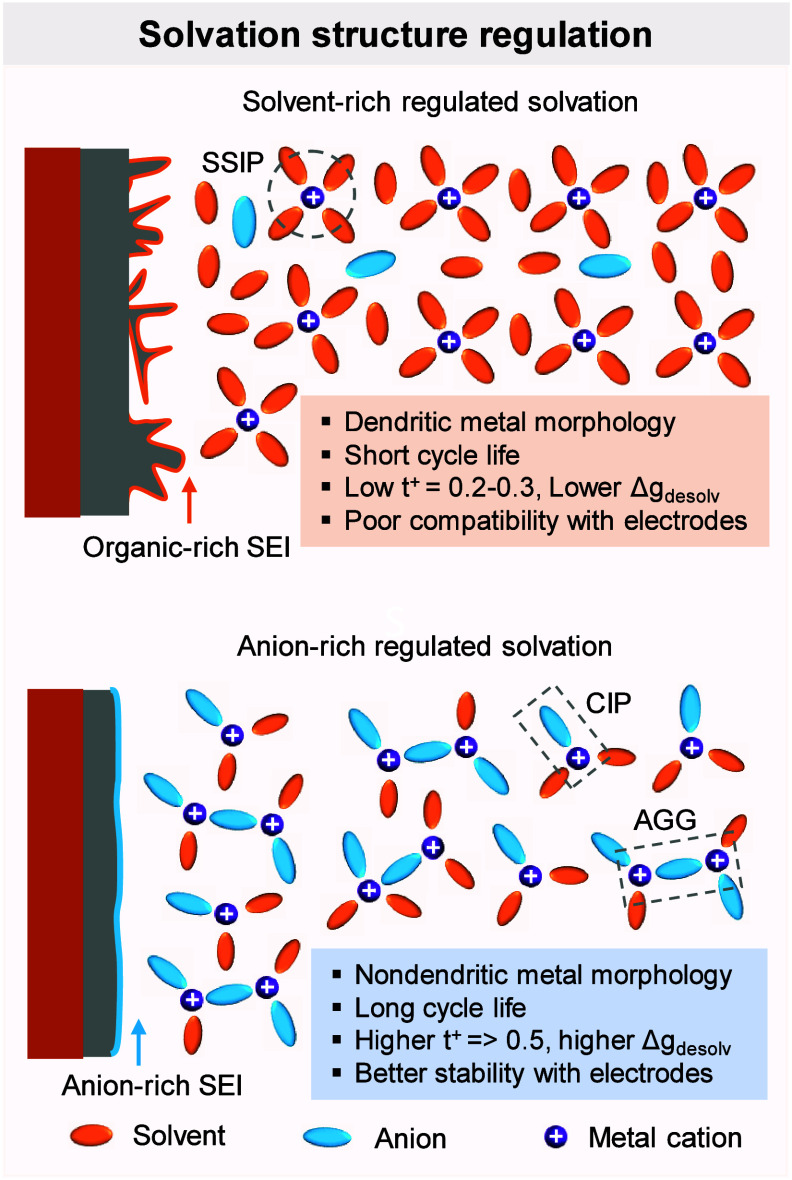
Solvation structure regulation by solvents. Top: solvent-rich solvation dominated by solvent-separated ion pairs. Bottom: solvents that promote anion coordination shift the ensemble toward contact ion pairs or aggregates, enabling anion-first reduction.

For example, in LiFSI + TTE/DME formulations, molecular dynamics studies indicate that Li^+^ is often cocoordinated by FSI- and TTE, resulting in LiF-rich SEIs and suppressed dendrite growth.^77,288,289^ Effective functionalized solvents are essential for stable, high-performance AF-LMBs. For instance, adding -CF_2_- units into DME creates the fluorinated solvent fluorinated 1,4-dimethoxylbutane (FDMB) (Figure 28(a)).^290^ A 1 M LiFSI in FDMB electrolyte exhibits high anion content and strong Li-F interactions, facilitating dense Li deposition, broader oxidative stability, and improved CE, enabling Cu∥NMC532 AF-LMBs to maintain 80% capacity and >99 average CE after 100 cycles at 0.3C. Compared with low-entropy electrolytes (LEE), high-entropy electrolytes (HEE) use multisolvent and anion cocktails to diversify solvation and promote anion-first reduction, resulting in inorganic-rich interphases, smoother plating, and higher CE than LEE.^291,292^ An HEE developed by dissolving LiFSI in DME/DEE/DEGDME/TTE/BTFE solvents (Figure 28(b)) that increases molecular diversity to raise configurational entropy, weaken ion aggregation, and still maintain anion-dominated solvation; in AF-LMBs, this delivers reliable operation for ∼80 cycles at up to 2 C with ∼70% capacity retention.^293^ In another case, a weakly solvating/weakly dissociating design (Figure 28(c)) tunes solvent donor strength and salt dissociation constant to stabilize anion-rich sheaths, pushing anion reduction into the inner Helmholtz plane and yielding LiF-rich interphases; this produces compact Li plating with 98.7% CE and pouch cells reaching 442.5 Wh kg^–1^ with 80% retention after 100 cycles.^294^ Structuring entropy-enabled mixing and intentionally weakening solvation promotes anion-controlled interfacial chemistry. Solvation-structure engineering enhances conductivity and SEI formation while reducing side reactions. In anode-free sodium metal battery (AF-SMB), the weakly solvating anion-stabilized (WSAS) electrolyte balances Na^+^-solvent and anion interactions.^295^ Figure 28(d) shows it promotes CIP/AGG clustering at the interface (vs strongly solvating anion-disturbed (SSAD)), resulting in a uniform SEI and stabilizing 4.0 V layered oxides across various temperatures. Operationally, WSAS = 2.0 M NaPF_6_ in DEE, whereas SSAD = 2.0 M NaPF_6_ in DME; WSAS weakens solvent coordination and strengthens Na^+^-PF_6_^–^ association (CIP/AGG) relative to SSAD, which favors SSIPs. Under identical coin-cell conditions with layered Na­(Ni_0.3_Fe_0.4_Mn_0.3_)­O_2_ (NFM) (2.0–4.0 V, 0.5 C), WSAS delivers 82.3% capacity retention at 100 cycles and an average plating/stripping CE of 99.89% (1 mA cm^–2^, 1 mAh cm^–2^), while SSAD shows inferior retention and less inorganic-rich SEI. In practical multilayer pouch cells (≈180 mAh; E/C = 4.0 g Ah^1–^), WSAS maintains ≥ 80% after 50 cycles and preserves 74.3% capacity at −30 °C. In practice, these blends act as “function splitters”: one component mainly transports ions and determines viscosity, while the other facilitates desirable SEI/CEI precursors and directs interfacial reactions toward inorganic products.

**Figure 28:**
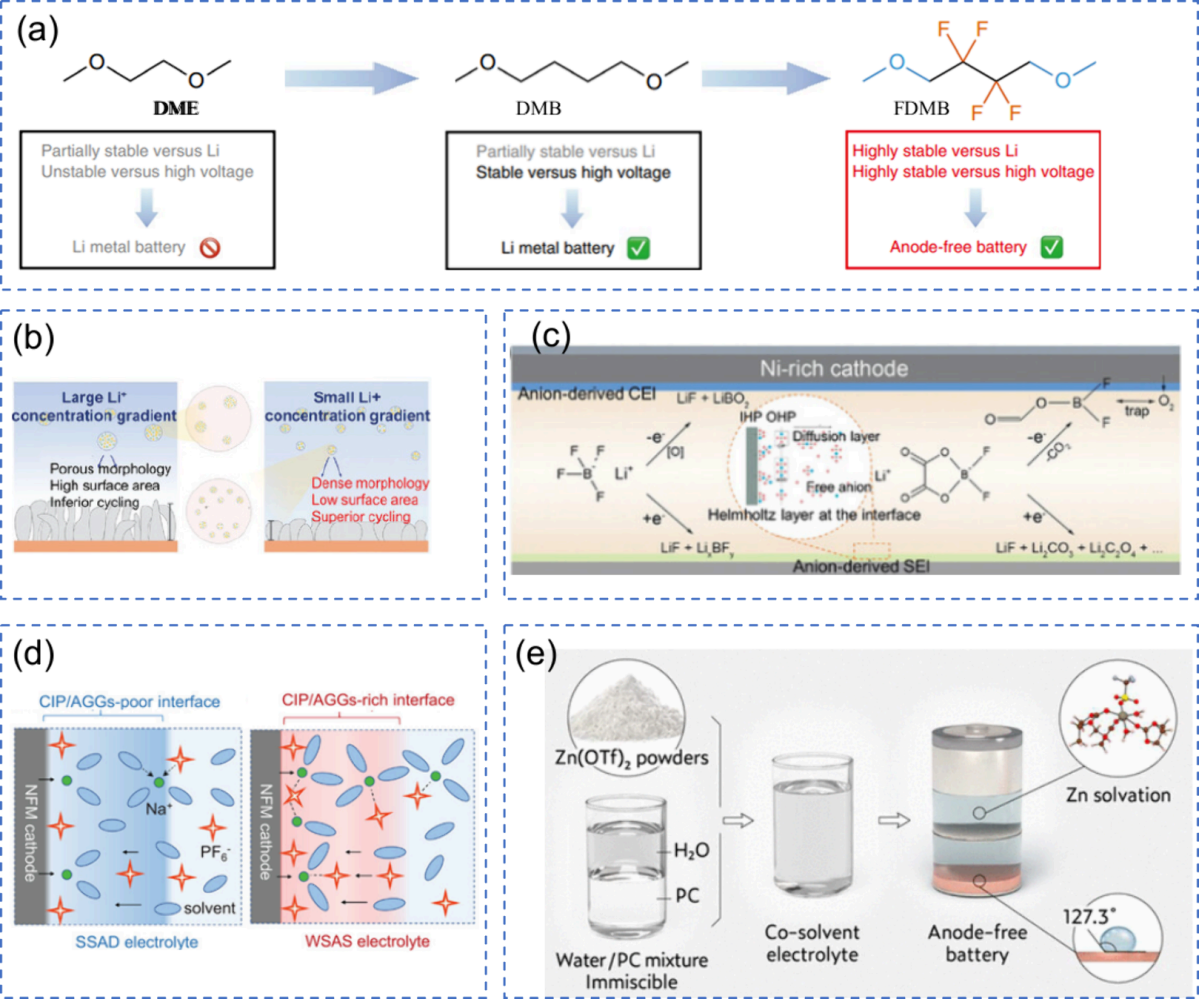
(a) design molecular structure transformation from DME → DMB to FDMB in the process of solvent fluorinating. Reproduced with permission,^290^ Copyright 2020 Springer Nature. (b) Comparison between low entropy electrolyte (left) and high entropy electrolyte (right). Reproduced with permission,^293^ Copyright 2023, Springer Nature. (c) Illustration showing how weak solvation and dissociative electrolytes form anion-rich solvated structures and interfaces. Reproduced with permission.^294^ Copyright 2023, Springer Nature. (d) Illustration of interfacial interaction using SSAD and WSAS electrolytes. Reproduced with permission,^295^ Copyright 2024 Wiley-VCH GmbH. (e) Diagrams showing the preparation process of a cosolvent electrolyte, the resulting zinc solvation structure, and the formation of a hydrophobic interface in an AFB setup. Reproduced with permission,^239^ Copyright 2022 American Chemical Society.

In aqueous battery systems, functional cosolvents decrease free-water activity and modify both the hydrogen-bond network and cation solvation structure, expanding the practical electrochemical window while reducing HER and dendritic growth.^296,297^

Lowering free H_2_O shifts the primary solvation shell of Zn^2+^ from solvent-rich to anion-involved coordination, which increases the Zn^2+^ Δ*G*_desolv_ and promotes an anion-derived interphase that yields smoother Zn deposition. Aqueous systems have a limited electrochemical window, are prone to HER, and develop Zn dendrites; functional cosolvents address these issues by lowering water activity and modifying solvation. Alcohols and polyols (such as ethanol, methanol, ethylene glycol, glycerol) decrease free-water activity. For example, 2 M ZnSO_4_ in water/glycerol (9:1 v/v) enables remarkably stable Zn plating and stripping, even at high current densities.^298^ This occurs because alcohols partly replace H_2_O in the Zn^2+^ sheath, which increases the HER overpotential and leads to smoother operation deposits.^299^ Dimethyl sulfoxide (DMSO) and related sulfoxides coordinate strongly with Zn^2+^, compacting deposits and suppressing corrosion.^300,301^

Hybrid aqueous–organic systems, such as those with a propylene carbonate (PC)/water solution, as shown in Figure 28 (e), suppress unwanted side reactions and dendrite formation.^239^ Since PC and water are immiscible and most Zn salts do not dissolve well in PC, the addition of 1 M ZnSO_4_ induced a salting-out effect that attracted water molecules to dissolve within PC. This process enhanced phase separation, reducing water activity in the electrolyte.^302^ The resulting anion-participating solvation and anion-derived interphase are consistent with the regime depicted in Figure 27. Therefore, the aqueous cosolvent functions similarly to the nonaqueous case: it suppresses the most reactive species (free H_2_O or high-HOMO solvent). It enriches the interface with inorganic, mechanically durable products.^303,304^ Significantly, the cosolvent not only “dilutes” water but also changes the primary solvation shell, shifting the deposition morphology from dendritic to compact by modifying the double-layer structure and desolvation energetics.^305,306^

Solvent and cosolvent engineering is a versatile tool but involves practical trade-offs: (i) in organic systems, fluorinated media can be costly and environmentally problematic, while high nitrile content raises flammability and toxicity risks; (ii) in aqueous systems, heavy cosolvation may reduce conductivity and cause phase separation, and alcohol-rich mixtures often perform poorly at low temperatures due to high viscosity; and (iii) across both, formulation complexity makes mechanistic understanding and scale-up more challenging. Nonetheless, the core principle remains: by designing solvation structures, one controls interfacial reactions. By carefully pairing complementary solvents and considering salt structure, electrolytes can be tailored to enhance conductivity, interfacial stability, and dendrite suppression, thereby fulfilling the strict requirements of AFB operation.

#### 6.1.2 Electrolyte Concentration

Because concentration directly affects the local coordination environment, it establishes the “initial conditions” that determine desolvation barriers, SEI composition, transport, and ultimately plating morphology.^88,216,290,312^ In liquid electrolytes, metal cations are solvated by surrounding solvent molecules and, depending on concentration, coordinated by anions. The identity of the solvation sheath influences both desolvation energetics (Δ*G*_desolv_) during metal deposition and the resulting SEI chemistry after reductive decomposition.^312^ In dilute electrolytes (e.g., 1 M LiPF_6_ in carbonate or 1 M ZnSO_4_ in water), cations are mainly surrounded by solvent molecules, leaving a significant portion of “free” solvent uncoordinated.^4,28,217,313^ These free solvent molecules are the primary cause of side reactions, solvent reduction at the anode, hydrogen evolution in aqueous systems, and cointercalation into cathodes, establishing a baseline where even minor parasitic pathways can become rate- and lifetime-limiting in AFBs. AFBs are especially vulnerable to such inefficiencies because the lack of an excess anode means that every lost electron–ion pair leads to irreversible capacity loss. Therefore, adjusting electrolyte concentration to reduce free solvent activity is a key strategy for both organic and aqueous chemistries, meaning it shifts reduction from solvent-rich to anion-rich coordination at the interface and directs decomposition to areas where it can form protective species.^4,28,217,313^

The concept of high-concentration electrolytes (HCEs) originates in the Li-ion field, where increasing the salt concentration from ∼1 M to greater than 3–5 M dramatically alters the solvation structures. In LiFSI-DME or LiFSI-DOL systems, for example, Li^+^ cations become coordinated predominantly by anions (FSI^–^, TFSI^–^) rather than solvent. This has two consequences:^313,314^

(i). Reduced solvent reactivity: Fewer “free” ether or carbonate molecules are available to be reduced, which lowers parasitic currents.
(ii). Inorganic-rich SEI formation: The decomposition of anion-coordinated solvation complexes yields inorganic-rich SEI, which is both mechanically robust and ionically conductive, thereby coupling the kinetic suppression of parasitic reactions with mechanically stiffer interphases that favor uniform metal deposition.

As shown in Table 3, this approach has been employed in several studies, resulting in improved AFB performance. For Li–Cu asymmetric cell, HCEs have been shown to improve CE from approximately 97–98% in 1 M systems to about 99.3–99.5% in 4–5 M systems.^315^ Full cells with NMC811 cathodes and Cu CCs maintain over 76% capacity for 100 cycles,^316^ a performance that is not achievable with dilute electrolytes. This pushes CE into the practical range needed for long-lasting AFB cycling at realistic E/S ratios. Challenges of HCEs include high viscosity and poor wettability, which limit ion transport and scale-up.^317,318^ In practice, AFBs require both interfacial stability (from concentrated environments) and processability (from dilute-like transport). This prompts the development of localized HCEs (LHCEs),^218^ recognizing that while HCEs improve interphase chemistry, they strain mass transport; thus, LHCEs aim to preserve the former while easing the latter. A comparison of conventional electrolytes versus HCEs and LHCEs, and the resulting interphase textures on the CC, is demonstrated in Figure 29. It is observed that the transition from a rough, organic-rich SEI in dilute electrolytes to an inorganic-rich, smoother interphase occurs as anion-rich coordination develops in HCEs/LHCEs.

**Figure 29:**
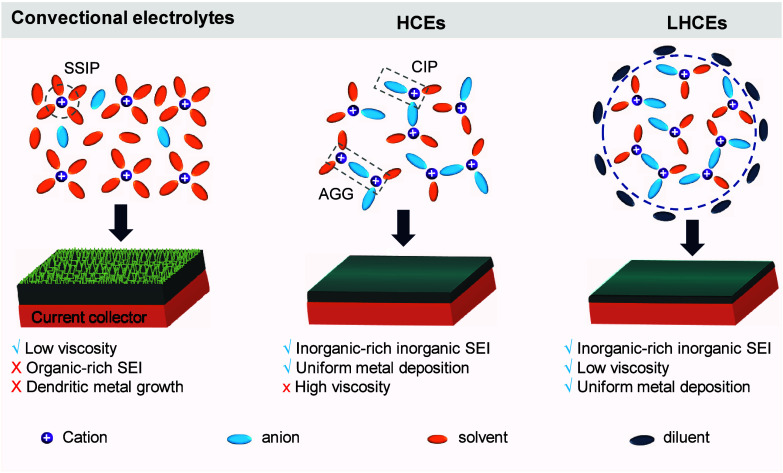
Schematic comparison of conventional electrolytes, HCEs, and LHCEs at a CC in anode-free metal batteries.

**Table 3:** Performance of AFBs with Highly Concentrated and Locally High-Concentration Electrolytes

Battery type	Cell configuration	Electrolyte	Potential window (V)	Capacity (mAh cm^–2^)	Current	Cycle	CR (%)	CE (%)	Ref.
AF-LMB	Cu||LFP	4 M LiFSI in DME, HCE	3–3.8	1.71	0.2 mA cm^–2^	100	54	98.8	^28^
	Cu||NMC523	3 M LiFSI in DOL/DME, HCE	3–3.8	-	0.3C	100	40	-	^88^
	Cu||LFP	3 M LiFSI in DME/DOL (1:1 v/v) HCE	3–3.8	1.6	0.3C	100	40	98.8	^342^
	Cu||NMC622	4.6 M LiFSI + 2.3 m LiTFSI in DME, HCE	3–4.4	1.44	0.1/0.33C	55	54	-	^343^
	Cu||NMC111	2 M LiPF_6_ in EC/DEC (1:1vol.) + 50% vol. FEC, LHCE	2.5–4.5	2.0	0.1C	50	40	97.8	^218^
	Cu||NMC811	0.1 M LiTFSI in FEC/FB, LHCE	3–4.3	-	0.3C	50	50	-	^344^
	Cu||NMC111	1 M LiPF_6_ in FEC/TTE (3:7 v/v), LHCE	2.5–4.5	2.0	0.5 mA cm^–2^	65	50	98.7	^345^
	Cu||NMC811	1 M LiPF_6_ in AN2-DME/FEC/TTE (2:2:1 v/v), LHCE	3–4.3	4.25	0.5C	100	76	98.4	^316^
	Cu||NMC811	1 M LiFSI/1.2DME/2HFE in molar ratio)), LHCE	3.5–4.4	4.0	0.33C	200	54	>90	^346^
	Cu||LiNi_0.95_Mn_0.015_Co_0.02_Al_0.01_Mg_0.005_O_2_	LiFSI:DME: TTE (1:1.2:3 mol/mol)	3.5–4.4	1.6	0.2C	100	50	>99	^347^
AF-SBs	Cu||Li_2_S	[Li(Sulfolane))_2_][TFSA_0.9_FSA_0.1_]-2HFE, LHCE	1.1–3.3	3.27	0.4 mA cm^–2^	75	22.6	96.7	^348^
AF-SMB	Al||(NNCFM)	3A zeolite in 1 M NaPF_6_ in G2	2–4.25	0.8	0.3C	250	67.8	-	^326^
AF-ZMB	Cu||Z-PANI	1 M ZnSO_4_ and 4 M EMImCl in H_2_O, WiSE	0.5–1.5	-	1 A g^–1^	300	78.7	99.9	^331^

LHCEs are formed by diluting an HCE with an inert, nonsolvating cosolvent such as hydrofluoroethers or fluorobenzene. Although the bulk molarity is lowered, the local solvation structure remains HCE-like: cations remain coordinated with anions, while the diluent acts only as a physical spacer. The advantages are evident, including a 30–50% reduction in viscosity, improved wettability on porous cathodes, and facilitated practical cell assembly, while maintaining robust interfacial chemistry.^218,319,320^ The SEI is still fluorine-rich, which suppresses dendritic metal growth, effectively decoupling bulk transport from local coordination, so that thinning the medium does not reintroduce free solvent activity.

For the first time in 2019, our research group investigated the use of LiPF6-based LHCE in carbonate-based solvents, utilizing fluoroethylene carbonate (FEC) as a diluent for AF-LMB.^218^ As demonstrated in Figure 30(a), LHCEs incorporate specific diluents into their formulation, concentrating lithium ions near the electrode interface where their presence is most critical for efficient cycling. These diluents are carefully selected to maintain a high-concentration solvation structure in targeted areas while simultaneously reducing the overall viscosity and salt concentration of the electrolyte.

**Figure 30:**
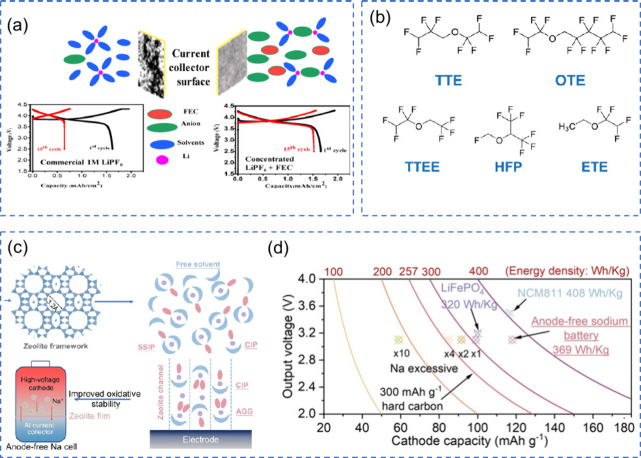
(a) Illustration and representative cycling curves showing how LHCEs employ specific diluents to concentrate lithium ions near the electrode interface, promoting stable interfacial reactions and efficient cycling. Reproduced with permission,^218^ Copyright 2019 American Chemical Society (b) Molecular structures of common diluents used in preparing LHCEs. (c) Construction of a high-voltage AF-SMB. (d) Energy density comparison of AF-SMBs, Li-ion batteries, and NMBs. (c, d) Reproduced with permission,^326^ Copyright 2022 Wiley-VCH.

Fluorinated ethers are commonly used as diluents in LHCEs, including 1,1,2,2-tetrafluoroethyl-2,2,3,3-tetrafluoropropyl ether (TTE), ethyl 1,1,2,2-tetrafluoroethyl ether (ETE), 1,1,1,3,3,3-hexafluoro-2-(fluoromethoxy) propane (HFP), 2,2,2-trifluoroethyl 1,1,2,2-tetrafluoroethyl ether (TTEE), and 1H,1H,5H-octafluoropentyl 1,1,2,2-tetrafluoroethyl ether (OTE), as shown in Figure 30(b). These LHCEs have a unique solvation structure, depicted in Figure 30(c), characterized by tightly packed clusters of salts and solvents interspersed with nonsolvating diluent molecules. LHCEs typically comprise three main components: (i) ion-conducting salt (ii) solvating solvent, and (iii) nonsolvating diluent. This composition allows LHCEs to form a robust and stable SEI on the lithium-metal surface, preventing dendrite formation and extending the battery’s cycle life. Additionally, LHCEs exhibit enhanced chemical stability, which reduces the likelihood of degradation and capacity loss over time.^218,319–321^ However, their complex formulation process and associated costs remain significant obstacles to large-scale commercialization.

Similar to AF-LMBs, both HCEs,^322,323^ and LHCEs,^324–326^ suppress Na dendrites in AF-SMBs. An HCE composed of 5 M NaFSI in DME resulted in highly stable cycling during sodium plating/stripping.^323^ To address the viscosity and cost issues of HCEs, BTFE was used as a diluent to prepare an LHCE (2.1 M NaPF_6_ in DME/BTFE, 1:2 molar ratio), which achieved highly reversible sodium plating and stripping.^325^ To further realize the HCE approach, recently Lu et al. introduced a novel electrolyte design that exceeds the solubility limit by using 3A zeolite molecular sieves in 1 M NaPF_6_ in G2.^326^ These sieves help move the highly reactive desolvation process away from the electrode surface, utilizing the size effect (Figure 30(d). The A||NaCu_1/9_Ni_2/9_Fe_1/3_Mn_1/3_O_2_ (NNCFM) AF-SMB achieved 94.1 mAh g^–1^ capacity showed excellent cycling stability, with capacity retention of 67.9% after 250 cycles at 0.3C. Similarly, in anode-free potassium metal batteries (AF-PMBs), HCEs of 3.75 M KFSI in TEP:DMC (2:1 v/v) demonstrate excellent reversibility in plating and stripping within K||Al asymmetric cells, achieving an average CE of approximately 98.9% over 1200 cycles. With Al||graphite system, it exhibits stable cycling for over 200 cycles, maintaining a high capacity retention rate 100%.^327^ However, oxidative stability remains a limitation due to the high HOMO of ether solvents, so solvent selection becomes progressively more constraining from Li to K as the oxidative ceiling drops.^328^

The aqueous analogue of HCEs is the “water-in-salt electrolyte” (WiSE). The WiSE systems invert the solvent–solute ratio: water molecules become fully coordinated within cation solvation shells, leaving negligible free water. This suppresses the hydrogen evolution reaction (HER) and extends the electrochemical stability window.^329,330^ It functions similarly to HCEs/LHCEs by reducing free-water activity and altering interfacial decomposition pathways. 1 M ZnSO_4_ + 4 M EMImCl WiSE enables dense, dendrite-free Zn plating with over 300 cycles of life in Cu||Z-PANI Zn AF-ZMBs, achieving 78.7% capacity retention and 99.9% CE.^331^ In Al-based AFBs systems, concentrated AlCl_3_-based WiSEs enable reversible Al plating and stripping at room temperature, though corrosivity remains a concern.^332^ The expanded stability window results from water molecules locked within ion pairs or clusters rather than from additive-derived passivation alone. In WiSEs, cations are often coordinated simultaneously by both water and anions, leading to complex solvation motifs (e.g., Zn^2+^-TFSI^–^-H_2_O bridges).^333–335^ This hybrid coordination facilitates the formation of protective interphases (ZnF_2_, Zn_3_(PO_4_)_2_) instead of corrosive ZnO, creating the mechanistic link between bulk solvation structure and inorganic-rich, corrosion-resistant interphases on Zn and Al. This concentration-driven evolution in aqueous systems, and the associated suppression of HER at the electrode, is illustrated in Figure 31 (“From dilute to WiSE”).

**Figure 31:**
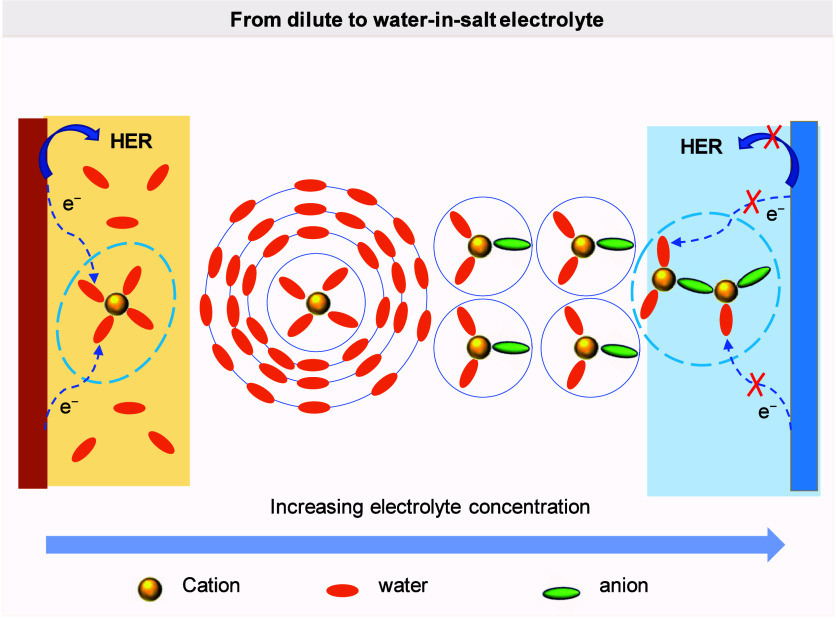
Evolution of interfacial solvation and HER behavior with increasing electrolyte concentration. Left (dilute electrolyte): solvent-dominated cation coordination and abundant free H_2_O near the anode enable electron access and hydrogen-evolution reaction (HER). Right (highly concentrated/anion-rich interface): a tight primary sheath with anion participation depletes free water at the anode, blocking electron pathways and suppressing HER.

Concentration tuning restructures the solvation shell and shifts the balance among various parameters, including voltage window, ionic conductivity, density, viscosity, wettability, and cost, as shown in Figure 32. Moving from dilute solutions to HCEs generally widens the voltage window by making the inner shell anion-rich and promoting interphase formation. However, these benefits come with trade-offs: viscosity and density increase, wettability worsens, material costs rise, and ionic conductivity becomes nonmonotonic (initially improving, then decreasing as viscosity limits mobility)^317,318^ Figure 29 emphasize that LHCEs address these issues by preserving the anion-rich inner shell while lowering bulk viscosity with a noncoordinating diluent, partially restoring ionic conductivity and wettability, and reducing density and cost compared to HCEs. Still, they are not inexpensive and may introduce volatility or environmental concerns. In organic electrolytes, HCEs are effective at building interphases but remain costly and can fail near the upper end of the voltage window (>4.5 V) due to anion or diluent oxidation, aluminum corrosion, or gas evolution. High viscosity also impairs wetting and increases polarization. LHCEs tackle these issues while maintaining anion-first reduction chemistry.

**Figure 32:**
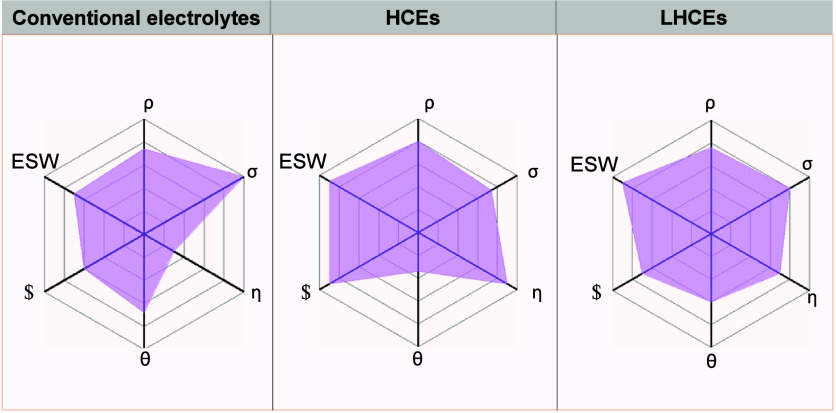
Qualitative benchmarking of dilute electrolytes, HCEs, and LHCEs. Radar plots compare the relative values of voltage window (ESW), ionic conductivity (σ), viscosity (η), wettability (θ), density (ρ), and cost ($).

The use of conventional aqueous electrolytes limits the practical voltage window due to water splitting (∼1.23 V vs SHE), which restricts Zn-based cells to cathode plateaus typically around 1.6–1.9 V vs Zn/Zn^2+^ under neutral or mildly acidic conditions.^336,337^ However, WiSEs extend the apparent voltage window toward ∼3 V,^338^ but require large amounts of expensive salts (e.g., LiTFSI, Zn­(TFSI)_2_) and increase the density and viscosity of the electrolyte, which worsens low-temperature performance and manufacturability.^339^ WiSE-like variants (hydrotropes, mixed-anion strategies) are helpful but rarely match the low cost of brines and can experience crystallization or phase separation.^340,341^ Practically, the ideal concentration must meet the cathode-imposed voltage, enable effective transport, preserve the wet porous electrodes, and remain within cost and safety limits. Nevertheless, electrolyte concentration and solvation control provide the most fundamental lever for stabilizing AFBs across chemistries. All subsequent electrolyte strategies, multisalt design, cosolvent selection, and additive engineering are best understood as refinements of solvation structure control.

#### 6.1.3 Anion and Cation Synergy (Dual or Multisalt Systems)

Unlike high-concentration strategies that mainly suppress free-solvent activity, dual/multisalt electrolytes improve performance by deliberately increasing chemical complexity in the ionic environment.^4,141,331,347,349–352^ Using two or more salts enables control over (i) solvation structure, as different cation–anion pairs influence coordination equilibria and modify solvent–ion interactions, thus impacting desolvation barriers; (ii) interphase chemistry, where one component can decompose sacrificially to produce desirable SEI/CEI species (such as fluoride-rich layers) while the other salt maintains bulk stability; (iii) transport properties, since mixed anions and cations provide ways to balance ionic conductivity, cation transference number, and viscosity; and (iv) electrochemical stability, as careful pairing can expand both reductive and oxidative limits beyond what a single salt can achieve. These features are especially beneficial for AFBs, which need to prevent filamentary metal growth at the CC and protect high-voltage cathodes from dissolution or crossover.^239,240,353,354^ By distributing complementary roles across different salts, well-designed mixtures can address both issues without sacrificing processability. As illustrated in Figure 33, moving from a single-salt electrolyte to a dual or multisalt formulation replaces a brittle, organic-rich SEI and dendritic deposition with a fluoride-rich, mechanically robust SEI and dense, columnar plating at the CC, lowering overpotential and extending cycle life, as also seen from the results of various reports summarized in Table 4.^355,356^

**Figure 33:**
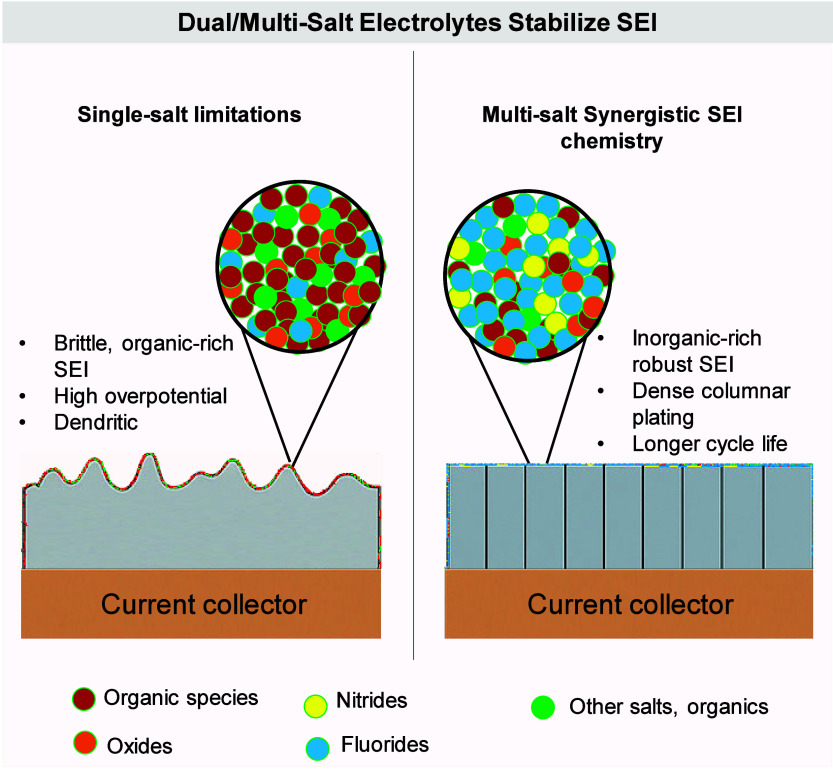
Schematic illustration of how single- and multisalt electrolytes affect SEI stability and deposition regulation; Left: single-salt, and right: multisalt.

**Table 4:** Performance of AFBs with Dual or Multisalt Systems

Battery type	Cell configuration	Electrolyte	Potential window (V)	Capacity (mAh cm^–2^)	Current	Cycle	CR (%)	CE (%)	Ref.
AF-LMB	Cu||LFP	2 M LiFSI + 1 M LiTFSI in DME/DOL (1:1 vol.)	3–3.8	1.6	0.12C	100	33	98.90	^357^
	Cu||LFP	1 M LiNO_3_+1 M LiFSI in DME	3–3.8	-	0.5 mA cm^–2^	50	66	-	^390^
	Cu||NMC532	1 M LiDFOB + 0.2 M LiBF_4_ in FEC: DEC (1:2 vol.)	3.6–4.5	2.4	0.2/0.5C	90	80	99.75	^141^
	Cu||NMC111	0.9 M LiTFSI + 0.3 M LiDFOB in FEC/TTE (2:3 vol.)	2.5–4.5	2.2	0.5 mA cm^–2^	100	37	98.60	^358^
	Cu||NMC532	2 M LiDFOB + 1.4 LiBF_4_ in FEC/DEC (1:2 v/v)	3.6–4.5	3.1	0.2/0.5C	200	80	99.89	^391^
	Cu||NMC811	1 M LiBF_4_ + 1 M LiDFOB in tFEP/FEC (1:2 v/v)	3–4.6	4.64	0.1/0.5C	100	80	98.70	^392^
	Cu||NMC811	0.5 m LiDFOB + 0.5 m LiBF_4_ in FEC: DEC (1:2 v/v))	3.6–4.5	2.4	0.2/0.5C	90	80	-	^393^
	Cu||LFP	0.2 M LiPF_6_ + 3.8 M LiFSI in EC/DEC/EMC/TEP (1.5:2.5:1:5, v/v) + 5 wt % FEC+ 2 wt % VC + 0.2 wt % LiNO_3_	2.8–4.2	0.5	0.5 mA cm^–2^	50	60	-	^394^
	Cu||LiNi_0.95_Mn_0.015_Co_0.02_Al_0.01_Mg_0.005_O_2_	0.6 M LiDFOB and 0.6 M LiBF_4_ in FEC: DEC(1 : 2 v/v).	3.5–4.4	1.6	0.2C	50	25	∼98	^347^
AF-SMB	p-Al@C||Na_3_V_2_(PO_4_)_3_	0.6 M NaOTF + 0.4 M NaBF4 in G2	2.8–3.8	1.4	0.05C	50	60	93.8	^395^
	graphitic carbon@Al||Na[Cu_1/9_Ni_2/9_Fe_1/3_Mn_1/3_]O_2_	0.9 m NaPF_6_ and 0.1 m NaBF_4_ in G2	2–4	2.0	0.5 A g^–1^	260	80	-	^190^
AF-ZMB	Cu||Z-PANI	1 M ZnSO_4_ and 4 M EMImCl in H_2_O	0.5–1.5	154.4 mAh g^–1^	1 A g^–1^	300	78.7	99.9	^331^

Dual-salt electrolytes in organic liquid electrolytes, such as those in AF-LMBs and other types of AFBs, have been widely studied (Table 3). These dual-salt electrolytes, such as LiFSI + LiTFSI in DME/DOL (1:1, v/v), have been investigated.^357^ LiFSI enhances SEI stability by decomposing into LiF, while LiTFSI boosts oxidative stability at high-voltage cathodes. For example, a dual-salt electrolyte (2 M LiFSI + 1 M LiTFSI in DME/DOL) improved the average CE to as much as 99% in Cu||LiFePO_4_ AF-LMB over 100 cycles at 0.2 mA cm^–2^.^357^ The electrolyte formed an SEI layer is rich with inorganic components like LiF and Li_2_O, as illustrated in Figure 34(a). Significantly, the LiFSI: LiTFSI ratio influences both viscosity and solvation structure: FSI^–^ promotes stronger Li^+^ coordination, while TFSI^–^ offers bulkier, weaker coordination. Therefore, composition is not just a passive mixture but an active “dial” that comodulates rheology, solvation symmetry, and interphase formation. Other examples include LiBOB + LiTFSI, where BOB^–^ anions decompose to form a robust Li_2_CO_3_/Li_2_O interphase, complementing LiF produced from TFSI^–^.^44,294,358^ In such systems, the cathode electrolyte interphase (CEI) is stabilized, reducing transition-metal dissolution. These pairings couple a reductively sacrificial anion (BOB^–^/FSI^–^) with a high-voltage-tolerant anion (TFSI^–^), aligning anode and cathode requirements within a single formulation.^347,359^

**Figure 34:**
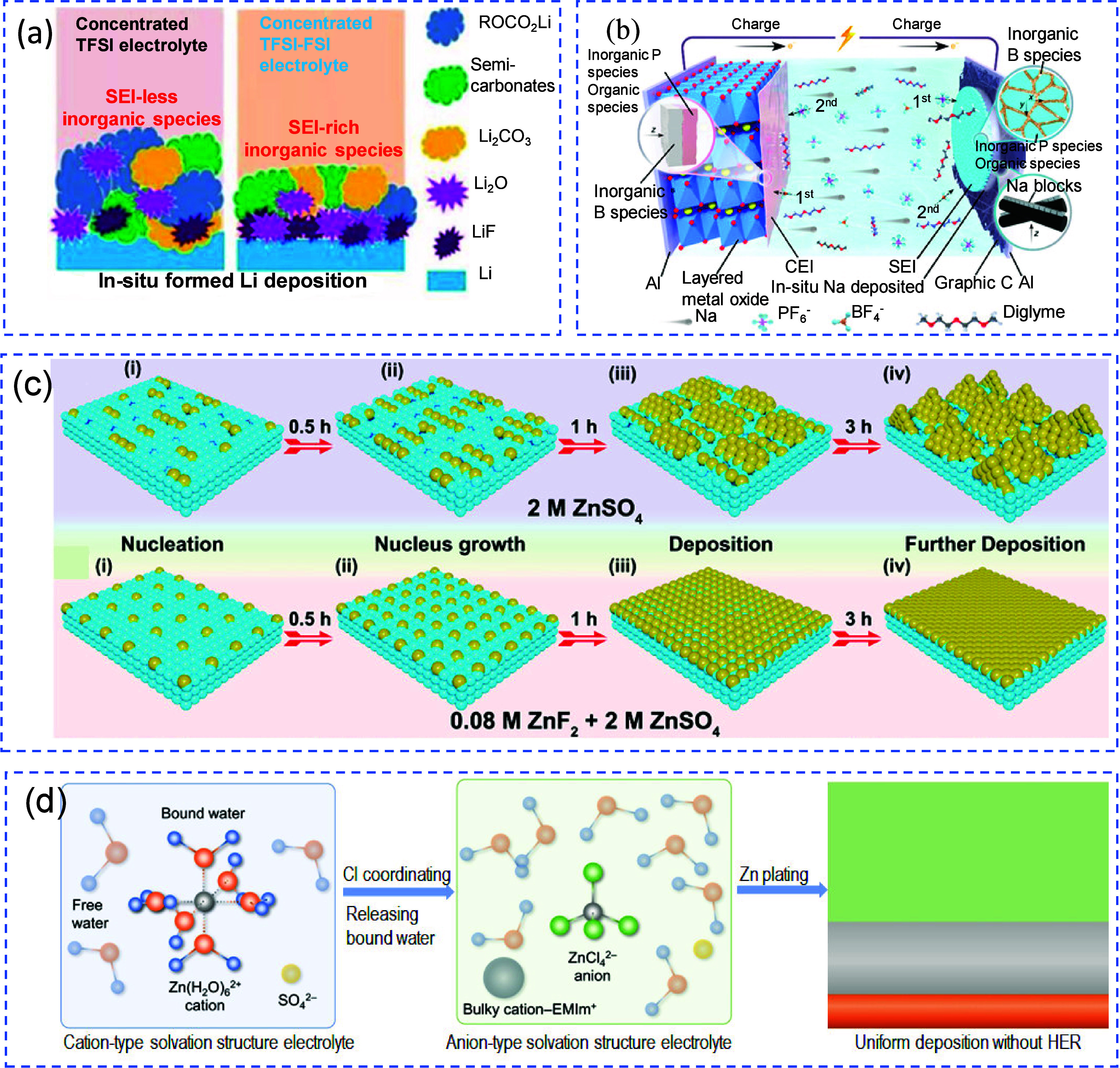
(a) schematic of estimated SEI formed in 2 M LiFSI+1 M LiTFSI (dual salt)) in DME/DOL (1:1, v/v). Reproduced with permission,^357^ Copyright 2019, The Author(s) Published by The Electrochemical Society. (b) Schematic of an AF-SMB featuring a cathode made from layered metal oxide, an anode comprising aluminum foil coated with graphitic carbon, and an electrolyte solution containing 0.1 M NaBF_4_ and 0.9 M NaPF_6_ in diglyme. Reproduced with permission,^190^ Copyright 2022 Springer Nature. (c) Schematic representation illustrating zinc deposition processes with and without the use of ZnF_2_ as an electrolyte additive. Reproduced with permission,^240^ Copyright 2021 John Wiley & Sons. (d) Conceptual strategy for designing an anion-based, water-free Zn^2+^ solvation structure electrolyte. Reproduced with permission,^331^ Copyright 2021 Wiley-VCH GmbH.

In AF-SMBs and AF-PMBs, dual salts are less advanced but follow a similar logic. For AF-SMBs, NaFSI + NaClO_4_ combine high ionic conductivity (from ClO_4_^–^) with interphase stability (from FSI^–^ decomposition). Formulations combining NaPF_6_, NaClO_4_, NaTFSI, NaFSI, NaBF_4_, and NaSO_3_CF_3_ in dipolar aprotic solvents foster thin, stable SEI/CEI layers, boosting average CE and cycle life.^265,266,360^ For example, 0.9 M NaPF_6_ + 0.1 M NaBF_4_ electrolyte in G2 formed an amorphous interphase: B–O species spread 2D on the SEI outer layer and three-dimensional (3D) within the CEI (Figure 34(b)).^190^ The robust SEI suppresses dead Na/dendrites and heals plating/stripping cracks, while an ultrathin CEI protects the cathode and limits transition-metal dissolution; together, synergies at the collector/Na, Na/electrolyte, and electrolyte/cathode interfaces enable highly reversible Na deposition.

Dual salts containing KFSI + KPF_6_ in the AF-PMB are also examined, where PF_6_^–^ enhances oxidative stability, but KFSI ensures an inorganic-rich SEI formation.^361^ However, oxidative stability above 4.0 V remains a limiting factor for potassium battery electrolytes.^362–364^ As a result, cation type determines the accessible voltage range, while the anion mix influences how close operation can get to that limit without speeding up parasitic reactions. Overall, in these systems, mixed-anion solvation alters the cation solvation number (CN) and changes the free energy of ion transport.^365,366^ Spectroscopic and molecular dynamics studies reveal that dual-salt electrolytes form “hybrid” solvation sheaths, where Li^+^ or Na^+^ coordinates with both FSI^–^ and TFSI^–^ simultaneously, thereby directly affecting the SEI composition. This sheath-level hybridity is the main reason linking bulk composition to interphase chemistry and, ultimately, to deposition morphology and high-voltage tolerance.^365,367–369^

Multisalt systems in aqueous AFBs, such as zinc systems, face challenges like dendritic Zn plating and parasitic hydrogen evolution.^370^ Dual-salt strategies address these issues by combining salts with complementary roles. For example, in ZnSO_4_ + Zn­(TFSI)_2_: SO_4_^2–^ ensures high solubility and conductivity, while TFSI^–^ modifies Zn^2+^ solvation, reducing water coordination and suppressing dendrites.^371^ Additionally, a dual salt containing Zn­(OTf)_2_ and Me_3_EtNOTf facilitates the in situ formation of a fluorinated and hydrophobic SEI composed of ZnF_2_, ZnCO_3_, ZnSO_3_, and polyanions on the CC’s surface.^353^ The resulting hydrophobic SEI effectively suppressed the hydrogen evolution reaction (HER) while enabling efficient Zn^2+^ ion migration. Additionally, in cases of ZnSO_4_ + halide additives (F^–^ Cl^–^, Br^–^), halides adsorb on Zn, to create a stable fluoride-rich interface on the surface of the CC as demonstrated in Figure 34(c),^240^ or influencing the solvation structure, into a water-free, anion-dominated solvation structure, ZnCl_4_^2–^, as demonstrated in in Figure 34(d),^331^ Here, the “second salt” primarily controls water activity and surface adsorption-not just bulk conductivity-reshaping the double layer to reduce both H_2_ evolution and dendrite nucleation.

In Aluminum Systems, Al^3+^ chemistry requires chloride-rich environments for reversible plating. Eutectic mixtures such as AlCl_3_/1-Ethyl-3-methylimidazolium chloride ([EMIM]­Cl) are effectively dual-salt systems: Al_2_Cl_7_^–^ species stabilize deposition, while EMIM^+^ cations extend the liquidus range and lower the melting point.^372–374^ By adjusting the AlCl_3_:EMIMCl ratio, both conductivity and reversibility can be improved. Similar approaches are used with deep eutectic solvents (AlCl_3_ + urea, AlCl_3_ + acetamide). Practically, changing the Lewis acidity through the AlCl_3_:donor ratio directs the formation of AlCl_4_^–^ and Al_2_Cl_7_^–^, which impacts nucleation overpotentials and metal morphology.^375–377^

In magnesium AFBs, dual salts like Mg­(TFSI)_2_ and AlCl_3_ enable reversible magnesium plating and stripping.^378,379^ Here, AlCl_3_ acts as a Lewis acid, transforming inactive Mg-TFSI solvation structures into active Mg-Cl complexes. This synergy demonstrates how multisalt strategies can overcome the limitations of single salts. It is a classic example of salt pairing that “unlocks” otherwise slow multivalent electrochemistry through targeted ligand exchange.

The performance of multisalt electrolytes involves a deliberate balance between ionic transport and interphase stability, where complementary anion chemistries are combined to meet both needs. In practice, dual-salt designs enable simultaneous control over: (i) conductivity, as adding small, highly mobile anions (e.g., ClO_4_^–^, SO_4_^2–^) maintains bulk ionic conductivity;^380,381^ (ii) interphase stability, as bulky, fluorinated anions (e.g., FSI^–^, TFSI^–^) preferentially reduce or oxidize to form inorganic-rich passivation layers at the anode and resilient CEIs at the cathode;^382–384^ and (iii) overall trade-offs between transport and stability, because pairing small and large anions lessens the conductivity loss caused by the larger anions’ increased hydrodynamic radius and stronger ion pairing.^385,386^ This approach allows room-temperature conductivities of approximately 8–12 mS cm^–1^ while maintaining interfacial integrity. On the metal-anode side, the selective decomposition of the fluorinated component produces LiF/NaF/ZnF_2_-rich or mixed-oxide SEIs, which lower desolvation barriers and prevent filament growth. At the cathode, the same bulky anions create compact CEIs that reduce solvent attack and transition-metal dissolution.^387–389^ This dual protection is especially important for AFBs operating at high current densities, where both interfaces are under stress, and failure on either side quickly leads to capacity loss and potential short circuits. A helpful design guideline, therefore, is to pair a “transport” anion (small, mobile, minimally surface-active) with an “interphase-forming” anion (bulky, fluorinated, surface-reactive), and adjust their ratio to achieve a desirable balance of t^+^, viscosity, and stability.

Dual- and multisalt electrolytes demonstrate how compositional diversity can enable emerging behaviors: in AFBs, where efficiency margins are very narrow, increasing CE from approximately 98% to about 99.5% can, with a fixed end-of-life standard, nearly double the cycle life because cumulative capacity retention scales exponentially with CE. However, several challenges temper this optimizm: (i) the cost and supply issues of specialty salts (e.g., LiFSI, Zn­(TFSI)_2_); (ii) corrosion and parasitic reactivity risks in Cl^–^-containing systems; and (iii) the analytical complexity of untangling composition-dependent solvation structures and interphase chemistry in mixed-salt solutions. Practical progress relies on mapping compositions that distinguish “transport” and “interphase-forming” roles, standardized reporting of t^+^, viscosity, and conductivity under application temperatures, and real-time characterization (Raman/NMR/XPS) combined with MD/DFT to establish cause and effect. Thus, future reports should go beyond nominal molar ratios to mechanistic descriptors, such as coordination numbers, free-solvent fraction, and contact-ion-pair/aggregate populations, that directly connect formulation to interphase outcomes. When feasible, operando multimodal probes (Raman/NMR/XPS) coregistered with MD/DFT can complete the link from composition to solvation, interphase, and performance, thereby accelerating rational optimization.

#### 6.1.4 Interphase-Directed Additives

Unlike bulk solvent or salt options, additives are intentionally chosen sacrificial molecules that preferentially reduce or oxidize before the main electrolyte, thereby programming the SEI/CEI composition at trace loadings (typically 0.1–5 wt % or mol %).^396–399^ In practice, additives function as molecular coders of interphase chemistry by (i) lowering the threshold for targeted decomposition products, (ii) influencing anion/cation participation in the early SEI/CEI formation, (iii) adjusting nucleation kinetics and metal morphology from the first cycle, and (iv) achieving significant gains in CE at low cost.^270,400,401^ As demonstrated in Figure 35, interphase-directed additives widen the usable voltage window by moving both stability limits. On the anode, sacrificial reduction of F-rich, nitrate, or phosphite species forms inorganic, high-modulus SEIs (Metal fluorides, M_a_F_b_, e.g., LiF; Metal nitrides, M_a_N_b_, e.g., Li_3_N; oxyfluorophosphate, M_a_PO_b_F_c_, e.g., Li_*x*_PO_*y*_F_*z*_) that block solvent reduction, lower nucleation overpotential, and keep deposition uniform, shifting the cathodic limit to more negative potentials. At the cathode, phosphites/phosphates and fluorinated sulfones oxidize to a robust CEI that suppresses electrolyte oxidation and O-radical attack while binding dissolved transition metals, raising the anodic limit. Therefore, they serve as one of the most economical and flexible tools for electrolyte design in AFBs. Table 5 summarizes studies using this approach as a strategy to address issues in AFBs.

**Figure 35:**
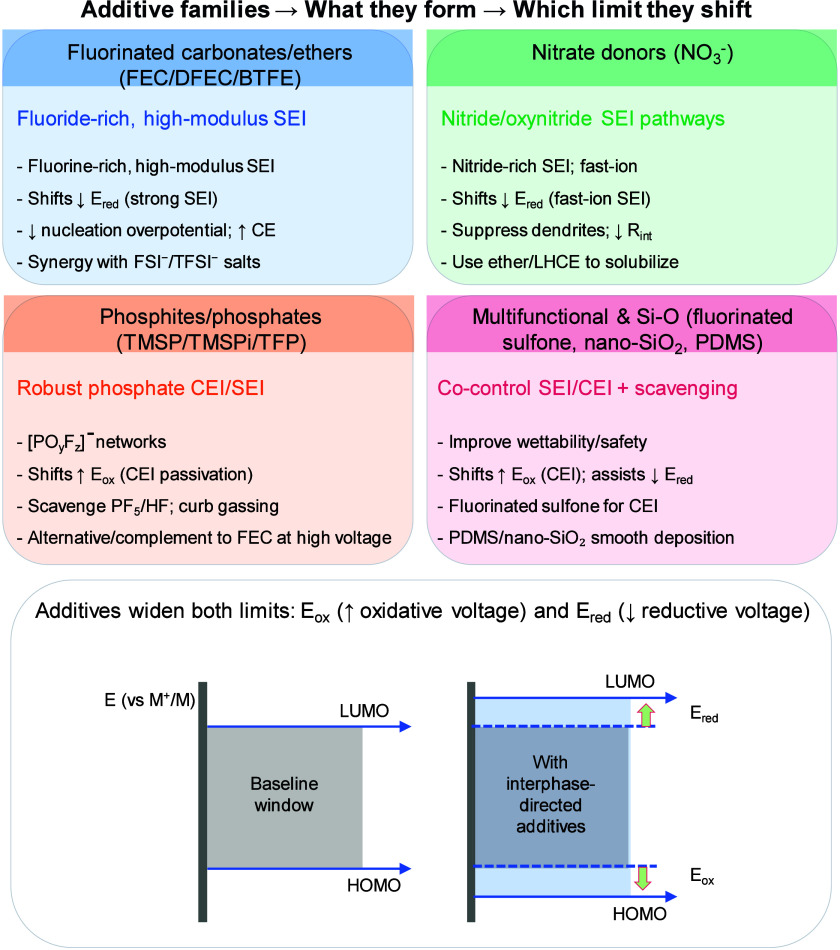
Interphase-directed additives broaden the electrochemical window: fluorinated and nitrate additives form fluoride/nitride-rich SEIs that lower E_red_, while phosphite/phosphate and fluorinated-sulfone chemistries passivate the cathode CEI to raise *E*_ox_, yielding a wider stable operating range.

**Table 5:** Performance of AFBs with Interphase-Directed Electrolyte Additive Strategies

Battery type	Cell configuration	Electrolyte	Potential window (V)	Capacity (mAh cm^–2^)	Current	Cycle	CR (%)	CE (%)	Ref.
AF-LMB	Cu||NMC111	1 M LiPF_6_ in EC/DEC + 0.5 M KNO_3_	2.5–4.3	2.0	0.2 mA cm^–2^	50	40	96.50	^405^
	Cu||LFP	1.2 M LiPF_6_ in EC/EMC with VC	2–4	-	0.1C	50	30	-	^412^
	Cu||NMC811	1 M LiPF_6_ in EC/DMC/EMC with 0.02 M LiDFOB	2.7–4.3	3.0	0.9 mA cm^–2^	50	95	99.4	^165^
	Cu||NMC532	1 M LiPF_6_ in EC: DEC: DMC (1:1: 1, vol.) with 2 wt % LiAsF_6_ and 2 wt % FEC	3–4.3	3.98	0.1/0.25C	50	75	98.3	^402^
	Cu||LFP	1.2 M LiPF_6_ in EC/EMC (3:7 v/v) + 5 wt % VC	2–4	-	0.1C	50	30	-	^432^
	Cu||NMC532	1 M LiPF_6_ in EC:DEC/DMC (1:1: 1, v/v) + 2 wt % LiAsF_6_ + 2 wt % FEC	3–4.3	3.98	0.1/0.25C	50	75	98.3	^433^
	Cu||NMC631	4 M LiFSI in DME + 0.2 M [Pyr_14_][DFOB] + 0.08 M [Pyr_14_][PO_2_F_2_]	2.8–4.35	4.0	1.0 mA cm^–2^	142	80	98.4	^434^
	Cu||NMC111	1 M LiPF_6_ in EC/DEC (1:1 v/v) + 0.5 M KNO_3_	2.5–4.3	2.0	0.2 mA cm^–2^	50	40	96.50	^435^
	Cu||NMC811	1 M LiPF_6_ in EC/DMC/EMC (1:1:1 v/v) + 0.02 M LiDFOB	2.7–4.3	3.0	0.9 mA cm^–2^	50	95	99.4	^436^
AF-SBs	Ni||Li_2_S	1 M LiTFSI + 0.1 M LiNO_3_ in DOL/DME	1.8–2.8	3.1	0.1C	100	56.1	-	^350^
	Ni||Li_2_S	1 M LiTFSI) in DME/DOL (1/1, v/v) + 0.2 M LiNO_3_ + 1.5 mM Nd(OTf)_3_	1.8–2.8	6.344	0.2C	100	53.0	94.0	^437^
	Cu||Li_2_S	1 M LiTFSI) in DME/DOL (1/1, v/v) + 2 wt % LiNO_3_ + 0.5% SiO_2_	1.7–2.8	2.94	0.1C	200	50.3	-	^408^
	Ni||Li_2_S	1 M LiTFSI) in DME/DOL (1/1, v/v) + 0.5 M LiNO_3_ + 0.2 M SnI_2_/LiI	1.8–2.8	8.4	0.1C	100	59.0	-	^438^
	Ni||Li_2_S	1 M LiTFSI) in DME/DOL (1/1, v/v) + 0.25 M LiNO_3_ + 0.1 M LiTe_3_	1.8–2.8	3.65	0.2C	100	71.1	97.5	^439^
AF-SMB	Cu||Na_3_V_2_(PO_4_)_3_	1.0 M NaPF_6_ in diglyme + Nanosilica electrolyte additive	2.6–3.8	0.38	0.5 mA cm^–2^	50	75	-	^409^
	Al@C||NaNi_1/3_Fe_1/3_Mn_1/3_O_2_	1 M NaBF_4_ in G2/G4 + 2 vol % PDMS	2–4.1	2.52	0.1C	100	87	-	^440^
AF-PMB	Al||KPTPAn	0.5 M KPF_6_ in DME:G2 (8:2 v/v)	2–4.3	0.64	0.2C	300	62.5	∼99	^151^
	Cu||KPTCDA	0.4 M KPF_6_ in DME + 2 vol % PDMS	2.5–4.2	0.64	0.2C	50	82	98.9	^410^
AF-ZMB	SUS||LMO	2 M ZnSO_4_, 1 M Li_2_SO_4_, in H_2_O + 0.08 M ZnF_2_ additive	1.4–2.1	0.15	200 mA g^–1^	100	75.64	98.75	^240^
	Ti ||Zn_*x*_VOPO_4_	4 m Zn(OTF)_2_ in H_2_O + 0.5 M Me_3_EtNOTF	0–2.1	0.41	0.5 mA cm^–2^	90	80	-	^353^
	NIL@Zn||Cl_2_ NIL: nucleophilic interfacial layer	1 M ZnSO_4_ and 1 M LiCl in H_2_O + 0.4 M tetramethylammonium chloride	0.9–2.4	0.53	0.2 A g^–1^	200	95	-	^354^
	CuNC@Cu||G/PVP@ZnI_2_	2 M ZnSO_4_ in H_2_O + 10 mM I^3–^	0.6–1.6	0.63	1 A g^–1^	200	63.8	99.91	^420^
	Cu||LFP	4 M ZnSO_4_ and 2 M Li2SO_4_ in H_2_O/DME (9:1 v/v) + 5 mM SnBr_2_	0.4–1.6	1.01	0.5C	100	35.2	99.1	^425^
	Cu||Zn_*x*_aV_2_O_5_@graphene	ZT-electrolyte Zn(OTf)_2_ 4 M Zn(OTf)_2_ + 3 M trimethylazaniumyl acetate	0.2–1.8	0.5	10 A g^–1^	100	71.4	98.7	^238^

Overall, within organic electrolytes for Li-, Na-, and K-based AFBs, four additive families are prominent. First, FEC and related fluorinated additives decompose near ∼1.3 V vs Li^+^/Li to produce LiF-rich (or NaF-rich) SEIs that are mechanically robust; in Li∥Cu AFBs, ∼5 wt % FEC in ether media can raise CE from ∼97.5% to >99.3% while lowering nucleation overpotential.^402^ For instance, in Cu∥NMC111 AF-LMBs, a dual additive (2 wt % KPF_6_ + 2 vol % TMSP) containing 1 M LiPF_6_ in EC/DEC (1:1 vol %) electrolyte achieved much higher average CE over cycles compared to without an additive, attributed to the KPF_6_’s shielding effect and TMSP scavenging of HF (via PF_5_/H_2_O). as demonstrated in Figure 36(a).^219^ In AF-SMB systems, they often require complementary coadditives because Na^+^ binds F^–^ more weakly.^270^ This makes FEC particularly effective when paired with FSI^–^ or TFSI^–^ based salts, where abundant fluorine sources converge to yield inorganic-rich SEIs. Second, nitrate additives such as LiNO_3_ generate Li_3_N-containing SEIs that reduce interfacial resistance and suppress dendrites; their low solubility in carbonates limits their utility outside ether systems.^217,403,404^ Bifunctional KNO_3_ also formed PF_6_^–^/NO_3_^–^-derived SEI and used K^+^ shielding as shown in Figure 36(b), yielding improved CE.^405^ In such cases, partial ether cosolvation or ionically compatible nitrate donors can recover activity without sacrificing cathode stability. Third, phosphite/phosphate additives (e.g., tris­(trimethylsilyl)­phosphite (TMSPi)) reductively yield phosphate-based SEIs with high ionic conductivity, and are particularly useful when high-voltage cathode stability must be balanced alongside anode protection.^406^ These are often deployed where FEC alone risks gas evolution or cathode impedance growth, providing a complementary inorganic network. Fourth, multifunctional additives and silicon-based interphase modifiers, including fluorinated sulfones,^407^ and Si–O systems,^408^ offer combined SEI/CEI control, HF/PF_5_/H_2_O scavenging, wettability tuning, and reduced flammability. This broadened multifunctional additives and silicon-based interphase modifiers class is exemplified by (i) nano-SiO_2_ in AF-SMBs, which boosts Na^+^ diffusion (∼×10^3^), lowers nucleation overpotential, and enables >500 stable Na|Cu cycles and superior NVP|Cu full-cell retention (Figure 36(c)),^409^ and (ii) addition of polydimethylsiloxane (PDMS) to 0.4 M KPF_6_ in 1,2-dimethoxyethane for AF-PMBs helps smooth K deposition through Si-O-mediated field effects as shown in Figure 36(d).^410^ Together, these cases illustrate how Si–O systems and multifunctional chemistries codeliver SEI/CEI control, impurity scavenging, wettability tuning, and dendrite suppression. Taken together, the choice of family depends on which failure mode is dominant (early nucleation, cathode attack, or flammability), with dosage adjusted to prevent viscosity increase or parasitic gas formation.^218,411–414^

**Figure 36:**
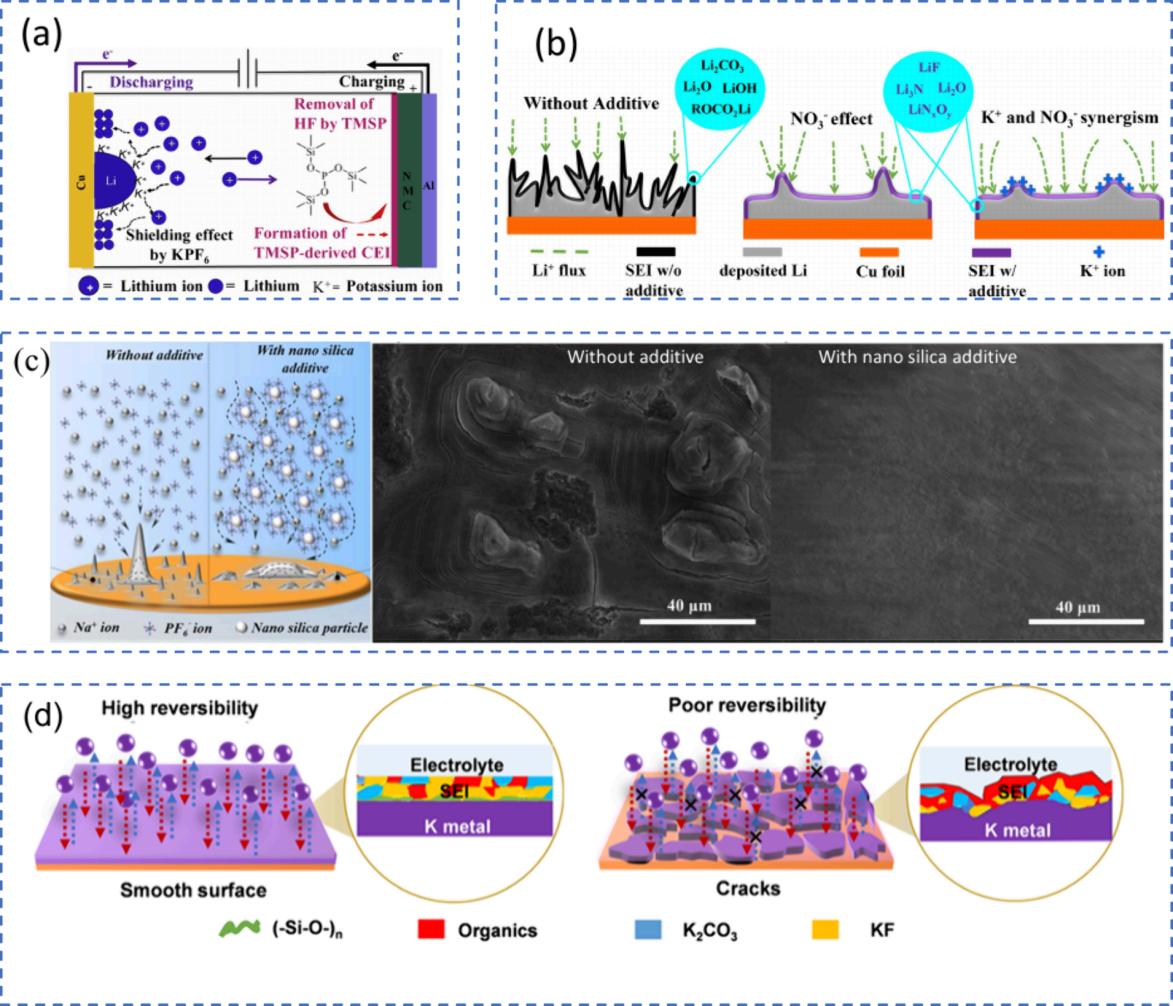
(a) Illustration of the effect of dual additives, 2 wt % KPF_6_ and 2 vol % TMSP, in 1 M LiPF_2_ in EC/DEC (1:1 vol %) electrolyte for AF-LMBs. Reproduced with permission,^219^ Copyright 2019 Elsevier. (b) Illustration of the effect of KNO_3_ additive in promoting an inorganic-rich interfacial SEI on a Cu CC for AF-LMBs. Reproduced with permission,^405^ Copyright 2019 Elsevier. (c) Illustration of depicting the Na metal electrodeposition, instantaneous nucleation and growth without an additive, and progressive nucleation and growth with an additive in AF-SMB, and their respective SEM image of Na electrodeposits on Cu from Na|Cu cell after 50 cycles. Reproduced with permission,^409^ Copyright 2024 Elsevier. (d) Schematic illustration of the SEI film derived from the 0.4 M KPF_6_-DME electrolyte in AF-PMB; with and without 2% vol. PDMS additives. Reproduced with permission,^410^ Copyright 2022 The Authors published by Springer Nature.

In aqueous electrolyte-based AFBs, where HER, narrow stability windows, and dendrite formation are key issues, additive choices often focus on water activity and Zn^2+^ (or Al^3+^/Mg^2+^) coordination. Effective additive families for AF-ZMBs include acetate/citrate salts, whose organic anions strongly adsorb onto Zn, modify nucleation, and reduce HER, for instance, ZnSO_4_ + NaAc promoting dense Zn deposition).^415^ Phosphates (e.g., KH_2_PO_4_, Na_3_PO_4_), form in situ Zn_3_(PO_4_)_2_-rich interphases that prevent corrosion and extend Zn∥Zn cycle life beyond ∼800 h.^416^ Nitrates (NaNO_3_, Zn­(NO_3_)_2_) also mitigate corrosion and can push CE beyond 99% by forming ZnO/Zn_3_N_2_ surface layers and more uniform nuclei.^417^ (iv) Halides (Cl^–^, Br^–^) facilitate 2D nucleation and produce flatter deposits, but must be used carefully to prevent chloride-induced corrosion issues.^418,419^ For example, introducing SnBr_2_ as an electrolyte additive forms an in situ Cu/Sn/Zn alloy on Cu in AF-ZMBs (Cu∥LFP), promoting dense Zn plating, as shown in Figure 37(a, b), improving CE, and enabling E/C optimization. While modified zincophilic sites mainly enhance early stage nucleation, at higher current densities, the system becomes transport-limited (Zn^2+^ depletion, resulting in rising overpotential), causing morphology degradation unless zincophilicity is combined with CC design and improves ion transport. Building on this idea, adding a trace of I_3_^–^ (10 mM) into ZnSO_4_ converts surface Cu to CuI and reconstructs porous Cu nanoclusters (CuNC), adding zincophilic sites, as shown in Figure 37(c, d).^420^ The CuNC@Cu∥Zn half-cell AFB then achieves 99.88% CE over more than 4000 cycles at 5 mA cm^–2^ and 99.91% for 7000 cycles at 20 mA cm^–2^ (1 mAh cm^–2^). Similarly, LiI/I^–^ reduces the Zn nucleation barrier through an I^–^-rich electric double layer (EDL) (Figure 37(e)), enabling highly reversible Zn∥Cu cycling and durable, Zn∥I_2_ AFBs (0.99 mAh cm^–2^; 88.2% retention after 10,000 cycles at 50 mA cm^–2^).^421^ Finally, deep-eutectic-type additives (such as urea or acetamide, well-known in Al/Mg systems) act as coligands that generate more electroactive chloroaluminate/chloromagnesium complexes and support reversible metal deposition.^422,423^ A cost-effective, nonflammable hybrid eutectic electrolyte (H_2_O/sulfolane/Zn­(OTf)_2_ + KI) maintains fully hydrated Zn^2+^ solvation, suppresses side reactions, and enables dendrite-free plating and stripping in AF-ZMBs.^424^ Notably, these aqueous additives complement the cosolvent approach in Section 6.1.3, as free-water activity decreases, adsorption and competitive coordination become more dominant, resulting in smoother morphology at lower nucleation overpotentials.

**Figure 37:**
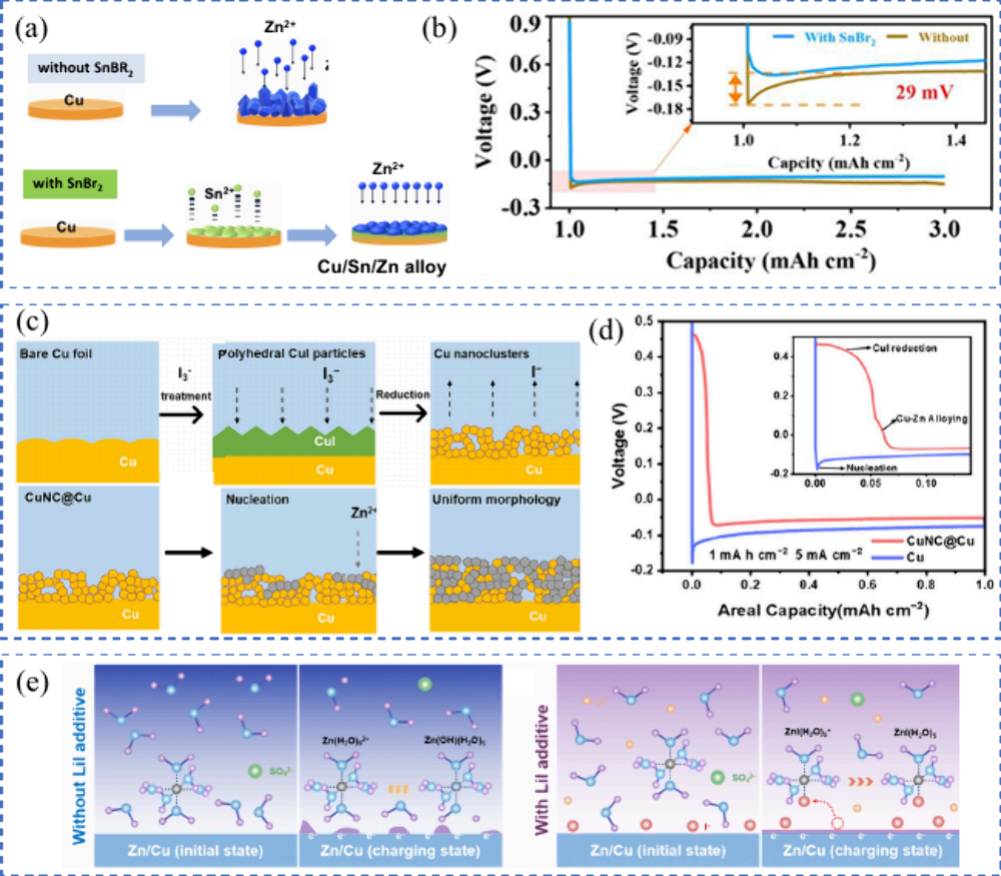
(a) Schematics depicting the working mechanisms of electrolytes without and with SnBr_2_. (b) Zn plating overpotential test using electrolyte with and without SnBr_2_ additive. (a, b) Reproduced with permission,^425^ Copyright 2023 Elsevier. (c) Illustrations showing surface morphology changes on Cu foil during reconstitution and Zn deposition on CuNC@Cu. (d) First-cycle galvanostatic deposition curves for CuNC@Cu and Cu electrodes at 5 mA cm^–2^, with an inset showing differences in the deposition process. (c, d) Reproduced with permission,^420^ Copyright 2022 Springer. (e) Schematic representation of Zn^2+^ charge transfer in electrolytes with and without LiI additive. Reproduced with permission,^421^ Copyright 2024 The Royal Society of Chemistry.

The action of interphase-directed additives can be explained through three complementary frameworks that often work together: (i) sacrificial reduction or decomposition, where additives with lower reduction potentials than the solvent break down first and influence interphase chemistry (e.g., FEC → fluorine-rich SEI);^426,427^ (ii) surface adsorption, where species like acetate or halides stick to CC or metal surfaces, change surface energy, and promote high nucleation density and even lateral growth;^428,429^ and (iii) competitive solvation, where anions such as NO_3_^–^ enter the primary solvation shell of Li^+^ or Zn^2+^, modify desolvation barriers, and shift reduction pathways toward more favorable SEI/CEI products.^430,431^ These mechanisms often work together, resulting in lower nucleation overpotential, higher and more uniform nucleus density, and less dendrite formation. Importantly, these frameworks are not mutually exclusive: one additive can adsorb, enter the solvation shell, and undergo sacrificial decomposition, so diagnostics should target all three (Raman/NMR for coordination; impedance/chronopotentiometry for nucleation overpotential; and XPS/ToF-SIMS for SEI composition). Linking additive dose to changes in CN, CIP/AGG populations, and SEI inorganic content provides a direct connection from mechanism to morphology. Overall, additives are attractive in AFBs because they (i) provide significant performance gains at low concentrations, which reduces costs, (ii) are chemically modular, enabling customized SEI/CEI structures across Li/Na/K/Zn/Al/Mg batteries, and (iii) allow rapid prototyping since small compositional changes can be tested efficiently.

However, there are several remaining challenges including (i) additive depletion over cycling that lessens benefits, (ii) solubility limits restricting formulation options (e.g., LiNO_3_ in carbonates), (iii) corrosion risks from certain halides at higher loadings, and (iv) the difficulty of achieving true multifunctionality that balances SEI requirements at the anode with CEI needs at the cathode. Future directions involve the rational design of “multi-tasking” additives (e.g., fluorinated organophosphates) that concurrently form inorganic-rich SEIs and passivating CEIs, as well as redox-shuttle concepts that prevent overcharge and help stabilize interfaces. Both of these approaches could enable high CE and stable morphology under lean-electrolyte, high-rate conditions typical of AFBs.

#### 6.1.5 Ionic Liquids and Gel Polymer Electrolytes

Ionic liquids (ILs) and gel polymer electrolytes (GPEs) have emerged as attractive alternatives to traditional organic or aqueous media for AFBs, as summarized from several reported studies in Table 6. They offer low volatility, nonflammability, broad electrochemical stability, and tunable interfacial chemistry, as shown in Figure 38. ILs, which are fully ionic fluids composed of organic cations and organic or inorganic anions, enable direct molecular design to influence solvation strength, viscosity, and redox stability directly.^441^ Meanwhile, GPEs involve a liquid electrolyte immobilized within a polymer matrix, which combines mechanical strength with high ionic conductivity. In AFBs, these properties are especially beneficial: by (i) mechanically constraining the electrode/electrolyte interface to prevent dendrite growth, (ii) directing selective decomposition toward protective SEI/CEI chemistries, (iii) ensuring stability under low-electrolyte and high-voltage conditions, and (iv) improving safety through negligible vapor pressure, ILs and GPEs provide a platform for reversible plating and stripping without a preinstalled anode. In AF-LMBs specifically, room-temperature ILs (e.g., EMIM-TFSI and related RTILs) mixed with LiTFSI/LiFSI offer nonflammability, thermal/oxidative stability, and good conductivity but are ultimately limited by higher viscosity, especially at low temperatures.^441–443^

**Figure 38:**
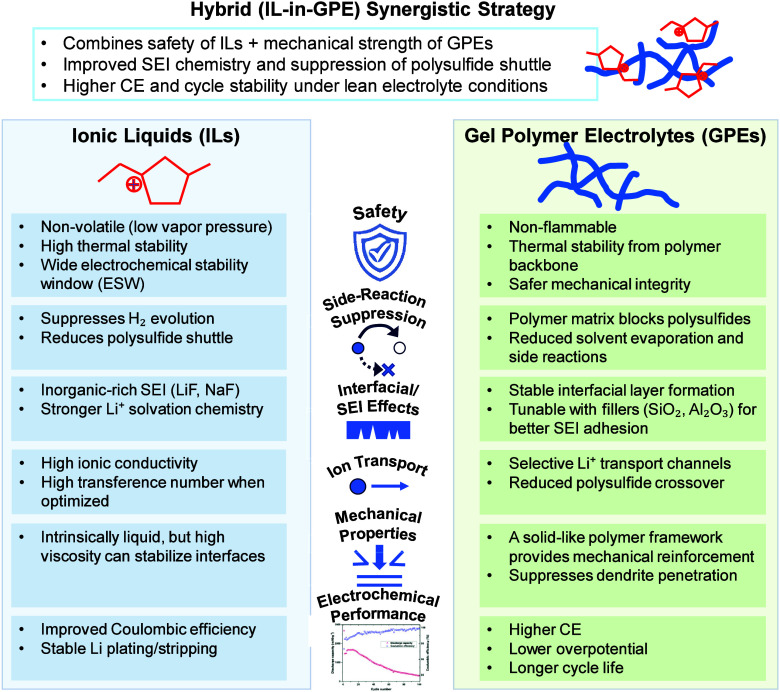
Comparative overview of ILs and GPEs for AFB applications.

**Table 6:** Performance of AFBs with Ionic Liquid Electrolytes and Gel Polymer Electrolytes

Battery type	Cell configuration	Electrolyte	Potential window (V)	Capacity (mAh cm^–2^)	Current	Cycle	CR (%)	CE (%)	Ref.
AF-LMB	Cu||NMC	[P1222][FSI]: LiFSI = 1:1 (mol/mol) IL electrolyte	1.5–4.0	3.4	0.05C	100	22.4	98.6	^448^
	Cu||LiFePO_4_	[P1222][FSI]: LiFSI = 1:1 (mol/mol) IL electrolyte	2.8–3.8	3.4	0.05C	100	33.0	98.9	^448^
	Cu||NMC111	PVDF-HFP with NASICON filler gelled using 3 M LiFSI in EC/DMC (1:1 v/v)	2.5–4.3	2.4	0.2 mA cm^–2^	80	40.2	98.3	^454^
	Cu||NMC811	PVDF-HFP + polyhedral oligomeric silsesquioxane gelled using 1 M LiPF_6_ in EC/DEC (1:1 v/v)	3–4.3	235	0.2C	14	80	-	^470^
	Cu||NMC811	PVDF-HFP-flurinated PI gelled using 1.5 M LiFSI + 0.058 M LiDFOB in DME/TTE (1:4 v/v)	2.7–4.3	2.7	0.5 mA cm^–2^	100	70	99.8	^457^
	Cu||LiFePO_4_	PVDF-HFP-PHEMA gelled using 0.5 M LiTFSI + 0.5 M LiPF_6_ with 3 wt % LiNO_3_ in DOL/DME	2.5–4	1.30	0.3 mA cm^–2^	100	62.2	-	^455^
AF-SBs	Cu||Li_2_S	PVDF-Ti_3_C_2_T_*x*_-LiTFSI composite GPE	1.7–2.8	7.32	0.2C	300	80.0	-	^461^
	Cu||Li_2_S	Thermoplastic polyurethane-SiO_2_-x(OH)x) gelled using 1 M LiTFSI) in DME/DOL (1:1 v/v)	1.7–2.7	1.4	0.1C	500	38.5	98.6	^471^
AF-ZMB	ITO@Ag@Cu||Zn_*x*_VO_2_	GPE of acrylamide, N, *N*-methylenebis(acrylamide), potassium persulfate (1000:5:8 w/w/w) + w/w/w) + 2 mL of 3 M Zn(OTF) solution	0.2–1.4	266.2 mAh g^–1^	0.5 A g^–1^	100	80		^472^
	ITO: indium tin oxide								

ILs have been extensively studied as primary solvents or cosolvents in Li, Na, and K systems, with imidazolium,^444^ pyrrolidinium,^445^ and phosphonium,^446^ cations commonly paired with TFSI^–^, FSI^–^, or BF_4_^–^ anions. Their benefits in AFBs stem from (i) broad electrochemical windows (often tolerating 5–6 V) that support high-voltage cathodes such as NMC811; (ii) inherently stable interphases because reductive transformation of TFSI^–^/FSI^–^ produces inorganic fluorides (LiF/NaF/KF) that inhibit dendritic growth; (iii) low volatility and nonflammability, which enhance safety; and (iv) high intrinsic ion density that aids transport despite viscosity potentially causing kinetic limitations.^441,447,448^ Notable results include LiFSI in P_1222_FSI achieving approximately 99.4% CE with 53% capacity retention after 100 cycles in Cu∥NMC622 AF-LMB,^448^ NaFSI in DME:TTE:C_3_ mpyrFSI (v/v 1:1.3:1:0.3) supporting dense Na deposition with about 99.1% CE after 500 cycles in Cu||Na half-cell AF-SMB at 20 °C.^449^

Mechanistically, IL solvation involves strong anion coordination; for example, in LiFSI in Pyr_13_TFSI, a large majority (>80%) of Li^+^ ions exist in FSI^–^-rich environments, which increases the desolvation barrier compared to ethers but favors SEI formation with LiF-rich, dendrite-resistant layers, leading to more uniform nucleation. In practice, this creates a key trade-off: higher desolvation barriers raise nucleation overpotential but promote dense, uniform metal growth, which is often desirable in AFBs. The choice of cation and anion also offers additional control: shorter-alkyl pyrrolidiniums generally lower viscosity than imidazoliums. At the same time, FSI^–^ enhances kinetics and yields a LiF-rich SEI, though at slightly narrower oxidative limits compared to TFSI^–^. When viscosity or low-temperature kinetics restrict performance, small organic cosolvents or multisalt configurations can be added to boost conductivity without compromising IL-driven SEI chemistry.^441–443^

ILs also provide advantages to aqueous chemistries (Zn, Al, Mg) by either partly replacing water to reduce its activity or creating IL-water hybrids that combine aqueous conductivity with IL stability. In Zn-based AFB systems, HFE is added to a Zn­(TFSI)_2_/N-butyl-N-methylpyrrolidinium IL, creating an IL-HFE electrolyte for AF-ZMBs.^450^ The HFE interacts with Pyr^+^, dispersing ion aggregates, reducing viscosity, and increasing Zn^2+^ diffusion by approximately 30 times. In half-cells, Zn∥Ti achieved 99.9% plating/stripping CE over 2000 cycles, and Zn∥Cu delivered higher accumulated capacity and smoother plating than the control (Figure 39(a)). Moreover, mixtures like Zn­(TFSI)_2_ + EMIMTFSI lower the free-water content, inhibit hydrogen evolution, and produce dense Zn deposits, with CE values exceeding 99% during hundreds of hours; ILs containing halide anions (e.g., EMIMCl) can further alter Zn^2+^ solvation to enhance deposition.^330^ In Al-based AFB systems, AlCl_3_/EMIMCl eutectics are among the most established IL electrolytes: electroactive Al_2_Cl_7_^–^ species allow reversible Al plating and stripping. At the same time, EMIM^+^ stabilizes the chloroaluminate melt, providing excellent oxidative stability and nonflammability despite some residual corrosivity.^330^ For Mg, mixtures like Mg­(TFSI)_2_ in Pyr_13_TFSI can partially desolvate Mg^2+^, enabling reversible plating with approximately 97% CE. IL-like deep eutectic solvents (e.g., MgCl_2_ + AlCl_3_) produce active Mg-Cl complexes that support deposition.^451^ Essentially, these IL-water (or eutectic) hybrids resemble LHCEs: the IL-rich environment suppresses the most reactive species (free H_2_O) and modifies the primary solvation shell, resulting in increased HER overpotentials and favoring the formation of inorganic, mechanically durable interphase products. The restructuring of the double layer, reduction in water activity, and improved anion behavior influence the overall electrochemical stability, promoting smoother morphology and higher CE.^452,453^

**Figure 39:**
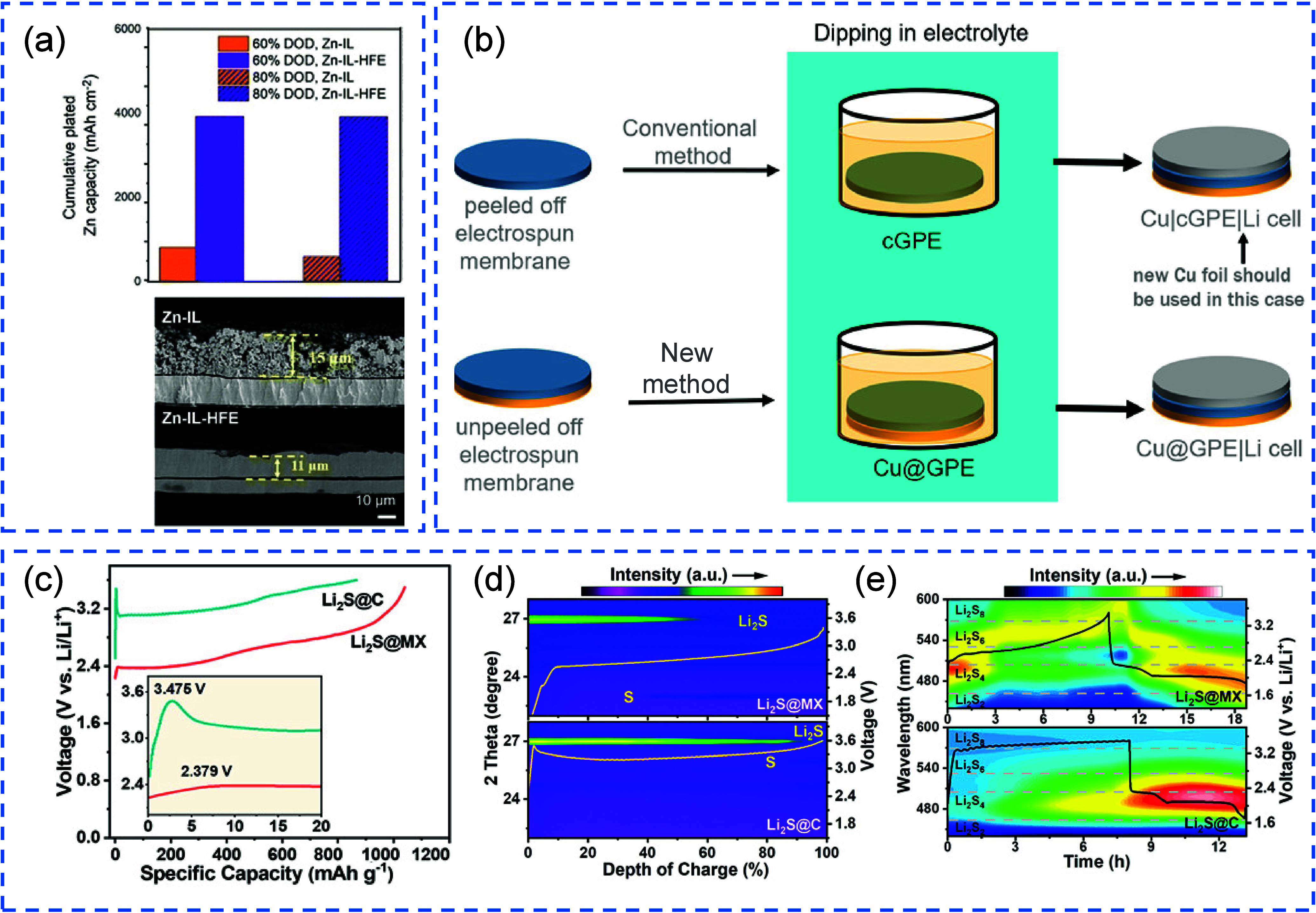
(a) Zn∥Cu capacity and cross-sectional SEM with IL-HFE vs IL. Reproduced with permission,^450^ Copyright 2023, Wiley-VCH. (b) New AFB-oriented GPE preparation vs conventional. Reproduced with permission,^454^ Copyright 2023 Elsevier. (c) Initial charge of Li_2_S@MX vs Li_2_S@C at 116.6 mA g^–1^ (inset: activation barrier). (d) Operando XRD (first charge). (e) Operando UV–vis during cycling. (c–e) Reproduced with permission,^461^ Copyright 2022 Springer Nature.

GPEs immobilize liquid electrolytes within polymer hosts, including PEO, PVDF-HFP, PAN, PMMA, and bioderived matrices such as chitosan or cellulose, thereby combining solution-like conductivity with solid-like mechanical strength. Their benefits for AFBs are well demonstrated by (i) dendrite suppression through mechanical confinement that prevents crack growth and filament penetration, (ii) improved safety due to reduced leakage and negligible volatility, (iii) better interfacial wetting that lowers nucleation overpotential on metal-free CCs, and (iv) broad applicability to both organic (Li, Na) and aqueous (Zn) systems. For AF-LMBs in particular, GPEs are valued for minimizing leakage/flammability while maintaining near-liquid-like conductivity and providing mechanical suppression of dendrites, making them strong candidates for stable Li plating on bare CCs.^454–459^ For example, our research group developed a new GPE preparation method suitable for AFBs (Figure 39(b)),^454^ and the resulting PVDF-HFP-LiFSI GPE achieved approximately 99% CE over about 80 cycles at 0.5 mA cm^–2^ in Cu||NMC11 AF-LMBs.^454,460^ In AF-SB also, specifically, a GPE paired with Li_2_S@MX (Ti_3_C_2_) cathodes accelerates Li_2_S activation (−3.93 eV), yielding faster conversion and lower residual polysulfides than Li_2_S@C (Figure 39(c–e)).^461^

The fundamental chemistry can be explained by (i) stress-electrochemistry coupling, where the polymer scaffold applies interfacial pressure that reduces stress-driven dendrite growth;^462,463^ (ii) confinement of solvation structure within nanoscale pores, which changes local coordination and reaction pathways;^464,465^ and (iii) selective transport through functionalized backbones (e.g., sulfonated PEO) that increase cation transference numbers and promote uniform ion flux during plating and stripping.^466,467^ Compared to pure ILs, GPEs separate mechanics from chemistry: polymer crystallinity, cross-link density, and filler content (such as SiO_2_, LLZO) adjust modulus and ion pathways independently of solvent type. Plasticizers (EC/PC, fluorinated ethers) and ionogels (polymer-infiltrated ILs) expand the design possibilities, allowing operation below ambient temperature while maintaining interphase selectivity.^468,469^ Generally, aiming for a shear modulus roughly equal to or greater than the stress level for filament growth improves stability, while still allowing enough segmental motion to prevent interfacial transport limitations.

### 6.2 Current Collector and Interface Design

In AFBs, the CC functions as a “virtual anode,” guiding where and how metal nucleates and grows during operation. Unlike traditional foil anodes, this inert substrate must simultaneously offer low electronic resistance, controlled nucleation, interfacial stability, and morphology regulation, all while avoiding parasitic reactions. When these roles are not properly engineered, uneven fluxes of Li^+^/Na^+^/K^+^/Zn^2+^/Mg^2+^/Al^3+^ and local current hotspots cause filamentary growth, dead metal, and reduced CE. This section discusses three main strategies: (i) metallophilic/ionophilic surface modification, (ii) artificial interphases and protective coatings, and (iii) three-dimensional (3D) host collectors, which are applicable across organic and aqueous chemistries.

#### 6.2.1 Alloying Current Collectors

Nucleation on pristine Cu, Ni, or Al is often energetically unfavorable due to poor wettability and surface energy mismatch, resulting in high nucleation overpotential and filament initiation.^84,473^ Adding metallophilic (i.e., Li-, Na-, K-, Zn-, Mg-, Al-affine) or ionophilic (aqueous-ion compatible) sites lowers the barrier and spreads out the interfacial field. Extensive studies using this approach are reported, with some summarized in Table 7, to address the nucleation issue in AFB. In alkali systems, Ag and Au seeds form temporary alloys (e.g., Li-Ag, Na-Au) that decrease nucleation overpotential and promote compact growth.^474,475^ At the same time, oxides/halides such as ZnO, SnO_2_, TiO_2_, SrF_2_, and AlF_3_ provide lithiophilicity through favorable Li^+^ binding and help stabilize the interphase (Figure 40).^476,477^ In aqueous Zn, hydrophilic and functional coatings, polydopamine, MnO_2_, and conductive polymers (Polyaniline, PEDOT:PSS), increase Zn^2+^ affinity and suppress hydrogen evolution at the same time; for Mg/Al, low-melting seeds (Bi, In, Ga, Sn) reduce the nucleation barrier for divalent/trivalent deposition, and thin, stable oxide layers lessen self-passivation.^330,478^ Mechanistically, performance improves due to (i) more negative adsorption free energy (ΔG_ads_) for incoming cations, which increases nucleus density, (ii) field homogenization by polar groups (-O, -F, -N, -S) that prefer to adsorb solvated ions, and (iii) controlled alloying or chelation (e.g., Li-Ag, Zn^2+^-catechol in polydopamine) that speeds up lateral growth instead of vertical filaments.^476,479^

**Figure 40:**
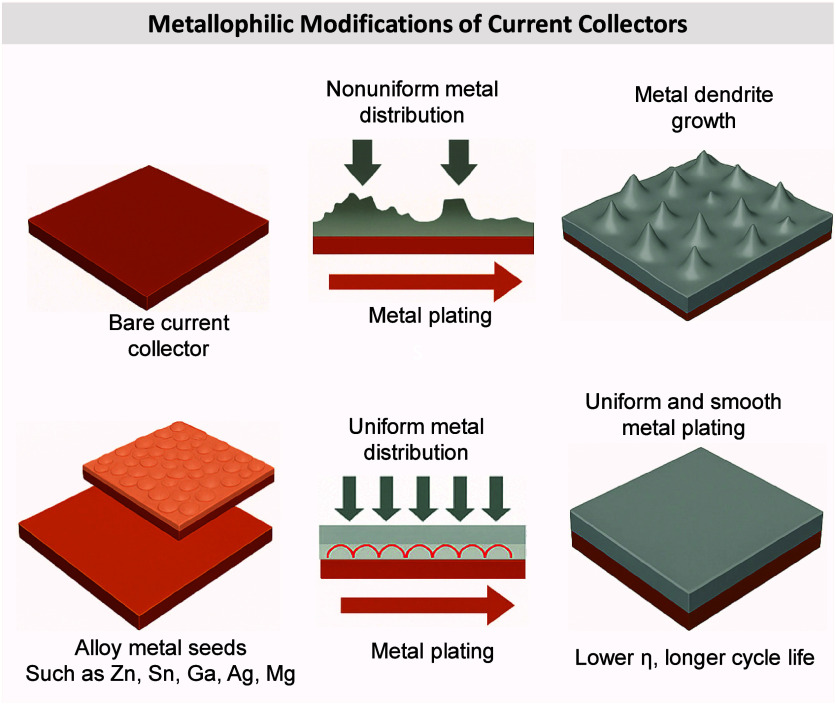
Schematic illustration of metallophilic seeding of CCs for uniform, smooth metal plating, in AFBs.

**Table 7:** Performance of AFBs with Alloy-Based Metallophilic Modification of CCs

Battery type	Cell configuration	Electrolyte	Potential window (V)	Capacity (mAh cm^–2^)	Current	Cycle	CR (%)	CE (%)	Ref.
AF-LMB	PdTe_2_–Cu||LiFePO_4_	1 M LiTFSI in DOL/DME + 2% LiNO_3_	3.0–3.8	0.74	0.2C	100	70.7	99.65	^83^
	HCu||NMC532	6 M LiFSI in DME	3.0–4.35	3	0.5C	100	41	99.11	^504^
AF-SBs	Au@Cu||Li_2_S	1 M LiTFSI) in DME/DOL (1/1, v/v) + 0.75 M LiNO_3_	1.7–2.8	0.1	0.05C	150	52.4	-	^484^
	Ag@Cu||Li_2_S	1 M LiTFSI) in DME/DOL (1/1, v/v) + 1 wt % LiNO_3_	1.7–2.8	2.85	0.1C	180	56.4	97.0	^485^
AF-SMB	Cu_2_Sb@Cu||Na_3_V_2_(PO_4_)_3_	0.9 M NaPF_6_+0.1 M NaBF_4_ in diglyme	2.0–3.8	0.76	300 mA g^–1^	600	74.2	-	^492^
	Cu@Bi||Na_3_V_2_(PO_4_)_3_	NaPF_6_ in DME	2.5–3.8	0.2	1C	80	92.4	99.2	^505^
	Zn@Ag||Prussian blue	1 M NaPF_6_ in diglyme	2.4–3.7	1.26	100 mA g^–1^	100	97	99.5	^506^
	NbMoTaWV@Al/Na||Na_3_V_2_(PO_4_)_3_	1 M NaPF_6_ DME	2.4–3.8	0.19	10C	1200	98	99.8	^507^
	GaInSn@CuNa_3_V_2_(PO_4_)_3_	1 M NaPF_6_ in diglyme+ HMDSO	2.0–3.8	1.0	200 mA g^–1^	200	87.6	99	^508^
	SnCu@Al||Na_3_V_2_(PO_4_)_3_	1 M NaPF_6_ in diglyme	2.5–3.6	0.71	1C	200	90%	99.79%	^509^
	In@Cu||Na_4_Fe_2.91_(PO_4_)_2_(P_2_O_7_)	1.0 M NaPF_6_ in DEGDME	1.5–3.9	1.44	0.5C	200	83.1	90.87	^510^
	Sn@Al||Na_3_V_2_(PO_4_)_3_	1 M NaPF_6_ in diglyme	2.4–3.8	1.2	0.5C	100	81	82.19	^511^
AF-ZMB	Cu-Sn@SSM||NVO	2 M ZnSO_4_ in H_2_O	0.2–1.6	0.49	2 A g^–1^	1000	84	-	^512^
	Sn@Na–Ti_3_C_2_T_*x*_@Cu||LMO	2 M ZnSO_4_ and 1 M Li_2_SO_4_ in H_2_O	1.4–2.1	20	0.1 A g^–1^	100	73.97	99.31	^513^
	NC-Cu@Ti||MnO_2_	2 M ZnSO_4_ in H_2_O	0.2–1.6	0.15	1 mA cm ^–2^	200	-	99.3	^514^
	NC: nanocrystalline								
AF-PMB	Cu_6_Sn_5_@Cu||KPTCDA	4 M KFSI in DME	1.5–3.5	0.77	20 mA g^–1^	30	69.4	-	^515^
AF-AMB	Au@SUS||Graphene	1.5 M AlCl_3_ with 1 M of [EMIm]Cl in H_2_O	0.4–2.45	0.28	1.4 mA cm^–2^	1000	80	98	^516^
	Au@Ti||Graphite	1.3 M AlCl_3_ with 1 M of [EMIm]Cl in H_2_O	0.4–2.45	0.3	0.2 A g^–1^	900	80	-	^517^

The main trade-offs are that (i) highly reactive seeds can cause side reactions (like Ag dissolution or displacement), (ii) oxide/fluoride layers may add interfacial resistance if they become too thick, and (iii) environment-specific stability (acidic aqueous versus basic/organic) must be matched to prevent coating degradation. Beyond these general points, alloy-forming metallophilic layers provide a direct, measurable way to lower nucleation overpotential by offering Li-M (or Na-M, Zn-M, Mg-M) phases with low interfacial energy against the depositing metal, thereby increasing nucleus density and promoting lateral growth.^35,480,481^

In AF-LMBs, lithiophilic alloy coatings based on Ag, Zn, Al, In, Sn, Bi, and Mg form Li-M alloys and have repeatedly reduced nucleation barriers and dendrite propensity. Our research group demonstrated Ag@PDA-GO as an artificial lithiophilic layer (Figure 41(a)),^482^ which regulates Li^+^ flux and suppresses dendrite growth. We also realized SrF_2_@Cu (Figure 41(b)),^483^ and a dual-coated Cu-Sn@SrF_2_@SFPH electrode that combines LiF-rich SEI formation with Sr-Li/Sn-Li alloy nucleation to stabilize plating synergistically. Additionally, an epitaxially induced plating collector (GaInSn@Cu) employing a liquid Ga-In-Sn alloy (68.5:31.5:10) lowers the Li deposition overpotential (Li deposition potential ≈0.75 V vs Li^+^/Li), promotes LiF-rich SEI, and improves Li^+^ surface diffusion and charge transfer, resulting in higher initial and overall CE compared to bare Cu (Figure 41(c)).^223^

**Figure 41:**
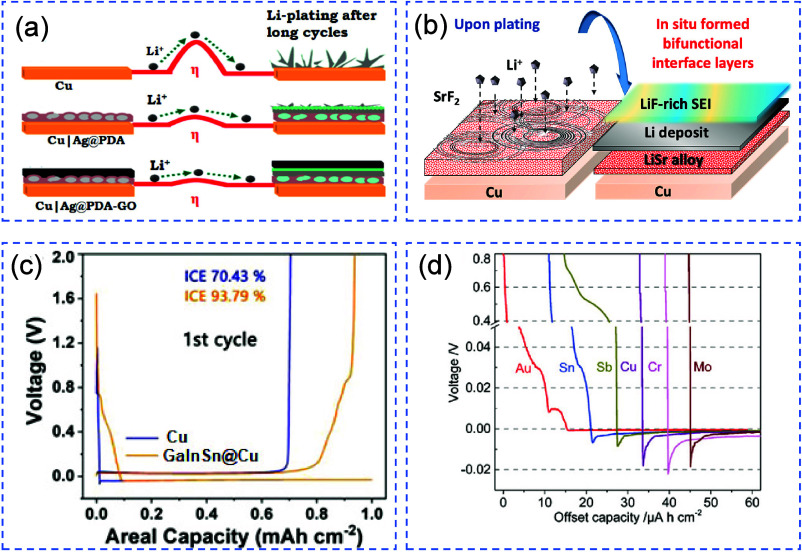
(a) Illustration of the effect of lithiophilic Ag@PDA@Cu serving as a nucleation seed after long-term cycling in AF-LMBs. Reproduced with permission,^482^ Copyright 2020 Elsevier. (b) Coating of Cu using SrF_2_ as a bifunctional for stable SEI in AF-LMBs. Reproduced with permission,^483^ Copyright 2022 Elsevier. (c) Voltage profiles of Cu||Li and GaInSnCu||Li cells during cycling at a capacity of 1 mAh cm^–2^. Reproduced with permission,^223^ Copyright 2021 Wiley-VCH GmbH. (d) Nucleation overpotentials measured for various anode CCs. Reproduced with permission,^487^ Copyright 2019 Wiley-VCH.

Cost-sensitive metallophilic alloys are equally effective in AF-SBs: an ∼80 nm Au layer on Cu transforms a lithiophobic surface into lithiophilic, reducing nucleation barriers and increasing initial discharge capacity to 770 mAh g^–1^ with 53% retention after 150 cycles in Au@Cu||Li_2_S AF-SB using the electrolyte 1 M LiTFSI + 0.75 M LiNO_3_ (DOL:DME 1:1).^484^ Additionally, a Cu sponge mesh with a ∼100 nm Ag coating decreases local current density while lowering the nucleation overpotential, enabling an initial CE of 70.7%, at 0.1C and ultrahigh S loading (14.6 mg cm^–2^ of areal loading and 7.4 mAh cm^–2^ of areal capacity) in an AFB system.^485^ These examples highlight a general principle: pairing metallophilic coatings with topology (2D→3D) amplifies benefits by aligning area dilution and nucleation control.

Sodium benefits from similar sodiophilic metals such as Sn, Bi, Sb, Au, Ag, and Zn.^486–493^ Ultrathin Au on Cu forms Na-Au alloy during plating, which eliminates measurable nucleation overpotential in some cases and promotes dense nuclei; Mao et al. observed reduced nucleation potential and improved nucleation uniformity on Au-modified Cu. Meanwhile, Tang et al. compared Cu-Mo, Cu-Cr, Cu, Cu-Sb, Cu-Sn, and Cu-Au, finding approximately 0 mV nucleation overpotential for Au and only about 5 mV for Sb/Sn, versus much higher values for Mo/Cr/Cu (Figure 41(d)).^487^ Binder-immobilized Sn nanoparticles and Sb microparticles prevent uncontrolled interlayer alloying while offering long-life reversibility in an asymmetric cell with Na foil (Cu@Sn-NP: 2000 cycles at 2 mA cm^–2^, CE ≈ 99.9%; Cu@Sb-MP: 1700 cycles at 1 mA cm^–2^, CE ≈ 99.9%), highlighting practical sodiophilic pathways that avoid noble metals.

In AF-ZMBs, zincophilic alloying and heterostructures improve field uniformity and promote low-energy nucleation.^494^ A two-dimensional Sb/Cu heterointerface that forms Cu_2_Sb on Cu (Sb@Cu) stabilizes Zn plating and stripping, achieving an average CE of 98.3% at 50 mA cm^–2^ for about 220 h (Figure 42(a)).^495^ Overall, these zincophilic designs utilize both nucleation-energy reduction and electric-field leveling by increasing contact areas and providing abundant charge centers, thus decreasing dendrite formation and balancing Zn^2+^ flux across the interface.^496–499^

**Figure 42:**
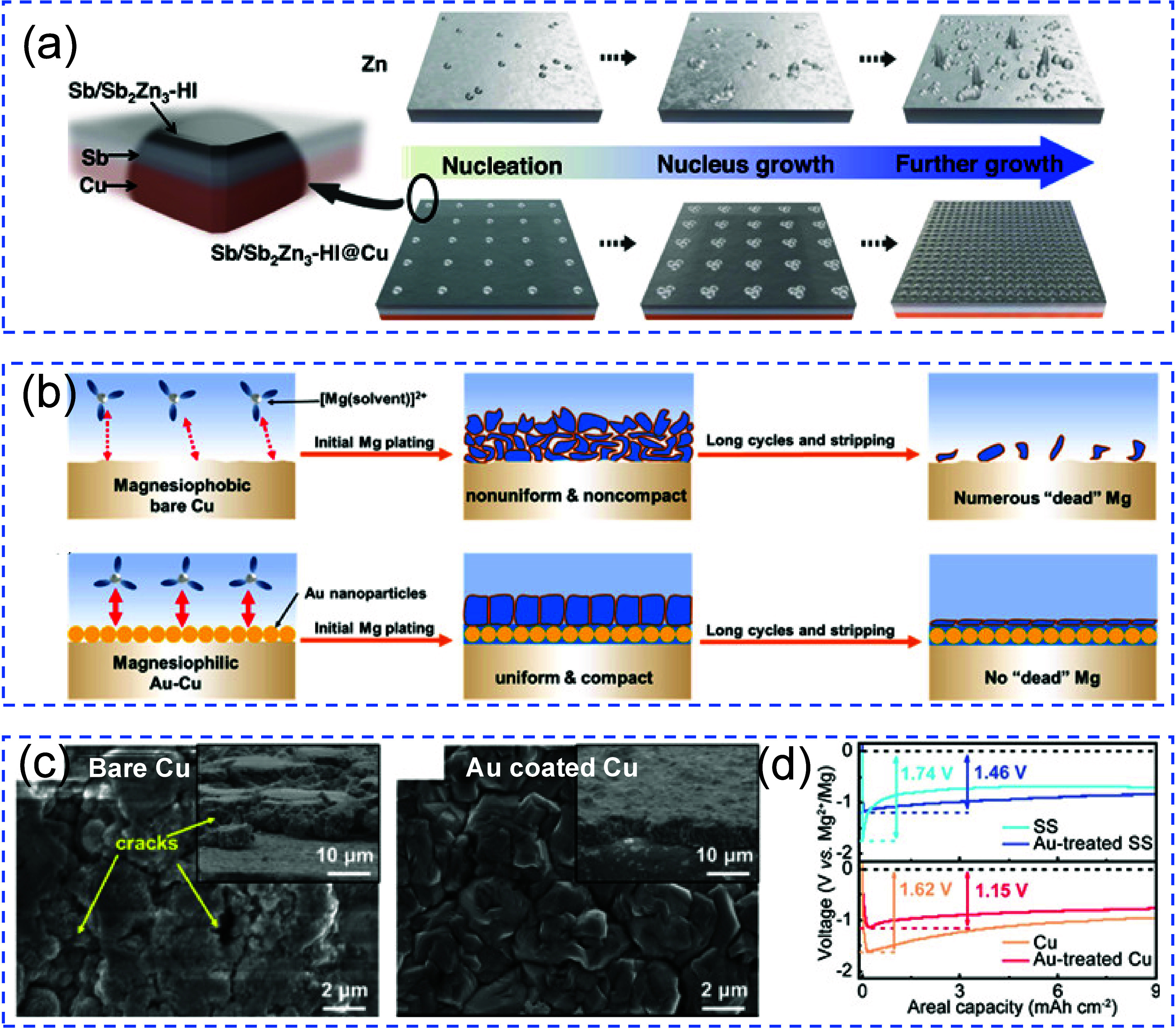
(a) Schematics of Zn electrodeposition on Zn and Sb/Sb_2_Zn_3_-HI@Cu surfaces. Reproduced with permission,^495^ Copyright 2023 Springer Nature. (b) schematic illustration of Mg plating/stripping behavior on bare Cu and Au-coated Cu electrodes, (c) SEM images of Mg plating on bare Cu and Au-coated Cu foils. (b, c) Reproduced with permission,^500^ Copyright 2021 American Chemical Society. (d) Comparisons of electrochemical profiles of Mg depositions on different substrates. Reproduced with permission,^501^ Copyright 2022 American Chemical Society.

For Mg, which passivates easily, adding a thin, conformal Au layer on Cu using ion-beam sputtering yields smooth, compact Mg deposits, unlike the cracked films observed on bare Cu (Figure 42(b, c)).^500^ Diamond-shaped Au nanoseeds sputtered onto stainless-steel (SS) and Cu further reduce nucleation overpotential (∼0.57 → 0.43 V at 10 μA cm^–2^) and remain effective up to 10 mA cm^–2^ (Figure 42(d)).^501^ A modified carbon cloth coated with α-/β-Ni­(OH)_2_ reduces the nucleation overpotential and enhances cycling by offering strong binding sites for Mg atoms formed after Mg^2+^ reduction at the interface. Favorable lattice matching and the structured capture sites of the cloth direct epitaxial, nondendritic growth of Mg, while the coating also regulates the influx of Mg^2+^ from the electrolyte.^502,503^ These findings indicate that oxide-free nucleants (e.g., Au), hydroxide terminations, and lattice-matched surfaces can collectively steer Mg deposition toward lateral growth even under high utilization.

Emerging strategies also utilize metallophilicity and alloying at the collector: in an anode-free aluminum metal battery (AF-AMB) proof-of-concept, a (polyaniline-coated graphene/Al_2_TiO_5_ cathode (PANI@G-Al_2_TiO_5_) consistently replenished Al during cycling while forming a uniform Al-Cu alloy layer on Cu, achieving 175 mAh g^–1^ initially and approximately 410 Wh L^–1^ power density with about 60% capacity retention after 1000 cycles.^503^ This pairing of alloyable collectors with prealuminum sources demonstrates how “virtual anode” alloy chemistries can stabilize trivalent deposition.

For practical considerations, noble-metal coatings (Au/Ag) significantly improve nucleation but might add cost and weight, which can diminish the advantages of anode-free designs. Therefore, prioritizing earth-abundant metallophiles (Sn, Sb, Zn, Al, Mg) or ultrathin noble layers is crucial for scaling.^84,484–493^ A streamlined workflow includes: (1) identifying the dominant cation and electrolyte anion (FSI^–^/TFSI^–^/PF_6_^–^/Cl^–^) to align alloying and SEI chemistry; (2) selecting a metallophilic seed (preferably alloy-forming) to reduce nucleation overpotential on interior surfaces; (3) incorporating ionophilic or dielectric features (such as LiF@PVDF) when field homogenization is limited; (4) validating performance under target current, electrolyte, and speed through nucleation overpotential, CE, operando imaging, and XPS/ToF-SIMS analysis; and (5) adjusting thickness and composition to prevent excessive resistance and cost. When applied to Li, Na, Zn, Mg, or Al systems, the common goal remains the same: creating dense, laterally propagating nuclei seeded by metallophilic and ionophilic motifs that are compatible with the electrolyte’s SEI/CEI precursors, thereby stabilizing CE during lean-electrolyte, high-rate operation.

#### 6.2.2 Artificial Interphases and Protective Coatings

Because native SEIs on bare collectors are compositionally uncontrolled in AFBs, pre-engineered interphases can set ion pathways and mechanics before the first cycle.^483,518–520^ As shown in Figure 43, thin polymeric films (PEO, PAN, PVDF, cross-linked networks) provide elasticity and facilitate ion conduction; ALD-grown ceramics/oxides (Al_2_O_3_, TiO_2_, ZrO_2_, Li_3_PO_4_) offer high permittivity and uniform fields; 2D carbons (graphene oxide, reduced GO, MXenes, carbon nitride) deliver electronic passivation with tunable ionic permeability; and polymer-ceramic hybrids (e.g., PEO-Al_2_O_3_, PVDF-SiO_2_) optimize compliance and modulus.^521–524^ Their effectiveness comes from (i) ion-selective transport through nanochannels or polar groups that favor Li^+^/Na^+^/Zn^2+^ over anions/solvent, (ii) a barrier function that separates the metal or CC from corrosive or HER-active species, and (iii) mechanical suppression where a sufficiently high shear modulus resists protrusions.^75,150,525^ In practice, ALD-Al_2_O_3_ can decrease nucleation overpotential and achieve CE > 99% in Li/Na/K AFBs; GO-like films on Zn reduce dendrites and HER; and polymer–ceramic laminates stabilize Mg/Al ion transport. The design window is narrow: (i) too thin risks pinholes and early failure; (ii) too thick increases ohmic and charge-transfer resistance; (iii) brittle films crack under cycling strain; and (iv) the scalability and cost of ALD or MXene processing must be balanced with manufacturing needs. Several studies, summarized in Table 8, have used artificial SEI and protective strategy approaches and achieved improved AFB performance.

**Figure 43:**
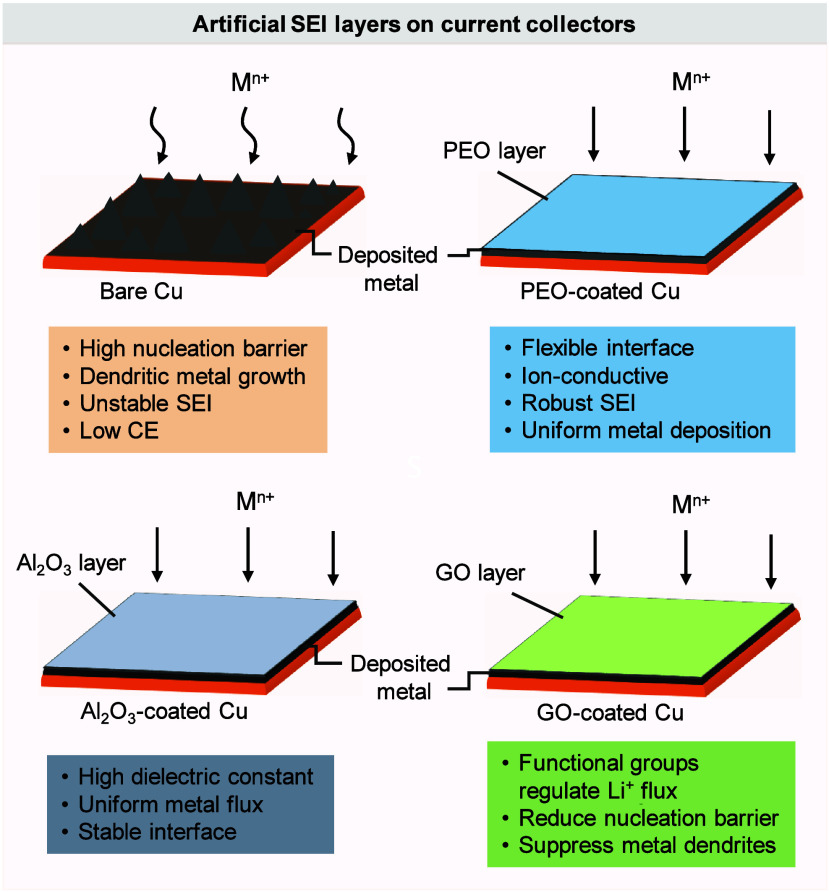
Demonstration of artificial SEI coatings, polymers, oxides, and graphene oxides on CCS to control metal-ion flux and build durable ion-conductive interphases.

**Table 8:** Performance of AFBs with Artificial Interphase-Forming Protective Coatings on the CC

Battery type	Cell configuration	Electrolyte	Potential window (V)	Capacity (mAh cm^–2^)	Current	Cycle	CR (%)	CE (%)	Ref.
AF-LMB	Cu@PEO||LiFePO_4_	1 M LiTFSI in DOL/DME (1:1 v/v) + 2% LiNO_3_	3–3.8	0.711	0.2C	100	50	98.6	^404^
	Cu@LiF-PVDF||LiFePO_4_	1 M LiTFSI in DOL/DME (1:1 v/v) + 3% LiNO_3_	2.5–3.8	0.68	0.5C	80	44	97.6	^528^
	Cu@SiO_*x*_ ||LiFePO_4_	4 M LiFSI in DME	3–3.8	2.0	0.9 mA cm^–2^	100	93.5	99.9	^551^
	Ag-PCP@Cu|| LFP	3 M LiTFSI in DME/DOL) (1:1, v/v)	2.5–4.2	2.21	1C	200	72	-	^87^
AF-SBs	BP@Cu||Li_2_S	1 M LiTFSI) in DME/DOL (1/1, v/v) + 5 wt % LiNO_3_	1.7–2.8	3.10	0.2C	100	64.8	-	^531^
AF-SMB	Ti_3_C_2_T_*x*_/CNT NAFs||Na_3_V_2_(PO_4_)_3_@C	NaPF_6_ + NaBF_4_ in DME	2.5–4	1.07	2C	1000	69.4	-	^552^
	HCOONa@Cu||Na_3_V_2_(PO_4_)_3_	NaPF_6_ in diglyme	2.8–3.7	0.98	0.5C	400	88.2	99.9	^553^
	fluorinated Al||Na_3_V_2_(PO_4_)_3_	Na[FSA]-[C2C1im][FSA] ([FSA]^−^ = bis(fluorosulfonyl)amide anion and [C2C1im]^+^ = 1-ethyl-3-methylimidazolium cation) ionic liquid	2.4–3.6	0.91	0.3C	50	46.1	98	^554^
	RPC@Al||Na_3_V_2_(PO_4_)_3_	1 M NaPF_6_ in diglyme	2.0–3.8	1.24	1C	500	92.4	-	^555^
	C@Al||Na_3_V_2_(PO_4_)_3_	1 M NaPF_6_ in diglyme	2.6–3.6	4.2	0.5C	100	93.0	99	^224^
	Ketjenblack@Cu||Na_3_V_2_(PO_4_)_3_	1 M NaPF_6_ in diglyme	2.5–3.8	1.0	0.5C	150	94.2	99.8	^556^
	plasma-treated carbon-coated Al ||PTPAn	1 M NaPF_6_ in diglyme	2.2–4.1	1.0	2C	500	56.2	-	^349^
	Ru/NC-Cu||Na_3_V_2_(PO_4_)_3_	1.0 M NaPF_6_ in DEGDME	2–3.6	0.3	0.5C	100	98.1	90.75	^279^
	ZnIn_2_S_4_/CNT@Cu||Na_3_V_2_(PO_4_)_3_	1 M NaPF_6_ DME	2.4–3.6	0.17	0.2C	170	99	99.9	^557^
AF-ZMB	Cu@PMMA:Zn||ZnMnO_2_	3 M Zn(CF_3_SO_3_)_2_ and 0.1 M Mn(CF_3_SO_3_)_2_ in H_2_O	0.8–1.8	1.0	1 A g^–1^	300	80	∼100	^558^
	graphene@ Al||K-FeS_2_	4 M KFSI in DME		0.33	0.1 A g^–1^	30	∼30	-	^541^
	C/Cu||Zn-MnO_2_	3 M Zn(CF_3_SO_3_)_2_ and 0.1 M Mn(CF_3_SO_3_)_2_ in H_2_O	0.8–1.8	3.0	1 mA cm^–2^	80	68.2	-	^545^
	Cu@AOF||Zn_0.5_VO_2_	2 M ZnSO_4_ in H_2_O	0.2–1.6	0.47	1 A g^–1^	300	80	99.97	^543^
AF-AMB	Au@Ti||graphite	AlCl_3_/[EMIM]Cl ionic liquid electrolyte	0.4–2.4	0.5	200 mA g^–1^	900	80	-	^517^
	Au@SUS||graphine	AlCl_3_/[EMIM]Cl ionic liquid electrolyte	0.4–2.4	0.3	1.4 mA cm^–2^	1000	80	∼98	^516^

Lithiophilic artificial SEI/coating strategies deliberately lower the Li nucleation barrier and surface diffusion energy to promote planar growth. Examples include PEO coatings that even out Li^+^ flux and decrease nucleation overpotential (Figure 44(a)),^404^ ultrathin GO on Cu that stabilizes the interface (Figure 44(b)),^413^ and Al_2_O_3_/PAN composite layers (AOP) that support uniform subfilm deposition.^526^ Multicomponent interphases can both conduct ions and act as a Li reservoir: a branched-PEI/Ag NP/LiNO_3_ stack (BPEI-Ag/LiNO_3_@Cu) forms a Li_3_N-rich, ion-conductive SEI, while Ag-Li alloy buffers plating, enabling high areal capacity (4.2 mAh cm^–2^) with 61.6%/52% retention after 50/100 cycles in carbonate electrolytes.^527^ High-dielectric, ionophilic coatings can further homogenize metal plating and decrease nucleation overpotential.^527,528^ A LiF-nanoparticle-PVDF composite film biases PVDF toward its high-polarity β-phase, smoothing interfacial fields and reducing nucleation and plateau overpotentials; the approximately 2.5 μm LiF@PVDF layer enables uniform, dendrite-free Li growth and strong full-cell performance in LiF@PVDF||LFP (Figure 44(b)).^528^ Moreover, a silver nanoparticles incorporated p-doped conjugated polymer (Ag-PCP) wetting agent on the copper CC (Ag-PCP@Cu) promotes uniform Li nucleation and plating.^87^ An AF-LMB configured as Ag-PCP@Cu||LFP demonstrated a high capacity retention of 72% and an average CE of 99.8% at 1C-rate after 200 cycles. These dielectric-enhanced layers demonstrate how ionophilicity and field homogenization couple with metallophilicity to influence both nucleation thermodynamics and early SEI chemistry.^209,529,530^ Nonmetallic, chemoselective coatings can also shape SEI chemistry in Li–S AFBs: black phosphorus (BP) on Cu enhances lithiophilicity and reacts with polysulfides to form Li_7_PS_6_ within the SEI, improving Li^+^ conductivity and enabling reversible plating/stripping.^531^ Practical deployment will require addressing BP’s stability, polysulfide migration, scalability, and cost challenges.

**Figure 44:**
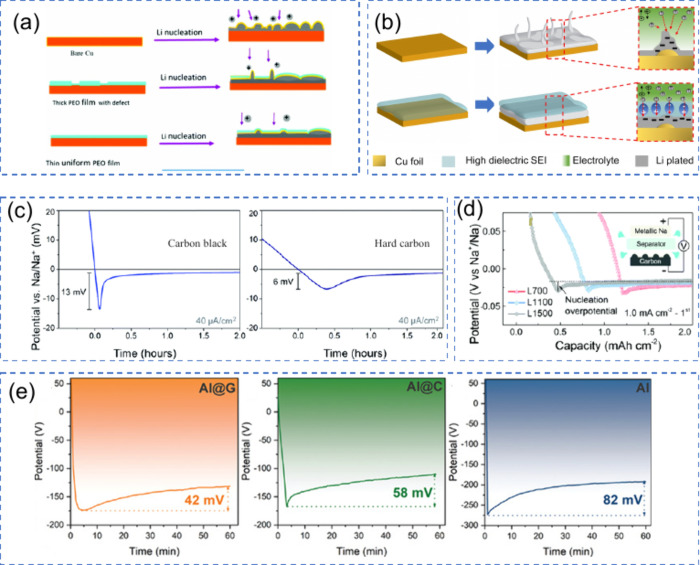
(a) Schematic of Li plating on a bare Cu and PEO-coated Cu CCs in AF-LMBs. Reproduced with permission,^404^ Copyright 2018 The Royal Society of Chemistry. (b) Schematic of Li-ion depletion under concentration polarization: top, no high dielectric-constant artificial SEI; bottom, high dielectric-constant artificial SEI reducing surface concentration gradients. Reproduced with permission,^528^ Copyright 2021 American Chemical Society. (c) Comparison of nucleation overpotentials for carbon black and hard carbon CCs in AF-SMBs. Reproduced with permission,^532^ Copyright 2018 The Royal Society of Chemistry. (d) Nucleation overpotentials observed during sodium deposition on L700, L1100, and L1500 substrates. Reproduced with permission,^533^ Copyright 2021 The Royal Society of Chemistry. (e) First cycle overpotentials of three Al-based CCs in AF-PMBs. Reproduced with permission,^541^ Copyright 2019 John Wiley and Sons.

For Na in AF-SM B systems, carbon interphases influence nucleation through surface area, defects, and heteroatom chemistry: nongraphitized carbons display different behaviors, hard carbon shows a lower nucleation overpotential (∼6 mV) than carbon black (∼13 mV) (Figure 44(c)),^532^ while defect-rich, porous carbons (e.g., L700) decrease nucleation overpotential and stabilize cycling compared to more-graphitized frameworks (L1500) (Figure 44(d)).^533^ Sodiophilicity can be tailored by doping or functionalization: N-doped carbon (including plasma-treated Al∥N-carbon) supports uniform Na nucleation and growth,^395^ and heteroatoms or functional groups (N, O, S; -NH) create active sites that reduce nucleation barriers and enhance morphology.^534–536^

For AF-PMB systems, potassiophilic coatings increase surface energy and improve wetting: reduced graphene oxide (rGO) on Cu enables complete wetting of molten K within approximately 6 s, whereas no infusion occurs on bare Cu.^537–539^ High-defect carbons (e.g., those with NiO particles) and N-doped motifs further reduce plating overpotential.^361,540^ Roll-to-roll PECVD graphene on Al (Al@G) minimizes K inventory loss, adheres strongly, and maintains smooth K plating, resulting in stable plating and stripping at 4.0 mA cm^–2^ (4.0 mAh cm^–2^) and lasting up to 1000 h at 0.5 mA cm^–2^. Durability remains at 0.1–2.0 mA cm^–2^, with a stable SEI supporting high CE (Figure 44(e).^541^

For AF-ZMBs, interphases must both conduct Zn^2+^ and block side reactions. A carbon nanodisc treated on a Cu foil CC (C/Cu) promotes uniform Zn nucleation and plating. In an AF-ZMB, configured as C/Cu||Zn-MnO_2_, it offered 200 mAh g^–1^ at 1 mA cm^–2^ and retains 68.2% of its initial capacity after 80 cycles.^542^ On the other hand, aluminum hydroxide fluoride-coated Cu foils (AOF@Cu), as shown in Figure 45(a), demonstrate a high affinity for water and a low diffusion energy barrier for Zn adatoms. This promotes faster desolvation and regulates Zn^2^ flux.^543^ AOF@Cu||Zn_0.5_VO_2_ AF-ZMB shows a capacity of 203 mAh g^–1^ and retains 80% of its capacity after 300 cycles at 1 A g^–1^, with an ultrahigh average CE of >99.9%. Strong adhesion and proper zincophilicity at the CC|interphase|Zn interfaces are essential:^544,545^ Weak bonding can cause interphase delamination, contact loss, local current spikes, and dendrites; in contrast, zincophilic, well-adhered interfaces lower the nucleation barrier and promote dense, lateral Zn growth.

**Figure 45:**
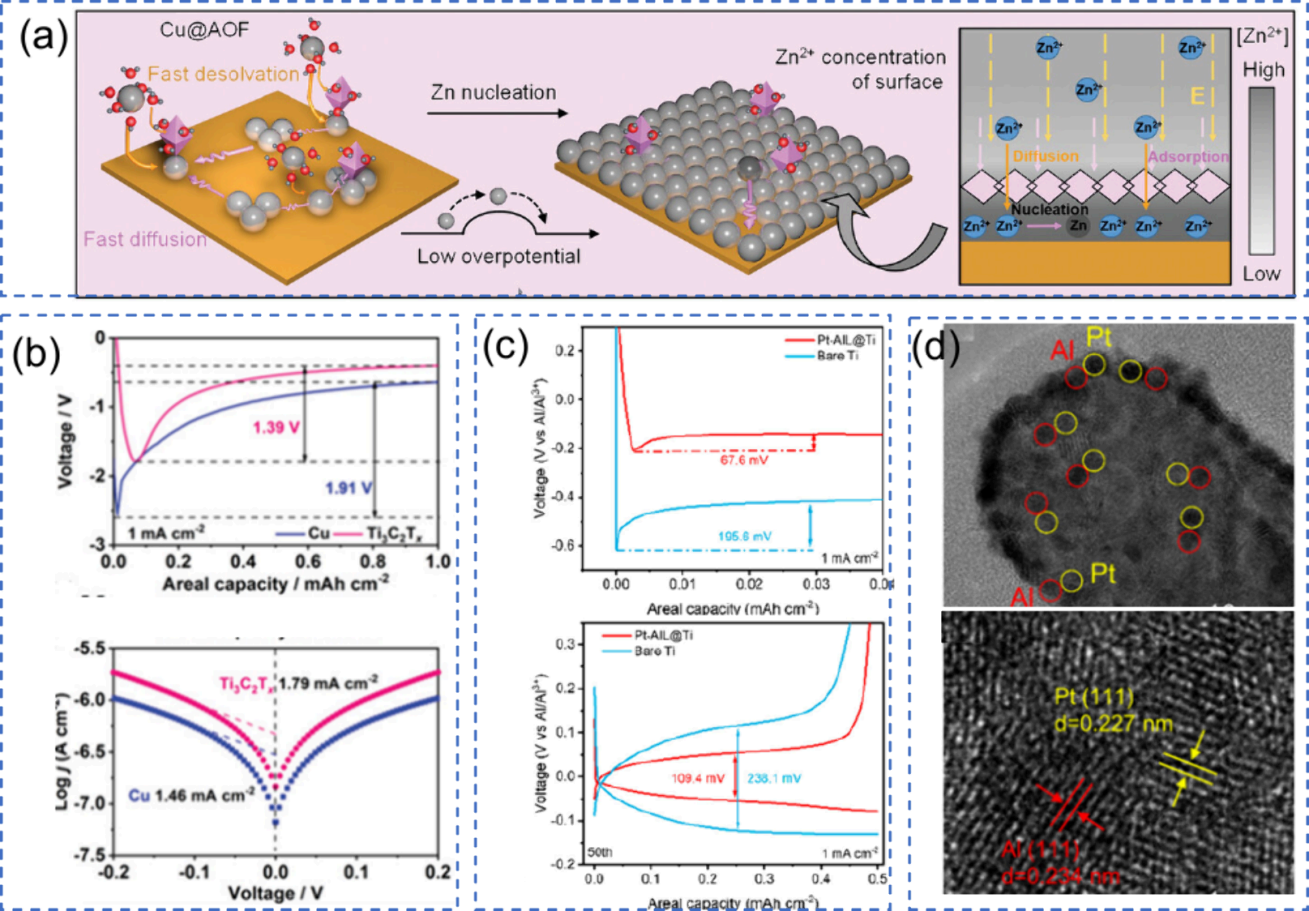
(a) Representation of Zn deposition on a Cu@AOF surface in AF-ZMB. Reproduced with permission,^543^ Copyright 2023 John Wiley and Sons. (b) Initial voltage-capacity curves and Tafel plots of Mg plating on Cu foil and Ti_3_C_2_T_*x*_ film in AF-MMB. Reproduced with permission,^546^ Copyright 2023 Wiley-VCH GmbH. (c) Nucleation and cell overpotential of bare Ti and Pt-AIL@Ti-based AF-AMBs (d) TEM and HRTEM images of Pt-AIL@Ti. (c, d) Reproduced with permission,^549^ Copyright 2023 American Chemical Society.

In anode-free magnesium metal batteries (AF-MMBs), Ti_3_C_2_T_*x*_ MXene films serve as oriented, ion-concentrating interphases: O-terminations support uniform Mg, and F-terminations promote MgF_2_-rich SEI, guiding lateral growth; nucleation overpotential decreases (Cu: ∼1.91 V → MXene: ∼1.39 V at 1 mA cm^–2^) (Figure 45(b),^546^ and higher exchange current enhances kinetics.^547,548^ Complementary hydroxide coatings, α/β-Ni­(OH)_2_ on carbon cloth, reduce nucleation overpotential and improve stability through strong Mg binding, favorable lattice matching, and structured capture sites that favor epitaxial (nondendritic) growth.^502^

In AF-AMB systems, artificial interfaces that exploit lattice matching and aluminophilicity regulate nucleation density and interfacial stability: Au lattice-matching layers improved nucleation density and provided ∼80% retention over 900 cycles with expanded-graphite cathodes,^517^ an ultrathin Pt-aluminophilic interface on Ti (Pt-AIL@Ti) yielded highly reversible, dendrite-free Al plating/stripping, with ex situ high-resolution transmission electron microscopy (HRTEM) showing ∼4–5 nm Al nuclei anchored near Pt nanoparticles (Al(111)/Pt(111) *d*-spacings 0.234/0.227 nm (Figure 45(c, d)).^549^ The Pt-AIL@Ti||Al asymmetric cell enables a stable plating/stripping for over 2000 h at 10 mAh cm^–2^ with an average CE of 99.9%. Interfacial bonding chemistry is equally critical: substrates promoting strong Al-O-C bonds enable uniform Al deposition,^550^ while textured 2D Au nanosheets developed on a stainless-steel electrode (Au@SUS) with minimal lattice mismatch to Al(111) controlled morphology and achieved 80% capacity retention to 1000 cycles in AF-AMB configured as Au@SUS||graphine.^516^

A helpful sequencing rule is “surface first, then interphase”: use affinity promoters to set nucleation density, overlay an ion-selective or mechanically tunable film to guide growth, and ensure that SEI/CEI products penetrate and integrate with the coating’s porous network rather than plugging it, thereby maintaining efficient ionic transport. When electrolyte viscosity or low-temperature kinetics limit charge transfer (Section 6.1.2), selecting higher-permittivity or plasticized coatings (e.g., LiF@PVDF, polymer–ceramic hybrids) can restore interfacial conduction without compromising the modulus. If the local current still concentrates after interphase design, then geometric redistribution via 3D hosts (Section 6.2.3) becomes necessary. Finally, thinness and adhesion are important for AFB practicality: coatings must be extremely thin to minimize mass and energy costs, yet they need to be continuous, strongly adherent, and chemically compatible with both the collector and the plated metal to prevent delamination and resistive growth.

Overall, these results show that artificial interphases—polymeric, ceramic, carbonaceous, and lattice-matched metallic, offer a versatile set of options to (i) reduce nucleation overpotential, (ii) equalize fields and ion flux, (iii) preselect SEI/CEI chemistry, and (iv) promote lateral, compact growth across Li/Na/K/Zn/Mg/Al AFBs, as long as composition, thickness, adhesion, and lattice/chemical matching are carefully optimized with the electrolyte and operating window.

#### 6.2.3 3D Current Collector Architecture

Designing the collector as a porous scaffold changes local kinetics by spreading current, providing internal free space, and directing ion flux.^559,560^ Metal foams and meshes (such as Cu foam, Ni mesh, stainless foams), carbon frameworks (including carbon cloth, graphene sponges, CNT felts, and electrospun nanofibers), and hybrid lattices (for example, 3D-printed structures that are later metallized and made metallophilic) have all shown promise. As shown in Figure 46, their effectiveness comes from^561–563^ (i) lowering effective current density through increased surface area, which helps prevent hotspot formation; (ii) enhancing electrolyte flow via open channels and winding pathways that help balance concentration gradients; (iii) allowing plated metal to grow within pores, reducing shape distortion and dead-metal buildup; and (iv) boosting wettability when doped with oxygen/nitrogen or coated with affinity promoters. Expanding the active area decreases local current density and delays concentration polarization, a phenomenon initially confirmed in lithium-excess cells and later applied to anode-free setups.^564–566^ Importantly, however, 3D “area dilution” only offers advantages when deposition happens inside the scaffold; if not, skin-plating on the outer surface negates the geometric benefit.^411,485^

**Figure 46:**
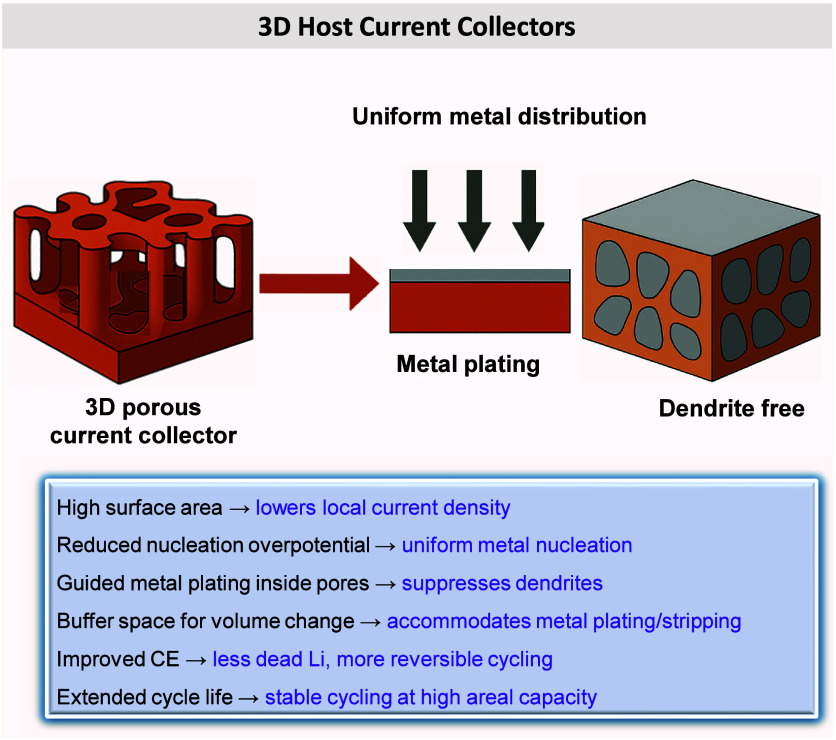
Diagrammatic illustration of the advantages of 3D porous host CCs in AFBs.

Three effective strategies for promoting in-depth filling are (i) pore-scale transport guidance and field shaping to direct ions into channels; (ii) pore-scale transport guidance and field shaping to direct ions into channels; and (iii) hierarchical architectures that combine macro-porosity for electrolyte access with meso/microporosity for nucleation and volume accommodation. Surface-affine chemistries lower the nucleation overpotential and increase nucleus density on internal walls, biasing deposition inward. Li-affine Cu_3_P decoration on 3D Cu promotes uniform Li nucleation and dendrite-free growth (Figure 47(a)).^561^ At the same time, a porous CuZn framework decorated with [Cu­(NH_3_)_2_]Cl guides Li^+^ into tunnels and residual Zn enhances lithiophilicity.^567,568^ Co_3_O_4_-coated Ni foam (Co@NF) tunes the nucleation barrier and maintains high CE across conditions (Figure 47(b));^561^ sodiophilic Bi layers on Cu ensure uniform Na nucleation;^505^ Cu_3_P nanowires on Cu reduce Na adsorption/diffusion barriers;^569^ zincophilic Sb nanoparticles on 3D nano-Cu provide uniform Zn nuclei;^560^ and magnesiophilic motifs such as Au nanoseeds or Ni­(OH)_2_ coatings lower Mg nucleation overpotential and promote dense growth.^501,502^

**Figure 47:**
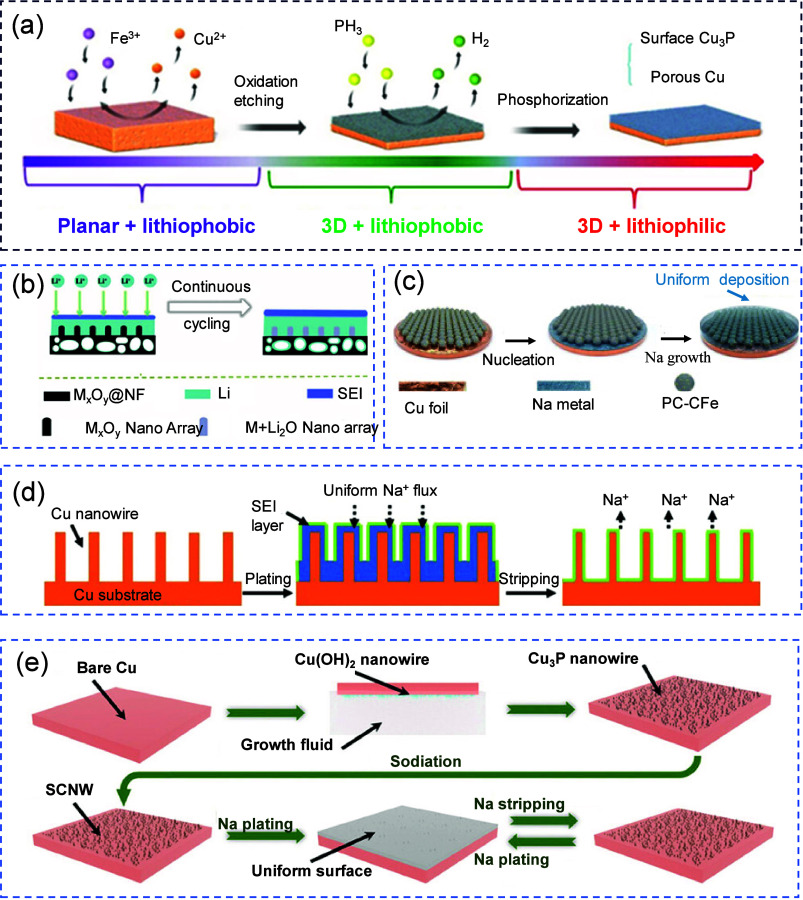
(a) Diagrammatic illustration of the synthesis process of Cu_3_P-decorated 3D copper for AF-LMBs. Reproduced with permission,^561^ Copyright 2024 American Chemical Society. (b) Li plating/stripping behavior of metal oxide-based 3D CC in AF-LMBs. Reproduced with permission,^572^ Copyright 2022 Elsevier. (c) Illustration of uniform Na deposition achieved on PC-CFe-coated Cu foil. Reproduced with permission,^571^ Copyright 2022 Wiley-VCH GmbH. (d) Comparative depiction of Na deposition on flat Cu and Cu nanowire-coated (Cu NW-Cu) substrates. Reproduced with permission,^574^ Copyright 2018 Elsevier. (e) illustration of an in situ synthesis of Cu_3_P@Cu and its Na deposition/stripping behavior. Reproduced with permission,^569^ Copyright 2024 Wiley-VCH GmbH.

Pore-scale transport guidance and field shaping further drive ions into interior channels; for example, combining Ni-Co alloy and ZnO with Cu foam generates micromagnetic fields that push Li deposition deeper into the scaffold, yielding dense layers and enhanced stability.^570^ Hierarchical architectures marry macro-porosity (electrolyte access) with meso/microporosity (nucleation and volume accommodation) to sustain in-depth filling: hierarchical open-channel carbon with Fe nanoparticles (PC-CFe) accelerates ion access and seeds uniform Na plating/stripping (Figure 47(c)),^571^ mesoporous carbon nanofiber films (MCNF) balance access and potassiophilicity to stabilize K cycling and enable high-energy AF-PMBs,^361^ and terminations on Ti_3_C_2_T_*x*_ MXene (O^–^ vs F^–^) guide Mg to plate laterally with lower nucleation overpotential and higher exchange current density.^546–548,572^

Notably, combining 3D topology with a thin, lithiophilic coating can further reduce local current density and the nucleation barrier.^84,485^ Scholars have developed 3D CC for AFBs, and some representative designs are summarized in Table 9. For example, a hierarchical porous Cu (HPCu) CC configured as HPCu||LFP using 1 M LiTFSI in DOL/DME (1:1 v/v) + 2% LiNO_3_ electrolyte achieves 124 mAh g^–1^ discharge capacity at 0.2C and retains 55.5% after 100 cycles with an average CE of 98.3%.^573^

**Table 9:** Performance of AFBs with 3D CC Architecture

Battery type	Cell configuration	Electrolyte	Potential window (V)	Capacity (mAh cm^–2^)	Current	Cycle	CR (%)	CE (%)	Ref.
AF-LMB	HPCu||LiFePO_4_	1 M LiTFSI in DOL/DME (1:1 v/v) + 2% LiNO_3_	3–3.8	0.56	0.2C	100	55.5	98.3	^573^
	Cu@Cu_3_P||LiFePO_4_	1 M LiTFSI in DOL/DME (1:1 v/v) + 2% LiNO_3_	3–3.8	2.9	1C	100	-	98.64	^561^
	Cu_2_O@Cu||LiFePO_4_	1 M LiTFSI in DOL/DME (1:1 v/v) + 2% LiNO_3_	2.5–4	0.55	0.1C	50	-	98.21	^577^
	Cu_2_O coated Cu foam								
AF-SMB	3D Zn@Al||Na_3_V_2_(PO_4_)_3_	1 M NaPF_6_ in DME	2.5–3.8	0.21	0.5C	100	98.8	-	^563^
	Na-SnNCNF||Na_3_V_2_(PO_4_)_3_	1 M NaClO_4_ in EC/DEC (1:1, v/v%) + 5 wt % FEC	0.75–2.8	1.02	10C	1000	92.1	99.93	^578^
	Cu_3_P@Cu||Na_3_V_2_(PO_4_)_3_	1 M NaPF_6_ in diglyme	2.5–3.5	0.7	0.5C	75	87	99.5	^569^
	fluorinated covalent triazine framework, (FCTF)||Na_3_V_2_(PO_4_)_3_	1 M NaPF_6_ in diglyme	2.5–3.8	0.82	2C	300	94.7	-	^579^
	CuNW-Cu||FeS_**2**_	1 M NaPF6 in G2	0.9–3	1.6	0.2 A g^–1^	50	88	97.5	^574^
AF-PMB	Cu-OSe NWs||K_0.5_MnO_2_	4 M KFSI in DME	1.5–3.9	1.33	0.2 A g^–1^	^500^	81.8	98	^580^
AF-ZMB	Cu_3_Zn@Cu ||Zn_3_V_3_O_8_	3 M Zn(CF_3_SO_3_)_2_ in H_2_O	0–1.6	0.18	0.1 A g^–1^	200	80	-	^497^
	ZA@3D-nanoCu||MnO_2_	2 M ZnBr_2_ in H_2_O	0–1.8	0.8	10 mA cm^–2^	1000	89	96.2	^560^
	3D CNT@Cu||VACNT	Zn(OTf)_2_ in H_2_O	0.6–2.5	819 mAh g^–1^	0.4 mA cm ^–2^			81%	^581^

Similarly, in Na systems, 3D Cu foam reinforced with in situ Cu nanowires (CuNW-Cu) in a CuNW-Cu||FeS2 configured AF-SMB using 1 M NaPF_6_ in G2 electrolyte created uniformly distributed nucleation sites, delivering over 320 mAh g^–1^ capacity and retaining 88% after 50 cycles with a CE exceeding 97.5% (Figure 47(d)).^574^ Moreover, in situ-grown Cu_3_P nanowires on Cu (Cu_3_P@Cu), a 3D CC, (Figure 47(e)),^569^ enable uniform Na nucleation and a smooth interface, because Na_3_P provides low Na adsorption energy and a small diffusion barrier, as revealed by first-principles calculations. The Cu_3_P@Cu delivered an average CE of 99.8% over 800 cycles at 1 mA cm^–2^/1 mA h cm^–2^ in an asymmetric Cu_3_P@Cu||Na coin cell. In anode-free full cells, a Cu_3_P@Cu||Na_3_V_2_(PO_4_)_3_ pouch cell using 1 M NaPF_6_ in G2 electrolyte delivered an initial capacity of 87.5 mAh g^–1^ at 0.5 C, with 87% capacity retention and a CE of 99.5% after 75 cycles, demonstrating the effectiveness of this 3D CC.

3D CC are also crucial in aqueous battery systems. For instance, a 3D nano-Cu host decorated with Sb nanoparticles (ZA@3D-nanoCu) provides abundant Zn-affine sites, ensuring uniform current distribution and guiding deposition into compact forms, which supports highly reversible cycling.^560^ The AF-ZMB configured as ZA@3D-nanoCu||MnO_2_ using 2 M ZnBr_2_ in H_2_O achieves 200 mAh g^–1^ at 10 mA cm^–2^ with 89% capacity retention and 96.2% CE after 1000 cycles. Overall, 3D hosts help reduce local current density and promote uniform metal nucleation for Li, Na, K, Zn, Mg, and Al. However, the added surface area can also trigger side reactions and trap dead metal. The most reliable improvements occur when the 3D structure is combined with a durable, metal-attracting surface and an electrolyte that supports it under high areal capacity and pressure.

Limitations include (i) increased mass and volume that reduce specific energy, (ii) fabrication complexity and variability, and (iii) the difficulty of directing deposition deep inside the scaffold rather than on the outer surface. Another practical trade-off is electrolyte use: a very high surface area can increase SEI/CEI formation and decrease the effective cathode/electrolyte ratio, resulting in lower practical energy density.^575,576^ To address these trade-offs, figures of merit should combine geometry and kinetics, for example, match interior pore volume to the targeted plated-metal inventory at the desired CE, verify interior wetting (porosimetry/contact-angle), and measure in-depth filling through operando imaging while tracking nucleation overpotential and impedance. When surface chemistry alone is not enough, field-shaping techniques (such as magnetic CNZ scaffolds) or termination engineering (like MXene O/F terminations) can direct ion paths and growth orientation to take full advantage of 3D topology.

### 6.3 Cathode Materials Design

In AFBs, the cathode is more than just a positive electrode; it is the sole source of active ions. Without preinstalled metal on the anode side, any irreversible loss during formation and cycling must be compensated by the cathode. Practical designs, therefore, require cathodes that combine high capacity with structural resilience and interfacial compatibility, while also providing enough mobile ions to support reversible plating and stripping at the CC.^228,582^ The principles below are chemistry-agnostic and apply to various battery systems; the highlighted figures mainly come from AF-LMBs, but we also note related anchors for other chemistries where they naturally occur.

#### 6.3.1 Cation-Rich Cathodes and Sacrificial Cation Sources

Since the cathode is the only reservoir of working ions, the most effective way to keep an AFB functional is to embed additional cations in the cathode or maintain a sacrificial reserve.^23,150,583^ Several studies have been reported in this regard, as summarized in Table 10. For AF-LMBs, lithium-rich layered oxides and spinel-related Li_2_Ni_0.5_Mn_1.5_O_4_ (L_2_NMO) are a natural choice. They release a significant amount of Li^+^ during the first charge while preserving the host lattice. In situ XRD shows the appearance of L_2_NMO reflections without damaging tetrahedral Li and octahedral transition-metal sites (Figure 48(a)).^584^ The same idea applies to high-Ni layered oxides: Li-rich Li_2_[Ni_0.8_Mn_0.1_Co_0.1_]­O_2_ (L_2_NMC811) forms a lithiated shell around an NMC811 core. Contour XRD and electron energy loss spectroscopy (EELS) observe reversible overlithiation (lattice spacing expanding from 0.473 to 0.506 nm during discharge) and a Ni valence shift from +3 to +2 that disappears on recharge, indicating reversible phase changes instead of permanent damage (Figure 48(b–e)).^585^ With adequate high-voltage surface stabilization, Li-rich cathodes can provide surplus Li^+^ that offsets irreversible formation losses (SEI/CEI), enabling improved capacity retention and longer cycle life.

**Figure 48:**
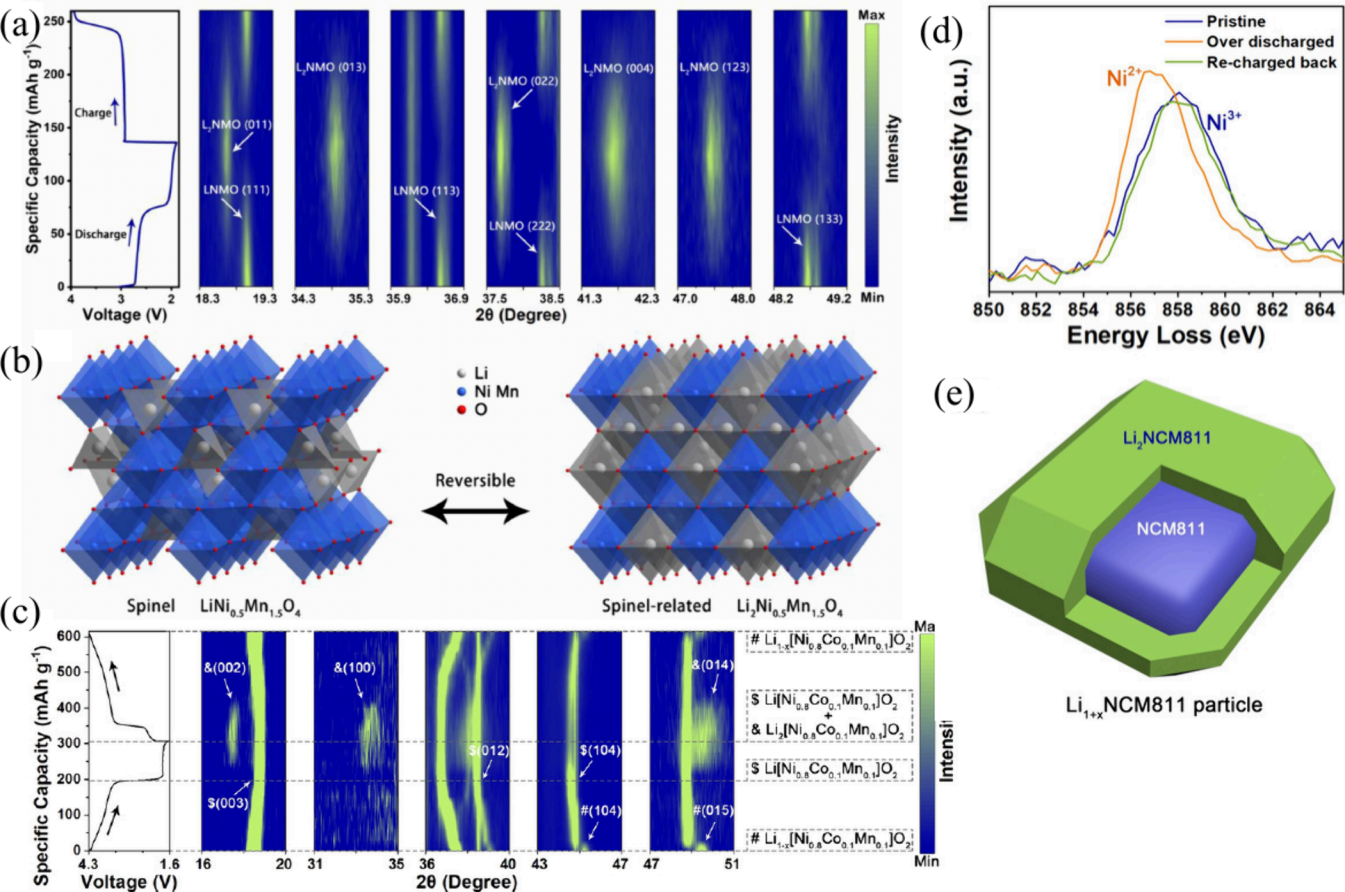
(a) Contour plot showing in situ XRD patterns of an LNMO half-cell alongside the corresponding voltage profiles, (b) crystal structures of LNMO and L_2_NMO. (a, b) Reproduced with permission,^584^ Copyright 2022, Elsevier B.V. (c) Contour plot of in situ XRD patterns for an NMC811 half-cell, accompanied by the respective voltage profiles, (d) EELS spectra of pristine NMC811, overdischarged NMC811, and recharged NMC811 cathodes, (e) a schematic of a lithiated Li_1+*x*_NMC811 particle. (c–e) Reproduced with permission,^585^ Copyright 2021, Wiley-VCH.

**Table 10:** Performance of AFBs with Cation-Rich and Sacrificial Cation Source Cathodes

Battery type	Cell configuration	Electrolyte	Potential window (V)	Capacity (mAh cm^–2^)	Current	Cycle	CR (%)	CE (%)	Ref.
AF-LMB	Cu||NMC811@Li_2_O	1.0 M LiTFI + 1.5 M LiFSI in G3 + 10% HFE (v/v)	3–3.8	4.85	0.2C	300	90	>99	^587^
		HFE: 1,1,2,2- tetrafluoroethyl 2,2,3,3-tetrafluoropropyl ether							
	Cu||NMC622 @LiDF	4.0 M LiFSI + 2.0 M LiTFSI in DME	2.8–4.3	3.7	0.1C	100	78	-	^596^
	Cu||LiFePO_4_ @Li_2_S	PEGDA gelled using 1.0 M LiTFSI in DOL/DME with 2.0 wt % LiNO_3_	2.5–3.8	3.85	0.2C	100	98	99.6	^589^
	Cu||Li_2_Ni_0.5_Mn_1.5_O_4_	6 M LiFSI in DME	3.3–4.8	3.2	0.2C	50	88	-	^584^
	Cu||Li_1.31_NMC811	6 m LiFSI in DME	3–3.8	5.1	1 mA cm^–2^	100	84	-	^585^
AF-SBs	Ni||Li_2_S+Li_2_CS_3_	1 M LiTFSI in DME/DOL (1/1, v/v) + 0.2 M LiNO_3_	1.8–3	1.94	0.2C	125	51.0	95.4	^597^
AF-AMB	Cu||PANI@G-Al_2_TiO_5_	0.5 M Al_2_(SO_4_)_3_ in H_2_O	0.01–1.4	0.07	1.3 A g^–1^	1000	60	90	^598^

As demonstrated in our previous work, controlled oxidative reactions at the cathode/electrolyte interface during the formation step can induce additional electron flow, thereby depositing more Li on CC and effectively increasing the initial N/P ratio in AFBs.^88^ This enhances early cycle reversibility when maintained within a high-voltage stabilization window.^586^ In parallel, sacrificial metal-ion sources embedded in the cathode offer a practical way to compensate for metal loss in the first cycle with fewer structural drawbacks. For example in AF-LMB, Li_2_O dispersed on NMC generates Li^+^ and a dense CEI through O^2–^ reactions with fluorinated ethers (e.g., 1.0 M LiTFSI and 1.5 M LiFSI in triethylene glycol dimethyl ether, G3), and pouch-format Cu||NMC@Li_2_O AFBs have been reported to deliver approximately 2.46 Ah (around 320 Wh kg^–1^) with about 80% capacity retention after 300 cycles.^587^ Li_3_N can serve a similar purpose,^588^ although gas-forming byproducts require caution. Li_2_S has also been used as a sacrificial source; in GPE-based AF-LMBs (LFP@Li_2_S with an ether gel), approximately 16.5 wt % Li_2_S enabled about 65% retention over 500 cycles at 0.5 C (initially 154 mAh g^–1^) and approximately 98% retention over the first 100 cycles.^589^ This concept of inventory-buffering extends beyond Li: Na-rich layered oxides and Na-excess polyanions supply additional Na^+^ for sodium-based AFBs,^152,590,591^ while K-rich layered oxides and Prussian-blue analogs can support potassium-based AFBs, provided that structural “breathing” and electrolyte compatibility are carefully managed.^592^ For multivalent AFBs (Mg, Al), maintaining a sufficient cation inventory is even more essential; cathodes that reversibly host divalent or trivalent ions, or composites with sacrificial reservoirs, help offset early losses without causing significant strain.^153,593–595^

Overall, as schematically shown in Figure 49, cathodes such as NMC, MnO_2_, and Prussian-blue analogs can be paired with either premetalation (to preload working ions) or with sacrificial additives embedded in the positive electrode to “pay the ion bill” during formation and early cycling. In Li systems, Li_2_O, Li_3_N, LiNO_3_, Li oxalate and Li_2_S exemplify this approach and directly correspond to the case studies discussed above.^584,585,587–589^ The same logic applies to Na (Na_3_P, Na_3_N, Na_2_C_2_O_4_), K (K_2_S), Zn (ZnO), and even multivalent chemistries where Mg_3_N_2_ or Al-bearing sources (e.g., Al_2_O_3_, AlCl_3_-EMImCl) can serve as ion reservoirs or premetalation partners to maintain CE.^153,593–595^

**Figure 49:**
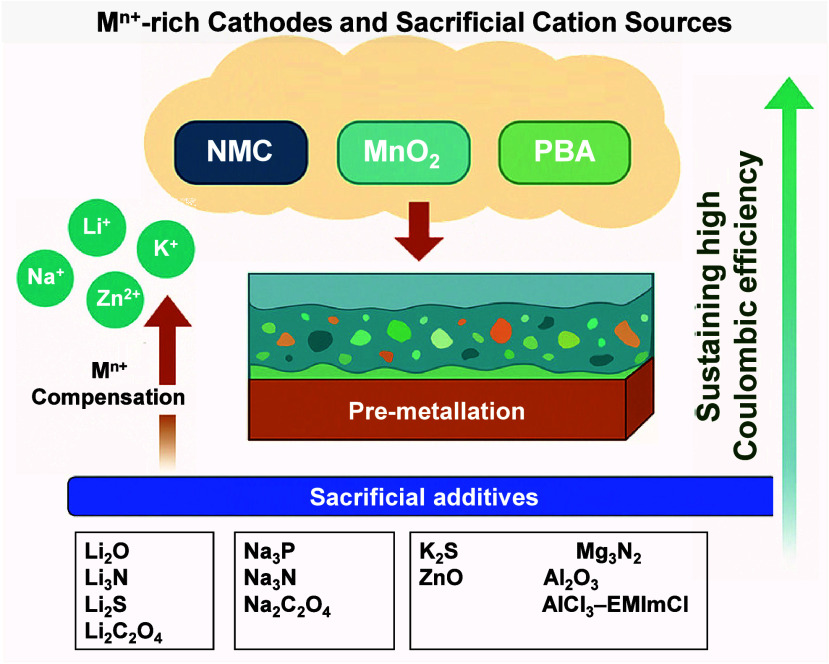
Cation-rich cathodes and sacrificial ion sources for AFBs.

#### 6.3.2 Cross-Talk Suppression

Securing the ion budget is essential, but not sufficient. If cathode-derived species or high-voltage byproducts escape, they can cross the separator and poison the metal nucleation and cause SEI fracture at the anode.^599–601^ These issues occur across different chemistries: transition-metal dissolution from layered or spinel oxides (especially Mn^2+^ and Ni), soluble intermediates from conversion cathodes (polysulfides in S-based systems), and electrolyte oxidation products at high voltage (HF, CO_2_, radicals in organics; reactive oxygen species in aqueous cells). Each of these speeds up SEI damage and causes uneven plating, lowering CE and raising the risk of short circuits.

Two main factors at the cathode are interphase quality and operating voltage. Regarding the first, thin, inorganic-rich CEIs and conformal coatings prevent dissolution and limit solvent access. As demonstrated in Figure 50, Al_2_O_3_, TiO_2_, and Li_3_PO_4_ shells are well-known barriers; fluorinated salts and solvents like LiFSI or NaFSI tend to form LiF and NaF-rich CEIs with the mechanical strength needed for high-voltage cycling. In Ni-rich layered oxides, combining surface coatings with bulk stabilizers reduces parasitic reactions and has become an effective way to control TM dissolution, precisely the safeguard needed when these oxides are repurposed for AFBs.^602,603^ Similar methods are emerging for Na- and K-based AFBs: NaNO_3_ and NaFSI-based electrolytes promote strong inorganic CEIs on Na-rich layered oxides and help prevent dissolved Mn from reaching the plating side, while K-AFBs benefit from fluorinated salts that encourage KF-rich, mechanically durable layers on high-voltage K hosts.^604,605^

**Figure 50:**
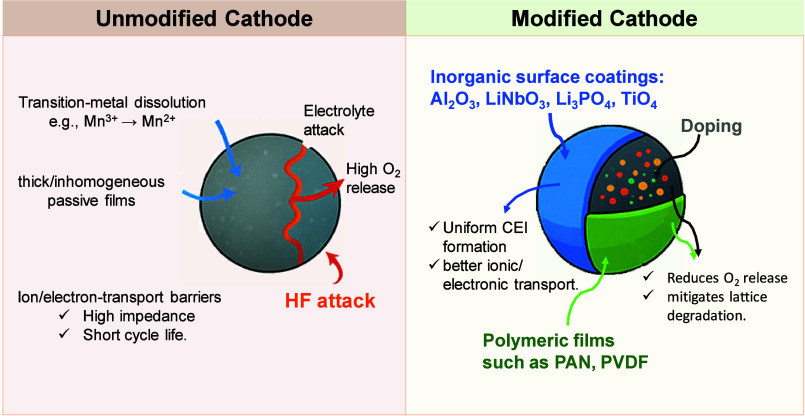
Illustration of cathode surface-modification using different modifiers in suppressing the cross-talk effect and interface stabilization in comparison to the unmodified one.

On the second level, the voltage applied to the cathode directly controls its reversibility at the plating interface. In three-electrode experiments, NMC811∥Cu cells exhibit transport-limited charging, fluctuating nucleation overpotentials, and increasing stripping overpotentials, resulting in capacity loss. Meanwhile, LFP∥Cu maintains low, stable overpotentials and higher CE under the same conditions. Operando NMR tracks the additional loss of Li inventory in the high-voltage case.^29^ The same principle applies across different chemistries: lower-voltage Na and K hosts tend to be more tolerant in AFB systems unless high-voltage surfaces are exceptionally well protected. In aqueous Zn AFBs, modest upper cutoff voltages limit OER-driven oxidative products that would otherwise migrate and destabilize Zn plating.^606,607^

Chemistry-specific cross-talk mechanisms build on this foundation. In Li_2_S-based AFBs, thiometallates like (NH_4_)_2_MoS_4_ and (NH_4_)_2_WS_4_ act as polysulfide buffers at the cathode, capturing long-chain species into well-defined dinuclear complexes, shortening chains through S^0^ uptake, and seeding a Mo/W-enriched SEI without draining the electrolyte, effects observable in UV–vis and WAXS-PDF signatures (Figure 51(a–c)).^608,609^ Tellurium plays a beneficial role: it dissolves via polysulfides, migrates, and forms a Li_2_TeS_3_-enriched passivating layer at the plating interface, creating a bilayer SEI (Li_2_S/Li_2_Te near the metal; Li_2_TeS_3_ near the electrolyte) that significantly improves Li recyclability (Figure 51(d–f).^610^ A polymerizable electrolyte additive (TTCA-Li) can polymerize in situ on Li_2_S during the first charge, forming a poly organosulfide coating that boosts CE above 99.5% by guiding conversion pathways.^611^ For aqueous Zn-AFBs with MnO_2_-type cathodes, mild electrolytes containing Mn^2+^ additives buffer dissolution and reduce Mn leakage to the anode, which otherwise poisons Zn plating; related chelation strategies, such as citrate or EDTA-like agents, are being explored to trap dissolved metals before they pass through the separator.^612,613^ In Al-based AFBs, where trivalent species and corrosive intermediates complicate matters, stable CEIs and interfacial layers matched to the lattice on graphitic hosts are essential to prevent Al-bearing complexes from drifting and damaging the plating interface.^614,615^

**Figure 51:**
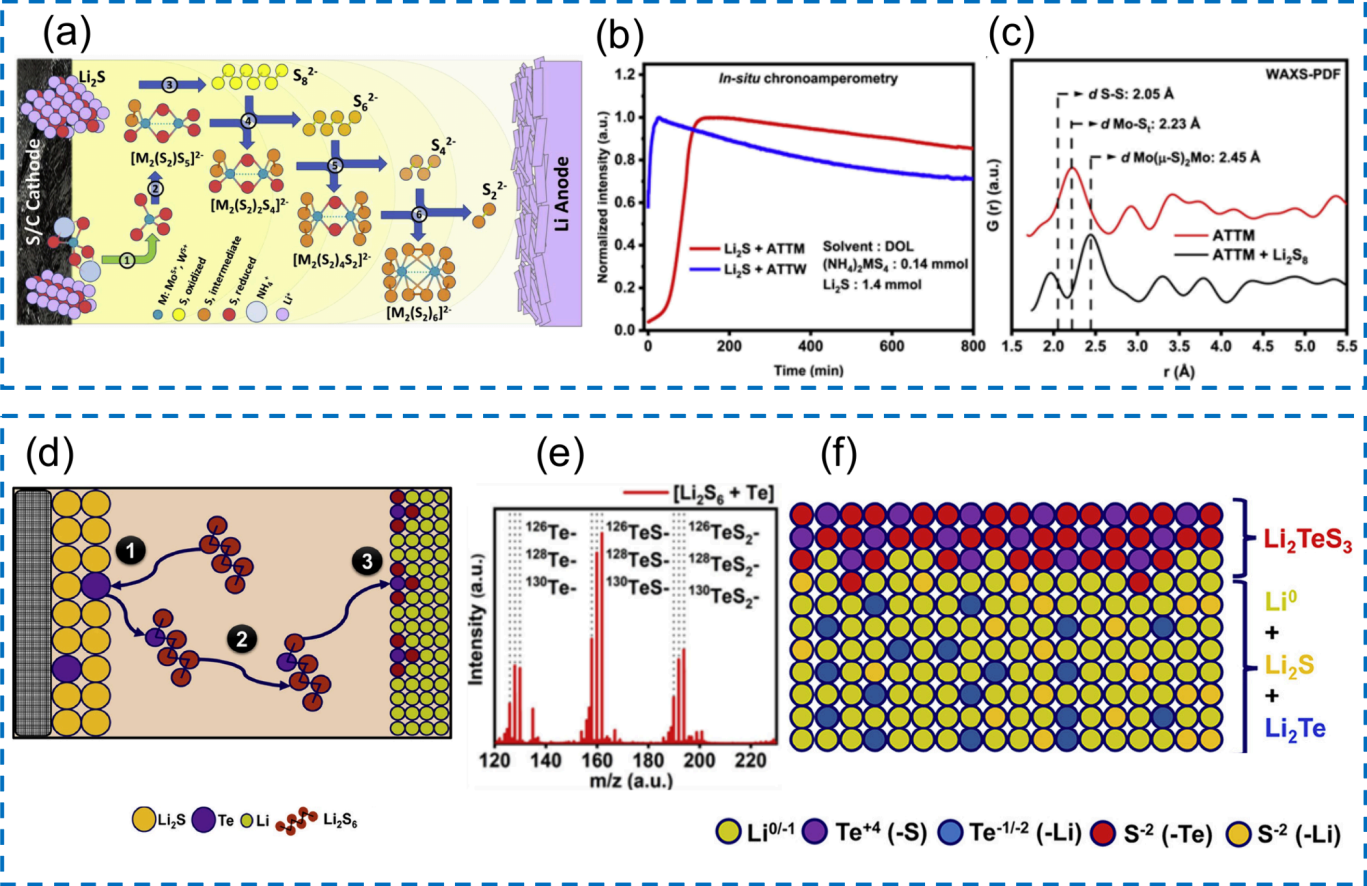
(a) Diagram illustrating the introduction of MS_4_^2–^ species into the electrolyte, their dimerization, and subsequent reaction with long-chain polysulfides through sulfur atom (S^0^) uptake, (b) in situ chronoamperometric reaction curves for (NH_4_)_2_MS_4_ and Li_2_S, showing the rate at which redox-active MS_4_^2–^ species are introduced into the electrolyte, (c) WAXS-PDF analysis of the reaction product from (NH_4_)_2_MS_4_ and Li_2_S_8_. (a–c) Reproduced with permission,^608^ Copyright 2022 Elsevier. Proposed mechanism for tellurium (Te) behavior in Li_2_S cathodes; (d) Te dissolution via polysulfides, (e) its migration to the anode and subsequent formation of Li_2_TeS__3_3_ on lithium deposits, and (f) a dual-layer structure of tellurized and sulfurized Li SEI. (d–f) Reproduced with permission,^610^ Copyright 2020 Elsevier.

Additionally, the choices of electrolyte and separator also play an essential role. Nitrates (LiNO_3_/NaNO_3_) create N-rich interphases that passivate both electrodes in Sulfur-based AFBs. Concentrated or hybrid electrolytes reduce solvent activity and expand the potential window. Chelators can bind dissolved Mn^2+^/Ni^2+^ before they reach the anode.

#### 6.3.3 Structural Engineering

In AFBs, the cathode must deliver energy and compensate irreversibility, so weak architectures quickly suffer from high polarization, volume-change-induced contact loss, and soluble intermediates that shuttle and poison interfaces.^616^ Structural engineering addresses these challenges by creating percolating electron networks, through-plane ionic highways, buffered void spaces, and chemically active sites that trap/catalyze intermediates.^602,603,617,618^ As summarized in Figure 52, the goal is to convert these choices into lower overpotential, uniform ion flux, confined species, and high-loading stability with a durable CE across conversion-type cathodes, especially for AF-SB systems.^619,620^ In practice, effective designs combine a continuous conductive scaffold, hierarchical porosity with internal free volume, and elastic binders, along with interactive surfaces that lower activation barriers.

**Figure 52:**
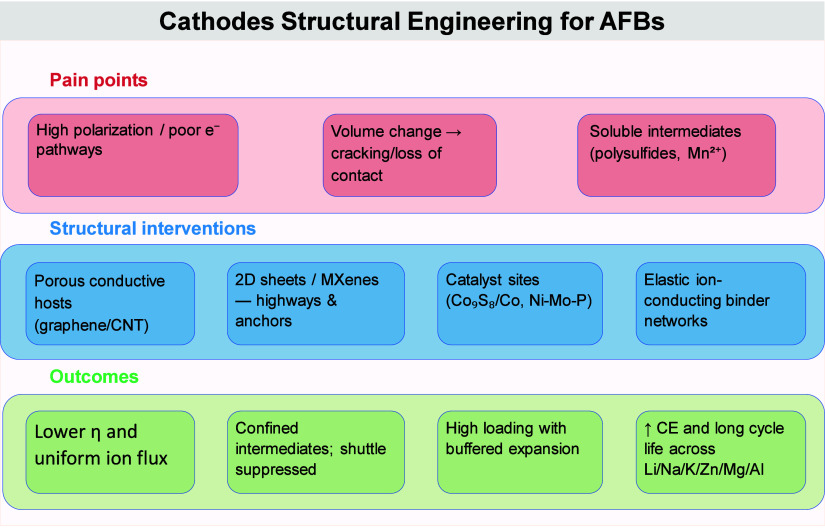
Schematic of structural engineering of conversion-type cathodes for AFBs linking pain points, interventions, and outcomes.

Table 11 provides an overview of scholarly developments in 3D CCs for AFBs. Conductive, porous hosts offer the most general solution. Graphene, graphene oxide interlinked poly-1,3-dioxolane (GPD), carbon nanotubes (CNTs), and customized porous carbons provide continuous electron pathways and internal voids that buffer expansion while confining soluble intermediates. Framework materials enhance chemical selectivity: MOFs and COFs feature tunable pore environments that can be engineered to adsorb specific intermediates;^621,622^ MXenes (e.g., Ti_3_C_2_T_*x*_) offer high conductivity and dense functional groups, serving as both highways and anchors.^623,624^ In conversion systems, architecture directly influences the activation barrier. Embedding Li_2_S in a Co_9_S_8_/Co-decorated carbon matrix lowers the first-charge barrier from approximately 3.3 to 2.3 V and increases capacity (969 mAh g^–1^ in anode-free format), because Co_9_S_8_/Co provides abundant nucleation sites and accelerates redox reactions (Figure 53(a–c)).^168^ A bimetallic phosphide catalyst (Ni–Mo–P) advances this further: operando XRD shows complete Li_2_S → β-S conversion by the end of the first charge when Ni_*x*_Mo_*y*_P_*z*_ is present, while Li_2_S signatures remain without it; the Li_2_S@Ni_*x*_Mo_*y*_P_*z*_@C composite retains about 50% after 300 cycles and reaches 703 mAh g^–1^ in pouch-based AFB (Figure 53(d)).^625^ These catalysts are integrated into the cathode’s structure; they reside inside the composite and modify transport and kinetics from within.

**Figure 53:**
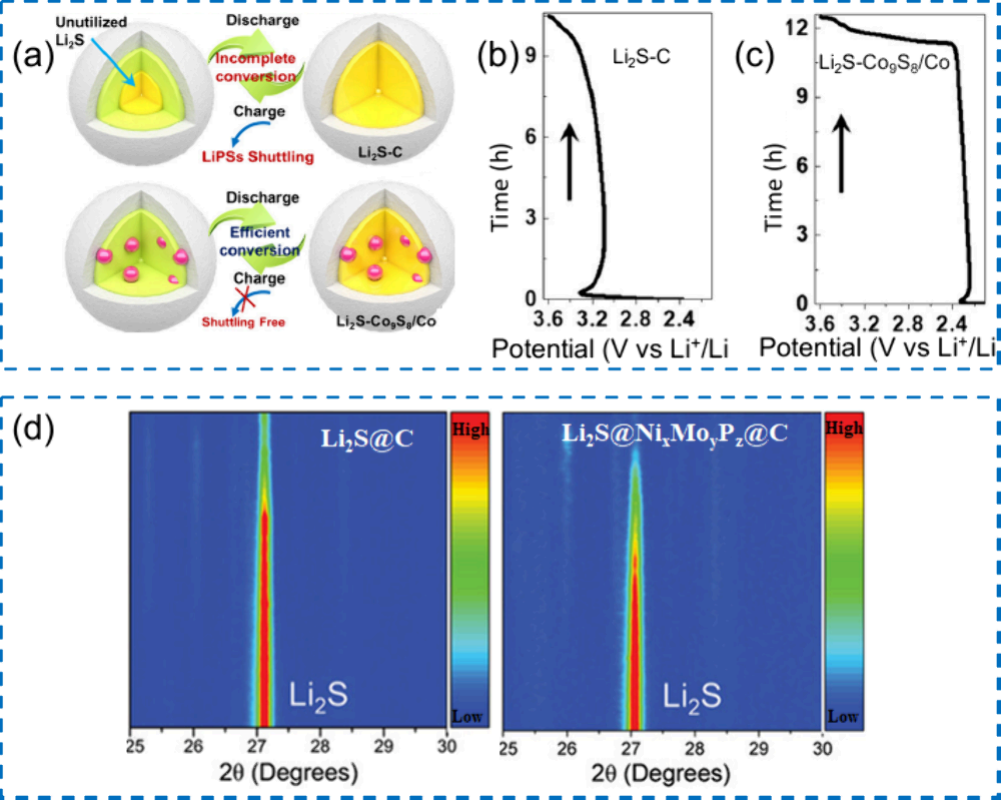
(a) Diagram illustrating the benefits of Li_2_S-Co9S8/Co in Li-S batteries, First-cycle charge profiles for (b) Li_2_S-C and (c) Li_2_S-Co_9_S_8_/Co cathodes. (a–c) Reproduced with permission,^168^ Copyright 2022 American Chemical Society. (d) In situ XRD patterns during the initial charging process for Li_2_S@C and Li_2_S@Ni_*x*_Mo_*y*_P_*z*_@C cathodes. Reproduced with permission,^625^ Copyright 2024 Wiley-VCH GmbH.

**Table 11:** Performance of AFBs with Cathode Structural Engineering Strategies

Battery type	Cell configuration	Electrolyte	Potential window (V)	Capacity (mAh cm^–2^)	Current	Cycle	CR (%)	CE (%)	Ref.
AF-SBs	Ni||Li_2_S+NH_4_)_2_WS_4_	1 M LiTFSI in DME/DOL (1:1 v/v) + 0.25 M LiNO_3_	1.8–2.8	3.65	0.2C	200	43.8	-	^608^
	Ni||Li_2_S+Te	1 M LiTFSI) in DME/DOL (1:1 v/v) + 0.1 M LiNO_3_	1.8–2.8	2.48	0.2C	240	50.0	96.4	^610^
	Ni||Li_2_S+PEO/MHPP	1 M LiTFSI) in DME/DOL (1:1 v/v) + 0.3 M LiNO_3_	1.7–2.8	3.67	0.2C	100	52.2	-	^626^
	Ni||Li_2_S+Co_9_S_8_/Co + Te	1 M LiTFSI) in DME/DOL (1:1 v/v) + 0.2 M LiNO_3_	1.8–2.8	4.61	0.1C	100	84.4	-	^168^
	Ni||Li_2_S+Te (90:10 wt %)	1 M LiTFSI) in DME/DOL (1:1 v/v) + 0.25 M LiNO_3_	1.8–3	2.49	0.2C	300	38.0	91.3	^627^
	Cu||Li_2_S+In_2_Se_3_	1 M LiTFSI) in DME/DOL (1:1 v/v) + 0.2 M LiNO_3_	1.7–2.8	3.43	0.2C	160	59.9	98.3	^628^
	Ag@Cu||Li_2_S+GPD	1 M LiTFSI) in DME/DOL (1:1 v/v) + 2 wt % LiNO_3_	1.7–2.8	1.33	0.2C	100	59.9	98.0	^629^

Mechanical coherence forms the other half of structural design. For example, high-mass-loading sulfur electrodes benefit from binders that are both tough and ion-conductive. A binary PEO-quadripolymer network reinforces the electrode, creates multiple pathways for Li-ions, and traps polysulfides. Using the binary PEO-quadripolymer binder, anode-free Li_2_S cells (≈5.4 mg cm^–2^; E/S ≈ 7 μL mg^–1^) retained ∼79% after 100 cycles, compared to the controlled PEO-PVP binder, which retains 67% after 50 cycles under the same conditions.^626^ Similar strategies, such as elastic binders, hierarchical porosity, and continuous conductive scaffolds, are being adopted in Na- and K-AFBs to accommodate larger ionic radii and increased framework breathing, as well as in Zn-AFBs to support fast proton and metal-ion transport in mildly acidic media without pulverization.^152^ Ultimately, the same structural principles also appear in multivalent AFBs. For instance, a composite Mg_2_Mo_6_S_8_-MgS cathode uses Mg_2_Mo_6_S_8_ to catalyze MgS decomposition (reducing conversion hysteresis). It adsorbs polysulfides, while a Mg^2+^-conductive, electron-insulating SEI protects plated Mg. The proof-of-concept achieves 190 mAh g^–1^ with approximately 92% retention after 100 cycles,^153^ suggesting that architecture and internal catalysis could unlock otherwise limited host chemistries for Mg and other multivalent systems.^593–595^ Although the bare ionic radius of Mg^2+^ (∼0.72 Å) is smaller than that of Li^+^ (∼0.90 Å), the species that reaches the cathode in practical electrolytes is the solvated Mg^2+^ complex, which is effectively larger and has a higher desolvation barrier. Therefore, cathode design should focus on the solvated ion: providing wide, well-connected pores, adding desolvation-assisted sites (such as Lewis-acidic or polar functionalities), and using graded wettability to guide entry, along with Mg^2+^-conductive coatings or interphases to ensure uniform charge transfer. This combination of architecture and chemistry helps reduce transport bottlenecks for Mg and other multivalent ions.

### 6.4 Separator Design

The separator also functions in the cell by guiding ions, reducing dendrite growth, preventing cross-talk, and ensuring safety. Traditional polyolefin membranes like polyethylene (PE) and polypropylene (PP) were designed for conventional lithium-ion cells. However, they often have poor wetting properties, lack stiffness, and provide no chemical protection selectivity.^630–632^ The AFB field, therefore, needs separators that are chemically active, mechanically robust, and structurally designed to extend lifespan across Li, Na, K, Zn, Mg, and Al systems. Representative separator-based designs for AFBs are summarized in Table 12.

**Table 12:** Performance of AFBs with Separator Designing Strategy

Battery type	Cell configuration	Electrolyte	Potential window (V)	Capacity (mAh cm^–2^)	Current	Cycle	CR (%)	CE (%)	Ref.
AF-LMB	Cu||NMC532	1 M LiPF_6_ in EC/DEC (1:1 v/v) + 5 vol % FEC	2.5–4.3	2.12	0.2 mA cm^–2^	100	18.2	98.0	^671^
		PVDF-HFP/Kevlar separator integrated with Cu							
	Cu||LiFePO_4_	LiFSI-1.2DME-3TTE using LFO@6FAP coated PP separator, where LFO = Li_5_FeO_4_ and 6FAP is hexafluoropropane	3–3.8	2.53	1.0 mA cm^–2^	200	73.1	98.4	^674^
	Cu||NMC811	Sb-coated PP/PE/PP trilayer separator	2.7–4.2	0.97	0.5C	60	93.6	99.8	^643^
AF-SMB	Al@C||NNMFO	1 M NaPF_6_ in diglyme using Al_2_O_3_-coated PP/PE/PP trilayer separators	2.0–4.0	0.89	1C	100	69.2	-	^675^
	Cu||Na_3_V_2_(PO_4_)_3_	1 M NaPF_6_ in G2 using solvent adsorption separator with Na supplementation (SAS-N)	2.2–3.8	0.04	2C	600	95.1	-	^676^
AF-PMB	Cu||K_0.51_V_2_O_5_	3 M KTFSI in DME using LiNO_3_@graphine modified glass fiber separator	2–3.5	0.14	0.5 A g^–1^	130	97	98	^538^
	rGO@Cu||K_0.51_V_2_O_5_	3 M KTFSI in DME rGO modified separator	2–3.4	0.5	0.5 A g^–1^	70	95.2	99.7	^539^

#### 6.4.1 Transport and Selectivity Engineering

The way ions move through the separator mainly determines whether plating remains uniform or becomes unstable. High cation transference numbers are particularly crucial in this context. When t^+^ is low (as it usually is, below about 0.4 for polyolefins in carbonate media), concentration gradients form quickly.^633,634^ Aqueous systems pose their own challenges: freely migrating anions, such as sulfate or chloride, can destabilize the cathode and accelerate side reactions, including hydrogen release and evolution.^635,636^ To address these issues, ion-exchange “skins” on the separator surface create Donnan exclusion, slowing problematic anions while allowing the working cations to pass; this same principle also controls polysulfides in Li/Na-S batteries and dissolved Mn^2+^ in Zn-MnO_2_ cells. Porous molecular sieves, MOF or COF interlayers with subnanometer windows, further improve selectivity by physically filtering bulky anions like TFSI^–^, polysulfides, or AlCl_4_^+^ without blocking cation flow.^637–639^ An even more advanced method involves tethering the anion to the polymer backbone to make a single-ion conductor; by immobilizing the counterion, these separators can increase t^+^ above ∼0.7 and reduce concentration polarization at the plating interface.^640^ In water-based electrolytes, zwitterionic or amide-rich coatings stabilize Zn^2+^ solvation, cut down OH^–^ crossover, and boost the overpotential for hydrogen evolution.^635,639,641,642^ As schematically shown in Figure 54, a well-designed AFB separator functions like an ion-selective membrane, guiding the “right” ions. It filters out the “wrong,” providing more time before gradients and instabilities develop.

**Figure 54:**
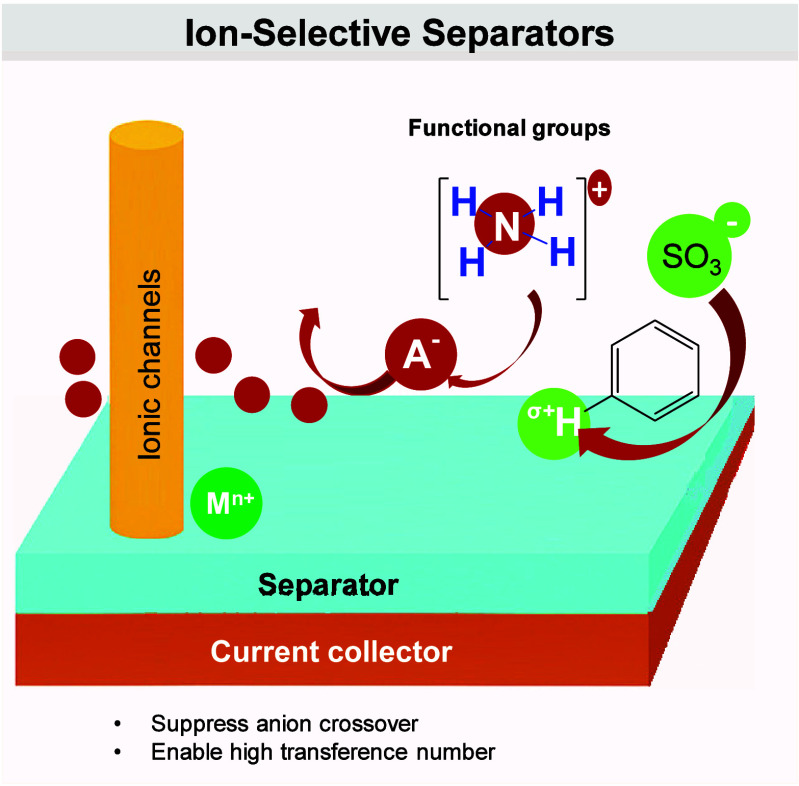
Ion-selective separators incorporating functional groups and ionic channels to regulate ion transport.

Similarly, a metallically functionalized separator can directly participate in plating-side chemistry: an amorphous Sb-coated separator enhances the effective Li–Sb alloying kinetics at the membrane interface, improves Li wetting on pristine Cu CC.^643^ It lowers the nucleation overpotential and produces compact, dendrite-free deposited Li. An NMC811/Cu, full cell AFB achieved a 99.8% CE and 93.6% capacity retention after 60 cycles at 0.5C, whereas cells with a pristine separator performed worse. This type of separator-level metallization shows how transport control and interfacial chemistry can be coengineered to stabilize plating in AFBs. In the AF-PMBs, a layer is also created that facilitates the diffusion of K^+^ ions, ensuring consistent ion transport across the separator.^641,644,645^ For instance, reduced graphene oxide (rGO) coated glass-fiber (rGO/GF) separator directs K^+^ flux, prevents dendrite formation, and, due to its potassiophilicity, helps form a stable, KF-rich SEI on Cu.^538,539^ This design results in smoother K plating and stripping,^539^ and higher peak currents in cyclic voltammetry,^538^ indicating improved ionic conduction pathways and controlled cation diffusion. The stability improvements are primarily attributed to rGO, underscoring that carefully selected, lightweight separator coatings can significantly impact both ion transport and interphase development in AF-PMBs.

#### 6.4.2 Mechanical Stabilization

Separators not only impact the ion transport but also impact the local current distribution, which is critically important when metal grows directly on a bare CC.^646,647^ Small variations in ion flux can cause mossy or dendritic deposits.^648^ Adding stiff ceramic fillers, such as Al_2_O_3_, SiO_2_, or garnet-type LLZO, to a polyolefin matrix increases the modulus to the gigapascal range, reduces shrinkage, and disperses stress (Figure 55). Hence, tips are less likely to puncture through. For example, studies in AF-SMBs demonstrated that thin ceramic “skin” (e.g., Al_2_O_3_ on PP) raises surface modulus and polarity, homogenizes Na^+^ flux, and suppresses protrusions by distributing local stress at the CC.^649^

**Figure 55:**
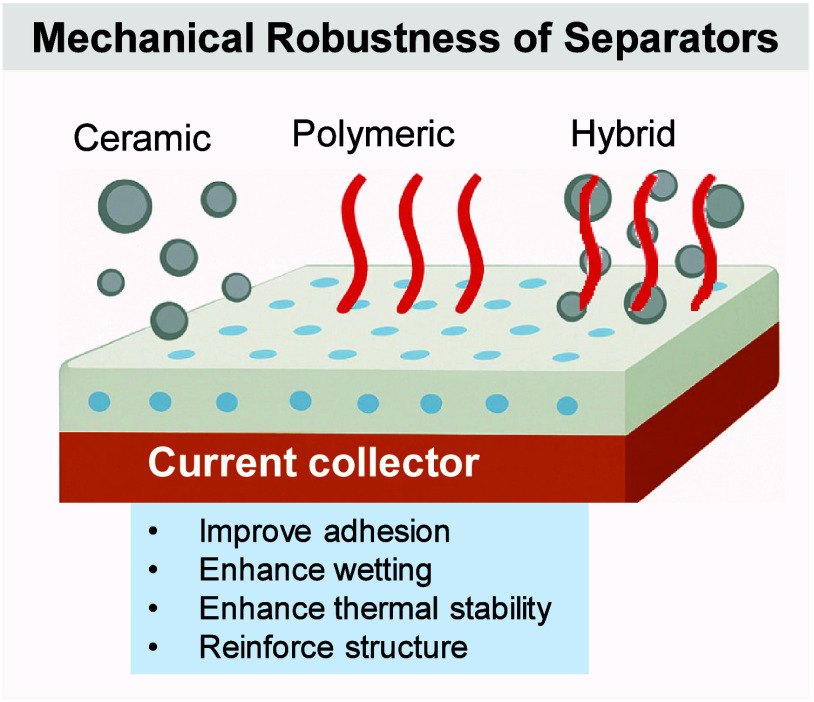
Functional coatings on separators, including ceramic, polymeric, and hybrid layers, designed to improve mechanical robustness.

Electrospun mats made from PVDF-HFP, PAN, or PI offer different benefits.^650^ They are highly porous, low-tortuosity, and wet easily, allowing ions to flow more smoothly. The result is more uniform plating, whether involving Zn^2+^ in aqueous or Li^+^ in organics.^651,652^ Designing the through-plane geometry also helps. Vertically aligned channels and ridged or patterned surfaces lower local current density and even out concentration fields. This strategy is beneficial in Mg and Al chemistries, where interfacial kinetics are slow and hot spots tend to dominate.^653,654^ Gradient designs push this concept further: a dense, more rigid surface facing the plating side resists protrusions, while a more open surface toward the cathode maintains low polarization.^636,644^ When stiffness, wetting, and structure are balanced, the separator becomes a quiet stabilizer, preventing short circuits and supporting flat, compact growth under lean-electrolyte conditions.

#### 6.4.3 Interfacial Chemistry and Scavenging

In AFBs, the separator does more than facilitate ionic conduction and physically isolate the electrodes. It actively manages interfacial chemistry, reducing degradation pathways that originate at the cathode and spread to the delicate plating surface on the CC.641. Cross-talk from soluble intermediates, acidic species, transition-metal dissolution, and parasitic gas reactions can significantly destabilize the SEI/CEI and accelerate capacity loss. As illustrated in Figure 56, functional coatings on separators, whether catalytic or adsorptive, provide a valuable platform for capturing unwanted species and preventing interfacial degradation.^234,655^

**Figure 56:**
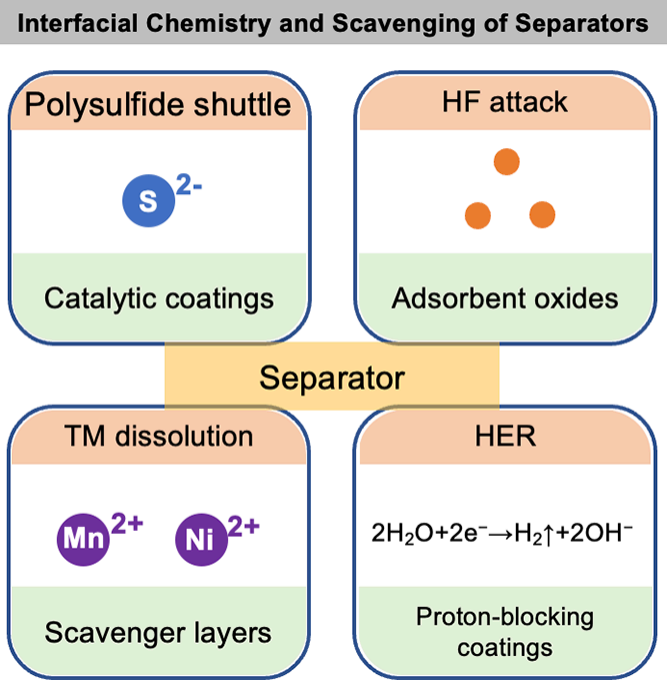
Interfacial chemistry and scavenging strategies enabled by functional separator coatings in AFB.

##### 6.4.3.1 Polysulfide Shuttle Suppression through Catalytic Coatings

In Li-S and Na-S AFBs, soluble polysulfides (Li_2_S_6_, Li_2_S_8_, Na_2_S_6_) easily diffuse across the separator, leading to parasitic reduction at the CC and causing dendritic growth.^644,656^ Catalytic coatings made from transition-metal oxides, such as TiO_2_,^657^ MnO_2_,^658^ Co_3_O_4_,^659^ or sulfides such as MoS_2_,^660^ or MXenes (Ti_3_C_2_T_*x*_),^661^ can adsorb polysulfides and speed up their conversion into insoluble, short-chain species. This catalytic anchoring keeps redox reactions close to the cathode, decreases shuttle-induced CE losses, and promotes more uniform plating at the CC.^662^

##### 6.4.3.2 Adsorption of Acidic and Metallic Contaminants

In carbonate electrolytes containing PF_6_^–^ salts, trace water leads to the formation of HF, and high-voltage operation accelerates the dissolution of Mn^2+^, Ni^2+^, and Co^2+^ from layered or spinel cathodes. Both HF and TM ions migrate to the plating side, where they corrode the SEI or promote parasitic reactions. Separator coatings with high adsorption affinity, such as Al_2_O_3_, MgO, CeO_2_, or porous carbons, trap HF and TM species before they migrate.^663–665^ This adsorption protects both the cathode and the plated metal interface, helping to extend cycle life and reduce interfacial instability.

##### 6.4.3.3 Suppression of Hydrogen Evolution in Aqueous Systems

In aqueous Zn and Al AFBs, the hydrogen evolution reaction (HER) is a major parasitic process that consumes electrons, produces bubbles, and destabilizes the local pH. This not only wastes charge but also accelerates electrode corrosion and passivation. Separator coatings designed with HER-suppressing functionalities, such as hydrophobic polymers, ZnO/TiO_2_ nanoparticles, or ion-selective membranes, limit proton transport and prevent hydrogen reduction.^666–668^ By selectively allowing migration of Zn^2+^ or Al^3+^ while blocking protons, these coatings enhance CE and facilitate smoother metal plating and stripping.

The most effective strategies combine multiple functions into hybrid separators. For instance, carbon-coated separators with embedded metal oxides can simultaneously catalyze polysulfide conversion, adsorb HF, and immobilize TM ions. Hybrid ceramic–polymer coatings offer both mechanical strength and chemical selectivity.^669–671^ These multifunctional approaches recognize that separator design is not just about fixing one failure mode, but about creating a chemical protection system that blocks multiple degradation pathways simultaneously.^672,673^

Future advancements in separator-based interfacial chemistry are likely to focus on adaptive and multifunctional designs. Smart separators with redox-active coatings, self-healing scavengers, or pH-responsive ion channels could dynamically reduce crossover species based on operating conditions. Crucially, the scalability of these functional modifications must be balanced with cost and manufacturability to make commercial AFBs viable. By rethinking separators as chemically active interfacial regulators instead of passive membranes, researchers can overcome some of the biggest hurdles to practical, long-lasting AFBs.

## 7 Anode-Free All-Solid-State Batteries

All-solid-state batteries (ASSBs) replace flammable liquids with inorganic or polymeric solid electrolytes, providing a safer and thermally stable way to store energy.^677–681^ When combined with an anode-free design (AF-ASSB), this approach offers a promising path to achieving higher gravimetric and volumetric energy densities while also improving resistance to abuse.^682–686^ For example, as shown in Figure 57, in Cu∥NMC811, 4 mAh cm^–2^ AFB with both separator and sulfide SE set at 20 μm is supposed to have an identical thickness (119.1 μm) of the cell, resulting in the same volumetric energy density (1243 Wh L^–1^). However, adding a realistic +15% separator swelling in the liquid cell (from 20 to 23 μm) increases stack thickness to 122.1 μm. It reduces volumetric energy density to 1212 Wh L^–1^, while the areal capacity remains unchanged. Gravimetrically, the AF-ASSB (with 20 μm sulfide SSE) stays lighter at 39.0 mg cm^–2^ and offers 379 Wh kg^–1^. In contrast, the liquid electrolyte-based AFB (E/C = 2.0 g Ah^–1^) weighs 45.0 mg cm^–2^ and provides 329 Wh kg^–1^. Therefore, under realistic swelling conditions, the AF-ASSB maintains roughly a 15% advantage in Wh kg^–1^ and gains about 2.6% in Wh L^–1^. Practically, the geometry influences Wh L^–1^ through thin, stable separators/SSEs and tight stacking, while solvent removal and low-density SSEs provide a substantial Wh kg^–1^ benefit, with even an advantage in Wh L^–1^ once swelling occurs. At the same time, solid–solid interfaces fundamentally change plating and stripping processes in AF-ASSBs because solids cannot flow to fill gaps. Therefore, contact quality, stack pressure, and SE chemo-mechanics control nucleation, void development, and filament suppression. The following sections discuss these challenges and designs related to solid-electrolyte development, interfacial engineering, and CC architecture.

**Figure 57:**
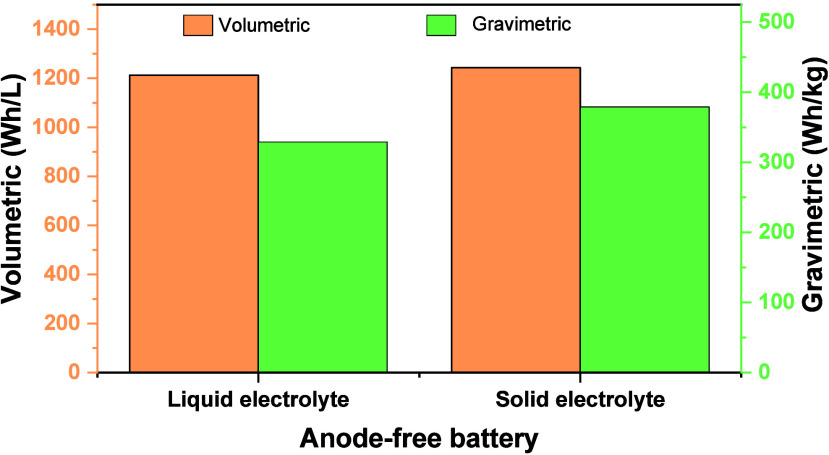
Energy density comparison for anode-free Cu∥NMC811 at 4 mAh cm^–2^ with identical electrodes and CCs; only the electrolyte differs.

### 7.1 Challenges in Anode-Free All-Solid-State Batteries

Although both liquid-electrolyte and solid-electrolyte (SE) AFBs are assembled without active metal on the anode side to maximize energy density, the fundamentals of metal plating dynamics differ between the two media, as shown in Table 13 and Figure 58. In liquids, isotropic ion transport and fluid rewetting generally lead to more uniform initial nucleation on the CC, and the electrolyte can partly “self-heal” interfacial gaps during stripping.^687^ However, unstable, consumptive SEI and current hot spots still cause dendrite formation and progressive loss of metal.^688^ In SEs, the rigid, nonflowable medium results in heterogeneous contact and high local interfacial resistance; nucleation is highly localized, voids remain after stripping, and replating focuses current at asperities, promoting filament growth along grain boundaries or defects at interfaces.^689–691^ Despite higher nominal critical current densities in solids, once filaments start forming, the solid matrix poorly relaxes stress, leading to abrupt failure.^692,693^

**Table 13:** Comparison of Key Factors Governing Anode-Free Metal Plating in Liquid vs Solid Electrolytes

Feature	Liquid electrolyte	Solid-state electrolyte
Li Nucleation	More uniform initially	Highly localized
SEI Stability	Poor (consumptive)	Good (often nonconsumptive)
Contact with the electrolyte	Self-healing	Needs external pressure
Dendrite risk	High	High
Cycle life	Limited by SEI and Li loss	Limited by voids and contact loss
Initial CE	Higher	Lower
Microcrack/void evolution inside plated metal	Cracks filled with electrolyte, and the new metal rapidly passivates, leading to dead-metal formation.	Fresh metal can reconnect under pressure, or continued plating provides self-healing.

**Figure 58:**
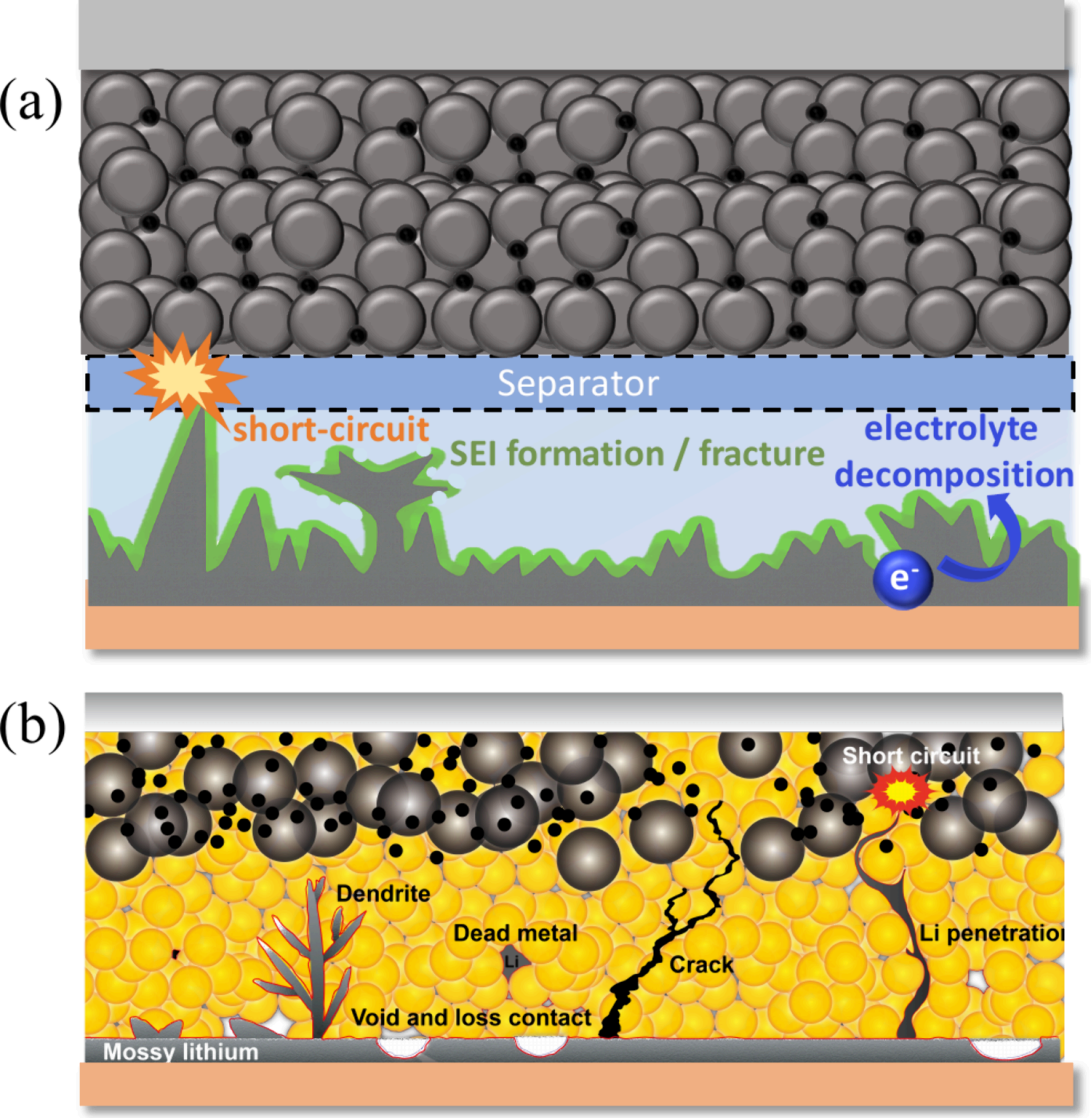
Schematic comparison of metal plating behavior in AFBs with liquid (top) and solid (bottom) electrolytes. In liquids, isotropic ion transport and electrolyte flow promote more uniform initial nucleation but lead to consumptive SEI and dendrite formation. In solids, rigid interfaces cause heterogeneous nucleation, persistent voids after stripping, and defect or grain boundary-guided filament growth; therefore, external pressure and interfacial engineering are essential.

In liquid electrolytes, any microcrack or gap forming within the plated metal is quickly infiltrated by the electrolyte, and the freshly exposed metal surface is immediately passivated by additional SEI formation, especially when the SEI is unstable and keeps regenerating. Consequently, the crack faces become coated with insulative SEI, and the metallic phase can no longer “knit” across the gap, leading instead to dead-metal buildup, which hinders the development of long cycle life of liquid electrolyte-based AFBs. In solid electrolytes, however, the stiff, nonwetting medium may not penetrate into these microcracks, and the fresh plated metal in these protected regions remains fresh metal and unpassivated. As a result, either through stack pressure or through continued plating, the deposited metal can plastically flow and reconnect, thereby enabling intrinsic self-healing, which gives us bright opptuninity to develop long cycle life of solid electrolyte-based AFBs.

These differences, summarized in Figure 58 and Table 13, highlight various limiting mechanisms for AF-ASSBs, such as void-induced contact loss and filamentary short-circuiting issues.^41,694–696^ Therefore, external pressure and interfacial engineering are crucial in AF-ASSBs. Although SEs provide mechanical strength and enhanced safety, AF-ASSBs face specific degradation issues, including interfacial instability, significant volume changes, and uneven ion flux. These problems, shown in Figure 59, remain the main obstacles to long-term performance cycling.

**Figure 59:**
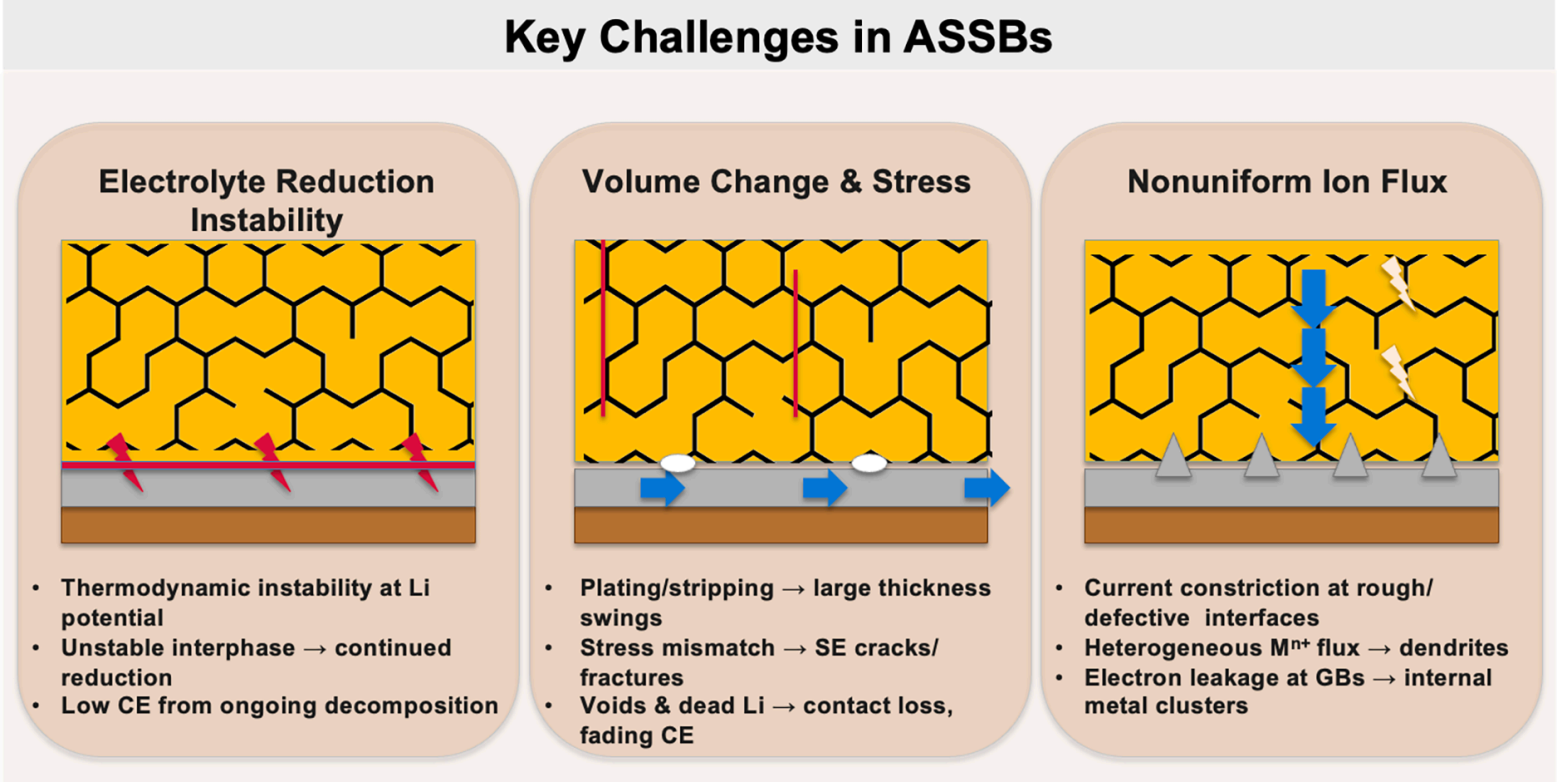
Key challenges in AF-ASSBs that cause dendrite formation and internal metal growth.

#### 7.1.1 Electrolyte Reduction and Unstable Interphases

Several SEs face instability issues in ambient environments,^679,697–702^ but here their interfacial instability is highlighted. Due to the low standard reduction potentials (vs SHE) of Li^+^/Li = −3.04 V, K^+^/K = −2.93 V, and Na^+^/Na = −2.71 V, most electrolytes are thermodynamically prone to reduction upon contact with the metal anode. When in contact with the CC, where metal plating occurs, sulfide-based electrolytes (e.g., LGPS, LPS),^686,703^ and oxide-based electrolytes (e.g., LLZO, NASICONs),^680,698,704,705^ undergo reductive decomposition. These reactions produce interphases that are often electronically conductive but ionically resistive, leading to rapid capacity loss. For instance, LGPS decomposes into Li_15_Ge_4_, a conductive alloy phase that encourages ongoing side reactions. Likewise, NASICON phases containing Ti or Al can produce alloy products that destabilize the electrode–electrolyte interface.^705–711^ As illustrated in Figure 60(a), few SEs fall within the lithium stability window.^706^ Even polymeric and composite electrolytes are susceptible to fragmentation or side reactions, increasing interfacial resistance.^684,712–715^ Unlike conventional lithium-metal cells, AF-ASSBs lack excess lithium to replace irreversibly consumed ions, making electrolyte reduction particularly harmful.

**Figure 60:**
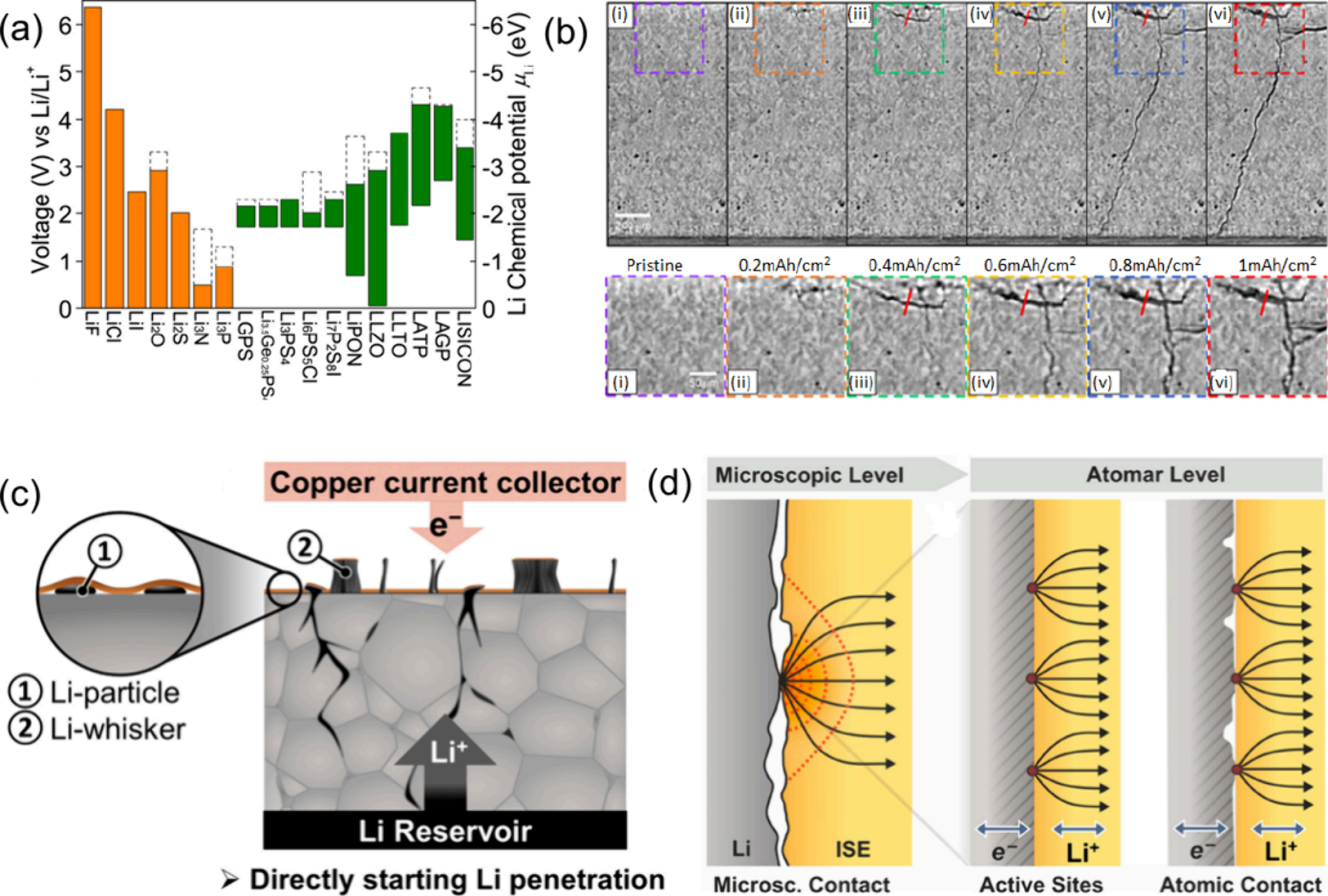
(a) Stability ranges of SE-anode interfaces for various SEs (green bars) and binary lithium compounds (orange bars), along with the interfacial stability range of lithium chemical potential (dashed bars). Reproduced with permission,^706^ Copyright 2016 Royal Society of Chemistry. (b) Crack formation and progression in LPSCl during lithium plating. Reproduced with permission,^720^ Copyright 2021 Springer Nature. (c) Uneven lithium deposition on copper collectors. Reproduced with permission,^718^ Copyright 2019 Elsevier. (d) Concentration of current lines at discrete contact points. Reproduced with permission,^728^ Copyright 2020 American Chemical Society.

#### 7.1.2 Volume Change and Mechanical Fracture

Lithium plating and stripping in anode-free architectures cause severe volume changes.^689,716–719^ For example, depositing 5 mAh cm^–2^ of Li creates a roughly 25 μm thick layer, but uneven deposition can increase this expansion to about 33 μm, as shown in Figure 60(b).^720^ Solid electrolytes, which are rigid and brittle, have difficulty accommodating such expansion, leading to stress concentration, interface debonding, and electrolyte fracture. Thiophosphate-based SEs (with a modulus of 20–40 GPa) can partially buffer stress, while oxides like LLZO (with a modulus over 150 GPa) are highly prone to fracture.^721,722^ X-ray tomography and SEM analyses have shown lateral cracks followed by dendrite-assisted fractures, eventually causing short circuits.^50,723,724^ Volume shrinkage during stripping also creates voids and “dead lithium,” worsening contact loss.^723^

#### 7.1.3 Nonuniform Ion Flux and Dendritic Growth

The nucleation and growth of lithium in AF-ASSBs is controlled by current constriction and interfacial unevenness. Defects on the solid electrolyte (SE) surface, poor wettability with the CC, and grain boundary conduction pathways encourage localized plating. In LLZO, lithium tends to nucleate at structural defects, leading to filamentary growth and isolated islands of metal deposition.^718,725–727^ Likewise, Cu and stainless-steel collectors show limited lithiophilicity, promoting dendritic shapes (Figure 60(c)).^718^ During stripping, uneven dissolution forms voids, increasing resistance and further destabilizing the deposition process. In sulfide-based SEs, void formation during stripping raises interfacial impedance, (Figure 60(d)),^728^ while in oxide SEs, electron leakage at grain boundaries allows isolated Li clusters.^729^ The combination of poor interfacial compatibility and uneven flux is a key failure mode in AF-ASSBs.^729,730^

### 7.2 Designed Strategies in AF-ASSBs

The challenges outlined above require strategies that work across different length scales, ranging from atomic design of solid electrolytes to mesoscale CC architectures. Many studies have reported various designs, as summarized in Table 14. Three main approaches currently drive research: electrolyte design, interfacial engineering, and CC optimization.

**Table 14:** Configuration and Performances of Various AF-ASSBs^753–765^

Battery type	Cell configuration	Electrolytes	Potential window (V)	Capacity (mAh cm^–2^)	Current	Cycle	CR (%)	CE (%)	Ref.
Li-based AF-ASSBs	Ag-In@SUS||NMC7, 1.5, 1.5	Li_6_PS_5_Cl_0.5_Br_0.5_	2.5–4.3	2.0	1C	250	80.2	99.8	^738^
	Alloying on CC								
	Li_2_Te-Cu||NMC811 (lithiated one)	LPSC	3.2–4.3	1.32	0.2C	50	80	99	^746^
	Alloying on CC								
	Mg/W@Cu||NMC811	LPSC	2.8–4.3	1.0	0.33C	150	76	99.5	^753^
	Alloying on CC								
	In@Cu||LFP	Li_6.75_La_3_Zr_1.75_Ta_0.25_O_12_	2.5–3.8	2.30	0.05C	20 at 65 °C	78.9	99.2	^754^
	Alloying on CC								
	Cu||NMC	PEO/LLZTO CPE	2.5–4.3	2.2	0.2 mA cm^–2^	65	41.2	98.8	^683^
	Electrolyte design								
	Ag@Cu||NMC811	3.5 wt % of LPSC + 22.5 wt % of eutectic solution from succinonitrile and LiTFSI (2:1 w/w) + 2 wt % LiF + 2% PVDF	2.5–4.3	1.48	0.1 mA cm^–2^	40	58.1	96.4	^682^
	Electrolyte design								
	Cu||NCA	3LiBH_4_-LiI ||LPSC	2.4–4.2	0.5	0.05 mA cm^–2^	6	55.7	90	^755^
	Electrolyte design								
	SUS||NMC811	Li_6‑*x*_Ag_*x*_PS_5_Cl |Li_6_PS_5_Cl_0.5_Br_0.5_ layer	2.5–4.3	7.0	0.7 mAcm^–2^	50 at 60 °C	95	-	^756^
	Electrolyte design								
	LiPAA-Ag@Cu||LFP	PEO/LLZTO CPE	2–4.2	0.72	0.07 mA cm^–2^	120	90.3	99	^740^
	Artificial SEI on CC								
	MoS_2_@SUS||LiNbO_3_@ NMC622	LPSC	2.5–4.2	2.83	0.2	20	58.9	96.7	^757^
	Artificial SEI on CC								
	C@SUS||NMC532	LPSC	2.5–4.3	2.55	0.1C	300	78.8	-	^758^
	Artificial SEI on CC								
	Ag-C@SUS||NMC333	LLZTO	2.8–4.3	3.15	1 mA cm^–2^	600 at 60 °C	78.3	99.8	^741^
	3D CC								
	Cu-Sn @Cu||NMC811	LPSC	2.5–4.25	2.9	0.1C	150	83.8	99.8	^759^
	3D + alloying on CC								
	SUS||NMC811+Li_2_Cu_0.6_Ni_0.4_O_2_	LPSC	3–4.3	2.57	0.1C	50	82.7	99.5	^760^
	Cathode engineering								
	Ag-C||NCA	LPSC	2.5–4.25	4.4	0.1C	300	89.5	-	^761^
	Alloying on CC + cathode engineering								
	Ag/CB@SUS||Ni_0.83_Mn_0.06_Co_0.11_	LPSC	2.5–4.3	2.72	1 mA cm^–2^	100	43	98	^762^
Na-based AF-ASSBs	Al||NaCrO_2_	Na_0.625_Y_0.25_Zr_0.75_Cl_4.375_||Na_4_B_10_H_10_B_12_H_12_	1.8–3.5	1.0	1 mA cm^–2^	400	70	99.9	^763^
	Electrolyte design								
	Al||Na_3_V_2_(PO_4_)_3_	PEG membrane with LiN_3_ additive, infiltrated with 15 wt % of 1 M NaPF_6_ in G2	2.5–3.8	1.56	0.5C	400	81.6	-	^764^
	Electrolyte design								
	BaTiO_3_/C@Al||Na_3_V_2_(PO_4_)_3_	Na_3_Zr_2_Si_2_PO_12_	2.5–3.8	0.56	0.1C	100	95.5	99	^765^
	Artificial SEI on CC								

#### 7.2.1 Design of the Solid Electrolyte

Solid-electrolyte design lies at the heart of AF-ASSB development. The ideal SE must combine high ionic conductivity (>10^–3^ S cm^–1^), wide electrochemical stability, chemical inertness against Li metal, and mechanical resilience to suppress dendrites while tolerating stress. However, no single class of SEs currently meets all criteria.

Sulfide-based electrolytes, such as Li_10_GeP_2_S_12_ (LGPS) and Li_6_PS_5_Cl, offer conductivities comparable to liquid electrolytes (>10^–2^ S cm^–1^) but suffer from severe instability against Li. Interphases formed at their surfaces are typically electronically conductive, sustaining parasitic reactions. Recent strategies to overcome this include chemical substitution (e.g., Si- or Sb-substituted thiophosphates) that tune the band structure to widen the reduction stability window. Similarly, halogen substitution (e.g., replacing S^2–^ with Cl^–^ or Br^–^) or heteroanion doping (e.g., O/S mixing) have improved stability windows.^18,678,680,704,731^ Composite designs such as garnet infiltrated into a 3D conductive framework (Figure 61(a)),^732^ or 3D micropatterned garnet electrolyte using laser processing (Figure 61(b)),^733^ also show promise, offering both ion transport and structural stability. Oxide electrolytes such as LLZO exhibit superior chemical stability against Li, but their brittleness leads to cracking under volume fluctuations. Strategies here focus on tailoring grain boundaries, introducing dopants (Al, Ta) to stabilize cubic LLZO, or embedding the oxide into hybrid multilayer designs. Halide electrolytes (e.g., Li_3_YCl_6_, Li_3_InCl_6_) are emerging as promising options, balancing conductivity and ease of processing. Based on this advantage, multilayer SEs further combine chemistries, such as pairing sulfide with halide solid electrolyte, to achieve >94% initial CE compared to 70.5% with sulfide alone (Figure 61(c)).^734^

**Figure 61:**
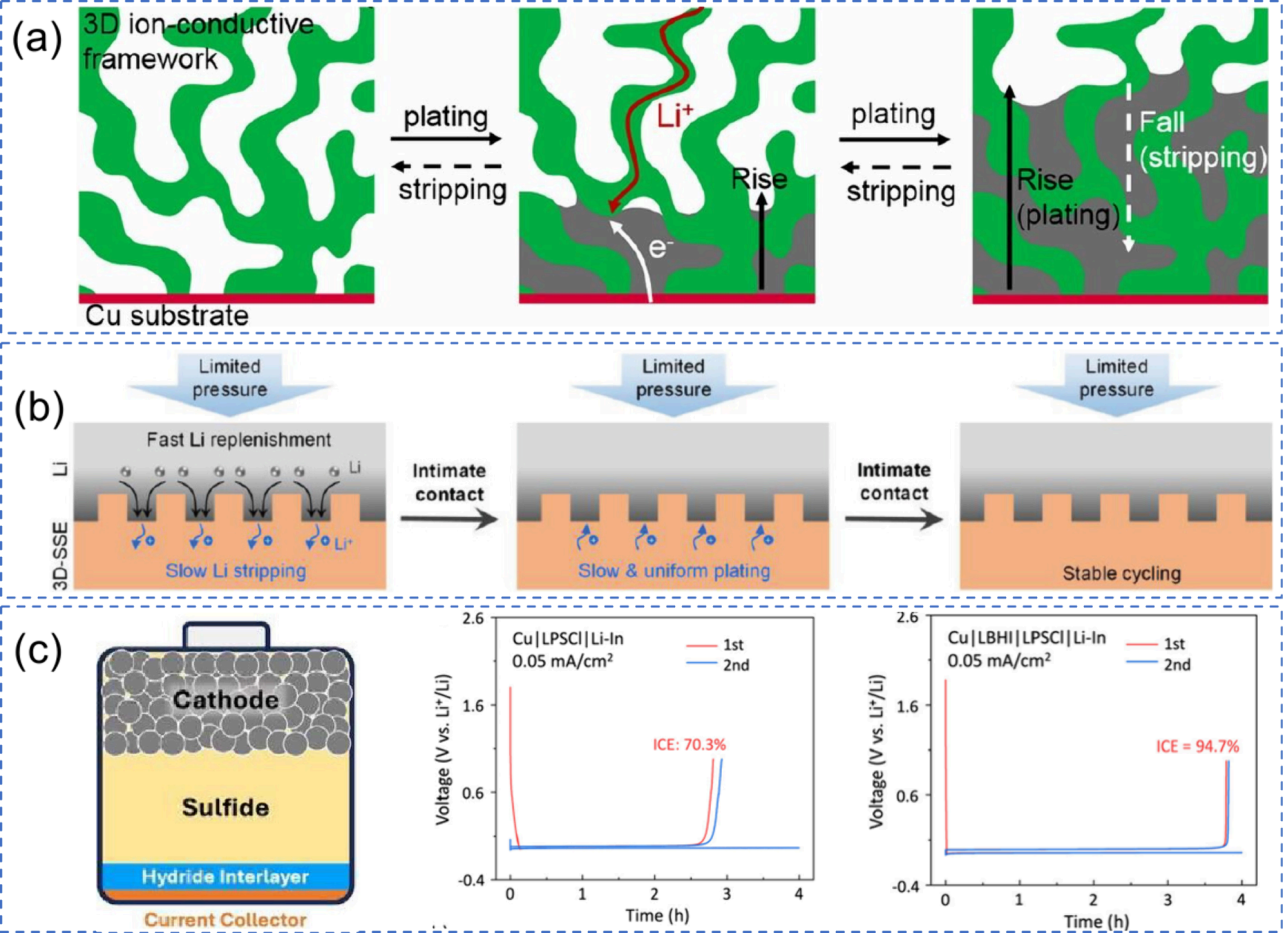
(a) Lithium deposition and growth process within a three-dimensional SE structure. Reproduced with permission,^732^ Copyright 2018 The Author(s) published by National Academy of Sciences. (b) The 3D-SE interface demonstrates reduced local current density and enhanced mechanical stress distribution, mitigating morphological degradation at operational interfaces. Reproduced with permission,^733^ Copyright 2021 Wiley-VCH. (c) Hydride interlayer inserted between the Cu CC and sulfide SSE promotes intimate contact and rapid interfacial Li transport. Reproduced with permission,^734^ Copyright 2024 American Chemical Society.

Polymer-based SEs, such as PEO or PVDF-HFP composites, offer mechanical flexibility and can absorb stress; however, they are still limited by conductivity and interfacial reactivity.^171,657^ Combining inorganic and polymeric phases leverages the benefits of both types. For example, sulfide–polymer composites reduce reduction by encapsulating sulfide particles in flexible matrices, while oxide–halide hybrids prevent fracture through ductile secondary phases.^15,715^ Such designs are especially promising for scaling pouch cells where mechanical and electrochemical stability need to coexist.

Despite progress, SEs still face trade-offs among conductivity, interfacial resistance, and mechanical brittleness. Developing kinetically stable, chemically compatible SEs that promote uniform metal nucleation remains an urgent priority. Electrolyte design in AF-ASSBs must strike a balance between conductivity, chemical stability, and mechanical properties. The trend is toward multifunctional, hybrid, or multilayer electrolytes that combine the advantages of different classes while compensating for their weaknesses.

#### 7.2.2 Interfacial Engineering

Because solids cannot reflow to fill voids, interlayers that improve wetting, reduce nucleation overpotential, and stay compliant under stack pressure are essential in AF-ASSBs. Effective interfaces reduce nucleation overpotential, homogenize current distribution, and buffer mechanical stress. The SE-CC interface remains the most vulnerable connection in AF-ASSBs. Strategies here focus on stabilizing the interface chemically, mechanically, and electrochemically.

Thin protective coatings, such as LiNbO_3_, Li_3_PO_4_, or LiF-based layers, are placed between SEs and the CC to prevent reductive decomposition.^706–708,735^ Self-forming interphases, created by electrolyte additives like LiNO_3_, can also produce Li_3_N-rich layers that help stabilize cycling. Interlayers such as Ag, Au, Sb, Bi, and Zn have been shown to form an alloy with the plated metal, improving interfacial wetting, lowering nucleation barriers, and promoting uniform deposition.^736–739^ Ag-C composites, where silver dissolves and carbon provides elasticity, have enabled pouch cells with over 1000 cycles at an energy density of more than 900 Wh L^–1^ (Figure 62(a)).^690^ However, using elements like Au and Ag raises concerns about cost and scalability. Polymer-based layers (for example, PEO-based films, lithiated poly­(acrylic acid), PAA) accommodate stress while maintaining ionic conductivity (Figure 62(b)).^740^ Thin Ag-modified Cu foils reduce interfacial resistance and stabilize cycling, while more advanced designs, such as TiN nanotubes filled with Ag-C, combine mechanical buffering with chemical lithiophilicity (Figure 62(c)).^741^

**Figure 62:**
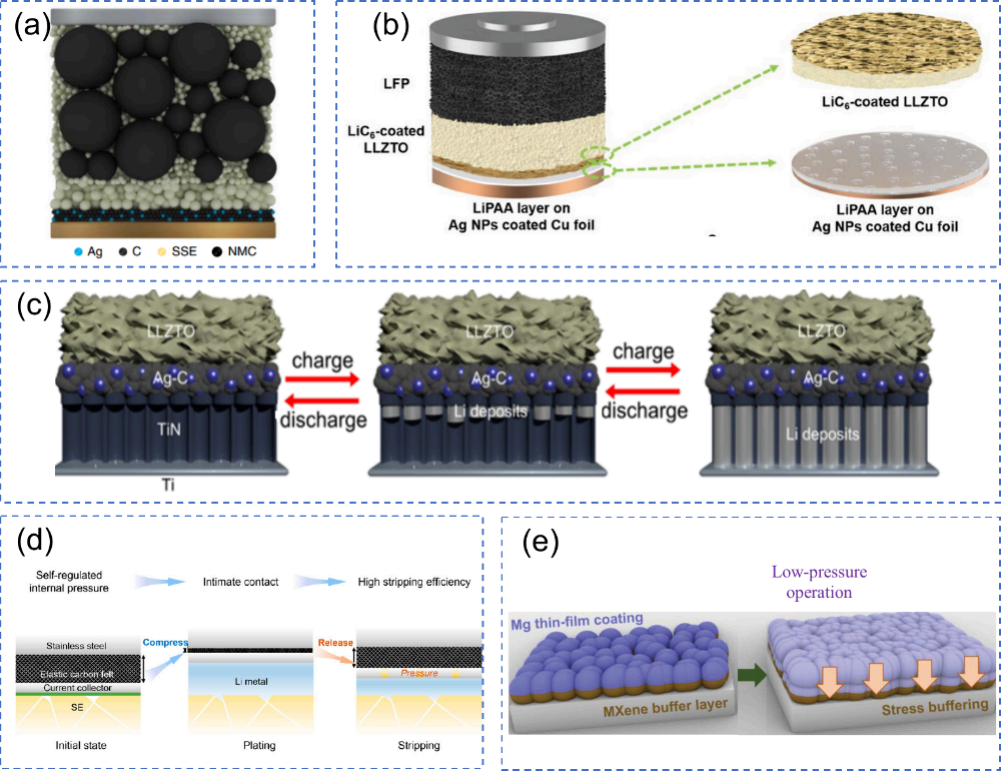
(a) Diagram of an AF-ASSLB featuring a high-capacity NMC cathode, SE, and Ag-C anode layer. Reproduced with permission.^690^ Copyright 2020 Springer Nature. (b) configuration of the LiPAA@Ag@Cu||LiC_6_-LLZTO||LFP AF-ASSB. Reproduced with permission,^740^ Copyright 2023 Wiley-VCH GmbH. (c) Diagram of the TiN NT-incorporated AF-ASSB during charge/discharge. Reproduced with permission,^741^ Copyright 2023 Wiley-VCH GmbH. (d) Illustration of the AF-ASSB with self-regulated pressure during initial states, plating, and stripping. Reproduced with permission,^361^ Copyright 2023 American Chemical Society. (e) Lithiation schematic of the MXene/Mg double-layer promoting strong SE-electrode contact at low pressure. Reproduced with permission,^743^ Copyright 2023 Wiley-VCH GmbH.

Emerging designs also focus on regulating stack pressure and emphasize self-healing or dynamic interphases. For example, glassy lithium–boron oxides can slightly flow under stress, filling voids and cracks that develop during stripping.^682,697,742^ Catalytically active interlayers, such as carbon-felt elastic layer (Figure 62(d)),^361^ and Mg-Ti_3_C_2_T_*x*_ MXenes (Figure 62(e)),^743^ control lithium nucleation and encourage columnar deposition, reducing dendrite growth, and maintain consistent interfacial contact even under high areal capacities.^744^ Such designs address the inevitable stress fluctuations from plating and stripping, preventing interfacial delamination and void formation.

#### 7.2.3 Current Collector Architecture

AF-ASSBs require engineered, lithiophilic and/or 3D collectors to create uniform nucleation and evenly distribute current, thereby minimizing void growth during stripping. Bare Cu, while widely used, is poorly metallophilic, leading to inhomogeneous nucleation and dendritic growth. Surface modification with metallophilic metals (Ag, In, Zn) or compounds (Li_2_Te, LiF) significantly reduces nucleation overpotential. For example, In-modified Ag–Cu collectors achieved >250 stable cycles with CE > 99.8% (Figure 63(a)),^738^ while carbon-paper (CP) composites with dual ionic/electronic conductivity provided abundant nucleation sites and >5000 cycles at high areal capacity (Figure 63(b, c)).^745^ These modifications in general have been demonstrated to reduce overpotential and improve CE in thin-film AF-ASSBs. Advanced strategies also focus on incorporating catalytic or self-healing coatings that dynamically stabilize the interface. Cryo-FIB imaging of Li_2_Te-modified Cu revealed smooth, uniform deposition without filament formation, underscoring the importance of tailored surface chemistry (Figure 63(d, e)).^746^

**Figure 63:**
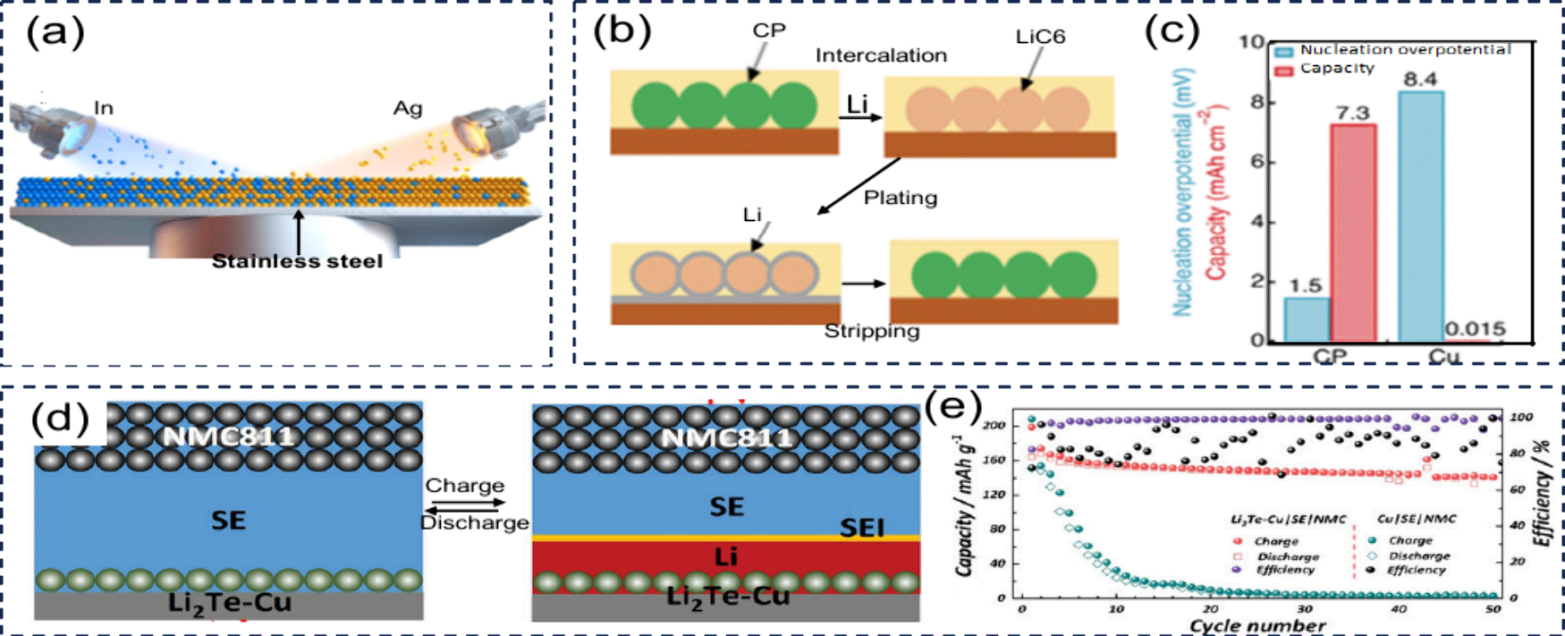
(a) Illustration of Ag-In coated stainless-steel CC fabricated via sputtering method. Reproduced with permission,^738^ Copyright 2024 Elsevier (b) Working mechanism in CP CC (c) Comparison of nucleation overpotential and areal capacity of CP and Cu CCs. (b, c) Reproduced with permission,^745^ Copyright 2023 The Royal Society of Chemistry. (d) Schematic diagram of Li_2_Te formation on Cu CC. (e) Cycling performance of Li_2_Te-Cu||SE||NMC and Cu||SE||NMC cells. (d, e) Reproduced with permission,^746^ Copyright 2023 Wiley-VCH GmbH.

Porous Cu scaffolds, carbon frameworks, and patterned CCs can distribute current more evenly and accommodate volume changes.^747–750^ For example, 3D Cu hosts coated with lithiophilic oxides promote uniform lithium infiltration, delaying short circuits and extending cycle life in Li-based AF-ASSBs. In Zn- and Al-based AF-ASSBs, catalytic 3D hosts also speed up sluggish multivalent ion nucleation.^751,752^ Although nanoscale and 3D engineered collectors enhance performance in laboratory cells, scalable manufacturing techniques are still limited. Methods such as electrospinning, templated electrodeposition, and additive manufacturing show promise but require modification for high-throughput roll-to-roll processes to become commercially practical. The universal design principle is clear: CCs must evolve from passive substrates into active regulators of nucleation and interfacial chemistry.

While this section discussed AF-ASSBs across alkali and multivalent chemistries, most thoroughly validated demonstrations to date remain Li-based, with Na-based AF-ASSBs only just emerging and still showing limited data. What is encouraging is that the core strategies discussed, solid-electrolyte design, interfacial engineering, and CC architecture, are chemistry-agnostic and directly applicable as a framework for Na, K, Zn, Mg, and Al systems. At the same time, solid–solid interfaces create failure modes (contact loss, voiding, filamentary shorting) that are common but manifest differently among ions and electrolytes, emphasizing the need for coupled chemo-mechanical metrics and pressure-aware testing in any AF-ASSB chemistry. Early Na-based AF-ASSB studies highlight this transferability and reveal unique interphase and transport constraints that will influence material selection and stack design moving forward. In short, Li-based AF-ASSBs now set the standards for performance and diagnostics; the immediate goal is to adapt that approach, focusing on SE compatibility, engineered interphases that remain permeable under stress, and scalable, metallophilic/3D collectors, to extend these methods to Na/K and multivalent systems where chemistry-specific interphase rules, pressure windows, and scalable collector/coating strategies are essential.

## 8 Summary

AFBs represent a transformative and disruptive technology in the field of energy storage, offering the potential to redefine the metrics of energy density, manufacturing simplicity, and cost-effectiveness. By eliminating the conventional preformed anode, AFBs achieve a significant reduction in inactive material usage, improving volumetric and gravimetric energy densities. This streamlined architecture also aligns with global efforts to reduce reliance on resource-intensive materials and transition toward more sustainable battery technologies. The scientific advancements in AFBs have shed light on the complexities of their operation. The fundamental processes, plating and stripping dynamics, nucleation and growth behavior, electrolyte decomposition, and dendrite formation, are now better understood, thanks to concerted research efforts. Additionally, challenges such as dead metal formation, heterogeneous plating, and metal inventory losses have been rigorously studied, providing valuable insights into failure mechanisms. These foundational studies have driven innovations across multiple research fronts, including electrolyte engineering, interfacial stabilization, advanced CC designs, and optimized cathode materials. Strategies to address these challenges have undergone significant evolution. Electrolyte engineering, for instance, has been pivotal in stabilizing the metal–electrolyte interface and suppressing dendrite growth. Functional additives and novel solvent systems have been developed to improve ionic conductivity and promote the formation of stable SEIs. Similarly, interfacial engineering approaches, such as ultrathin protective coatings and self-healing materials, have mitigated uneven plating and enhanced cycling stability. Advances in CC designs, particularly three-dimensional and porous architectures, have also contributed to uniform metal plating and reduced localized stress. These strategies have collectively enabled significant progress in addressing the core challenges of AFB technology. Beyond these innovations, AFBs have emerged as a powerful research tool for probing fundamental interfacial phenomena. Techniques for visualizing metal plating, quantifying dead metal, and analyzing irreversible CE have provided unprecedented insights into the dynamics of metal anodes. These studies have not only deepened our understanding of AFB operation but also informed the development of more effective materials and designs. The ability to decouple interfacial processes and systematically evaluate their contributions to overall performance underscores the value of AFBs as a platform for advancing battery science. Critical parameters such as CE, electrolyte volume, external pressure, and cycling protocols play a crucial role in determining the performance and reliability of AFBs. High CE minimizes irreversible lithium loss, while optimized electrolyte volumes ensure stable ion transport without excessive side reactions. Controlling external pressure and temperature helps mitigate dendrite growth and mechanical degradation, while carefully designed cycling protocols enhance long-term stability and energy retention. These parameters are essential for achieving the high performance and safety standards required for commercial applications. Despite these advances, significant technical hurdles remain. The highly reactive nature of metal anodes in AFB systems leads to parasitic reactions with the electrolyte, adversely affecting cycling performance and long-term stability. The lack of a preformed anode exacerbates issues related to nonuniform plating and dendrite formation, posing risks to both efficiency and safety. These challenges necessitate continued exploration of strategies to enhance the reliability and performance of AFBs, such as the incorporation of solid-state electrolytes and the development of hybrid designs that combine the best attributes of different battery chemistries. Beyond lithium, the application of the anode-free concept to alternative metals like sodium, potassium, zinc, magnesium, and aluminum has opened new research frontiers. Each system presents unique advantages and challenges, such as cost-effectiveness, abundance, or compatibility with existing technologies, making them promising candidates for diverse applications. Moreover, the integration of solid-state configurations in AFBs holds immense potential to address safety concerns while delivering enhanced energy densities. In conclusion, AFBs are positioned as a game-changing technology for next-generation energy storage. Their promise lies not only in their superior performance metrics but also in their alignment with sustainable and cost-efficient manufacturing goals. The combination of innovative strategies, advanced characterization techniques, and critical parameter optimization has brought us closer to overcoming the inherent challenges of AFBs. While the journey toward commercialization involves overcoming significant obstacles, the combination of interdisciplinary research, material innovations, and strategic collaborations will undoubtedly accelerate the development of robust, scalable AFB systems. Realizing the full potential of AFBs will not only meet the growing energy demands of modern society but also contribute meaningfully to global sustainability initiatives.

## 9 Outlook

AFBs are poised to revolutionize the landscape of electrochemical energy storage by offering ultrahigh energy density, simplified design, and reduced manufacturing complexity. However, the translation of these promising systems into practical applications remains hindered by a constellation of challenges that span across electrochemical reversibility, interfacial dynamics, structural integrity, and scalable manufacturability. In this outlook, we delineate five interconnected research areas, as demonstrated in Figure 64, which monitor the progression, liquid-based AFBs, AF-ASSBs, multivalent AFBs, and scaling and commercialization. These areas, together, chart a strategic roadmap toward overcoming the intrinsic limitations of AFBs and realizing their full potential in advanced energy systems.

**Figure 64:**
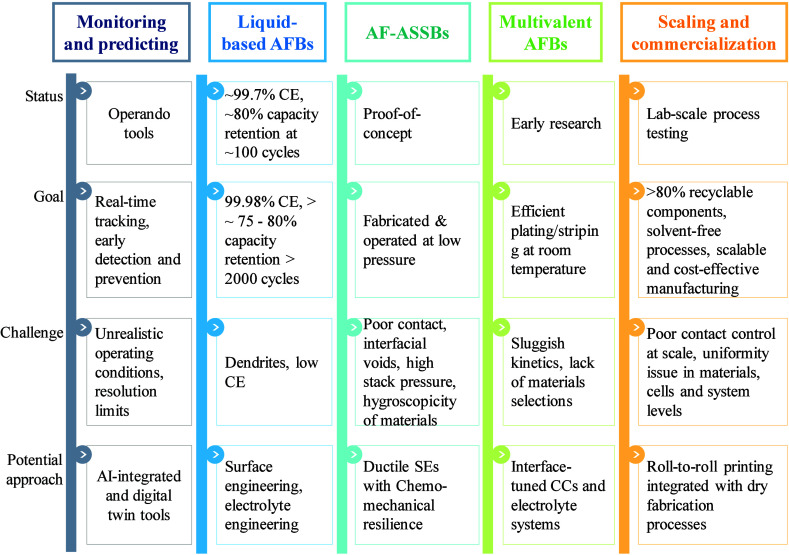
Future opportunities and transformative perspectives in AFBs.

### 9.1 Monitoring and Predicting: Toward Intelligent Diagnosis, Control, and Automation

A fundamental prerequisite for advancing AFB technology lies in the ability to precisely monitor, predict, and interpret the dynamic physicochemical transformations that occur during cycling. These include lithium or multivalent metal nucleation and growth, interfacial evolution, void formation, and electrolyte degradation, phenomena that directly influence CE, reversibility, and lifetime. Although considerable progress has been made with operando characterization tools, such as synchrotron-based X-ray tomography, cryo-TEM, solid-state NMR, optical microscopy, and EIS, significant limitations persist. These include restricted spatial and temporal resolution, beam-induced damage, incompatibility with sealed or stacked formats, and difficulty distinguishing active from inactive interfaces. To overcome these hurdles, the next frontier lies in the convergence of advanced in situ diagnostics with artificial intelligence (AI) driven data analytics and digital twin platforms. AI-integrated multimodal analysis can extract high-value features from large, noisy data sets, enabling real-time interpretation and early anomaly detection. For instance, machine learning algorithms can deconvolute overlapping spectral signatures, identify latent failure modes, and correlate structural or compositional evolution with electrochemical signals. Digital twin technology, which creates a virtual replica of the physical battery system, enables predictive simulations of battery behavior under various conditions, offering a proactive approach to degradation management and lifetime extension. In parallel, embedding microscale sensing units, such as strain gauges, voltage sensors, pressure transducers, and thermal probes within the cell stack, will enable localized, real-time monitoring. These sensors can feed data into adaptive battery management systems (BMS) that automatically adjust current densities, cutoff voltages, or operating temperatures in response to early degradation signals. With closed-loop feedback control, the BMS can dynamically suppress incipient failure mechanisms before they propagate. The long-term vision is to develop smart AFBs that integrate self-monitoring, self-predicting, and self-regulating capabilities, ensuring safer, longer-lasting operation. Such systems will be instrumental in bridging the gap between laboratory-scale insights and commercially relevant cell designs. Meanwhile, the AI-autonomous laboratory, integrated with computational simulation, generative AI, and experimental automation for anode-free battery (AFB) development, enables rapid exploration of complex chemical and design spaces beyond human capability. It leverages active learning to extract maximum insight from minimal experiments, while robotic operation ensures consistent and reproducible experimental conditions. AI-driven adaptive control continuously refines experimental parameters based on real-time data, dramatically shortening R&D cycles from years to weeks. By seamlessly combining human creativity with machine precision and scalability, the AI-autonomous laboratory will transform the development of AFBs from a traditional empirical process into a self-driving, intelligent cycle of continuous discovery and innovation.

### 9.2 Liquid-Based AFBs: Toward High Reversibility and Long Cycle Life

Liquid electrolyte-based AFBs currently represent the most mature configuration among AFB technologies, having demonstrated >99.7% CE and ∼80% capacity retention over ∼100 cycles. Nonetheless, these systems remain plagued by dendritic metal growth, unstable interphases, dead metal accumulation, and electrolyte depletion, particularly under high charging current density or lean electrolyte conditions.^1000^ The root cause of many of these challenges lies in the heterogeneous ion flux and uncontrolled nucleation at the bare CC interface, which promotes nonuniform deposition and localized stress buildup. Recent advancements have focused on surface engineering and electrolyte optimization to mitigate these issues. The application of metallophilic seed layers or coatings (e.g., Ag, ZnO, AlN) significantly reduces the nucleation overpotential, promoting uniform metal deposition. Additionally, artificial SEIs with adaptive capability, designed with high mechanical resilience and ionic conductivity, can protect the plated metal from parasitic reactions. On the electrolyte side, the development of localized high-concentration electrolytes (LHCEs), fluorinated ether solvents, and multifunctional additives has shown promise in suppressing dendrite formation and improving interfacial stability. Target performance metrics for next-generation liquid-based AFBs include a CE of ≥99.98%, >80% capacity retention over >1000 cycles, and compatibility with high-loading cathodes (>4 mAh cm^–2^). These advances, combined with mechanistic insights, AI-guided material design, and advanced manufacturing, can position liquid-based AFBs as viable contenders for high-power and high-energy applications, such as electric aviation.

### 9.3 AF-ASSBs: Toward Safer and Low-Pressure Operation

AF-ASSBs offer a compelling pathway toward intrinsically safe, high-energy storage systems by eliminating the flammable liquid electrolyte. Moreover, their compatibility with high-voltage cathodes and thermal stability positions them favorably for use in electric vehicles and aerospace technologies. However, their practical deployment is constrained by several key issues: (i) poor interfacial contact between the solid-state electrolyte (SSE) and the plated metal, (ii) formation of voids and interfacial gaps during cycling, and (iii) the necessity for high stack pressure (>10 MPa) to maintain ionic continuity, all of which contribute to capacity fade and poor cycle life. Recent studies have introduced ductile SSEs, such as sulfide-based electrolytes (e.g., Li_6_PS_5_Cl) blended with elastomeric polymers, to provide chemo-mechanical adaptability. These soft-solid composites can maintain intimate contact even during the volumetric expansion/contraction associated with metal plating and stripping. Additionally, interface engineering strategies, including the use of alloy-forming interlayers, metallophilic coatings, and active buffer layers, can lower the nucleation barrier and prevent the formation of “dead” metal regions. The ultimate goal is to develop AF-ASSBs capable of operating under ambient or near-ambient pressure conditions, with an initial CE > 90%, and a cycle life exceeding 2000 cycles. Achieving this will require a multidisciplinary convergence of thin-film deposition, solid-state interface modeling, and mechanical–electrochemical coupling simulations, supported by advanced in situ diagnostic tools. As research moves from coin-cell configurations to pouch and prismatic formats, mechanical integration strategies such as stackable compliant layers or pressure-distributing interlayers will become increasingly vital. A completely new manufacture processes for AF-ASSBs is needed compared to those for liquid-based AFBs.

### 9.4 Multivalent AFBs: Unlocking the Potential of beyond-Lithium Chemistries

Multivalent AFBs, based on metals such as magnesium, aluminum, and zinc, promise extraordinary volumetric capacities, enhanced safety, and low raw material cost. For instance, aluminum has a theoretical volumetric capacity of 8046 mAh cm^–3^, nearly twice that of lithium. Furthermore, these metals exhibit lower reactivity with air and moisture, offering potential benefits in terms of handling and safety. However, the anode-free configuration exacerbates the intrinsic limitations of multivalent systems, which include less available cathode chemistry, passivating oxide layers on metals, sluggish ion kinetics, and electrolyte incompatibility. Addressing these issues necessitates breakthroughs in electrolyte chemistry, interfacial science, and materials engineering. Chloride-based electrolytes, hydride-stabilized ionic liquids, and complexing agents have been investigated to mitigate surface passivation and facilitate reversible plating and stripping. Simultaneously, the development of artificial interphases incorporating multivalent-conducting polymers or inorganic layers (e.g., AlN, Mg_3_N_2_) can support ion transport while suppressing side reactions. On the cathode side, the use of metal-containing frameworks (e.g., Al-doped oxides, Prussian Blue analogs) can provide sacrificial metal reservoirs to compensate for early cycle irreversibility. Although multivalent AFBs are currently in early research stages, a realistic near-term target includes achieving initial CE > 90% and stable cycling over several hundred cycles in prototype cells. Long-term success will depend on the integration of coordination chemistry, quantum-level interfacial modeling, and electrolyte–electrode interface diagnostics. These systems may be especially attractive for grid-scale energy storage where energy density is less critical than cost, safety, and sustainability.

### 9.5 Scaling and Commercialization: Toward Green, High-Throughput Manufacturing

Beyond the laboratory, the scalable and sustainable manufacturing of AFBs is crucial for commercial adoption. The absence of a metal foil anode presents a unique advantage: AFBs inherently have a simplified architecture, enabling streamlined fabrication processes. Nevertheless, achieving consistent plating behavior, ensuring reproducibility, and controlling interfacial quality across large-format cells remain major obstacles. Emerging manufacturing strategies include roll-to-roll printing of artificial SEIs, dry electrode fabrication, and solvent-free processing methods that eliminate the use of toxic or volatile chemicals. These innovations not only reduce production costs and cycle time, but also align with green chemistry principles and regulatory compliance. Further, the incorporation of recyclable materials, including reusable CCs, binder-free electrodes, and low-waste separators, contributes to improving the sustainability profile of AFBs. Advanced manufacturers, powered by machine learning and digital twins, can ensure real-time quality assurance by detecting or predicting misalignments, defects, nonuniformity, inconsistencies, and variation evolution during production. In parallel, lifecycle assessment (LCA)^1000^ and techno-economic analysis (TEA) frameworks must be adopted to quantify environmental impact and economic feasibility at scale. As a benchmark, achieving >80% recyclability of components and full solvent-free processing represents a realistic and impactful goal for near-future commercialization.

Overall, the journey toward commercially viable AFBs will not be defined by incremental improvements, but by transformative, interdisciplinary innovation. Whether addressing dendrite suppression in liquid-based systems, interfacial stabilization in AF-ASSBs, compatibility issues in multivalent chemistries, or sustainable scale-up strategies, the key lies in integrating knowledge from materials science, electrochemistry, mechanical engineering, data science, and manufacturing. Future AFB platforms must feature intelligent interfaces capable of regulating ion flux, self-healing during cycling, and dynamically adjusting to external conditions via feedback from embedded diagnostics. Simultaneously, the implementation of AI-assisted materials discovery, automated cell assembly, and adaptive BMS algorithms will be pivotal in optimizing performance and safety. These systems, underpinned by real-time monitoring and robust design principles, can unlock unprecedented energy densities while minimizing environmental burden. Ultimately, the future of AFBs is not speculative, it is tangible, rapidly evolving, and firmly grounded in multidisciplinary science. The successful realization of AFB technology will be a hallmark of next-generation energy storage systems, enabling a sustainable and electrified global future.

## References

[1] Goodenough J. B., Park K.-S. (2013). The Li-ion rechargeable battery: a perspective. J. Am. Chem. Soc..

[2] Dunn B., Kamath H., Tarascon J.-M. (2011). Electrical
energy storage for the
grid: a battery of choices. Science.

[3] Li J., Manthiram A. (2019). A comprehensive
analysis of the interphasial and structural
evolution over long-term cycling of ultrahigh-nickel cathodes in lithium-ion
batteries. Adv. Energy Mater..

[4] Louli A., Eldesoky A., Weber R., Genovese M., Coon M., deGooyer J., Deng Z., White R., Lee J., Rodgers T. (2020). Diagnosing
and correcting anode-free cell failure via
electrolyte and morphological analysis. Nat.
Energy.

[5] Hagen M., Hanselmann D., Ahlbrecht K., Maça R., Gerber D., Tübke J. (2015). Lithium-sulfur
cells: the gap between
the state-of-the-art and the requirements for high energy battery
cells. Adv. Energy Mater..

[6] Dong L., Zhong S., Zhang S., Yuan B., Liu J., Xie H., Zhang C., Liu Y., Yang C., Han J. (2023). Toward practical anode-free
lithium pouch batteries. Energy Environ. Sci..

[7] Wu J., Cao Y., Zhao H., Mao J., Guo Z. (2019). The critical role of
carbon in marrying silicon and graphite anodes for high-energy lithium-ion
batteries. Carbon Energy.

[8] Chae S., Choi S. H., Kim N., Sung J., Cho J. (2020). Integration
of graphite and silicon anodes for the commercialization of high-energy
lithium-ion batteries. Angew. Chem., Int. Ed..

[9] Liu L., Yin Y.-X., Li J.-Y., Li N.-W., Zeng X.-X., Ye H., Guo Y.-G., Wan L.-J. (2017). Free-standing hollow carbon fibers
as high-capacity containers for stable lithium metal anodes. Joule.

[10] Vrankovic D., Graczyk-Zajac M., Kalcher C., Rohrer J., Becker M., Stabler C., Trykowski G., Albe K., Riedel R. (2017). Highly porous
silicon embedded in a ceramic matrix: a stable high-capacity electrode
for Li-ion batteries. ACS Nano.

[11] Fu K. K., Gong Y., Hitz G. T., McOwen D. W., Li Y., Xu S., Wen Y., Zhang L., Wang C., Pastel G. (2017). Three-dimensional
bilayer garnet solid electrolyte based high energy
density lithium metal-sulfur batteries. Energy
Environ. Sci..

[12] Lin D., Liu Y., Liang Z., Lee H.-W., Sun J., Wang H., Yan K., Xie J., Cui Y. (2016). Layered reduced graphene oxide with
nanoscale interlayer gaps as a stable host for lithium metal anodes. Nat. Nanotechnol..

[13] Fang C., Wang X., Meng Y. S. (2019). Key issues
hindering a practical
lithium-metal anode. Trends Chem..

[14] Qian J., Henderson W. A., Xu W., Bhattacharya P., Engelhard M., Borodin O., Zhang J.-G. (2015). High rate
and stable
cycling of lithium metal anode. Nat. Commun..

[15] Bai P., Li J., Brushett F. R., Bazant M. Z. (2016). Transition of lithium growth mechanisms
in liquid electrolytes. Energy Environ. Sci..

[16] Yang H., Guo C., Naveed A., Lei J., Yang J., Nuli Y., Wang J. (2018). Recent progress and
perspective on lithium metal anode protection. Energy Storage Mater..

[17] Wang H., Liu Y., Li Y., Cui Y. (2019). Lithium metal
anode materials design:
interphase and host. Electrochem. Energy Rev..

[18] Xiang J., Yang L., Yuan L., Yuan K., Zhang Y., Huang Y., Lin J., Pan F., Huang Y. (2019). Alkali-metal
anodes: from lab to market. Joule.

[19] Liu L., Yin Y. X., Li J. Y., Wang S. H., Guo Y. G., Wan L. J. (2018). Uniform
lithium nucleation/growth induced by lightweight
nitrogen-doped graphitic carbon foams for high-performance lithium
metal anodes. Adv. Mater..

[20] Liang Y., Dong H., Aurbach D., Yao Y. (2020). Current status and
future directions of multivalent metal-ion batteries. Nat. Energy.

[21] Zhang X., Lv R., Tang W., Li G., Wang A., Dong A., Liu X., Luo J. (2020). Challenges
and opportunities for multivalent metal
anodes in rechargeable batteries. Adv. Funct.
Mater..

[22] Mahmood A., Bai Z., Wang T., Lei Y., Wang S., Sun B., Khan H., Khan K., Sun K., Wang G. (2025). Enabling high-performance
multivalent metal-ion batteries: current advances and future prospects. Chem. Soc. Rev..

[23] Xu P., Huang F., Sun Y., Lei Y., Cao X., Liang S., Fang G. (2024). Anode-free alkali metal
batteries:
from laboratory to practicability. Adv. Funct.
Mater..

[24] Wang H., Yu D., Kuang C., Cheng L., Li W., Feng X., Zhang Z., Zhang X., Zhang Y. (2019). Alkali metal anodes
for rechargeable batteries. Chem..

[25] Mao C., Dong J., Li J., Zhai X., Ma J., Luan S., Shen X., Wang Y., Zhang P., Sun H. (2025). Toward
Practical All-Solid-State Batteries: Current
Status of Functional Binders. Adv. Mater..

[26] Hong B., Gao L., Li C., Lai G., Zhu J., Huang D., Zuo Y., Yin W., Sun M., Zhao S. (2025). All-solid-state
batteries designed for operation under extreme cold conditions. Nat. Commun..

[27] Xie Z., Wu Z., An X., Yue X., Wang J., Abudula A., Guan G. (2020). Anode-free rechargeable lithium metal
batteries: progress and prospects. Energy Storage
Mater..

[28] Qian J., Adams B. D., Zheng J., Xu W., Henderson W. A., Wang J., Bowden M. E., Xu S., Hu J., Zhang J. G. (2016). Anode-free rechargeable lithium metal batteries. Adv. Funct. Mater..

[29] Kwon Y., Svirinovsky-Arbeli A., Hestenes J. C., Botero P. J. B., Corpus K. R. M., Lepucki P., Pecher O., Marbella L. E. (2024). Elucidating the
role of cathode identity: Voltage-dependent reversibility of anode-free
batteries. Chem..

[30] Huo S., Wang L., Su B., Xue W., Wang Y., Zhang H., Li M., Qiu J., Xu H., He X. (2024). Anode-Free Li Metal Batteries: Feasibility Analysis
and Practical
Strategy. Adv. Mater..

[31] Petersen D., Gronenberg M., Lener G., Leiva E., Luque G. L., Rostami S., Paolella A., Hwang B. J., Adelung R., Abdollahifar M. (2024). Anode-free post-Li metal batteries. Mater. Horiz..

[32] Nanda S., Gupta A., Manthiram A. (2021). Anode-free full cells: a pathway
to high-energy density lithium-metal batteries. Adv. Energy Mater..

[33] Yao W., Zou P., Wang M., Zhan H., Kang F., Yang C. (2021). Design principle,
optimization strategies, and future perspectives of anode-free configurations
for high-energy rechargeable metal batteries. Electrochem. Energy Rev..

[34] Jo C.-H., Sohn K.-S., Myung S.-T. (2023). Feasible approaches
for anode-free
lithium-metal batteries as next generation energy storage systems. Energy Storage Mater..

[35] Zhou Y., Wang P., Wang K., Fang X., Li W., Nai J., Liu Y., Wang Y., Zou S., Yuan H. (2025). Developing High-Performance Anode-Free Lithium Batteries:
Challenges,
Strategies, and Opportunities. Adv. Funct. Mater..

[36] Liu P., Jin T., Jiao L. (2025). Toward Anode-Free
Sodium Metal Batteries: Engineering
Metal Utilization from Excess to Zero. Angew.
Chem..

[37] Wichmann L., Jiang S.-K., Thienenkamp J. H., Mohrhardt M., Hwang B. J., Winter M., Brunklaus G. (2025). Origins of
lithium inventory reversibility with an alloying functional layer
in anode-free lithium metal batteries. Nat.
Commun..

[38] Li H., Wu F., Wang J., Wang J., Qu H., Chen M., Zhang H., Passerini S. (2025). Anode-free sodium metal batteries:
optimization of electrolytes and interphases. Energy Environ. Sci..

[39] Saqib M., Mao J., Hao R. (2025). Spatial-temporally
resolved optical imaging of electroplating/stripping
processes in anode-free secondary batteries. Chem. Commun..

[40] Wang J., Zhou Y., Zhuo Y., Fang K., Wang S., Zhao B., Zhou J., Wang H. (2025). The challenges and
strategies towards high-performance anode-free post-lithium metal
batteries. Chem. Sci..

[41] Zor C., Turrell S. J., Uyanik M. S., Afyon S. (2024). Lithium Plating and
Stripping: Toward Anode-Free Solid-State Batteries. Adv. Energy Sustainability Res..

[42] Wang J. F., Weiling M., Pfeiffer F., Liu K. L., Baghernejad M. (2025). Molecular
Insights into the Interfacial Phenomena at the Li Metal| Polymer Solid-State
Electrolyte in Anode-Free Configuration During Li Plating-Stripping
via Advanced Operando ATR-FTIR Spectroscopy. Adv. Energy Mater..

[43] Duan X., Sun J., Shi L., Dong S., Cui G. (2025). Exploring the Active
Lithium Loss in Anode-Free Lithium Metal Batteries: Mechanisms, Challenges,
and Strategies. Interdisciplinary Materials.

[44] Vishweswariah K., Ningappa N. G., Bouguern M. D., Kumar MR A., Armand M. B., Zaghib K. (2025). Evaluation
and Characterization of SEI Composition
in Lithium Metal and Anode-Free Lithium Batteries. Adv. Energy Mater..

[45] Abdollahifar M., Paolella A. (2025). “Dead Lithium” Formation and Mitigation
Strategies in Anode-Free Li-Metal Batteries. Batter. Supercaps.

[46] Plieth, W. Electrochemistry for materials science; Elsevier: New York, 2008.

[47] Liaw B., Pawar G., Meng Y. S., Fang C., Lu B. (2022). Perspective—Lithium
Metal Nucleation and Growth on Conductive Substrates: A Multi-Scale
Understanding from Atomistic, Nano-, Meso-, to Micro-Scales. J. Electrochem. Soc..

[48] Cooper E. R., Li M., Gentle I., Xia Q., Knibbe R. (2023). A deeper understanding
of metal nucleation and growth in rechargeable metal batteries through
theory and experiment. Angew. Chem., Int. Ed..

[49] Tang K., Tian L., Zhang Y., Xu Z. J. (2024). Anode-free lithium
metal batteries: a promising flexible energy storage system. J. Mater. Chem. A.

[50] Chen X. R., Zhao B. C., Yan C., Zhang Q. (2021). Review on Li deposition
in working batteries: from nucleation to early growth. Adv. Mater..

[51] Dong K., Xu Y., Tan J., Osenberg M., Sun F., Kochovski Z., Pham D. T., Mei S., Hilger A., Ryan E. (2021). Unravelling the mechanism of lithium nucleation and growth and the
interaction with the solid electrolyte interface. ACS Energy Lett..

[52] Pei A., Zheng G., Shi F., Li Y., Cui Y. (2017). Nanoscale
nucleation and growth of electrodeposited lithium metal. Nano Lett..

[53] Barton J. L., Bockris J. O. M. (1962). The electrolytic
growth of dendrites from ionic solutions. J.
O. M Proc. R. Soc. London, Ser. A.

[54] Biswal P., Stalin S., Kludze A., Choudhury S., Archer L. A. (2019). Nucleation and early stage growth
of Li electrodeposits. Nano Lett..

[55] Thirumalraj B., Hagos T. T., Huang C.-J., Teshager M. A., Cheng J.-H., Su W.-N., Hwang B.-J. (2019). Nucleation and growth mechanism of
lithium metal electroplating. J. Am. Chem. Soc..

[56] Yan K., Lu Z., Lee H.-W., Xiong F., Hsu P.-C., Li Y., Zhao J., Chu S., Cui Y. (2016). Selective deposition
and stable encapsulation of lithium through heterogeneous seeded growth. Nat. Energy.

[57] Liu W., Lin D., Pei A., Cui Y. (2016). Stabilizing lithium metal anodes
by uniform Li-ion flux distribution in nanochannel confinement. J. Am. Chem. Soc..

[58] Ding F., Xu W., Graff G. L., Zhang J., Sushko M. L., Chen X., Shao Y., Engelhard M. H., Nie Z., Xiao J. (2013). Dendrite-free
lithium deposition via self-healing electrostatic shield
mechanism. J. Am. Chem. Soc..

[59] Xiao J. (2019). How lithium
dendrites form in liquid batteries. Science.

[60] Mistry A., Fear C., Carter R., Love C. T., Mukherjee P. P. (2019). Electrolyte
confinement alters lithium electrodeposition. ACS Energy Lett..

[61] Chen K.-H., Wood K. N., Kazyak E., LePage W. S., Davis A. L., Sanchez A. J., Dasgupta N. P. (2017). Dead lithium: mass
transport effects
on voltage, capacity, and failure of lithium metal anodes. J. Mater. Chem. A.

[62] Xu X., Liu Y., Hwang J. Y., Kapitanova O. O., Song Z., Sun Y. K., Matic A., Xiong S. (2020). Role of Li-ion depletion on electrode
surface: underlying mechanism for electrodeposition behavior of lithium
metal anode. Adv. Energy Mater..

[63] Brissot C., Rosso M., Chazalviel J.-N., Lascaud S. (1999). Dendritic growth mechanisms
in lithium/polymer cells. J. Power Sources.

[64] Bieker G., Winter M., Bieker P. (2015). Electrochemical
in situ investigations
of SEI and dendrite formation on the lithium metal anode. Phys. Chem. Chem. Phys..

[65] Rosso M., Brissot C., Teyssot A., Dollé M., Sannier L., Tarascon J.-M., Bouchet R., Lascaud S. (2006). Dendrite short-circuit
and fuse effect on Li/polymer/Li cells. Electrochim.
Acta.

[66] Hao F., Verma A., Mukherjee P. P. (2018). Mechanistic
insight into dendrite-SEI
interactions for lithium metal electrodes. J.
Mater. Chem. A.

[67] Tan J., Tartakovsky A. M., Ferris K., Ryan E. M. (2016). Investigating the
effects of anisotropic mass transport on dendrite growth in high energy
density lithium batteries. J. Electrochem. Soc..

[68] Wang W.-W., Gu Y., Wang J.-H., Chen Z.-B., Yin X.-T., Wu Q.-H., Yan J.-W., Mao B.-W. (2022). Probing mechanical properties of
solid-electrolyte interphases on Li nuclei by in situ AFM. J. Electrochem. Soc..

[69] Shen X., Zhang R., Chen X., Cheng X. B., Li X., Zhang Q. (2020). The failure of solid electrolyte interphase on Li metal
anode: structural
uniformity or mechanical strength?. Adv. Energy
Mater..

[70] Liu J., Pei N., Hua H., Deng Y., Ma H., Zhang P., Zhao J. (2022). From mosaic-type
to heterojunction-type SEI films on the Li anode:
decoupling chemical and electrochemical degradation of the electrolyte. ACS Sustainable Chem. Eng..

[71] Haregewoin A. M., Wotango A. S., Hwang B.-J. (2016). Electrolyte
additives for lithium
ion battery electrodes: progress and perspectives. Energy Environ. Sci..

[72] Tripathi A. M., Su W.-N., Hwang B. J. (2018). In situ
analytical techniques for
battery interface analysis. Chem. Soc. Rev..

[73] Shitaw K.
N., Huang C.-J., Yang S.-C., Nikodimos Y., Temesgen N. T., Merso S. K., Jiang S.-K., Wang C.-H., Wu S.-H., Su W.-N. (2022). Evolution of interfacial
phenomena induced by electrolyte formulation and hot cycling of anode-free
Li-metal batteries. ACS Appl. Energy Mater..

[74] Huang C.-J., Thirumalraj B., Tao H.-C., Shitaw K. N., Sutiono H., Hagos T. T., Beyene T. T., Kuo L.-M., Wang C.-C., Wu S.-H., Su W.-N., Hwang B. J. (2021). Decoupling the origins
of irreversible coulombic efficiency in anode-free lithium metal batteries. Nat. commun..

[75] Tong Z., Bazri B., Hu S.-F., Liu R.-S. (2021). Interfacial
chemistry
in anode-free batteries: challenges and strategies. J. Mater. Chem. A.

[76] Menkin S., O’Keefe C. A., Gunnarsdóttir A. B., Dey S., Pesci F. M., Shen Z., Aguadero A., Grey C. P. (2021). Toward
an understanding of SEI formation and lithium plating on copper in
anode-free batteries. J. Phys. Chem. C.

[77] Gervillié-Mouravieff C., Ah L., Liu A., Huang C.-J., Meng Y. S. (2024). Deciphering the
Impact of the Active Lithium Reservoir in Anode-Free Pouch Cells. ACS Energy Lett..

[78] Louli A., Coon M., Genovese M., deGooyer J., Eldesoky A., Dahn J. (2021). Optimizing Cycling
Conditions for Anode-Free Lithium Metal Cells. J. Electrochem. Soc..

[79] Genovese M., Louli A., Weber R., Hames S., Dahn J. (2018). Measuring
the coulombic efficiency of lithium metal cycling in anode-free lithium
metal batteries. J. Electrochem. Soc..

[80] Neudecker B., Dudney N., Bates J. (2000). “Lithium-Free”
thin-film
battery with in situ plated Li anode. J. Electrochem.
Soc..

[81] Zhou M.-Y., Ding X.-Q., Ding J.-F., Hou L.-P., Shi P., Xie J., Li B.-Q., Huang J.-Q., Zhang X.-Q., Zhang Q. (2022). Quantifying
the apparent electron transfer number of electrolyte decomposition
reactions in anode-free batteries. Joule.

[82] Gu D., Kim H., Lee J.-H., Park S. (2022). Surface-roughened current collectors
for anode-free all-solid-state batteries. J.
Energy Chem..

[83] Lee J. H., Cho Y.-G., Gu D., Kim S. J. (2022). 2D PdTe2 thin-film-coated
current collectors for long-cycling anode-free rechargeable batteries. ACS Appl. Mater. Interfaces.

[84] Molaiyan P., Abdollahifar M., Boz B., Beutl A., Krammer M., Zhang N., Tron A., Romio M., Ricci M., Adelung R. (2024). Optimizing
current collector interfaces for efficient
“anode-free” lithium metal batteries. Adv. Funct. Mater..

[85] Adhitama E., Refino A. D., Brake T., Pleie J., Schmidt C., Demelash F., Neuhaus K., Bornemann S., Wiemers-Meyer S., Peiner E. (2023). On the direct correlation
between the copper current collector surface area and ‘dead
Li’formation in zero-excess Li metal batteries. J. Mater. Chem. A.

[86] Shan C., Qin Z., Xie Y., Meng X., Chen J., Chang Y., Zang R., Wan L., Huang Y. (2023). Cu-CNTs current collector
fabricated by deformation-driven metallurgy for anode-free Li metal
batteries. Carbon.

[87] Pyo S., Ryu S., Gong Y. J., Cho J., Yun H., Kim H., Lee J., Min B., Choi Y., Yoo J. (2023). Lithiophilic
Wetting Agent Inducing Interfacial Fluorination for Long-Lifespan
Anode-Free Lithium Metal Batteries. Adv. Energy
Mater..

[88] Jote B. A., Beyene T. T., Sahalie N. A., Weret M. A., Olbassa B. W., Wondimkun Z. T., Berhe G. B., Huang C.-J., Su W.-N., Hwang B. J. (2020). Effect of diethyl carbonate solvent with fluorinated
solvents as electrolyte system for anode free battery. J. Power Sources.

[89] Kim E., Choi W., Ryu S., Yun Y., Jo S., Yoo J. (2023). Effect of 3D lithiophilic
current collector for anode-free Li ion
batteries. J. Alloy. Compd..

[90] Jiang C., Ding W., Wu H., Yu Z., Ma L., Zou Z. (2018). Hierarchical Li4Ti5O12 nanosheet arrays anchoring on
carbon fiber
cloth as ultra-stable free-standing anode of Li-ion battery. Ceram. Int..

[91] Wu W., Ning D., Zhang J., Liu G., Zeng L., Yao H., Wang M., Deng L., Yao L. (2023). Ultralight lithiophilic
three-dimensional lithium host for stable high-energy-density anode-free
lithium metal batteries. Energy Storage Mater..

[92] Lo C.-A., Chang C.-C., Tsai Y.-W., Jiang S.-K., Hwang B. J., Mou C.-Y., Wu H.-L. (2021). Regulated
Li electrodeposition behavior
through mesoporous silica thin film in anode-free lithium metal batteries. ACS Appl. Energy Mater..

[93] Lee N., Lee J., Lee T., Oh J., Hwang I., Seo G., Kim H., Choi J. W. (2023). Rationally
Designed Solution-Processible Conductive
Carbon Additive Coating for Sulfide-based All-Solid-State Batteries. ACS Appl. Mater. Interfaces.

[94] He Y., Xu H., Shi J., Liu P., Tian Z., Dong N., Luo K., Zhou X., Liu Z. (2019). Polydopamine coating layer modified
current collector for dendrite-free Li metal anode. Energy Storage Mater..

[95] Oyakhire S. T., Huang W., Wang H., Boyle D. T., Schneider J. R., de Paula C., Wu Y., Cui Y., Bent S. F. (2020). Revealing
and elucidating ALD-derived control of lithium plating microstructure. Adv. Energy Mater..

[96] Weldeyohannes H. H., Abrha L. H., Nikodimos Y., Shitaw K. N., Hagos T. M., Huang C.-J., Wang C.-H., Wu S.-H., Su W.-N., Hwang B. J. (2021). Guiding lithium-ion flux to avoid cell’s short
circuit and extend cycle life for an anode-free lithium metal battery. J. Power Sources.

[97] Chang W., Steingart D. (2021). Operando 2D acoustic characterization of lithium-ion
battery spatial dynamics. ACS Energy Lett..

[98] Jin C., Sheng O., Wei G., Li H., Han Q., Zhang Q., Tao X. (2025). Inhibiting
and rejuvenating dead
lithium in battery materials. Nat. Rev. Chem..

[99] Yoon G., Moon S., Ceder G., Kang K. (2018). Deposition and stripping
behavior of lithium metal in electrochemical system: continuum mechanics
study. Chem. Mater..

[100] Shen X., Zhang R., Shi P., Zhang X.-Q., Chen X., Zhao C.-Z., Wu P., Guo Y.-M., Huang J.-Q., Zhang Q. (2024). The dead lithium formation
under
mechano-electrochemical coupling in lithium metal batteries. Fundam. Res..

[101] Zhang R., Shen X., Zhang Y.-T., Zhong X.-L., Ju H.-T., Huang T.-X., Chen X., Zhang J.-D., Huang J.-Q. (2022). Dead lithium formation in lithium metal batteries:
A phase field model. J. Energy Chem..

[102] Tewari D., Rangarajan S. P., Balbuena P. B., Barsukov Y., Mukherjee P. P. (2020). Mesoscale
anatomy of dead lithium formation. J. Phys.
Chem. C.

[103] Lee H. G., Kim S. Y., Lee J. S. (2022). Dynamic observation
of dendrite growth on lithium metal anode during battery charging/discharging
cycles. Npj Comput. Mater..

[104] Liu S., Jiao K., Yan J. (2023). Prospective
strategies for extending
long-term cycling performance of anode-free lithium metal batteries. Energy Storage Mater..

[105] Tao M., Xiang Y., Zhao D., Shan P., Sun Y., Yang Y. (2022). Quantifying the evolution of inactive Li/lithium hydride
and their
correlations in rechargeable anode-free Li batteries. Nano Lett..

[106] Jin C., Liu T., Sheng O., Li M., Liu T., Yuan Y., Nai J., Ju Z., Zhang W., Liu Y. (2021). Rejuvenating dead lithium
supply in lithium metal anodes
by iodine redox. Nat. Energy.

[107] Chen J., He B., Cheng Z., Rao Z., He D., Liu D., Li X., Yuan L., Huang Y., Li Z. (2021). Reactivating dead Li by shuttle effect
for high-performance anode-free
Li metal batteries. J. Electrochem. Soc..

[108] Liu F., Xu R., Wu Y., Boyle D. T., Yang A., Xu J., Zhu Y., Ye Y., Yu Z., Zhang Z. (2021). Dynamic spatial progression
of isolated lithium during battery operations. Nature.

[109] Zhang W., Sayavong P., Xiao X., Oyakhire S. T., Shuchi S. B., Vilá R. A., Boyle D. T., Kim S. C., Kim M. S., Holmes S. E. (2024). Recovery of isolated
lithium through discharged state calendar ageing. Nature.

[110] Luo Y., Yang X., Wang C., Fraser A., Zhang H., Sun X., Li X. (2023). Advanced metal anodes
and their interface design toward
safe metal batteries: A comprehensive review. Prog. Mater. Sci..

[111] Wu H., Luo S., Wang H., Li L., Fang Y., Zhang F., Gao X., Zhang Z., Yuan W. (2024). A Review of
Anode Materials for Dual-Ion Batteries. Nanomicro
Lett..

[112] Ma J., Zheng S., Fu Y., Wang X., Qin J.-Q., Wu Z.-S. (2024). The status and challenging perspectives
of 3D-printed micro-batteries. Chem. Sci..

[113] Liu W., Liu P., Mitlin D. (2020). Review of
emerging concepts in SEI
analysis and artificial SEI membranes for lithium, sodium, and potassium
metal battery anodes. Adv. Energy Mater..

[114] Tewari D., Mukherjee P. P. (2019). Mechanistic
understanding of electrochemical
plating and stripping of metal electrodes. J.
Mater. Chem. A.

[115] Soto F. A., Marzouk A., El-Mellouhi F., Balbuena P. B. (2018). Understanding ionic
diffusion through SEI components
for lithium-ion and sodium-ion batteries: Insights from first-principles
calculations. Chem. Mater..

[116] Zeng X., Mao J., Hao J., Liu J., Liu S., Wang Z., Wang Y., Zhang S., Zheng T., Liu J. (2021). Electrolyte design for
in situ construction of highly
Zn2+-conductive solid electrolyte interphase to enable high-performance
aqueous Zn-ion batteries under practical conditions. Adv. Mater..

[117] Mogensen R., Brandell D., Younesi R. (2016). Solubility of the solid
electrolyte interphase (SEI) in sodium ion batteries. ACS Energy Lett..

[118] Seh Z. W., Sun J., Sun Y., Cui Y. (2015). A highly reversible
room-temperature sodium metal anode. ACS cent.
sci..

[119] Ma J.-l., Meng F.-l., Yu Y., Liu D.-p., Yan J.-m., Zhang Y., Zhang X.-b., Jiang Q. (2019). Prevention
of dendrite growth and volume expansion to give high-performance aprotic
bimetallic Li-Na alloy-O2 batteries. Nat. chem..

[120] Wang H., Zhai D., Kang F. (2020). Solid electrolyte interphase
(SEI) in potassium ion batteries. Energy Environ.
Sci..

[121] Zhu G., Tian X., Tai H.-C., Li Y.-Y., Li J., Sun H., Liang P., Angell M., Huang C.-L., Ku C.-S. (2021). Rechargeable Na/Cl2 and Li/Cl2 batteries. Nature.

[122] Forero-Saboya J., Davoisne C., Dedryvère R., Yousef I., Canepa P., Ponrouch A. (2020). Understanding the nature
of the passivation layer enabling reversible calcium plating. Energy Environ. Sci..

[123] Davidson R., Verma A., Santos D., Hao F., Fincher C., Xiang S., Van Buskirk J., Xie K., Pharr M., Mukherjee P. P. (2019). Formation of magnesium
dendrites during electrodeposition. ACS Energy
Lett..

[124] Zhao Q., Zachman M. J., Al Sadat W. I., Zheng J., Kourkoutis L. F., Archer L. (2018). Solid electrolyte interphases for
high-energy aqueous aluminum electrochemical cells. Sci. adv..

[125] Shinde S. S., Wagh N. K., Lee C. H., Kim D. H., Kim S. H., Um H. D., Lee S. U., Lee J. H. (2023). Scaling-Up
Insights for Zinc-Air Battery Technologies Realizing Reversible Zinc
Anodes. Adv. Mater..

[126] Hao J., Li B., Li X., Zeng X., Zhang S., Yang F., Liu S., Li D., Wu C., Guo Z. (2020). An in-depth study of Zn metal surface
chemistry for advanced aqueous
Zn-ion batteries. Adv. Mater..

[127] Zheng J., Zhao Q., Tang T., Yin J., Quilty C. D., Renderos G. D., Liu X., Deng Y., Wang L., Bock D. C. (2019). Reversible epitaxial
electrodeposition of metals in battery anodes. Science.

[128] An S. J., Li J., Daniel C., Mohanty D., Nagpure S., Wood D. L. (2016). The state of
understanding of the lithium-ion-battery graphite solid electrolyte
interphase (SEI) and its relationship to formation cycling. Carbon.

[129] Madec L., Gabaudan V., Gachot G., Stievano L., Monconduit L., Martinez H. (2018). Paving the way for K-ion batteries:
role of electrolyte reactivity through the example of Sb-based electrodes. ACS Appl. Mater. Interfaces.

[130] Jian Z., Luo W., Ji X. (2015). Carbon electrodes
for
K-ion batteries. J. Am. Chem. Soc..

[131] Chang W. C., Wu J. H., Chen K. T., Tuan H. Y. (2019). Red phosphorus
potassium-ion battery anodes. Adv. Sci..

[132] Hu Y.-S. (2016). Batteries: getting solid. Nat. Energy.

[133] Yoshida K., Nakamura M., Kazue Y., Tachikawa N., Tsuzuki S., Seki S., Dokko K., Watanabe M. (2011). Oxidative-stability
enhancement and charge transport mechanism in glyme-lithium salt equimolar
complexes. J. Am. Chem. Soc..

[134] Chen X., Li H. R., Shen X., Zhang Q. (2018). The origin
of the reduced reductive stability of ion-solvent complexes on alkali
and alkaline earth metal anodes. Angew. Chem.,
Int. Ed..

[135] Peljo P., Girault H. H. (2018). Electrochemical potential window
of battery electrolytes: the HOMO-LUMO misconception. Energy Environ. Sci..

[136] Shinde S. S., Wagh N. K., Kim S. H., Lee J. H. (2023). Li, Na,
K, Mg, Zn, Al, and Ca Anode Interface Chemistries Developed by Solid-State
Electrolytes. Adv. Sci..

[137] Tian Y., An Y., Wei C., Jiang H., Xiong S., Feng J., Qian Y. (2020). Recently advances
and
perspectives of anode-free rechargeable batteries. Nano Energy.

[138] Ding K., Zhong M., Zeng X., Yuan S., Wang Y. (2023). A perspective
on the critical design criteria for anode-free li metal
batteries. Energy Lab.

[139] Taklu B. W., Su W.-N., Chiou J.-C., Chang C.-Y., Nikodimos Y., Lakshmanan K., Hagos T. M., Serbessa G. G., Desta G. B., Tekaligne T. M. (2024). Mechanistic study on
artificial stabilization of lithium metal anode via thermal pyrolysis
of ammonium fluoride in lithium metal batteries. ACS Appl. Mater. Interfaces.

[140] Xiao J., Li Q., Bi Y., Cai M., Dunn B., Glossmann T., Liu J., Osaka T., Sugiura R., Wu B. (2020). Understanding and applying
coulombic efficiency in lithium metal batteries. Nat. energy.

[141] Weber R., Genovese M., Louli A., Hames S., Martin C., Hill I. G., Dahn J. (2019). Long cycle life and
dendrite-free lithium morphology in anode-free lithium pouch cells
enabled by a dual-salt liquid electrolyte. Nat.
Energy.

[142] Ouyang Z., Wang S., Wang Y., Muqaddas S., Geng S., Yao Z., Zhang X., Yuan B., Zhao X., Xu Q. (2024). An Ultralight Composite
Current Collector Enabling High-Energy-Density and High-Rate Anode-Free
Lithium Metal Battery. Adv. Mater..

[143] Shen X., Zhang R., Shi P., Chen X., Zhang Q. (2021). How does external pressure shape
Li dendrites in Li metal batteries?. Adv. Energy
Mater..

[144] Gao L. T., Lyu Y., Guo Z.-S. (2023). External Pressure
Affecting Dendrite Growth and Dissolution in Lithium Metal Batteries
During Cycles. ACS Appl. Mater. Interfaces.

[145] Liu W., Luo Y., Hu Y., Chen Z., Wang Q., Chen Y., Iqbal N., Mitlin D. (2024). Interrelation between
external pressure, SEI structure, and electrodeposit morphology in
an anode-free lithium metal battery. Adv. Energy
Mater..

[146] Louli A. J., Genovese M., Weber R., Hames S., Logan E., Dahn J. (2019). Exploring the impact of mechanical
pressure on the performance of anode-free lithium metal cells. J. Electrochem. Soc..

[147] Lim H.-S., Nguyen D. T., Lochala J. A., Cao X., Zhang J.-G. (2024). Improving Cycling Performance of Anode-Free Lithium
Batteries by Pressure and Voltage Control. ACS
Energy Lett..

[148] Yang H., Liu X., Zheng J., Yao W., Fang K., Lai X., Zhai G., Wu M., Li G., Luo W. (2024). Mitigating Overcharge in Ampere-Hour-Level Anode-Free
Pouch Cells by Improving Pressure Uniformity. ACS Energy Lett..

[149] Willow A., Hussein H. E., Vajirakaphan S., Chasri A., Margadonna S. (2022). Improving in-situ sodium metal plating
on copper foil through optimization of mechanical pressure: towards
high-performance anode-free sodium ion batteries. Front. Energy Res..

[150] Dong Y., Xu C., Zhao H., Chen L., Shi W., Irvine J., Lei Y. (2025). Interface Engineering Strategies
for Realizing Anode-Free Sodium Batteries: A Review. Adv. Energy Mater..

[151] Chen J., Lan H., Wang S., Liu X., Zhu Q., Zhang X., Tang M., Dong S., Yang J., Kurbanov M. (2025). Realizing an Energy-Dense
Potassium Metal Battery
at- 40° C via an Integrated Anode-Free and Dual-Ion Strategy. J. Am. Chem. Soc..

[152] An Y., Zeng Y., Luan D., Lou X. W. D. (2024). Materials design
for high-energy-density anode-free batteries. Matter.

[153] Mao M., Fan X., Xie W., Wang H., Suo L., Wang C. (2023). The Proof-of-Concept of Anode-Free Rechargeable Mg
Batteries. Adv. Sci..

[154] Thorpe M. A., Zhang M., Liao D. W., Sandoval S. E., Kim Y., McDowell M. T., Thouless M., Dasgupta N. P. (2025). Controlling stack
pressure inhomogeneity in anode-free solid-state batteries using elastomeric
interlayers. Matter.

[155] Sandoval S. E., Haslam C. G., Vishnugopi B. S., Liao D. W., Yoon J. S., Park S. H., Wang Y., Mitlin D., Hatzell K. B., Siegel D. J. (2025). Electro-chemo-mechanics
of anode-free solid-state batteries. Nat. Mater..

[156] Zhang J., Fu J., Lu P., Hu G., Xia S., Zhang S., Wang Z., Zhou Z., Yan W., Xia W. (2025). Challenges and Strategies of Low-Pressure All-Solid-State
Batteries. Adv. Mater..

[157] Wu B., Chen C., Raijmakers L. H., Liu J., Danilov D. L., Eichel R.-A., Notten P. H. (2023). Li-growth and SEI engineering for
anode-free Li-metal rechargeable batteries: A review of current advances. Energy Storage Mater..

[158] Chang W., Thorsteinsson G., Janakiraman U., Chowdhury R. R., Herman Z., Katzman L., Steingart D. A. (2024). Relating
chemo-mechanical hysteresis and formation protocols for anode-free
lithium metal batteries. J. Electrochem. Soc..

[159] Kim S., Didwal P. N., Fiates J., Dawson J. A., Weatherup R. S., De Volder M. (2024). Effect of
the Formation Rate on the Stability of Anode-Free
Lithium Metal Batteries. ACS Energy Lett..

[160] Cicvarić K., Pohlmann S., Zhang B., Rahmanian F., Merker L., Gaberšček M., Stein H. S. (2024). Fast formation
of anode-free Li-metal batteries by pulsed current. Phys. Chem. Chem. Phys..

[161] Genovese M., Louli A., Weber R., Martin C., Taskovic T., Dahn J. (2019). Hot formation for improved
low temperature
cycling of anode-free lithium metal batteries. J. Electrochem. Soc..

[162] Cheng X.-B., Zhang R., Zhao C.-Z., Zhang Q. (2017). Toward safe
lithium metal anode in rechargeable batteries: a review. Chem. Rev..

[163] Kim S., Didwal P. N., Fiates J., Dawson J. A., Weatherup R. S., De Volder M. (2024). Effect of
the Formation Rate on the Stability of Anode-Free
Lithium Metal Batteries. ACS Energy Lett..

[164] Seong I. W., Hong C. H., Kim B. K., Yoon W. Y. (2008). The effects
of current density and amount of discharge on dendrite formation in
the lithium powder anode electrode. J. Power
Sources.

[165] Zhang J., Zhang H., Deng L., Yang Y., Tan L., Niu X., Chen Y., Zeng L., Fan X., Zhu Y. (2023). An additive-enabled
ether-based electrolyte to realize stable cycling
of high-voltage anode-free lithium metal batteries. Energy Storage Mater..

[166] Zhang Y.-Y., Zhang C.-H., Guo Y.-J., Fan M., Zhao Y., Guo H., Wang W.-P., Tan S.-J., Yin Y.-X., Wang F. (2023). Refined electrolyte
and interfacial chemistry toward realization of high-energy anode-free
rechargeable sodium batteries. J. Am. Chem.
Soc..

[167] Shi W., Song Z., Zhang W., Lian S., Huang F., An Q., Li Q. (2024). Identifying iodide-ion regulation of early-stage zinc
nucleation and growth for high-rate anode-free zinc metal batteries. Energy Environ. Sci..

[168] He J., Bhargav A., Manthiram A. (2022). High-performance anode-free Li-S
batteries with an integrated Li2S-electrocatalyst cathode. ACS Energy Lett..

[169] Taklu B. W., Yeh T.-I., Chang C.-Y., Hagos T. M., Nikodimos Y., Vallal S. A., Windmüller A., Chang C.-C., Shitaw K. N., Agnihotri T. (2025). Two-Step Activations and Liquid Metal Fortified Copper Substrate
on Corrosion Tolerance in Anode-Free Lithium-Sulfur Battery. ACS Energy Lett..

[170] Sun H., Liang P., Zhu G., Hung W. H., Li Y.-Y., Tai H.-C., Huang C.-L., Li J., Meng Y., Angell M. (2020). A high-performance potassium
metal battery using safe
ionic liquid electrolyte. Proc. Natl. Acad.
Sci. U.S.A..

[171] Ni’Mah Y. L., Cheng M.-Y., Cheng J. H., Rick J., Hwang B.-J. (2015). Solid-state polymer nanocomposite electrolyte of TiO2/PEO/NaClO4
for sodium ion batteries. J. Power Sources.

[172] Chen J., Yu D., Zhu Q., Liu X., Wang J., Chen W., Ji R., Qiu K., Guo L., Wang H. (2022). Low-Temperature High-Areal-Capacity Rechargeable Potassium-Metal
Batteries. Adv. Mater..

[173] Pan C.-J., Yuan C., Zhu G., Zhang Q., Huang C.-J., Lin M.-C., Angell M., Hwang B.-J., Kaghazchi P., Dai H. (2018). An operando X-ray diffraction
study
of chloroaluminate anion-graphite intercalation in aluminum batteries. Proc. Natl. Acad. Sci. U.S.A..

[174] Angell M., Pan C.-J., Rong Y., Yuan C., Lin M.-C., Hwang B.-J., Dai H. (2017). High Coulombic
efficiency
aluminum-ion battery using an AlCl3-urea ionic liquid analog electrolyte. Proc. Natl. Acad. Sci. U.S.A..

[175] Wang D.-Y., Wei C.-Y., Lin M.-C., Pan C.-J., Chou H.-L., Chen H.-A., Gong M., Wu Y., Yuan C., Angell M. (2017). Advanced rechargeable
aluminium ion battery with a high-quality natural graphite cathode. Nat. commun..

[176] Xie J., Zhang Q. (2019). Recent Progress in multivalent metal (Mg, Zn, Ca, and
Al) and metal-ion rechargeable batteries with organic materials as
promising electrodes. Small.

[177] Mahmood A., Bai Z., Wang T., Lei Y., Wang S., Sun B., Khan H., Khan K., Sun K., Wang G. (2025). Enabling high-performance multivalent metal-ion batteries:
current advances and future prospects. Chem.
Soc. Rev..

[178] Kitaura H., Hayashi A., Tadanaga K., Tatsumisago M. (2009). High-rate
performance of all-solid-state lithium secondary batteries using Li4Ti5O12
electrode. J. Power Sources.

[179] Lee D., Lee H., Song T., Paik U. (2022). Toward high rate performance
solid-state batteries. Adv. Energy Mater..

[180] Lou S., Zhang F., Fu C., Chen M., Ma Y., Yin G., Wang J. (2021). Interface
issues and challenges in all-solid-state
batteries: lithium, sodium, and beyond. Adv.
Mater..

[181] Betz J., Bieker G., Meister P., Placke T., Winter M., Schmuch R. (2019). Theoretical versus practical energy:
a plea for more transparency in the energy calculation of different
rechargeable battery systems. Adv. Energy Mater..

[182] Lin L., Qin K., Hu Y. s., Li H., Huang X., Suo L., Chen L. (2022). A Better Choice to
Achieve High Volumetric Energy Density:
Anode-Free Lithium-Metal Batteries. Adv. Mater..

[183] Park, C. Y. ; Kim, J. ; Lim, W. G. ; Lee, J. In Toward maximum energy density enabled by anode-free lithium metal batteries: Recent progress and perspective; Exploration, Wiley Online Library, 2024; p 20210255. 10.1002/EXP.20210255 PMC11022618 38855623

[184] Salvatierra R. V., Chen W., Tour J. M. (2021). What Can be Expected
from “Anode-Free” Lithium Metal Batteries?. Adv. Energy Sustainability Res..

[185] Schmuch R., Wagner R., Hörpel G., Placke T., Winter M. (2018). Performance and cost of materials
for lithium-based rechargeable automotive batteries. Nat. energy.

[186] Shen X., Liu H., Cheng X.-B., Yan C., Huang J.-Q. (2018). Beyond lithium ion batteries: Higher energy density
battery systems based on lithium metal anodes. Energy Storage Mater..

[187] Choi J. W., Aurbach D. (2016). Promise and reality of post-lithium-ion
batteries with high energy densities. Nat. Rev.
Mater..

[188] Cao W., Zhang J., Li H. (2020). Batteries with high
theoretical energy
densities. Energy Storage Mater..

[189] Placke T., Kloepsch R., Dühnen S., Winter M. (2017). Lithium ion, lithium metal, and alternative rechargeable
battery technologies: the odyssey for high energy density. J. Solid State Electrochem..

[190] Li Y., Zhou Q., Weng S., Ding F., Qi X., Lu J., Li Y., Zhang X., Rong X., Lu Y. (2022). Interfacial
engineering to achieve an energy density of over 200
Wh kg- 1 in sodium batteries. Nat. Energy.

[191] Wang Y., Wang Y., Wang Y.-X., Feng X., Chen W., Ai X., Yang H., Cao Y. (2019). Developments
and perspectives on emerging high-energy-density sodium-metal batteries. Chem..

[192] Chen J., Peng Y., Yin Y., Liu M., Fang Z., Xie Y., Chen B., Cao Y., Xing L., Huang J. (2022). High energy density
Na-metal batteries enabled by a tailored carbonate-based electrolyte. Energy Environ. Sci..

[193] Choi J. U., Park Y. J., Jo J. H., Jung Y. H., Ahn D.-C., Jeon T.-Y., Lee K.-S., Kim H., Lee S., Kim J. (2020). An optimized approach
toward high energy density
cathode material for K-ion batteries. Energy
Storage Mater..

[194] Mao M., Gao T., Hou S., Wang F., Chen J., Wei Z., Fan X., Ji X., Ma J., Wang C. (2019). High-energy-density
rechargeable Mg battery enabled by a displacement reaction. Nano Lett..

[195] Deng M., Wang L., Vaghefinazari B., Xu W., Feiler C., Lamaka S. V., Höche D., Zheludkevich M. L., Snihirova D. (2021). High-energy and durable aqueous magnesium
batteries: Recent advances and perspectives. Energy Storage Mater..

[196] Ma L., Li N., Long C., Dong B., Fang D., Liu Z., Zhao Y., Li X., Fan J., Chen S. (2019). Achieving both high voltage and high capacity
in aqueous zinc-ion
battery for record high energy density. Adv.
Funct. Mater..

[197] Ruan P., Liang S., Lu B., Fan H. J., Zhou J. (2022). Design strategies
for high-energy-density aqueous zinc batteries. Angew. Chem..

[198] Wu Y., Xie L., Ming H., Guo Y., Hwang J.-Y., Wang W., He X., Wang L., Alshareef H. N., Sun Y.-K. (2020). An empirical model for
the design of batteries with
high energy density. ACS Energy Lett..

[199] Dixit M., Parejiya A., Essehli R., Muralidharan N., Haq S. U., Amin R., Belharouak I. (2022). SolidPAC is
an interactive battery-on-demand energy density estimator for solid-state
batteries. Cell Rep. Phys. Sci..

[200] Maksimovna Vakhrusheva D., Xu J. (2025). Model-Driven
Manufacturing
of High-Energy-Density Batteries: A Review. Batter. Supercaps.

[201] Aslam M. K., Niu Y., Hussain T., Tabassum H., Tang W., Xu M., Ahuja R. (2021). How to avoid
dendrite
formation in metal batteries: Innovative strategies for dendrite suppression. Nano Energy.

[202] Liu Y., Liu Q., Xin L., Liu Y., Yang F., Stach E. A., Xie J. (2017). Making Li-metal electrodes rechargeable
by controlling the dendrite growth direction. Nat. Energy.

[203] Hagos T. M., Bezabh H. K., Huang C.-J., Jiang S.-K., Su W.-N., Hwang B. J. (2021). A powerful protocol based on anode-free
cells combined with various analytical techniques. Acc. Chem. Res..

[204] KüPers V., Kolek M., Bieker P., Winter M., Brunklaus G. (2019). In situ 7 Li-NMR analysis of lithium
metal surface
deposits with varying electrolyte compositions and concentrations. Phys. Chem. Chem. Phys..

[205] Wang X., Zhang M., Alvarado J., Wang S., Sina M., Lu B., Bouwer J., Xu W., Xiao J., Zhang J.-G. (2017). New insights on the
structure of electrochemically deposited lithium metal and its solid
electrolyte interphases via cryogenic TEM. Nano
Lett..

[206] Yang Y., Zheng G., Misra S., Nelson J., Toney M. F., Cui Y. (2012). High-capacity micrometer-sized Li2S
particles as cathode materials for advanced rechargeable lithium-ion
batteries. J. Am. Chem. Soc..

[207] Cheng J.-H., Assegie A. A., Huang C.-J., Lin M.-H., Tripathi A. M., Wang C.-C., Tang M.-T., Song Y.-F., Su W.-N., Hwang B. J. (2017). Visualization of
lithium plating
and stripping via in operando transmission X-ray microscopy. J. Phys. Chem. C.

[208] Huang C.-J., Tao H.-C., Chao P.-J., Li C.-Y., Hotasi B. T., Liu H.-Y., Lin M.-H., Wu S.-H., Su W.-N., Hwang B. J. (2023). The Entanglement
of Li Capping and
Deposition: An Operando Optical Microscopy Study. ACS Nano.

[209] Fang C., Li J., Zhang M., Zhang Y., Yang F., Lee J. Z., Lee M.-H., Alvarado J., Schroeder M. A., Yang Y. (2019). Quantifying inactive
lithium in lithium metal batteries. Nature.

[210] Zhou Y., Su M., Yu X., Zhang Y., Wang J.-G., Ren X., Cao R., Xu W., Baer D. R., Du Y. (2020). Real-time
mass spectrometric
characterization of the solid-electrolyte interphase of a lithium-ion
battery. Nat. nanotechnol..

[211] Gunnarsdóttir A. B., Amanchukwu C. V., Menkin S., Grey C. P. (2020). Noninvasive in situ NMR study of
“dead lithium” formation and lithium corrosion in full-cell
lithium metal batteries. J. Am. Chem. Soc..

[212] Hsieh Y.-C., Leißing M., Nowak S., Hwang B.-J., Winter M., Brunklaus G. (2020). Quantification of dead lithium via
in situ nuclear magnetic resonance spectroscopy. Cell Rep..

[213] Zhang S., Li Y., Bannenberg L. J., Liu M., Ganapathy S., Wagemaker M. (2024). The lasting impact of formation cycling
on the Li-ion kinetics between SEI and the Li-metal anode and its
correlation with efficiency. Sci. Adv..

[214] Jeong W.-H., Kim H., Kansara S., Lee S., Agostini M., Kim K., Hwang J.-Y., Jung Y.-C. (2024). Stimulating
the electrostatic interactions in composite cathodes using a slurry-fabricable
polar binder for practical all-solid-state batteries. Energy Storage Mater..

[215] Fan X., Chen L., Borodin O., Ji X., Chen J., Hou S., Deng T., Zheng J., Yang C., Liou S.-C. (2018). Non-flammable electrolyte enables Li-metal batteries with aggressive
cathode chemistries. Nat. Nanotechnol..

[216] Hagos T. T., Su W.-N., Huang C.-J., Thirumalraj B., Chiu S.-F., Abrha L. H., Hagos T. M., Bezabh H. K., Berhe G. B., Tegegne W. A. (2020). Developing high-voltage
carbonate-ether mixed electrolyte via anode-free cell configuration. J. Power Sources.

[217] Rodriguez R., Loeffler K. E., Edison R. A., Stephens R. M., Dolocan A., Heller A., Mullins C. B. (2018). Effect of the electrolyte
on the cycling efficiency of lithium-limited cells and their morphology
studied through in situ optical imaging. ACS
Appl. Energy Mater..

[218] Hagos T. T., Thirumalraj B., Huang C.-J., Abrha L. H., Hagos T. M., Berhe G. B., Bezabh H. K., Cherng J., Chiu S.-F., Su W.-N., Hwang B. J. (2019). Locally concentrated
LiPF6 in a carbonate-based electrolyte with fluoroethylene carbonate
as a diluent for anode-free lithium metal batteries. ACS Appl. Mater. Interfaces.

[219] Hagos T. M., Berhe G. B., Hagos T. T., Bezabh H. K., Abrha L. H., Beyene T. T., Huang C.-J., Yang Y.-W., Su W.-N., Dai H., Hwang B. J. (2019). Dual electrolyte
additives of potassium hexafluorophosphate and tris (trimethylsilyl)
phosphite for anode-free lithium metal batteries. Electrochim. Acta.

[220] Hu Z., Liu L., Wang X., Zheng Q., Han C., Li W. (2024). Current Progress of
Anode-Free Rechargeable Sodium Metal Batteries:
Origin, Challenges, Strategies, and Perspectives. Adv. Funct. Mater..

[221] Shao A., Tang X., Zhang M., Bai M., Ma Y. (2022). Challenges,
strategies, and prospects of the anode-free lithium metal
batteries. Adv. Energy Sustainability Res..

[222] Li S., Wu F., Chen T., Kang K., Guo R., Liu C., Niu Y., Gao A., Zhao R., Wang X. (2025). Progress and Challenges
for Energy-Dense and Cost-Effective Anode-Free
Lithium Metal Batteries. Energy Mater. Adv..

[223] Lin L., Suo L., Hu Y. s., Li H., Huang X., Chen L. (2021). Epitaxial induced plating current-collector
lasting lifespan of anode-free
lithium metal battery. Adv. Energy Mater..

[224] Dahunsi O. J., Li B., An B., Abdul Razak I. B., Xia F., Gao S., Chen J., Li G., Cheng Y. (2023). Directing
High-Efficiency Na Plating with Carbon-Aluminum Junction Interfaces
for Anode-Free Na Metal Batteries. Energy Fuels.

[225] Wang Y., Qu Z., Geng S., Liao M., Ye L., Shadike Z., Zhao X., Wang S., Xu Q., Yuan B. (2023). Anode-free lithium metal batteries based on an ultrathin
and respirable interphase layer. Angew. Chem..

[226] Wang X., He Y., Tu S., Fu L., Chen Z., Liu S., Cai Z., Wang L., He X., Sun Y. (2022). Li plating on alloy with superior electro-mechanical
stability for high energy density anode-free batteries. Energy Storage Mater..

[227] Lee J.-A., Kang H., Kim S., Lee K., Byun J. H., Kwon E., Seo S., Kwak K., Ryu K. H., Choi N.-S. (2024). Unveiling degradation mechanisms
of anode-free Li-metal batteries. Energy Storage
Mater..

[228] Zhou Y., Wang P., Wang K., Fang X., Li W., Nai J., Liu Y., Wang Y., Zou S., Yuan H. (2025). Developing
High-Performance Anode-Free Lithium Batteries:
Challenges, Strategies, and Opportunities. Adv.
Funct. Mater..

[229] Offermann J., Paolella A., Adelung R., Abdollahifar M. (2024). Rising anode-free
lithium-sulfur batteries. Chem. Eng. J..

[230] Weret M. A., Jiang S.-K., Shitaw K. N., Chang C.-Y., Tekaligne T. M., Chiou J.-C., Yang S.-C., Temesgen N. T., Nikodimos Y., Wu S.-H. (2023). Reviving
Inactive Lithium
and Stabilizing Lithium Deposition for Improving the Performance of
Anode-Free Lithium-Sulfur Batteries. ACS Energy
Lett..

[231] He J., Bhargav A., Okasinski J., Manthiram A. (2024). A Class of
Sodium Transition-Metal Sulfide Cathodes With Anion Redox. Adv. Mater..

[232] Yang X., Zhang Y., Wang L., Kang J., Zhai Z., Zhang J., Zhang L., Lu H. (2025). Anode-free
Zn-I2 batteries without fear of Zn loss: Hybrid energy-storage mechanism
and dynamic capacity compensation. J. Colloid
Interface Sci..

[233] Im C. Y., Lee G. Y., Kim J. G., Choi J. H., Kim S. J. (2025). Controlling Lithium
Surface Diffusivity via 2D PtTe2,
PdTe2, and NiTe2 Coatings for Anode-Free and Lithium Metal Batteries. Adv. Mater..

[234] Shao A., Wang H., Zhang M., Liu J., Cheng L., Li Y., Guo Y., Wang Z., Jia Q., Wang X. (2025). Multiscale interfacial stabilization via prelithiation
separator engineering for Ah-level anode-free lithium batteries. Nat. Commun..

[235] Ma P., Kumar R., Wang K.-H., Amanchukwu C. V. (2025). Active
learning accelerates electrolyte solvent screening for anode-free
lithium metal batteries. Nat. Commun..

[236] Sun, J. ; Zhang, S. ; Li, J. ; Xie, B. ; Ma, J. ; Dong, S. ; Cui, G. , Robust Transport: An Artificial Solid Electrolyte Interphase Design for Anode-Free Lithium-Metal Batteries. Adv. Mater. 2023, 35, 2209404. 10.1002/adma.202209404 36573477

[237] Shin W., Manthiram A. (2022). A facile potential
hold method for
fostering an inorganic solid-electrolyte interphase for anode-free
lithium-metal batteries. Angew. Chem..

[238] Kim H.-I., Lee K. M., Kim W.-Y., Kweon S. H., Wang X., Zheng S., Kim S.-H., Ha J. H., Kang S. J., Wu Z.-S. (2024). Restructuring of aqueous
electrolytes using a soft-acidic/hard-basic zwitterion for low-temperature
anode-free Zn batteries. Energy Environ. Sci..

[239] Ming F., Zhu Y., Huang G., Emwas A.-H., Liang H., Cui Y., Alshareef H. N. (2022). Co-solvent
electrolyte engineering for stable anode-free zinc metal batteries. J. Am. Chem. Soc..

[240] An Y., Tian Y., Zhang K., Liu Y., Liu C., Xiong S., Feng J., Qian Y. (2021). Stable aqueous
anode-free
zinc batteries enabled by interfacial engineering. Adv. Funct. Mater..

[241] Weret M. A., Su W. N., Hwang B. J. (2022). Strategies towards
High Performance Lithium-Sulfur Batteries. Batter.
Supercaps.

[242] Lin H., Zhang S., Zhang T., Cao S., Ye H., Yao Q., Zheng G. W., Lee J. Y. (2019). A cathode-integrated sulfur-deficient
Co9S8 catalytic interlayer for the reutilization of “lost”
polysulfides in lithium-sulfur batteries. ACS
Nano.

[243] Vinay B., Nikodimos Y., Agnihotri T., Ahmed S. A., Hagos T. M., Hasan R., Tamilarasan E. B., Su W.-N., Hwang B. J. (2025). Fluorine-free electrolytes in batteries:
principles, strategies, and advances. Energy
Environ. Sci..

[244] Bezabh H. K., Chiou J.-C., Nigatu T. A., Hagos T. M., Jiang S.-K., Nikodimos Y., Taklu B. W., Tsai M.-C., Su W.-N., Hwang B. J. (2023). In-Depth Insight into a Passive Film
through Hydrogen-Bonding Network in an Aqueous Zinc Battery. ACS Appl. Mater. Interfaces.

[245] Oreiro S. N., Bentien A., Sloth J., Rahimi M., Madsen M. B., Drechsler T. (2024). Crossover
analysis in a commercial
6 kW/43kAh vanadium redox flow battery utilizing anion exchange membrane. Chem. Eng. J..

[246] Tekaligne T. M., Bezabh H. K., Merso S. K., Shitaw K. N., Weret M. A., Nikodimos Y., Jiang S.-K., Yang S.-C., Wang C.-H., Wu S.-H. (2024). Enhancing aluminum foil
performance in aqueous and organic electrolytes: dual-secure passivation
with phthalocyanine as a corrosion inhibitor. J. Mater. Chem. A.

[247] Wang Y., Li Y., Wang X., Gao Y., Li C., Meng T., Zhang H., Chee P. S., Makhlouf S. A., Guan C. (2025). Shape-Controlled Reversible Li Plating-Stripping
for Stable and High-Rate
Anode-Free Lithium Metal Batteries. Adv. Mater..

[248] Li W., Wang D. (2025). Conversion-type cathode
materials for aqueous Zn metal
batteries in nonalkaline aqueous electrolytes: progress, challenges,
and solutions. Adv. Mater..

[249] Li A., Liu H., Wang Z., Liu X., Wu X., Li H., Wang L. (2025). Dual-Anion Reinforced
Cathode-Electrolyte Interphase
for Stable Conversion-Type Cathode. ACS Appl.
Mater. Interfaces.

[250] Li Q., Liu H., Ye Y., Li K. J., Wu F., Li L., Chen R. (2025). The critical
importance of stack pressure in batteries. Nat.
Energy.

[251] Li D., Shen C., Zheng Y., Xu J. (2025). Electrochemo-Mechanical
Degradation and Failure of Active Particles in High Energy Density
Batteries: A Review. Small.

[252] Shitaw K. N., Weret M. A., Nikodimos Y., Tekaligne T. M., Jiang S.-K., Huang C.-J., Lin B.-H., Wu S.-H., Su W.-N., Hwang B. J. (2024). Fundamental phenomena
in anode-free coin cells and pouch cells configured with imide salt-based
ether electrolytes. Mater. Today Energy.

[253] Li S., Jiang M., Xie Y., Xu H., Jia J., Li J. (2018). Developing high-performance lithium
metal anode in liquid electrolytes:
challenges and progress. Adv. mater..

[254] Ren X., Zou L., Cao X., Engelhard M. H., Liu W., Burton S. D., Lee H., Niu C., Matthews B. E., Zhu Z. (2019). Enabling high-voltage
lithium-metal batteries under
practical conditions. Joule.

[255] Xu K. (2014). Electrolytes and interphases in Li-ion
batteries and beyond. Chem. Rev..

[256] Chao D., Zhu C., Yang P., Xia X., Liu J., Wang J., Fan X., Savilov S. V., Lin J., Fan H. J. (2016). Array of nanosheets render ultrafast and high-capacity
Na-ion storage by tunable pseudocapacitance. Nat. commun..

[257] Soto F. A., Ma Y., Martinez de la Hoz J. M., Seminario J. M., Balbuena P. B. (2015). Formation and growth mechanisms of
solid-electrolyte interphase layers in rechargeable batteries. Chem. Mater..

[258] Chen S., Yu Z., Gordin M. L., Yi R., Song J., Wang D. (2017). A fluorinated ether electrolyte enabled
high performance prelithiated graphite/sulfur batteries. ACS Appl. Mater. Interfaces.

[259] Koch V., Young J. (1979). 2-Methyltetrahydrofuran—lithium
hexafluoroarsenate: a superior electrolyte for the secondary lithium
electrode. Science.

[260] Abraham K., Goldman J., Natwig D. (1982). Characterization
of
ether electrolytes for rechargeable lithium cells. J. Electrochem. Soc..

[261] Chen X., Shen X., Li B., Peng H. J., Cheng X. B., Li B. Q., Zhang X. Q., Huang J. Q., Zhang Q. (2018). Ion-solvent
complexes promote gas evolution from electrolytes on
a sodium metal anode. Angew. Chem., Int. Ed..

[262] Zhang L., Tsolakidou C., Mariyappan S., Tarascon J.-M., Trabesinger S. (2021). Unraveling
gas evolution in sodium
batteries by online electrochemical mass spectrometry. Energy Storage Mater..

[263] Berhe G. B., Su W.-N., Hagos T. T., Bezabh H. K., Hagos T. M., Hwang B. J. (2023). Partially fluorinated electrolyte
for high-voltage cathode with sulfurized carbon anode from polyacrylonitrile
for lithium-ion battery. J. Power Sources.

[264] Hossain M. F., Abrha L. H., Razzaq A. A., Ogilvie R., Dirks W. C., Studer H., Poches C., Lama B., Oli H., Bandlamudi S. R. (2025). Mechanistic Study of
Functional Electrolyte Solvents for High-Voltage Lithium Batteries. ACS Appl. Mater. Interfaces.

[265] Eshetu G. G., Elia G. A., Armand M., Forsyth M., Komaba S., Rojo T., Passerini S. (2020). Electrolytes
and interphases in sodium-based rechargeable batteries: recent advances
and perspectives. Adv. Energy Mater..

[266] Li Y., Wu F., Li Y., Liu M., Feng X., Bai Y., Wu C. (2022). Ether-based electrolytes
for sodium ion batteries. Chem. Soc. Rev..

[267] Yin L., Wang M., Xie C., Yang C., Han J., You Y. (2023). High-Voltage Cyclic Ether-Based Electrolytes for Low-Temperature
Sodium-Ion Batteries. ACS Appl. Mater. Interfaces.

[268] Che C., Zhang R., Li Y., Li H., Li S., Qian J., Gong Y., Li H., Bai Y., Wu F. (2025). Fundamental Chemistry and Functional Mechanisms of
Nitrile-Based Electrolyte in Advanced Battery Systems. Adv. Mater..

[269] LI N., MA J., HUANG T., SHEN Y., SHEN M., JIANG Y., HONG T., MA G., MA Z. (2025). Research progress
on nitrile compounds in high potential electrolytes. Energy Storage Sci. Technol..

[270] Senthil C., Subramani A., Gupta R. K., Sofer Z. (2025). Functional
Electrolyte Additives: A Pinch of Salt/Solvent to an Electrolyte for
High Energy Density Lithium-Ion and Lithium-Metal Batteries. Small.

[271] Wang Z., He Z., Wang Z., Yang J., Long K., Wu Z., Zhou G., Mei L., Chen L. (2024). A nitrile solvent structure
induced stable solid electrolyte interphase
for wide-temperature lithium-ion batteries. Chem. Sci..

[272] Dato M. A., Edgington J., Hung C., Sinha R., Liu Z., Lopez J., Guo J., He M., Su C. C. (2024). Sulfur
solutions: advancing high voltage and high energy lithium batteries
with organosulfur electrolytes. Adv. Energy
Mater..

[273] Li Y., Amzil S., Xu T., Xiao Y., Liu X., Ru Z., Wu M., Luo S., Peng M., Tian S. (2025). A new strategy for sulfone-containing
electrolytes design enabling
long cycling high-voltage lithium-ion batteries. Adv. Funct. Mater..

[274] Li B., Chao Y., Li M., Xiao Y., Li R., Yang K., Cui X., Xu G., Li L., Yang C. (2023). A review of solid electrolyte
interphase (SEI) and
dendrite formation in lithium batteries. Electrochem.
Energy Rev..

[275] Lu H., Nagarathinam M., Chen Y., Zhang W., Chen X., Chen J., Tao J., Li J., Lin Y., Kolosov O. (2025). Recent
Advances on Characterization Techniques for
the Composition-Structure-Property Relationships of Solid Electrolyte
Interphase. Small Methods.

[276] Adenusi H., Chass G. A., Passerini S., Tian K. V., Chen G. (2023). Lithium batteries and the solid electrolyte
interphase (SEI)—progress and outlook. Adv. Energy Mater..

[277] Zou Y., Zhang B., Luo H., Yu X., Yang M., Zheng Q., Wang J., Jiao C., Chen Y., Zhang H. (2024). Electrolyte Solvation Engineering Stabilizing Anode-Free
Sodium Metal Battery With 4.0 V-Class Layered Oxide Cathode. Adv. Mater..

[278] Esmaeilpour M., Jana S., Li H., Soleymanibrojeni M., Wenzel W. (2023). A Solution-Mediated Pathway for the Growth of the Solid
Electrolyte Interphase in Lithium-Ion Batteries. Adv. Energy Mater..

[279] Hao C., Zhang X., He Z., Gao M., Liu Y., Pan H., Sun W. (2025). Robust Solid Electrolyte Interphase Engineered by Catalysis
Chemistry Toward Durable Anode-Free Sodium Metal Batteries. Angew. Chem., Int. Ed..

[280] Xu P., Liu Y., Qin M., Huang F., Liang S., Fang G. (2025). Electronic Structure
Regulation Inducing Robust Solid Electrolyte
Interphase for Stable Anode-free Sodium Metal Batteries. Adv. Powder Mater..

[281] Liu X., Dong X., Adenusi H., Wu Y., Passerini S. (2025). Co-solvent
strategy for rechargeable post-lithium metal batteries. Nat. Rev. Chem..

[282] Piao Z., Gao R., Liu Y., Zhou G., Cheng H. M. (2023). A review on regulating Li+ solvation structures in
carbonate electrolytes for lithium metal batteries. Adv. Mater..

[283] Shin W., Zhu L., Jiang H., Stickle W. F., Fang C., Liu C., Lu J., Ji X. (2020). Fluorinated
co-solvent promises Li-S batteries under lean-electrolyte conditions. Mater. Today.

[284] Zhang S. S., Read J. (2011). Partially fluorinated solvent as
a co-solvent for the non-aqueous electrolyte of Li/air battery. J. Power Sources.

[285] Wang Y., Li Z., Hou Y., Hao Z., Zhang Q., Ni Y., Lu Y., Yan Z., Zhang K., Zhao Q. (2023). Emerging electrolytes
with fluorinated solvents for rechargeable lithium-based batteries. Chem. Soc. Rev..

[286] Wang L., Ye Y., Chen N., Huang Y., Li L., Wu F., Chen R. (2018). Development
and challenges of functional
electrolytes for high-performance lithium-sulfur batteries. Adv. Funct. Mater..

[287] Chen K., Shen X., Luo L., Chen H., Cao R., Feng X., Chen W., Fang Y., Cao Y. (2023). Correlating
the solvating power of solvents with the strength of ion-dipole interaction
in electrolytes of lithium-ion batteries. Angew.
Chem..

[288] Zhang G., Li J., Chi S. S., Wang J., Wang Q., Ke R., Liu Z., Wang H., Wang C., Chang J. (2024). Molecular Design of
Competitive Solvation Electrolytes for Practical High-Energy and Long-Cycling
Lithium-Metal Batteries. Adv. Funct. Mater..

[289] Wang C., Wan K., Liu P., Zeng C., Wang S., Huang Y., Zhang Y., Xiao H., Shu C., Liang Z. (2025). Localized High-Concentration
Electrolytes With Semi-Solvated
Hexafluoroisopropyl Methyl Ether Diluent for Wide-Temperature-Range
Lithium Metal Batteries. Angew. Chem..

[290] Yu Z., Wang H., Kong X., Huang W., Tsao Y., Mackanic D. G., Wang K., Wang X., Huang W., Choudhury S. (2020). Molecular design for electrolyte solvents enabling
energy-dense and long-cycling lithium metal batteries. Nat. Energy.

[291] Li Y., Wang J., Wang Y., Wang S., Wu L., Zhou B., Yang D., Jiang L., Kan L., Zhu Q. (2025). Sole-Solvent High-Entropy Electrolyte Realizes Wide-Temperature
and High-Voltage Practical Anode-Free Sodium Pouch Cells. Adv. Mater..

[292] Ge J., Ma C., Zhang Y., Ma P., Zhang J., Xie Z., Wen L., Tang G., Wang Q., Li W. (2025). Edge Electron Effect Induced High-Entropy SEI for Durable Anode-Free
Sodium Batteries. Adv. Mater..

[293] Kim S. C., Wang J., Xu R., Zhang P., Chen Y., Huang Z., Yang Y., Yu Z., Oyakhire S. T., Zhang W. (2023). High-entropy electrolytes
for practical lithium metal batteries. Nat.
Energy.

[294] Mao M., Ji X., Wang Q., Lin Z., Li M., Liu T., Wang C., Hu Y.-S., Li H., Huang X. (2023). Anion-enrichment interface enables high-voltage anode-free
lithium
metal batteries. Nat. Commun..

[295] Zou Y., Zhang B., Luo H., Yu X., Yang M., Zheng Q., Wang J., Jiao C., Chen Y., Zhang H. (2024). Electrolyte Solvation
Engineering Stabilizing Anode-Free
Sodium Metal Battery With 4.0 V-Class Layered Oxide Cathode. Adv. Mater..

[296] Miao L., Wang R., Xin W., Zhang L., Geng Y., Peng H., Yan Z., Jiang D., Qian Z., Zhu Z. (2022). Three-functional ether-based co-solvents
for suppressing water-induced parasitic reactions in aqueous Zn-ion
batteries. Energy Storage Mater..

[297] Li Z., Liao Y., Wang Y., Cong J., Ji H., Huang Z., Huang Y. (2023). A co-solvent
in aqueous electrolyte
towards ultralong-life rechargeable zinc-ion batteries. Energy Storage Mater..

[298] Liu Y., Zhang L., Zhou X., Tang W., Li X., Duan J., Wu Q., Mo X., Yuan G. (2025). Unveiling
the Mechanism of Durable Zinc Metal Anodes Enabled by a Low-Concentration
Glycerol Electrolyte Additive. Langmuir.

[299] Wang M., Yin J., Feng X., Li F., Li Z., Zhang W., Cheng Y., Xu X. (2024). Enhanced electrostatic
shielding effect through incorporation of trace amounts of highly
chelating anions for establishing a more stable electric double layer. J. Mater. Chem. A.

[300] Cao J., Zhang D., Zhang X., Zeng Z., Qin J., Huang Y. (2022). Strategies of regulating
Zn 2+ solvation structures for dendrite-free
and side reaction-suppressed zinc-ion batteries. Energy Environ. Sci..

[301] Zhu X., Ding Y., Wen X., Song C., Pei C., Wang G. (2025). Recent Advances in
Electrolyte Additives for Aqueous Zn Metal Batteries:
Functional Mechanisms, Interfacial Engineering, and Dendrite Suppression
Strategies. Small.

[302] Chen W., Wang Y., Wang F., Zhang Z., Li W., Fang G., Wang F. (2024). Zinc chemistries of hybrid electrolytes
in zinc metal batteries: from solvent structure to interfaces. Adv. Mater..

[303] Sui Y., Ji X. (2024). Electrolyte interphases in aqueous batteries. Angew. Chem., Int. Ed..

[304] Zhou A., Wang H., Hu X., Hu Z., Zhao Y., Zhang B., Huang Y., Cui Y., Cui Y., Li L. (2025). Synergy of In Situ Heterogeneous Interphases
with Hydrogen Bond Reconstruction Enabling Highly Reversible Zn Anode
at- 40° C. Adv. Funct. Mater..

[305] Chen Z., Zhou W., Zhao S., Lou X., Chen S. (2025). In-Situ Construction of Solid Electrolyte Interphases
with Gradient
Zincophilicity for Wide Temperature Zinc Ion Batteries. Adv. Energy Mater..

[306] Ma Z., Xie Z., Liu J., Vatamanu J., Xing L., Li W. (2024). Distinct roles: Co-solvent and additive synergy for expansive electrochemical
range and low-temperature aqueous batteries. Energy Storage Mater..

[307] Hagos T. M., Hagos T. T., Bezabh H. K., Berhe G. B., Abrha L. H., Chiu S.-F., Huang C.-J., Su W.-N., Dai H., Hwang B. J. (2020). Resolving the Phase Instability of a Fluorinated Ether,
Carbonate-Based Electrolyte for the Safe Operation of an Anode-Free
Lithium Metal Battery. ACS Applied Energy Materials.

[308] Fan X., Chen L., Borodin O., Ji X., Chen J., Hou S., Deng T., Zheng J., Yang C., Liou S.-C. (2018). Non-flammable electrolyte enables Li-metal batteries with aggressive
cathode chemistries. Nature Nanotechnol..

[309] Zhou C., Zheng L., He T., Garakani M. A., Abouali S., Shen Y., Chen L., Thangadurai V. (2021). Rational design
of a carbonate-glyme hybrid electrolyte for practical anode-free lithium
metal batteries. Energy Storage Mater..

[310] Duan J., Min L., Wu M., Yang T., Chen M., Wang C. (2022). “Anode-free”
Zn/LiFePO4
aqueous batteries boosted by hybrid electrolyte. J. Ind. Eng. Chem..

[311] Li Y., Wang J., Wang S., Wang Y., Xu Y., Cheng L., Tang M., Wang G., Tian W., Huang W. (2024). Realizing high-areal-capacity anode-free Zn metal batteries. Energy Storage Mater..

[312] Woo J.-J., Maroni V. A., Liu G., Vaughey J. T., Gosztola D. J., Amine K., Zhang Z. (2014). Symmetrical impedance
study on inactivation induced degradation of lithium electrodes for
batteries beyond lithium-ion. J. Electrochem.
Soc..

[313] Seo D. M., Borodin O., Han S.-D., Boyle P. D., Henderson W. A. (2012). Electrolyte
solvation and ionic association II. Acetonitrile-lithium
salt mixtures: highly dissociated salts. J.
Electrochem. Soc..

[314] Beyene T. T., Jote B. A., Wondimkun Z. T., Olbassa B. W., Huang C.-J., Thirumalraj B., Wang C.-H., Su W.-N., Dai H., Hwang B.-J. (2019). Effects
of concentrated salt and resting protocol on solid electrolyte interface
formation for improved cycle stability of anode-free lithium metal
batteries. ACS Appl. Mater. Interfaces.

[315] Kim Y. M., Park B. K., Kang S., Yang S. J., Choi S. H., Yoo D. J., Kim K. J. (2024). Bespoke Dual-Layered
Interface Enabled by Cyclic Ether in Localized High-Concentration
Electrolytes for Lithium Metal Batteries. Adv.
Funct. Mater..

[316] Mao M., Gong L., Wang X., Wang Q., Zhang G., Wang H., Xie W., Suo L., Wang C. (2024). Electrolyte
design combining fluoro-with cyano-substitution solvents for anode-free
Li metal batteries. Proc. Natl. Acad. Sci. U.
S. A..

[317] Yamada Y., Wang J., Ko S., Watanabe E., Yamada A. (2019). Advances and issues in developing salt-concentrated
battery electrolytes. Nat. Energy.

[318] Li M., Wang C., Chen Z., Xu K., Lu J. (2020). New concepts
in electrolytes. Chem. Rev..

[319] Guo Z., Cui Z., Sim R., Manthiram A. (2023). Localized
High-Concentration Electrolytes with Low-Cost Diluents Compatible
with Both Cobalt-Free LiNiO2 Cathode and Lithium-Metal Anode. Small.

[320] Cao X., Jia H., Xu W., Zhang J.-G. (2021). Localized high-concentration
electrolytes for lithium batteries. J. Electrochem.
Soc..

[321] Agnihotri T., Chu T.-H., Jiang S.-K., Ahmed S. A., Ranjan A., Tamilarasan E. B., Yang S.-C., Hagos T. M., Muche Z. B., Jiang J.-C. (2024). Multifunctional fluorinated
phosphonate-based localized high concentration electrolytes for safer
and high-performance lithium-based batteries. Energy Storage Mater..

[322] Zheng X., Huang L., Ye X., Zhang J., Min F., Luo W., Huang Y. (2021). Critical effects of electrolyte recipes
for Li and Na metal batteries. Chem..

[323] Lee J., Lee Y., Lee J., Lee S.-M., Choi J.-H., Kim H., Kwon M.-S., Kang K., Lee K. T., Choi N.-S. (2017). Ultraconcentrated
sodium bis (fluorosulfonyl) imide-based electrolytes for high-performance
sodium metal batteries. ACS Appl. Mater. Interfaces.

[324] Li P., Jiang Z., Huang X., Lu X., Xie J., Cheng S. (2021). Nitrofullerene
as an electrolyte-compatible additive for high-performance
sodium metal batteries. Nano Energy.

[325] Zheng J., Chen S., Zhao W., Song J., Engelhard M. H., Zhang J.-G. (2018). Extremely stable
sodium metal batteries
enabled by localized high-concentration electrolytes. ACS Energy Lett..

[326] Lu Z., Yang H., Yang Q. H., He P., Zhou H. (2022). Building a
Beyond Concentrated Electrolyte for High-Voltage Anode-Free Rechargeable
Sodium Batteries. Angew. Chem..

[327] Zhang G., Wang S., Ma K., Sun C., Wang C., Zhou F., Zhao X., Huang X., Liu Z., Wu N. (2025). Non-flammable phosphate-ester-based electrolyte
with high concentration for high safety potassium-based anode-free
dual ion battery. Chem. Eng. J..

[328] Yuan Z., Liao J., Song L., Chen A., Su J., Wang J., Zhou X. (2025). Entropy-Repaired
Solvation Structure
Strategy for High-Efficiency Phosphate-Based Localized High-Concentration
Electrolytes in Potassium Batteries. Angew.
Chem..

[329] Nandi S., Pumera M. (2025). Anode Free Zinc-Metal Batteries (AFZMBs):
A New Paradigm in Energy Storage. Small.

[330] Wang T., Tang S., Xiao Y., Xiang W., Yu J. S. (2025). Strategies of interfacial chemistry
manipulated zinc deposition towards
high-energy and long-cycle-life aqueous anode-free zinc metal batteries. Energy Environ. Sci..

[331] Zhang Q., Ma Y., Lu Y., Zhou X., Lin L., Li L., Yan Z., Zhao Q., Zhang K., Chen J. (2021). Designing anion-type
water-free Zn2+ solvation structure for robust
Zn metal anode. Angew. Chem..

[332] Tang W., Deng L., Guo L., Zhou S., Jiang Q., Luo J. (2024). Reversible aqueous
aluminum metal
batteries enabled by a water-in-salt electrolyte. Green Energy Environ..

[333] Khan Z., Kumar D., Crispin X. (2023). Does water-in-salt
electrolyte subdue issues of Zn batteries?. Adv. Mater..

[334] Zhang L., Rodríguez-Pérez I. A., Jiang H., Zhang C., Leonard D. P., Guo Q., Wang W., Han S., Wang L., Ji X. (2019). ZnCl2 “Water-in-Salt”
electrolyte transforms the performance of vanadium oxide as a Zn battery
cathode. Adv. Funct. Mater..

[335] Tong Z., Lian R., Yang R., Kang T., Feng J., Shen D., Wu Y., Cui X., Wang H., Tang Y. (2022). An aqueous aluminum-ion
electrochromic full battery with water-in-salt electrolyte for high-energy
density. Energy Storage Mater..

[336] Ma L., Chen S., Long C., Li X., Zhao Y., Liu Z., Huang Z., Dong B., Zapien J. A., Zhi C. (2019). Achieving
high-voltage and high-capacity aqueous rechargeable zinc ion battery
by incorporating two-species redox reaction. Adv. Energy Mater..

[337] Liu Z., Huang Y., Huang Y., Yang Q., Li X., Huang Z., Zhi C. (2020). Voltage issue of aqueous rechargeable
metal-ion batteries. Chem. Soc. Rev..

[338] Smith L., Dunn B. (2015). Opening the window for aqueous electrolytes. Science.

[339] Xiao D., Zhang L., Li Z., Dou H., Zhang X. (2022). Design strategies and research progress for Water-in-Salt electrolytes. Energy Storage Mater..

[340] Reber D., Kühnel R.-S., Battaglia C. (2019). Suppressing
crystallization of water-in-salt electrolytes by asymmetric anions
enables low-temperature operation of high-voltage aqueous batteries. ACS Materials Lett..

[341] Dhasarathaboopathy M., Sabhapathy P., Gurkan B. (2025). Water-in-bisalt electrolytes
with mixed hydrophilic and hydrophobic anions for enhanced transport
and stability for potassium-ion batteries. RSC
Adv..

[342] Beyene T. T., Jote B. A., Wondimkun Z. T., Olbassa B. W., Huang C.-J., Thirumalraj B., Wang C.-H., Su W.-N., Dai H., Hwang B.-J. (2019). Effects
of concentrated salt and resting protocol on solid electrolyte interface
formation for improved cycle stability of anode-free lithium metal
batteries. ACS Appl. Mater. Interfaces.

[343] Ren X., Zou L., Jiao S., Mei D., Engelhard M. H., Li Q., Lee H., Niu C., Adams B. D., Wang C. (2019). High-concentration ether electrolytes for stable high-voltage lithium
metal batteries. ACS Energy letters.

[344] Li P., Zhang Z., Zhao Z., Zhang X., Zhang H., Li G. (2024). Localized medium concentration
electrolyte with fast kinetics for
lithium metal batteries. Angew. Chem. Int. Ed..

[345] Hagos T. T., Su W.-N., Huang C.-J., Thirumalraj B., Chiu S.-F., Abrha L. H., Hagos T. M., Bezabh H. K., Berhe G. B., Tegegne W. A., Cherng J.-Y., Yang Y.-W., Hwang B.-J. (2020). Developing high-voltage carbonate-ether mixed electrolyte
via anode-free cell configuration. J. Power
Sources.

[346] Lim H.-S., Nguyen D. T., Lochala J. A., Cao X., Zhang J.-G. (2024). Improving cycling performance of anode-free lithium
batteries by pressure and voltage control. ACS
Energy Letters.

[347] Su L., Charalambous H., Cui Z., Manthiram A. (2022). High-efficiency,
anode-free lithium-metal batteries with a close-packed homogeneous
lithium morphology. Energy Environ. Sci..

[348] Asano H., Liu J., Ueno K., Dokko K., Kojima T., Takeichi N., Miyuki T., Yamakawa Y., Watanabe M. (2023). Enhancing the reversibility
of Li deposition/dissolution
in sulfur batteries using high-concentration electrolytes to develop
anode-less batteries with lithium sulfide cathode. J. Power Sources.

[349] Zhao J., Tang M., Lan H., Zhu Q., Wang G., Yang G., Yang J., Zhou W., Wang H. (2024). An anode-free
sodium dual-ion battery. Energy
Storage Mater..

[350] Nanda S., Manthiram A. (2021). Delineating the lithium-electrolyte
interfacial chemistry and the dynamics of lithium deposition in lithium-sulfur
batteries. Adv. Energy Mater..

[351] Alpen U. (1979). Li3N: A promising Li ionic conductor. J. Solid
State Chem..

[352] Miao Z., Yu H., Xia M., Zheng R., Yang Z., Liu Y., Zhang L., Shu J. (2021). An anode-free
aqueous dual-ion battery. Sustainable Energy
Fuels.

[353] Cao L., Li D., Pollard T., Deng T., Zhang B., Yang C., Chen L., Vatamanu J., Hu E., Hourwitz M. J. (2021). Fluorinated interphase enables reversible aqueous
zinc battery chemistries. Nat. Nanotechnol..

[354] Xu Y., Zheng X., Sun J., Wang W., Wang M., Yuan Y., Chuai M., Chen N., Hu H., Chen W. (2022). Nucleophilic interfacial
layer enables stable Zn anodes for aqueous
Zn batteries. Nano Lett..

[355] Jiang H., Chen Z., Yang Y., Fan C., Zhao J., Cui G. (2023). Rational Design of Functional Electrolytes
Towards Commercial Dual-Ion Batteries. ChemSusChem.

[356] Ou X., Gong D., Han C., Liu Z., Tang Y. (2021). Advances and
prospects of dual-ion batteries. Adv. Energy
Mater..

[357] Beyene T. T., Bezabh H. K., Weret M. A., Hagos T. M., Huang C.-J., Wang C.-H., Su W.-N., Dai H., Hwang B.-J. (2019). Concentrated
dual-salt electrolyte to stabilize Li
metal and increase cycle life of anode free Li-metal batteries. J. Electrochem. Soc..

[358] Beyene T. T., Su W.-N., Hwang B. J. (2022). Dilute
dual-salt
electrolyte for successful passivation of in-situ deposited Li anode
and permit effective cycling of high voltage anode free batteries. J. Power Sources.

[359] Tang K., Tian L., Zhang Y., Xu Z. J. (2024). Anode-free
lithium metal batteries: a promising flexible energy storage system. J. Mater. Chem. A.

[360] Eshetu G. G., Grugeon S., Kim H., Jeong S., Wu L., Gachot G., Laruelle S., Armand M., Passerini S. (2016). Comprehensive
insights into the reactivity of electrolytes based on sodium ions. ChemSusChem.

[361] Cao D., Ji T., Wei Z., Liang W., Bai R., Burch K. S., Geiwitz M., Zhu H. (2023). Enhancing lithium stripping
efficiency in anode-free solid-state batteries through self-regulated
internal pressure. Nano Lett..

[362] Teng W., Wu J., Liang Q., Deng J., Xu Y., Liu Q., Wang B., Ma T., Nan D., Liu J. (2023). Designing advanced liquid electrolytes for alkali metal
batteries: Principles, progress, and perspectives. Energy Environ. Mater..

[363] Miyazaki K., Takenaka N., Watanabe E., Yamada Y., Tateyama Y., Yamada A. (2020). First-principles study
on the cation-dependent
electrochemical stabilities in Li/Na/K hydrate-melt electrolytes. ACS Appl. Mater. Interfaces.

[364] Wang J., Li G., Wang Q., Huang L., Gan X., Li M., Song Z. (2023). Influence
of alkali metal ions (Li+,
Na+, and K+) on the redox thermodynamics and kinetics of organic electrode
materials for rechargeable batteries. Energy
Storage Mater..

[365] Driscoll D. M., Lavan S. N., Zorko M., Redfern P. C., Ilic S., Agarwal G., Fister T. T., Assary R. S., Cheng L., Strmcnik D. (2023). Emergent solvation phenomena
in non-aqueous electrolytes with multiple anions. Chem..

[366] Huang Y., Zhao L., Li L., Xie M., Wu F., Chen R. (2019). Electrolytes and electrolyte/electrode interfaces in
sodium-ion batteries: from scientific research to practical application. Adv. Mater..

[367] Zhu B., Gao J., Li S., Zhou W., Yang Z., Jin R., Zhang Y., Ding Z., Lai Y., Li J. (2025). Anion competitive coordination for solvation structure regulation
in high-voltage sodium-ion batteries. Chem.
Eng. J..

[368] Zhang X., Lin X., Xu P., Yuan R., Gupta D., Rupp R., Barozzino-Consiglio G., Xu H., Dong Q., Vlad A. (2022). Strong ion pairing at the origin
of modified Li-cation solvation and improved performances of dual-salt
electrolytes. J. Power Sources.

[369] Jun K., Chen Y., Wei G., Yang X., Ceder G. (2024). Diffusion
mechanisms of fast lithium-ion conductors. Nat.
Rev. Mater..

[370] Wang D., Tang Y., Peng H., Ma G. (2025). Functional
Electrolyte Additives for Aqueous Zinc-Ion Batteries: Progress and
Perspectives. ChemSusChem.

[371] Patil N., de la Cruz C., Ciurduc D., Mavrandonakis A., Palma J., Marcilla R. (2021). An ultrahigh performance zinc-organic
battery using poly (catechol) cathode in Zn (TFSI) 2-based concentrated
aqueous electrolytes. Adv. Energy Mater..

[372] Ferrara C., Dall’Asta V., Berbenni V., Quartarone E., Mustarelli P. (2017). Physicochemical
characterization of AlCl3–1-Ethyl-3-methylimidazolium
chloride ionic liquid electrolytes for aluminum rechargeable batteries. J. Phys. Chem. C.

[373] Schoetz T., Leung O., de Leon C. P., Zaleski C., Efimov I. (2020). Aluminium deposition in EMImCl-AlCl3
ionic liquid and
ionogel for improved aluminium batteries. J.
Electrochem. Soc..

[374] Elterman V., Shevelin P. Y., Yolshina L., Vovkotrub E., Borozdin A. (2020). Effects of AlCl3–1-ethyl-3-methylimidazolium
chloride ionic liquid composition on transport properties. J. Mol. Liq..

[375] Zhang Y., Wang F., Li Y., Xie G., Shen Q., Yu X. (2024). Optimization of urea-1.3 AlCl3 deep
eutectic solvent electrolyte performance by AlF3 addition. Ionics.

[376] Bogolowski N., Drillet J.-F. (2018). Activity of different
AlCl3-based
electrolytes for the electrically rechargeable aluminium-air battery. Electrochim. Acta.

[377] Chu W., Zhang X., Zhao S., Tang M., Li S., Liu S., Yu H. (2024). High-voltage
deep eutectic solvent electrolyte with
fluorine-substituted acetamide additive for aluminum-ion battery. Adv. Funct. Mater..

[378] Yang L., Yang C., Chen Y., Pu Z., Zhang Z., Jie Y., Zheng X., Xiao Y., Jiao S., Li Q. (2021). Hybrid MgCl2/AlCl3/Mg
(TFSI) 2 electrolytes in DME enabling high-rate rechargeable Mg batteries. ACS Appl. Mater. Interfaces.

[379] He Y., Li Q., Yang L., Yang C., Xu D. (2019). Electrochemical-Conditioning-Free
and Water-Resistant Hybrid AlCl3/MgCl2/Mg (TFSI) 2 Electrolytes for
Rechargeable Magnesium Batteries. Angew. Chem..

[380] Song Z., Wang X., Feng W., Armand M., Zhou Z., Zhang H. (2024). Designer anions for better rechargeable
lithium batteries and beyond. Adv. Mater..

[381] Dong D., Zhao C. X., Zhang X., Wang C. (2025). Aqueous Electrolytes:
From Salt in Water to Water in Salt and Beyond. Adv. Mater..

[382] Li Z., Wang L., Huang X., He X. (2024). Lithium bis (trifluoromethanesulfonyl)
imide (LiTFSI): a prominent lithium salt in lithium-ion battery electrolytes-fundamentals,
progress, and future perspectives. Adv. Funct.
Mater..

[383] Li W., He Z., Jie Y., Huang F., Chen Y., Wang Y., Zhang W., Zhu X., Cao R., Jiao S. (2024). Understanding and Design of Cathode-Electrolyte
Interphase in High-Voltage
Lithium-Metal Batteries. Adv. Funct. Mater..

[384] Olbasa B. W., Huang C. J., Fenta F. W., Jiang S. K., Chala S. A., Tao H. C., Nikodimos Y., Wang C. C., Sheu H. S., Yang Y. W. (2022). Highly
reversible Zn metal anode stabilized by dense and anion-derived passivation
layer obtained from concentrated hybrid aqueous electrolyte. Adv. Funct. Mater..

[385] Ai F., Lu Y.-C. (2025). Coordination chemistry in advanced redox-active electrolyte
designs. Nat. Rev. Mater..

[386] Zhang H., Qiao L., Kühnle H., Figgemeier E., Armand M., Eshetu G. G. (2023). From lithium to
emerging mono-and multivalent-cation-based rechargeable batteries:
non-aqueous organic electrolyte and interphase perspectives. Energy Environ. Sci..

[387] Yao Y., Rui X., Bai R., Ouyang Y., Li G., Zhao Y., Zhu Y.-H., Zhao M., Li B.-Q., Zhang X. (2025). Roadmap
for Next-Generation Electrochemical Energy
Storage Technologies: Secondary Batteries and Supercapacitors. ACS Nano.

[388] Damircheli R., Hoang B., Castagna Ferrari V., Lin C.-F. (2023). Fluorinated artificial solid-electrolyte-interphase
layer for long-life sodium metal batteries. ACS Appl. Mater. Interfaces.

[389] Ge J., Zhang Y., Xie Z., Xie H., Chen W., Lu B. (2023). Tailored ZnF2/ZnS-rich interphase
for reversible aqueous Zn batteries. Nano Res..

[390] Rodriguez R., Edison R. A., Stephens R. M., Sun H.-H., Heller A., Mullins C. B. (2020). Separator-free and concentrated LiNO
3 electrolyte cells enable uniform lithium electrodeposition. J. Mater. Chem. A.

[391] Louli A., Eldesoky A., Weber R., Genovese M., Coon M., deGooyer J., Deng Z., White R., Lee J., Rodgers T. (2020). Diagnosing
and correcting anode-free cell
failure via electrolyte and morphological analysis. Nature Energy.

[392] Zhang G., Chang J., Wang L., Li J., Wang C., Wang R., Shi G., Yu K., Huang W., Zheng H. (2023). A monofluoride ether-based
electrolyte solution for fast-charging and low-temperature non-aqueous
lithium metal batteries. Nat. Commun..

[393] Weber R., Genovese M., Louli A., Hames S., Martin C., Hill I. G., Dahn J. (2019). Long cycle
life and
dendrite-free lithium morphology in anode-free lithium pouch cells
enabled by a dual-salt liquid electrolyte. Nature
Energy.

[394] Choi H., Bae Y., Lee S.-M., Ha Y.-C., Shin H.-C., Kim B. G. (2022). A LiPF 6-LiFSI Blended-Salt Electrolyte
System for Improved Electrochemical Performance of Anode-Free Batteries. J. Electrochem. Sci. Technol..

[395] Zhu Q., Yu D., Chen J., Cheng L., Tang M., Wang Y., Li Y., Yang J., Wang H. (2024). A 110 Wh kg-
1 Ah-level anode-free sodium battery at- 40°C. Joule.

[396] Bezabh H. K., Tsai M.-C., Hagos T. T., Beyene T. T., Berhe G. B., Hagos T. M., Abrha L. H., Chiu S.-F., Su W.-N., Hwang B. J. (2020). Roles of film-forming additives in
diluted and concentrated electrolytes for lithium metal batteries:
A density functional theory-based approach. Electrochem. Commun..

[397] Hagos T. M., Bezabh H. K., Redda H. G., Moges E. A., Huang W.-H., Huang C.-J., Su W.-N., Dai H., Hwang B. J. (2021). Exploring
the performance of carbonate and ether-based
electrolytes for anode-free lithium metal batteries operating under
various conditions. J. Power Sources.

[398] Chung C.-H., Wu L.-T., Sentosa D. M., Ho C.-C., Chi P.-W., Hsu W.-C., Yeh K.-W., Chang C.-C., Hwang B. J., Wu M.-K. (2025). Enhancing
Anode-Free
Battery Performance with Self-Healing Single-Ion Conducting PAMPS-co-PBA
Copolymer Interfaces. ACS Appl. Mater. Interfaces.

[399] Bezabh H. K., Chiu S.-F., Hagos T. M., Tsai M.-C., Nikodimos Y., Redda H. G., Su W.-N., Hwang B. J. (2021). Bridging
role of ethyl methyl carbonate in fluorinated electrolyte on ionic
transport and phase stability for lithium-ion batteries. J. Power Sources.

[400] Geng Y., Xin W., Zhang L., Han Y., Peng H., Yang M., Zhang H., Xiao X., Li J., Yan Z. (2024). Building electrode/electrolyte interphases
in aqueous zinc batteries via self-polymerization of electrolyte additives. Natl. Sci. Rev..

[401] Hu A., Liao Z., Yang Q., Huang J., Wei Z., Sun Y., Zhao E., Zhang Z., Yang L., Saito N. (2024). Interphase Optimum via Synergistic Effect of Poly (1, 3-dioxolane)
Electrolytes and Lithium Difluorophosphate for Stable Lithium Metal
Batteries. Adv. Funct. Mater..

[402] Wu B., Chen C., Danilov D. L., Chen Z., Jiang M., Eichel R. A., Notten P. H. (2024). Dual Additives
for Stabilizing Li
Deposition and SEI Formation in Anode-Free Li-Metal Batteries. Energy Environ. Mater..

[403] Assegie A. A., Chung C.-C., Tsai M.-C., Su W.-N., Chen C.-W., Hwang B.-J. (2019). Multilayer-graphene-stabilized
lithium
deposition for anode-Free lithium-metal batteries. Nanoscale.

[404] Assegie A. A., Cheng J.-H., Kuo L.-M., Su W.-N., Hwang B.-J. (2018). Polyethylene oxide film coating enhances
lithium cycling
efficiency of an anode-free lithium-metal battery. Nanoscale.

[405] Sahalie N. A., Assegie A. A., Su W.-N., Wondimkun Z. T., Jote B. A., Thirumalraj B., Huang C.-J., Yang Y.-W., Hwang B.-J. (2019). Effect of bifunctional
additive potassium nitrate on
performance of anode free lithium metal battery in carbonate electrolyte. J. Power Sources.

[406] Hotasi B. T., Hagos T. M., Huang C. J., Jiang S.-K., Jote B. A., Shitaw K. N., Bezabh H. K., Wang C.-H., Su W.-N., Wu S.-H. (2022). Developing ester-based
fluorinated electrolyte with LiPO2F2 as an additive for high-rate
and thermally robust anode-free lithium metal battery. J. Power Sources.

[407] Wang K., Xing L., Zhu Y., Zheng X., Cai D., Li W. (2017). A comparative study of Si-containing electrolyte additives
for lithium ion battery: Which one is better and why is it better. J. power sources.

[408] Liao Y., Yuan L., Liu X., Meng J., Zhang W., Li Z., Huang Y. (2022). Low-cost fumed
silicon
dioxide uniform Li+ flux for lean-electrolyte and anode-free Li/S
battery. Energy Storage Mater..

[409] Panchal R. A., Datta J., Varude V., Bhimani K., Mahajani V., Kamble M., Anjan A., Manoj R. M., Zha R. H., Datta D. (2024). Nano-silica
electrolyte
additive enables dendrite suppression in an anode-free sodium metal
battery. Nano Energy.

[410] Tang M., Dong S., Wang J., Cheng L., Zhu Q., Li Y., Yang X., Guo L., Wang H. (2023). Low-temperature
anode-free potassium metal batteries. Nat. Commun..

[411] Umh H. N., Park J., Yeo J., Jung S., Nam I., Yi J. (2019). Lithium metal anode
on a copper dendritic superstructure. Electrochem.
Commun..

[412] Brown Z. L., Jurng S., Lucht B. L. (2017). Investigation of
the lithium solid electrolyte interphase in vinylene carbonate electrolytes
using Cu|| LiFePO4 cells. J. Electrochem. Soc..

[413] Wondimkun Z. T., Beyene T. T., Weret M. A., Sahalie N. A., Huang C.-J., Thirumalraj B., Jote B. A., Wang D., Su W.-N., Wang C.-H., Brunklaus G., Winter M., Hwang B. J. (2020). Binder-free ultra-thin
graphene oxide
as an artificial solid electrolyte interphase for anode-free rechargeable
lithium metal batteries. J. Power Sources.

[414] Shi J., Koketsu T., Zhu Z., Yang M., Sui L., Liu J., Tang M., Deng Z., Liao M., Xiang J. (2024). In situ
p-block protective layer plating in carbonate-based electrolytes
enables stable cell cycling in anode-free lithium batteries. Nat. Mater..

[415] Chen S., Lan R., Humphreys J., Tao S. (2020). Salt-concentrated acetate electrolytes for a high voltage aqueous
Zn/MnO2 battery. Energy Storage Mater..

[416] Cao J., Zhao F., Guan W., Yang X., Zhao Q., Gao L., Ren X., Wu G., Liu A. (2024). Additives for Aqueous
Zinc-Ion Batteries: Recent Progress, Mechanism Analysis, and Future
Perspectives. Small.

[417] Guo H., Shao Z., Zhang Y., Cui X., Mao L., Cheng S., Ma M., Lan W., Su Q., Xie E. (2022). Electrolyte additives inhibit the surface reaction
of aqueous sodium/zinc
battery. J. Colloid Interface Sci..

[418] Chang L., Cheng H., Li J., Zhang L., Zhang B., Zheng L., Sun Q., Li J., Lu X., Zhao K. (2025). High-entropy solvation chemistry
towards affordable
and practical Ah-level zinc metal battery. Nat.
Commun..

[419] Zhang R., Cui Y., Liu L., Chen S. (2024). A dual-functional
rare earth halide additive for high-performance aqueous zinc ion batteries. J. Power Sources.

[420] Zhang Y., Wang L., Li Q., Hu B., Kang J., Meng Y., Zhao Z., Lu H. (2022). Iodine promoted
ultralow Zn nucleation overpotential and Zn-rich cathode for low-cost,
fast-production and high-energy density anode-free Zn-iodine batteries. Nanomicro Lett..

[421] Shi W., Song Z., Zhang W., Lian S., Huang F., An Q., Li Q. (2024). Identifying iodide-ion
regulation of early-stage zinc
nucleation and growth for high-rate anode-free zinc metal batteries. Energy Environ. Sci..

[422] Tsai H.-Y., Kumar M. S., Vedhanarayanan B., Shen H.-H., Lin T.-W. (2023). Urea-based deep eutectic solvent
with magnesium/lithium dual ions as an aqueous electrolyte for high-performance
battery-supercapacitor hybrid devices. Batteries.

[423] Lin T.-W., Kumar M. S., Shen H.-H., Lin J.-Y. (2024). Acetamide-based
deep eutectic solvents as efficient electrolytes for K-MnHCFe//Zn
dual-ion batteries. J. Power Sources.

[424] Li C., Kingsbury R., Thind A. S., Shyamsunder A., Fister T. T., Klie R. F., Persson K. A., Nazar L. F. (2023). Enabling
selective zinc-ion intercalation by a eutectic electrolyte for practical
anodeless zinc batteries. Nat. commun..

[425] Nigatu T. A., Bezabh H. K., Jiang S.-K., Taklu B. W., Nikodimos Y., Yang S.-C., Wu S.-H., Su W.-N., Yang C.-C., Hwang B. J. (2023). An anode-free aqueous
hybrid batteries
enabled by in-situ Cu/Sn/Zn alloy formation on pure Cu substrate. Electrochim. Acta.

[426] Wu H., Jia H., Wang C., Zhang J. G., Xu W. (2021). Recent progress
in understanding solid electrolyte interphase on lithium metal anodes. Adv. Energy Mater..

[427] Zhang H., Eshetu G. G., Judez X., Li C., Rodriguez-Martínez L. M., Armand M. (2018). Electrolyte additives
for lithium metal anodes and rechargeable lithium metal batteries:
progress and perspectives. Angew. Chem..

[428] Magnussen O. M. (2002). Ordered
anion adlayers on metal electrode surfaces. Chem. Rev..

[429] Ghosh S., Manna L. (2018). The many “facets” of
halide ions in the chemistry of colloidal inorganic nanocrystals. Chem. Rev..

[430] Huang G., Liao Y., Zhao X., Jin X., Zhu Z., Guan M., Li Y. (2023). Tuning a solvation
structure of lithium
ions coordinated with nitrate anions through ionic liquid-based solvent
for highly stable lithium metal batteries. Adv.
Funct. Mater..

[431] Hou T., Yang G., Rajput N. N., Self J., Park S.-W., Nanda J., Persson K. A. (2019). The influence of FEC on the solvation
structure and reduction reaction of LiPF6/EC electrolytes and its
implication for solid electrolyte interphase formation. Nano Energy.

[432] Brown Z. L., Jurng S., Lucht B. L. (2017). Investigation of
the lithium solid electrolyte interphase in vinylene carbonate electrolytes
using Cu|| LiFePO4 cells. J. Electrochem. Soc..

[433] Wu B., Chen C., Danilov D. L., Chen Z., Jiang M., Eichel R. A., Notten P. H. (2024). Dual Additives
for Stabilizing Li
Deposition and SEI Formation in Anode-Free Li-Metal Batteries. Energy & Environmental Materials.

[434] Zhou M., Liu W., Su Q., Zeng J., Jiang X., Wu X., Chen Z., Wang X., Li Z., Liu H. (2024). Ionic
Liquid Additive Mitigating Lithium Loss
and Aluminum Corrosion for High-Voltage Anode-Free Lithium Metal Batteries. ACS Nano.

[435] Sahalie N. A., Assegie A. A., Su W.-N., Wondimkun Z. T., Jote B. A., Thirumalraj B., Huang C.-J., Yang Y.-W., Hwang B.-J. (2019). Effect of bifunctional
additive potassium nitrate on
performance of anode free lithium metal battery in carbonate electrolyte. J. Power Sources.

[436] Zhang J., Zhang H., Deng L., Yang Y., Tan L., Niu X., Chen Y., Zeng L., Fan X., Zhu Y. (2023). An additive-enabled
ether-based electrolyte to realize stable cycling
of high-voltage anode-free lithium metal batteries. Energy Storage Materials.

[437] Yen Y.-J., Manthiram A. (2024). Anode-Free
Lithium-Sulfur Batteries
with a Rare-Earth Triflate as a Dual-Function Electrolyte Additive. ACS Appl. Mater. Interfaces.

[438] Ren Y., Bhargav A., Shin W., Sul H., Manthiram A. (2022). Anode-free
lithium-sulfur cells enabled by rationally tuning lithium polysulfide
molecules. Angew. Chem., Int. Ed..

[439] Lai T., Bhargav A., Manthiram A. (2023). Lithium Tritelluride
as an Electrolyte
Additive for Stabilizing Lithium Deposition and Enhancing Sulfur Utilization
in Anode-Free Lithium-Sulfur Batteries. Adv.
Funct. Mater..

[440] Zhu Q., Wang J., Wu L., Lan H., Chen J., Cheng L., Zhou B., Yang D., Yang J., Kurbanov M. (2025). A> 200 Wh kg- 1 anode-free Na pouch battery
at- 40° C enabled by manipulating electrolyte equilibrium. Natl. Sci. Rev..

[441] Sundari C. D. D., Ivansyah A. L., Floweri O., Arcana I. M., Iskandar F. (2022). Insights into the intermolecular interactions and temperature-concentration
dependence of transport in ionic liquid-based EMI-TFSI/LiTFSI electrolytes. New J. Chem..

[442] Liu X., Mariani A., Adenusi H., Passerini S. (2023). Locally concentrated
ionic liquid electrolytes for lithium-metal batteries. Angew. Chem., Int. Ed..

[443] Zhou W., Zhang M., Kong X., Huang W., Zhang Q. (2021). Recent advance in ionic-liquid-based
electrolytes for rechargeable
metal-ion batteries. Adv. Sci..

[444] Kim J.-K., Matic A., Ahn J.-H., Jacobsson P. (2010). An imidazolium
based ionic liquid electrolyte for lithium batteries. J. Power Sources.

[445] Yang B., Li C., Zhou J., Liu J., Zhang Q. (2014). Pyrrolidinium-based ionic liquid electrolyte with organic
additive
and LiTFSI for high-safety lithium-ion batteries. Electrochim. acta.

[446] Hilder M., Howlett P. C., Saurel D., Gonzalo E., Basile A., Armand M., Rojo T., Kar M., MacFarlane D. R., Forsyth M. (2018). The effect of cation
chemistry on
physicochemical behaviour of superconcentrated NaFSI based ionic liquid
electrolytes and the implications for Na battery performance. Electrochim. Acta.

[447] Pathirana T., Rakov D. A., Chen F., Forsyth M., Kerr R., Howlett P. C. (2021). Improving cycle
life through fast
formation using a superconcentrated phosphonium based ionic liquid
electrolyte for anode-free and lithium metal batteries. ACS Appl. Energy Mater..

[448] Pathirana T., Kerr R., Forsyth M., Howlett P. C. (2021). Application
of super-concentrated phosphonium based ionic liquid electrolyte for
anode-free lithium metal batteries. Sustainable
Energy Fuels.

[449] Guo D., Wang J., Cui Z., Shi Z., Henkelman G., Alshareef H. N., Manthiram A. (2024). Low-temperature sodium-sulfur batteries
enabled by ionic liquid in localized high concentration electrolytes. Adv. Funct. Mater..

[450] Li N., Wang J., Zhang Q., Zhou X., Wang H., Lu G., Zhao J., Chen Z., Cui G. (2023). Enhanced Zn2+ Transport
in Ionic Liquid Electrolyte by Hydrofluoroether Dilution for High-Power
and Long-Life Zn/Graphite Cells. Batter. Supercaps.

[451] Jeremias S., Giffin G. A., Moretti A., Jeong S., Passerini S. (2014). Mechanisms of magnesium ion transport
in pyrrolidinium
bis (trifluoromethanesulfonyl) imide-based ionic liquid electrolytes. J. Phys. Chem. C.

[452] Borodin O., Giffin G. A., Moretti A., Haskins J. B., Lawson J. W., Henderson W. A., Passerini S. (2018). Insights into
the structure and transport of the lithium, sodium, magnesium, and
zinc bis (trifluoromethansulfonyl) imide salts in ionic liquids. J. Phys. Chem. C.

[453] Zhu N., Zhang K., Wu F., Bai Y., Wu C. (2021). Ionic liquid-based
electrolytes for aluminum/magnesium/sodium-ion batteries. Energy Matter. Adv..

[454] Nikodimos Y., Su W.-N., Shitaw K. N., Jiang S.-K., Abrha L. H., Weret M. A., Merso S. K., Hagos T. M., Huang C.-J., Lakshmanan K., Huang W.-H., Chang C.-Y., Lin J.-M., Wu S.-H., Yang C.-C., Hwang B. J. (2023). Multifunctional
Electrospun PVDF-HFP Gel Polymer Electrolyte Membrane Suppresses Dendrite
Growth in Anode-Free Li Metal Battery. Energy
Storage Mater..

[455] Lin Y.-H., Shih C.-Y., Subramani R., Lee Y.-L., Jan J.-S., Chiu C.-C., Teng H. (2022). Ternary-salt
gel polymer electrolyte for anode-free lithium metal batteries with
an untreated Cu substrate. J. Mater. Chem. A.

[456] Redda H. G., Nikodimos Y., Su W.-N., Chen R.-S., Hagos T. M., Bezabh H. K., Weldeyohannes H. H., Hwang B. J. (2022). The surface modification
of electrode materials using
gel polymer electrolytes for anode-free lithium metal batteries (AFLMB). Mater. Today Energy.

[457] Agnihotri T., Ahmed S. A., Tamilarasan E. B., Hasan R., Hotasi B. T., Bezabh H. K., Suwito S., Nikodimos Y., Jiang S.-K., Shitaw K. N., Muche Z. B., Huang P. Y., Lee Y.-C., Su W.-N., Wu S.-H., Hwang B. J. (2024). Anion-trapping
composite gel electrolyte for safer
and more stable anode-free lithium-metal batteries. Chem. Eng. J..

[458] Jabbari V., Yurkiv V., Rasul M. G., Saray M. T., Rojaee R., Mashayek F., Shahbazian-Yassar R. (2022). An efficient
gel polymer electrolyte for dendrite-free and long cycle life lithium
metal batteries. Energy Storage Mater..

[459] Nikodimos, Y. ; Su, W.-N. ; Hwang, B.-J. In Lithium Dendrite Growth Suppression in Anode-Free Lithium Battery Using Bifunctional Electrospun Gel Polymer Electrolyte Membrane; Electrochemical Society Meeting Abstracts 243; The Electrochemical Society, Inc., 2023; pp 998–998.

[460] Asgedom, Y. N. ; Su, W.-N. ; Hwang, B. J. In Novel Gel Polymer Electrolyte Preparation Method for Anode-Free Lithium Metal Battery; Electrochemical Society Meeting Abstracts 247; The Electrochemical Society, Inc., 2025; pp 194–194.

[461] Liu Y., Meng X., Wang Z., Qiu J. (2022). Development of quasi-solid-state
anode-free high-energy lithium sulfide-based batteries. Nat. Commun..

[462] Sen S., Goodwin S. E., Barbara P. V., Rance G. A., Wales D., Cameron J. M., Sans V., Mamlouk M., Scott K., Walsh D. A. (2021). Gel-polymer electrolytes based on
poly (ionic liquid)/ionic
liquid networks. ACS Appl. Polym. Mater..

[463] Wang X., Zhu H., Girard G. M., Yunis R., MacFarlane D. R., Mecerreyes D., Bhattacharyya A. J., Howlett P. C., Forsyth M. (2017). Preparation
and characterization
of gel polymer electrolytes using poly (ionic liquids) and high lithium
salt concentration ionic liquids. J. Mater.
Chem. A.

[464] Pal P., Ghosh A. (2018). Solid-state gel polymer electrolytes based on ionic
liquids containing imidazolium cations and tetrafluoroborate anions
for electrochemical double layer capacitors: Influence of cations
size and viscosity of ionic liquids. J. Power
Sources.

[465] Ngai K. S., Ramesh S., Ramesh K., Juan J. C. (2016). A review
of polymer electrolytes: fundamental, approaches and applications. Ionics.

[466] Zhou T., Zhao Y., Choi J. W., Coskun A. (2021). Ionic liquid
functionalized gel polymer electrolytes for stable lithium metal batteries. Angew. Chem..

[467] Gabryelczyk A., Swiderska-Mocek A. (2024). Tailoring
the Properties of Gel Polymer
Electrolytes for Sodium-Ion Batteries Using Ionic Liquids: A Review. Chem. Eur. J..

[468] Pan J., Zhao P., Yao H., Hu L., Fan H. J. (2024). Inert filler
selection strategies in Li-ion gel polymer electrolytes. ACS Appl. Mater. Interfaces.

[469] Aruchamy K., Ramasundaram S., Divya S., Chandran M., Yun K., Oh T. H. (2023). Gel polymer
electrolytes: advancing solid-state batteries
for high-performance applications. Gels.

[470] Yim T., Kim K., Hassig M. Q., Nedsaengtip J., Zhang T., Jamkar S., Li C. Y., Kalra V. (2025). Lithium-Nitrate-Containing
Gel Polymer Electrolyte for Carbonate-Based Anode-Free Lithium Metal
Batteries. ACS appl. mater. interfaces.

[471] Zhao C., Pan Y., Li R., Hu A., Zhou B., He M., Chen J., Yan Z., Fan Y., Chen N. (2023). A safe anode-free lithium metal pouch cell
enabled by integrating stable quasi-solid electrolytes with oxygen-free
cathodes. Chem. Eng. J..

[472] Wang T., Xiao Y., Tang S., Xiang W., Yu J. S. (2025). Unlocking Quasi-Solid-State Anode-Free Zinc Metal Batteries Through
Robust Bilayer Interphase Engineering. Adv.
Energy Mater..

[473] Qian L., Zheng Y., Or T., Park H. W., Gao R., Park M., Ma Q., Luo D., Yu A., Chen Z. (2022). Advanced Material Engineering to
Tailor Nucleation and Growth towards
Uniform Deposition for Anode-Less Lithium Metal Batteries. Small.

[474] Müller A., Paravicini L., Morzy J., Krause M., Casella J., Osenciat N., Futscher M. H., Romanyuk Y. E. (2024). Influence
of Au, Pt, and C Seed layers on lithium nucleation dynamics for anode-free
solid-state batteries. ACS Appl. Mater. Interfaces.

[475] Xu P., Huang F., Sun Y., Lei Y., Cao X., Liang S., Fang G. (2024). Anode-free
alkali metal batteries:
from laboratory to practicability. Adv. Funt.
Mater..

[476] Zhou J., Qin J., Zhan H. (2024). Copper Current Collector:
The Cornerstones of Practical Lithium Metal and Anode-Free Batteries. ChemPhysChem.

[477] Hu Z., Liu L., Wang X., Lu H., Zheng Q., Gao Y., Wang J., Qi Y., Han C., Li W. (2025). In Situ Integration
of Rapid Ion-Diffusion Interlayers on Cu Current Collectors toward
Ultrafast Anode-Free Sodium Metal Batteries. ACS Nano.

[478] Qi Y., Liu J., Feng M., Tantratian K., Chen L., Xiao X., Sachdev A. K. (2025). From the
Passivation
Layer on Aluminum to Lithium Anode in Batteries. Metall. Mater. Trans. A.

[479] Cooper E. R., Li M., Gentle I., Xia Q., Knibbe R. (2023). A deeper understanding of metal nucleation and growth
in rechargeable metal batteries through theory and experiment. Angew. Chem..

[480] Shitaw K. N., Tekaligne T. M., Jiang S.-K., Huang C.-J., Wu S.-H., Su W.-N., Hwang B. J. (2023). Opportunities of
liquid metals and liquid metal cations for Li-metal batteries. Chem. Eng. J..

[481] Yu H., Sun P., Cheng H., Ding Z., Luo D. (2025). Ag-Cu Alloy
Induces Uniform Lithium Nucleation and Growth in Anode-Free Lithium
Metal Batteries. Electrochim. Acta.

[482] Wondimkun Z. T., Tegegne W. A., Shi-Kai J., Huang C.-J., Sahalie N. A., Weret M. A., Hsu J.-Y., Hsieh P.-L., Huang Y.-S., Wu S.-H., Su W.-N., Hwang B. J. (2021). Highly-lithiophilic
Ag@ PDA-GO film to Suppress Dendrite Formation on Cu Substrate in
Anode-free Lithium Metal Batteries. Energy Storage
Mater..

[483] Merso S. K., Tekaligne T. M., Weldeyohannes H. H., Nikodimos Y., Shitaw K. N., Jiang S.-K., Huang C.-J., Wondimkun Z. T., Jote B. A., Wichmann L. (2022). An in-situ
formed bifunctional layer for suppressing Li dendrite growth and stabilizing
the solid electrolyte interphase layer of anode free lithium metal
batteries. J. Energy Storage.

[484] Chen J., Xiang J., Chen X., Yuan L., Li Z., Huang Y. (2020). Li2S-based anode-free full batteries with modified
Cu current collector. Energy Storage Mater..

[485] Cheng H., Gao C., Cai N., Wang M. (2021). Ag coated
3D-Cu foam as a lithiophilic current collector for enabling Li 2 S-based
anode-free batteries. Chem. Commun..

[486] Lee M. E., Lee S., Choi J., Jin H. J., Han S., Yun Y. S. (2019). Anode-free
sodium metal batteries based on nanohybrid
core-shell templates. Small.

[487] Tang S., Zhang Y. Y., Zhang X. G., Li J. T., Wang X. Y., Yan J. W., Wu D. Y., Zheng M. S., Dong Q. F., Mao B. W. (2019). Stable Na plating
and stripping electrochemistry
promoted by in situ construction of an alloy-based sodiophilic interphase. Adv. Mater..

[488] Wang H., Matios E., Wang C., Luo J., Lu X., Hu X., Zhang Y., Li W. (2019). Tin nanoparticles embedded
in a carbon buffer layer as preferential nucleation sites for stable
sodium metal anodes. J. Mater. Chem. A.

[489] Tang S., Qiu Z., Wang X.-Y., Gu Y., Zhang X.-G., Wang W.-W., Yan J.-W., Zheng M.-S., Dong Q.-F., Mao B.-W. (2018). A room-temperature
sodium metal anode
enabled by a sodiophilic layer. Nano Energy.

[490] Wang Y., Dong H., Katyal N., Hao H., Liu P., Celio H., Henkelman G., Watt J., Mitlin D. (2022). A sodium-antimony-telluride
intermetallic allows sodium-metal cycling at 100% depth of discharge
and as an anode-free metal battery. Adv. Mater..

[491] Bai M., Tang X., Liu S., Wang H., Liu Y., Shao A., Zhang M., Wang Z., Ma Y. (2022). An anodeless,
mechanically flexible and energy/power dense sodium battery prototype. Energy Environ. Sci..

[492] Xie C., Wu H., Dai J., Fu Z., Zhang R., Ji H., Zhang Q., Tang Y., Qiu T., Wang H. (2024). Robust Anode-Free
Sodium Batteries with Durably Sodophilic Interfaces by Suppressing
Sodium-Alloy Transformation. Adv. Energy Mater..

[493] Guo W., Liu X., Mu Y., Yue G., Liu J., Zhu K., Cui Z., Wang N., Chen Z., Zhao Y. (2024). Outside-in
Directional Sodium Deposition Through Self-Supporting Gradient Fluorinated
Magnesium Alloy Framework toward High-Rate Anode-Free Na Batteries. Energy Storage Mater..

[494] Liu M., Zhang S., van Eck E. R., Wang C., Ganapathy S., Wagemaker M. (2022). Improving Li-ion interfacial transport in hybrid solid
electrolytes. Nat. nanotechnol..

[495] Zheng X., Liu Z., Sun J., Luo R., Xu K., Si M., Kang J., Yuan Y., Liu S., Ahmad T. (2023). Constructing robust heterostructured interface for
anode-free zinc
batteries with ultrahigh capacities. Nat. Commun..

[496] Li C., Liang L., Liu X., Cao N., Shao Q., Zou P., Zang X. (2023). A lean-zinc anode battery
based on metal-organic framework-derived
carbon. Carbon Energy.

[497] Xie S., Li Y., Dong L. (2023). Stable anode-free
zinc-ion batteries
enabled by alloy network-modulated zinc deposition interface. J. Energy Chem..

[498] Li C., Shao Q., Luo K., Gao Y., Zhao W., Cao N., Du S., Jin X., Zou P., Zang X. (2023). A Lean-Zinc
and Zincophilic Anode for Highly Reversible Zinc Metal Batteries. Adv. Funct. Mater..

[499] Ling W., Yang Q., Mo F., Lei H., Wang J., Jiao Y., Qiu Y., Chen T., Huang Y. (2022). An ultrahigh rate dendrite-free Zn metal deposition/striping enabled
by silver nanowire aerogel with optimal atomic affinity with Zn. Energy Storage Mater..

[500] Li Y., Yang G., Sun S., Zhang C., Lim C. Y. J., Wong A. J. Y., Lieu W. Y., Sofer Z., Ng M.-F., Liu W. (2022). High utilization of
composite magnesium metal anodes
enabled by a magnesiophilic coating. Nano Lett..

[501] Kwak J. H., Jeoun Y., Oh S. H., Yu S., Lim J.-H., Sung Y.-E., Yu S.-H., Lim H.-D. (2022). Operando
visualization of morphological evolution in Mg metal anode: insight
into dendrite suppression for stable Mg metal batteries. ACS Energy Lett..

[502] Liu J., Zhang J., Zhang Z., Du A., Dong S., Zhou Z., Guo X., Wang Q., Li Z., Li G. (2022). Epitaxial electrocrystallization of magnesium
via synergy
of magnesiophilic interface, lattice matching, and electrostatic confinement. ACS Nano.

[503] Lu C., Zhao F., Tao B., Wang Z., Wang Y., Sheng J., Tang G., Wang Y., Guo X., Li J. (2024). Anode-Free
Aqueous Aluminum Ion Batteries. Small.

[504] Xia H.-y., Wang Y.-k., Fu Z.-w. (2023). Growing
cuprite
nanoparticles on copper current collector toward uniform Li deposition
for anode-free lithium batteries. Appl. Surf.
Sci..

[505] Cheng X., Li D., Peng S., Shi P., Yu H., Jiang Y., Li S. (2023). In-Situ alloy-modified
Sodiophilic
current collectors for Anode-less Sodium Metal batteries. Batteries.

[506] Chen X., Zhou X., Yang Z., Hao Z., Chen J., Kuang W., Shi X., Wu X., Li L., Chou S.-L. (2024). A conductive and sodiophilic Ag coating layer regulating
Na deposition behaviors for highly reversible sodium metal batteries. Chem. Sci..

[507] Liu L., Cai Z., Yang S., Yang Y., Yao Y., He S., Xu S., Wu Z., Pan H., Rui X. (2025). Multifunctional High-Entropy
Alloy Nanolayer Toward Long-Life Anode-Free
Sodium Metal Battery. Adv. Mater..

[508] Ji H., Xie C., Zhang R., Wu H., Dai J., Li S., Zhang Q., Sun D., Tang Y., Wang P. (2025). Sodiophilic Interface
and Electrolyte Regulation Boost the Lifespan
of Anode-Free Sodium Battery. SusMat.

[509] Shi J., Wang D., Liu Q., Yu Z., Huang J.-Q., Zhang B. (2025). Intermetallic Layers with Tuned Na
Nucleation and Transport for Anode-Free
Sodium Metal Batteries. Nano Lett..

[510] Xie C., Liang K., Wu H., Xie Z., Ren Y., Dai J., Lu J., Tang Y., Wang H. (2025). Revealing the Formation
Mechanism of Inactive Sodium in Anode-Free Sodium Batteries: Crystal
Mismatch and Weak Lattice Force. Adv. Energy
Mater..

[511] Li M., Gong X., Hu Y., Shuai Y., Zhou Y., Wu M., Huangyang X., Huang J., Wu Y. (2025). A Eutectic Aluminum-Tin
Alloy Substrate for Anode-Free Na Battery. Small.

[512] Zhao Q., Liu W., Chen Y., Chen L. (2022). Ultra-stable
Zn metal batteries with dendrite-free Cu-Sn alloy induced high-quality
composite Zn mesh. Chem. Eng. J..

[513] An Y., Xu B., Tian Y., Shen H., Man Q., Liu X., Yang Y., Li M. (2023). Reversible Zn electrodeposition enabled
by interfacial chemistry manipulation for high-energy anode-free Zn
batteries. Mater. Today.

[514] Jing Y., Meng X., Chen L., Yuan C., Wei H. (2024). Modulation of Interfacial Characteristics
of Copper Electrode by
Electrodeposited Cu@ Ti for High-Performance Anode-Free Zinc Ion Batteries. ACS Appl. Mater. Interfaces.

[515] Ren N., Wang L., Li X., Cao K., He Z., Shao Y., Xiao J., Zhu Y., Pan B., Jiao S. (2024). Design Principles of Mediation Layer for Current
Collectors
Toward High-Performance Anode-Free Potassium-Metal Batteries: A Case
Study of Cu6Sn5 on Copper. Adv. Funct. Mater..

[516] Zhao Q., Zheng J., Deng Y., Archer L. (2020). Regulating
the growth of aluminum electrodeposits: towards anode-free Al batteries. J. Mater. Chem. A.

[517] Meng Y., Wang J., Wang M., Peng Q., Xie Z., Zhu Z., Liu Z., Wang W., Zhang K., Liu H. (2023). Anode-Free
Aluminum Electrode with Ultralong Cycle
Life and High Coulombic Efficiency Exceeding 99.92% Enabled by a Lattice-Matching
Layer. Adv. Energy Mater..

[518] Merso S. K., Tekaligne T. M., Weret M. A., Shitaw K. N., Nikodimos Y., Yang S.-C., Muche Z. B., Taklu B. W., Hotasi B. T., Chang C.-Y. (2024). Multiple protective
layers for suppressing Li dendrite growth and improving the cycle
life of anode-free lithium metal batteries. Chem. Eng. J..

[519] Hubert B., Nikodimos Y., Hwang B. J., Chu J. P. (2024). Ag-coated
3D groove as a study platform in evaluating the throwing power of
electrolytes for Li metal batteries. J. Power
Sources.

[520] Li J., Ma Z., Yang K., Zhao F., Yang H., Wang H., He Y. (2025). Laser-Generated
Au nanoparticles
as lithophilic sites in self-supported film host for anode-free lithium
metal battery. J. Colloid Interface Sci..

[521] Liu X., Liu J., Zhao H., Dong C., Liu F., Li L. (2025). In-situ construction
of high-performance artificial solid electrolyte
interface layer on anode surfaces for anode-free lithium metal batteries. J. Colloid Interface Sci..

[522] Nan W., Li B., Yan S., Dai S. l. (2025). Dynamic interface
layer enables epitaxial Li deposition for anode-free Li metal batteries. J. Phys. Chem. Solids.

[523] Shitaw K. N., Bezabh H. K., Nikodimos Y., Weret M. A., Tekaligne T. M., Merso S. K., Taklu B. W., Jiang S. K., Li C. Y., Liu H. Y. (2025). Potassium
Underpotential Deposition for Defect-Free Lithium Deposition in Anode-Free
Li-Metal Batteries. Small Methods.

[524] Temesgen N. T., Tegegne W. A., Shitaw K. N., Fenta F. W., Nikodimos Y., Taklu B. W., Jiang S.-K., Huang C.-J., Wu S.-H., Su W.-N., Hwang B. J. (2021). Mitigating
dendrite
formation and electrolyte decomposition via functional double layers
coating on copper current collector in anode-free lithium metal battery. J. Taiwan Inst. Chem. Eng..

[525] Zhang Q., Su Y., Shi Z., Yang X., Sun J. (2022). Artificial interphase layer for stabilized
Zn anodes: progress and
prospects. Small.

[526] Sahalie N. A., Wondimkun Z. T., Su W.-N., Weret M. A., Fenta F. W., Berhe G. B., Huang C.-J., Hsu Y.-C., Hwang B. J. (2020). Multifunctional
Properties of Al2O3/Polyacrylonitrile
Composite Coating on Cu to Suppress Dendritic Growth in Anode-Free
Li-Metal Battery. ACS Appl. Energy Mater..

[527] Cho S., Kim D. Y., Lee J. I., Kang J., Lee H., Kim G., Seo D. H., Park S. (2022). Highly reversible lithium host materials
for high-energy-density anode-free lithium metal batteries. Adv. Funct. Mater..

[528] Tamwattana O., Park H., Kim J., Hwang I., Yoon G., Hwang T.-h., Kang Y.-S., Park J., Meethong N., Kang K. (2021). High-Dielectric Polymer Coating for
Uniform Lithium Deposition in Anode-Free Lithium Batteries. ACS Energy Lett..

[529] Luo J., Fang C. C., Wu N. L. (2018). High polarity poly
(vinylidene difluoride)
thin coating for dendrite-free and high-performance lithium metal
anodes. Adv. Energy Mater..

[530] Abrha L. H., Nikodimos Y., Weldeyohannes H. H., Hagos T. T., Wang D.-Y., Huang C.-J., Jiang S.-K., Wu S.-H., Su W.-N., Tsai M.-C. (2021). Effects
of a Thermally Electrochemically Activated β-PVDF Fiber on Suppression
of Li Dendrite Growth for Anode-Free Batteries. ACS Appl. Energy Mater..

[531] Zhao Y., Ye H., Zhang H., Zhao D., Huang L., Lee J. Y. (2022). The beneficial effects
of black phosphorous
modification of the anode current collector in Li-metal free Li2S-based
batteries. Mater. Today Energy.

[532] Cohn A. P., Metke T., Donohue J., Muralidharan N., Share K., Pint C. L. (2018). Rethinking sodium-ion
anodes as nucleation
layers for anode-free batteries. J. Mater. Chem.
A.

[533] Xu Z., Guo Z., Madhu R., Xie F., Chen R., Wang J., Tebyetekerwa M., Hu Y.-S., Titirici M.-M. (2021). Homogenous
metallic deposition regulated by defect-rich skeletons for sodium
metal batteries. Energy Environ. Sci..

[534] Li T., Sun J., Gao S., Xiao B., Cheng J., Zhou Y., Sun X., Jiang F., Yan Z., Xiong S. (2021). Superior sodium metal
anodes enabled by sodiophilic carbonized coconut
framework with 3D tubular structure. Adv. Energy
Mater..

[535] Xie Y., Hu J., Han Z., Wang T., Zheng J., Gan L., Lai Y., Zhang Z. (2020). Encapsulating sodium deposition into
carbon rhombic dodecahedron guided by sodiophilic sites for dendrite-free
Na metal batteries. Energy Storage Mater..

[536] Huang B., Sun S., Wan J., Zhang W., Liu S., Zhang J., Yan F., Liu Y., Xu J., Cheng F. (2023). Ultrahigh nitrogen content
carbon nanosheets for high
stable sodium metal anodes. Adv. Sci..

[537] Liu P., Wang Y., Gu Q., Nanda J., Watt J., Mitlin D. (2020). Dendrite-free potassium
metal anodes in a carbonate
electrolyte. Adv. Mater..

[538] Wang J., Zuo Y., Chen M., Chen K., Chen Z., Lu Z., Si L. (2022). Bifunctional
separator
with a light-weight coating for stable anode-free potassium metal
batteries. Electrochim. Acta.

[539] Si L., Wang J., Chen M., Chen K., Chen Z., Lu Z., Zhang Y., Zhang Y., Liu H. (2023). Stable Solid Electrolyte
Interface Achieved by Separator Surface Modification for High-Performance
Anode-free Potassium Metal Batteries. ACS Appl.
Energy Mater..

[540] Li Y., Zhang L., Liu S., Wang X., Xie D., Xia X., Gu C., Tu J. (2019). Original growth mechanism for ultra-stable
dendrite-free potassium metal electrode. Nano
Energy.

[541] Zhao Y., Liu B., Yi Y., Lian X., Wang M., Li S., Yang X., Sun J. (2022). An Anode-Free
Potassium-Metal Battery Enabled by a Directly Grown Graphene-Modulated
Aluminum Current Collector. Adv. Mater..

[542] Wang G., Zhu M., Chen G., Qu Z., Kohn B., Scheler U., Chu X., Fu Y., Schmidt O. G., Feng X. (2022). An anode-free Zn-graphite battery. Adv. Mater..

[543] Wang C., Wang D., Lv D., Peng H., Song X., Yang J., Qian Y. (2023). Interface Engineering
by Hydrophilic and Zincophilic Aluminum Hydroxide Fluoride for Anode-Free
Zinc Metal Batteries at Low Temperature. Adv.
Energy Mater..

[544] Liu P., Fan X., Ouyang B., Huang Y., Hao R., Gao S., Liu W., Liu K. (2022). A zn ion hybrid capacitor with enhanced
energy density for anode-free. J. Power Sources.

[545] Zhu Y., Cui Y., Alshareef H. N. (2021). An anode-free
Zn-MnO2 battery. Nano Lett..

[546] Li Y., Feng X., Lieu W. Y., Fu L., Zhang C., Ghosh T., Thakur A., Wyatt B. C., Anasori B., Liu W. (2023). MXene-based anode-free
magnesium metal battery. Adv. Funct. Mater..

[547] Hou S., Ji X., Gaskell K., Wang P.-f., Wang L., Xu J., Sun R., Borodin O., Wang C. (2021). Solvation sheath reorganization
enables divalent metal batteries with fast interfacial charge transfer
kinetics. Science.

[548] Li G., Duan X., Liu X., Zhan R., Wang X., Du J., Chen Z., Li Y., Cai Z., Shen Y. (2023). Locking active Li metal
through localized redistribution of fluoride
enabling stable Li-metal batteries. Adv. Mater..

[549] Meng Y., Wang M., Li K., Zhu Z., Liu Z., Jiang T., Zheng X., Zhang K., Wang W., Peng Q. (2023). Reversible, Dendrite-Free,
High-Capacity Aluminum Metal
Anode Enabled by Aluminophilic Interface Layer. Nano Lett..

[550] Zheng J., Bock D. C., Tang T., Zhao Q., Yin J., Tallman K. R., Wheeler G., Liu X., Deng Y., Jin S. (2021). Regulating electrodeposition morphology in high-capacity
aluminium and zinc battery anodes using interfacial metal-substrate
bonding. Nat. Energy.

[551] Chen W., Salvatierra R. V., Ren M., Chen J., Stanford M. G., Tour J. M. (2020). Laser-induced silicon
oxide for anode-free
lithium metal batteries. Adv. Mater..

[552] Kandula S., Kim E., Ahn C. W., Lee J., Yeom B., Lee S. W., Cho J., Lim H.-K., Lee Y., Son J. G. (2023). A resilient MXene/CNT nano-accordion framework for
anode-free sodium-metal batteries with exceptional cyclic stability. Energy Storage Mater..

[553] Wang C., Zheng Y., Chen Z. N., Zhang R., He W., Li K., Yan S., Cui J., Fang X., Yan J. (2023). Robust Anode-Free Sodium Metal Batteries Enabled by
Artificial Sodium Formate Interface. Adv. Energy
Mater..

[554] Wu S., Hwang J., Matsumoto K., Hagiwara R. (2023). The Rational Design
of Low-Barrier Fluorinated Aluminum Substrates for Anode-Free Sodium
Metal Battery. Adv. Energy Mater..

[555] Yang H., Lv H., Zhou E., Ji X., Chen C., Yu H., Sun Z., Zhang D., Jin H., Kong X. (2024). Na3P interphase reduces Na nucleation energy
enabling stable anode-less sodium metal batteries. J. Energy Chem..

[556] Kang S., Geng F., Li Z., Jiang Y., Shen M., Chen Q., Lou X., Hu B. (2024). Progressive
Self-Leveling Deposition Improves the Cyclability of Anode-less Sodium
Metal Batteries Revealed by In Situ EPR Imaging. ACS Energy Lett..

[557] Yao Y., Yang Y., Wang Z., Guo M., Liu P., Xing Z. (2024). Regulating sodium deposition by specific
surface area composite ratio
for anode-free Na metal batteries. J. Power
Sources.

[558] Jeong D. Y., Chang W. J., Jang S., Kim M., Kim Y., Kim B., Park W. I. (2024). Controlling dendrite
growth and side
reactions in anode-free Zn-ion aqueous batteries with PMMA: Zn coated
electrode. J. Energy Storage.

[559] Li N., Jia T., Liu Y., Ouyang Y., Lv Y., Zhong G., Wang Y., Sun B., Lu S., Huang S. (2023). Super-three-dimensional lithiophilic Cu-based current
collector for anode-free lithium metal battery. Mater. Today Energy.

[560] Xu K., Zheng X., Luo R., Sun J., Ma Y., Chen N., Wang M., Song L., Zhao Q., Chen W. (2023). A three-dimensional zincophilic nano-copper
host enables dendrite-free
and anode-free Zn batteries. Mater. Today Energy.

[561] You X., Feng Y., Ning D., Yao H., Wang M., Wang J., Chen B., Zhong G.-H., Yang C., Wu W. (2024). Phosphorized 3D Current Collector
for High-Energy Anode-Free Lithium
Metal Batteries. Nano Lett..

[562] Kwon H., Lee J.-H., Roh Y., Baek J., Shin D. J., Yoon J. K., Ha H. J., Kim J. Y., Kim H.-T. (2021). An electron-deficient carbon current
collector for
anode-free Li-metal batteries. Nat. commun..

[563] Cai Z., Tang F., Yang Y., Xu S., Xu C., Liu L., Rui X. (2023). A multifunctional super-sodiophilic
coating on aluminum
current collector for high-performance anode-free Na-metal batteries. Nano Energy.

[564] Zhang R., Li N. W., Cheng X. B., Yin Y. X., Zhang Q., Guo Y. G. (2017). Advanced micro/nanostructures for
lithium metal anodes. Adv. Sci..

[565] Zheng J., Kim M. S., Tu Z., Choudhury S., Tang T., Archer L. A. (2020). Regulating electrodeposition
morphology
of lithium: towards commercially relevant secondary Li metal batteries. Chem. Soc. Rev..

[566] Jin S., Jiang Y., Ji H., Yu Y. (2018). Advanced 3D current
collectors for lithium-based batteries. Adv.
Mater..

[567] Jia A., Chao J., Qin C., Guo X., Yuan G., Liu J., Pang H. (2024). Self-supported 3D current collector modified with in-situ
formed lithiophilic [Cu (NH3) 2] Cl for high-performance Li-metal
batteries. Chem. Eng. J..

[568] Zhang D., Dai A., Wu M., Shen K., Xiao T., Hou G., Lu J., Tang Y. (2020). Lithiophilic
3D porous CuZn current collector for stable lithium metal batteries. ACS Energy Lett..

[569] Zhang W., Zheng J., Ren Z., Wang J., Luo J., Wang Y., Tao X., Liu T. (2024). Anode-free sodium metal
pouch cell using cu3p nanowires in situ grown on current collector. Adv. Mater..

[570] Zhang J., Chen H., Wen M., Shen K., Chen Q., Hou G., Tang Y. (2022). Lithiophilic 3D copper-based
magnetic current collector for lithium-free anode to realize deep
lithium deposition. Adv. Funct. Mater..

[571] Lee K., Lee Y. J., Lee M. J., Han J., Lim J., Ryu K., Yoon H., Kim B. H., Kim B. J., Lee S. W. (2022). A 3D hierarchical
host with enhanced sodiophilicity enabling anode-free sodium-metal
batteries. Adv. Mater..

[572] Guo C., Guo Y., Tao R., Liao X., Du K., Zou H., Zhang W., Liang J., Wang D., Sun X.-G. (2022). Uniform
lithiophilic layers in 3D current collectors enable ultrastable
solid electrolyte interphase for high-performance lithium metal batteries. Nano Energy.

[573] Zhang S., Zeng J., Ma Y., Zhao Y., Qian Y., Suo L., Huang J., Wang X., Li W., Zhang B. (2023). Ultrathin hierarchical
porous Cu current collector
fabricated by anodic oxidation in complexing agent system for stable
anode-free Lithium metal batteries. Electrochim.
Acta.

[574] Wang T.-S., Liu Y., Lu Y.-X., Hu Y.-S., Fan L.-Z. (2018). Dendrite-free Na metal plating/stripping
onto 3D porous
Cu hosts. Energy Storage Mater..

[575] Chen J., Wang Y., Li S., Chen H., Qiao X., Zhao J., Ma Y., Alshareef H. N. (2023). Porous
metal current collectors for alkali metal batteries. Adv. Sci..

[576] Dai S., Lin Z., Hu H., Wang Y., Zeng L. (2024). 3D printing
for sodium batteries: From material design to integrated devices. Appl. Phys. Rev..

[577] Yang X., Hao J., Zhao Y., Chen J., Guo J., Chi C. (2025). Synthesis
3D copper foam current collector with lithiophilicity
Cu2O and LiF-rich interface layer for anode-free lithium metal batteries. J. Energy Storage.

[578] Li S., Zhu H., Liu Y., Wu Q., Cheng S., Xie J. (2023). Space-Confined Guest
Synthesis to Fabricate Sn-Monodispersed N-Doped
Mesoporous Host toward Anode-Free Na Batteries. Adv. Mater..

[579] Zhuang R., Zhang X., Qu C., Xu X., Yang J., Ye Q., Liu Z., Kaskel S., Xu F., Wang H. (2023). Fluorinated
porous frameworks enable robust anode-less
sodium metal batteries. Sci. Adv..

[580] Cui Z., Song J., Chen M., Wang W., Zhang W., Zhu Q. (2024). Non-completely selenized
Cu-OSe nanowires as potassium-philic host
for anode-free potassium metal batteries. Energy
Storage Mater..

[581] Deckenbach D., Schneider J. J. (2024). Toward a Metal Anode-Free Zinc-Air
Battery for Next-Generation Energy Storage. Small.

[582] Louli A., Eldesoky A., deGooyer J., Coon M., Aiken C., Simunovic Z., Metzger M., Dahn J. (2022). Different
positive electrodes for anode-free lithium metal cells. J. Electrochem. Soc..

[583] Xu T., Qin K., Tian C., Lin L., Li W., Suo L. (2025). Searching for the ideal Li1+ xTMO2 cathode for anode-free
Li metal
batteries. Energy Storage Mater..

[584] Lin L., Qin K., Li M., Hu Y.-s., Li H., Huang X., Chen L., Suo L. (2022). Spinel-related Li2Ni0.
5Mn1. 5O4 cathode for 5-V anode-free lithium metal batteries. Energy Storage Mater..

[585] Lin L., Qin K., Zhang Q., Gu L., Suo L., Hu Y. s., Li H., Huang X., Chen L. (2021). Li-Rich Li2
[Ni0. 8Co0. 1Mn0. 1] O2 for Anode-Free Lithium Metal Batteries. Angew. Chem., Int. Ed..

[586] Jote B. A., Shitaw K. N., Weret M. A., Yang S.-C., Huang C.-J., Wang C.-H., Weng Y.-T., Wu S.-H., Su W.-N., Hwang B. J. (2022). Lithium nitrate
as a surplus lithium
source for anode-free cell with Ni-rich (NMC811) cathode. J. Power Sources.

[587] Qiao Y., Yang H., Chang Z., Deng H., Li X., Zhou H. (2021). A high-energy-density and long-life initial-anode-free
lithium battery enabled by a Li2O sacrificial agent. Nat. Energy.

[588] Park S. W., Choi H. J., Yoo Y., Lim H. D., Park J. W., Lee Y. J., Ha Y. C., Lee S. M., Kim B. G. (2022). Stable cycling of all-solid-state batteries with sacrificial
cathode and lithium-free indium layer. Adv.
Funct. Mater..

[589] Liu Y., Meng X., Shi Y., Qiu J., Wang Z. (2023). Long-Life
Quasi-Solid-State Anode-Free Batteries Enabled by Li Compensation
Coupled Interface Engineering. Adv. Mater..

[590] Xu T., Qin K., Tian C., Lin L., Li W., Suo L. (2025). Searching for the Ideal Li1+ xTMO2
Cathode for Anode-free Li Metal
Batteries. Energy Storage Mater..

[591] Kwon Y., Svirinovsky-Arbeli A., Hestenes J. C., Botero P. J. B., Corpus K. R. M., Lepucki P., Pecher O., Marbella L. E. (2024). Elucidating
the role of cathode identity: Voltage-dependent reversibility of anode-free
batteries. Chem..

[592] Li L., Hu Z., Lu Y., Wang C., Zhang Q., Zhao S., Peng J., Zhang K., Chou S. L., Chen J. (2021). A Low-strain potassium-rich
prussian blue analogue cathode for high
power potassium-ion batteries. Angew. Chem..

[593] Rosenbach C., Walther F., Ruhl J., Hartmann M., Hendriks T. A., Ohno S., Janek J., Zeier W. G. (2023). Visualizing
the Chemical Incompatibility of Halide and Sulfide-Based Electrolytes
in Solid-State Batteries. Adv. Energy Mater..

[594] Kotobuki M., Yan B., Lu L. (2023). Recent progress
on
cathode materials for rechargeable magnesium batteries. Energy Storage Mater..

[595] Ye X., Xiao X., Wu Z., Zhan Y., Wu X., Liu S. (2024). Recent advances in rechargeable aqueous magnesium-ion
batteries. J. Mater. Chem. A.

[596] Wu W., Wang A., Zhan Q., Hu Z., Tang W., Zhang L., Luo J. (2023). A molecularly engineered
cathode
lithium compensation agent for high energy density batteries. Small.

[597] Sul H., Bhargav A., Manthiram A. (2022). Lithium Trithiocarbonate as a Dual-Function
Electrode Material for High-Performance Lithium-Sulfur Batteries. Adv. Energy Mater..

[598] Lu C., Zhao F., Tao B., Wang Z., Wang Y., Sheng J., Tang G., Wang Y., Guo X., Li J. (2024). Anode-Free Aqueous Aluminum Ion Batteries. Small.

[599] Arifiadi A., Demelash F., Abke N. M., Brake T., Vahnstiege M., Alsheimer L., Wiemers-Meyer S., Winter M., Kasnatscheew J. (2025). Assessment
of “Inverse”
Cross-Talk (Anode to Cathode) in High-Voltage Li/Mn-Rich Layered Oxide||
Li Cells. Adv. Funct. Mater..

[600] Betz J., Brinkmann J. P., Nölle R., Lürenbaum C., Kolek M., Stan M. C., Winter M., Placke T. (2019). Cross talk between transition metal
cathode and Li
metal anode: unraveling its influence on the deposition/dissolution
behavior and morphology of lithium. Adv. energy
mater..

[601] Zhao T., Peng D., Zhang A., Han J., Liu T., You Y. (2025). Unrevealing the Capacity Fading from Anode-to-Cathode
Cross-Talk in Prussian White Cathode. Adv. Funct.
Mater..

[602] Jeevanantham B., Shobana M., Nikodimos Y., Vallal S. A., Su W.-N., Hwang B. J. (2025). Alleviating parasitic
reaction of nickel-rich layered active materials at high voltage cycling
by rare-earth metal oxide coating: A comparison of cyclic performance. J. Power Sources.

[603] Ahmed S. A., Agnihotri T., Ranjan A., Chang C.-Y., Chang C.-C., Hasan R., Nikodimos Y., Hagos T. M., Wu S.-H., Su W.-N., Hwang B. J. (2025). Boosting
stability in Ni-rich cathodes: a synergistic approach to surface and
bulk modifications for advanced lithium-ion batteries. J. Mater. Chem. A.

[604] Liu Z., Lu Z., Guo S., Yang Q.-H., Zhou H. (2023). Toward high
performance anodes for sodium-ion batteries: from hard carbons to
anode-free systems. ACS Cent. Sci..

[605] Li S., Zhu H., Gu C., Ma F., Zhong W., Liu M., Zhang H., Zeng Z., Cheng S., Xie J. (2023). Customized
electrolyte and host structures enabling high-energy-density anode-free
potassium-metal batteries. ACS Energy Lett..

[606] Rana A., Roy K., Heil J. N., Nguyen J. H., Renault C., Tackett B. M., Dick J. E. (2024). Realizing the Kinetic
Origin of Hydrogen Evolution for Aqueous Zinc Metal Batteries. Adv. Energy Mater..

[607] Yu X., Chen M., Li Z., Tan X., Zhang H., Wang J., Tang Y., Xu J., Yin W., Yang Y. (2024). Unlocking dynamic solvation chemistry and hydrogen
evolution mechanism in aqueous zinc batteries. J. Am. Chem. Soc..

[608] Nanda S., Asl H. Y., Bhargav A., Manthiram A. (2022). Thiometallate-mediated
polysulfide chemistry and lithium stabilization for stable anode-free
lithium-sulfur batteries. Cell Rep. Phys. Sci..

[609] Yao W., Liao K., Lai T., Sul H., Manthiram A. (2024). Rechargeable
metal-sulfur batteries: key materials to mechanisms. Chem. Rev..

[610] Nanda S., Bhargav A., Manthiram A. (2020). Anode-free,
lean-electrolyte lithium-sulfur batteries enabled by tellurium-stabilized
lithium deposition. Joule.

[611] Geng C., Qu W., Han Z., Wang L., Lv W., Yang Q. H. (2023). Superhigh coulombic
efficiency lithium-sulfur batteries
enabled by in situ coating lithium sulfide with polymerizable electrolyte
additive. Adv. Energy Mater..

[612] Chen H., Dai C., Xiao F., Yang Q., Cai S., Xu M., Fan H. J., Bao S. J. (2022). Reunderstanding
the reaction mechanism of aqueous Zn-Mn batteries with sulfate electrolytes:
role of the zinc sulfate hydroxide. Adv. Mater..

[613] Zhao S., Han B., Zhang D., Huang Q., Xiao L., Chen L., Ivey D. G., Deng Y., Wei W. (2018). Unravelling the reaction chemistry
and degradation mechanism in aqueous
Zn/MnO 2 rechargeable batteries. J. Mater. Chem.
A.

[614] Razaz G., Weißensteiner I., Ortegren J., Trink B., Pogatscher S., Arshadi Rastabi S. (2024). Impact of Surface Microstructure
and Properties of Aluminum Electrodes on the Plating/Stripping Behavior
of Aluminum-Based Batteries Using Imidazolium-Based Electrolyte. ACS Appl. Mater. Interfaces.

[615] Elia G. A., Kravchyk K. V., Kovalenko M. V., Chacón J., Holland A., Wills R. G. (2021). An overview and
prospective on Al and Al-ion battery technologies. J. Power Sources.

[616] Tamilarasan E. B., Huang Y.-C., Agnihotri T., Yeh T.-I., Nikodimos Y., Moges E. A., Ahmed S. A., Hasan R., Saravanan A. V., Taklu B. W. (2025). Probing
Li2S Activation Mechanism in Lithium-Sulfur Batteries via Multimodal
Operando Techniques. ACS Energy Lett..

[617] Fenta F. W., Olbasa B. W., Tsai M.-C., Temesgen N. T., Huang W.-H., Tekaligne T. M., Nikodimos Y., Wu S.-h., Su W.-N., Dai H. (2022). Structural
engineering of α-MnO2 cathode by Ag+ incorporation for high
capacity aqueous zinc-ion batteries. J. Power
Sources.

[618] Berhe G. B., Su W.-N., Huang C.-J., Hagos T. M., Hagos T. T., Bezabh H. K., Weret M. A., Abrha L. H., Yang Y.-W., Hwang B.-J. (2019). A new class of lithium-ion
battery
using sulfurized carbon anode from polyacrylonitrile and lithium manganese
oxide cathode. J. Power Sources.

[619] Hasan M. W., Abrha L. H., Hossain M. F., Oli H., Lama B., Bandlamudi S. R., Terkildsen N., Shchepin R., Pupek K. Z., Paudel T. R. (2025). A Synergistic
Strategy for the Development of Advanced, Scalable Lithium-Sulfur
Batteries. ACS Appl. Mater. Interfaces.

[620] Berhe G. B., Su W.-N., Abrha L. H., Bezabh H. K., Hagos T. M., Hagos T. T., Huang C.-J., Sahalie N. A., Jote B. A., Thirumalraj B. (2020). Garnet-PVDF composite
film modified lithium manganese oxide cathode and sulfurized carbon
anode from polyacrylonitrile for lithium-ion batteries. J. Mater. Chem. A.

[621] Li Z., He T., Gong Y., Jiang D. (2020). Covalent organic frameworks:
pore design and interface engineering. Acc.
Chem. Res..

[622] Sun Q., Fu C.-W., Aguila B., Perman J., Wang S., Huang H.-Y., Xiao F.-S., Ma S. (2018). Pore environment
control
and enhanced performance of enzymes infiltrated in covalent organic
frameworks. J. Am. Chem. Soc..

[623] Xiong D., Li X., Bai Z., Lu S. (2018). Recent advances
in layered Ti3C2Tx MXene for electrochemical energy storage. Small.

[624] Li K., Liang M., Wang H., Wang X., Huang Y., Coelho J., Pinilla S., Zhang Y., Qi F., Nicolosi V. (2020). 3D MXene architectures
for efficient energy storage
and conversion. Adv. Funct. Mater..

[625] Sul H., Manthiram A. (2024). Bi-Metallic
Phosphide Electrocatalyst-Integrated Li2S
Cathode for High-Performance Anode-Free Li-S Batteries. Adv. Funct. Mater..

[626] Jin B., Lai T., Manthiram A. (2023). High-Mass-Loading Anode-Free Lithium-Sulfur
Batteries Enabled by a Binary Binder with Fast Lithium-Ion Transport. ACS Energy Lett..

[627] Sul H., He J., Manthiram A. (2023). Tellurium
Nanowires for Lithium-Metal
Anode Stabilization in High-Performance Anode-Free Li-S Batteries. Small Sci..

[628] Zhao Y., Huang L., Zhao D., Yang Lee J. (2023). Fast polysulfide
conversion catalysis and reversible anode operation by a single cathode
modifier in Li-metal anode-free lithium-sulfur batteries. Angew. Chem., Int. Ed..

[629] Meng X., Liu Y., Yu L., Qiu J., Wang Z. (2023). Air-Stable Li2S Cathode for Quasi-Solid-State Anode-Free
Batteries
with High Volumetric Energy. Adv. Funct. Mater..

[630] Sheng L., Xie X., Sun Z., Zhao M., Gao B., Pan J., Bai Y., Song S., Liu G., Wang T. (2021). Role of
separator surface polarity in boosting the
lithium-ion transport property for a lithium-based battery. ACS Appl. Energy Mater..

[631] Ren Y., Liang X., Dou H., Ye C., Guo Z., Wang J., Pan Y., Wu H., Guiver M. D., Jiang Z. (2020). Membrane-based olefin/paraffin separations. Adv. Sci..

[632] Tan X., Rodrigue D. (2019). A review on porous polymeric membrane preparation.
Part II: Production techniques with polyethylene, polydimethylsiloxane,
polypropylene, polyimide, and polytetrafluoroethylene. Polymers.

[633] Lagadec M. F., Zahn R., Wood V. (2019). Characterization and
performance evaluation of lithium-ion battery separators. Nat. Energy.

[634] Parekh M. H., Oka S., Lutkenhaus J., Pol V. G. (2022). Critical-point-dried, porous, and safer aramid nanofiber
separator for high-performance durable lithium-ion batteries. ACS Appl. Mater. Interfaces.

[635] Zhang H., Ma X., Chen R., Wang X., Ma H., Chai Y., Cao T., Rao W., Chen J., Ji J. (2022). Selective ion transport
in assembled graphene oxide-modified
separator and the novel intra-series architecture for improved aqueous
batteries. Chem. Eng. J..

[636] Arora P., Zhang Z. (2004). Battery separators. Chem. Rev..

[637] Wang L., Wang Z., Bai Y., Yuan D., Chen J., Dou S. X., Liu H. K., Wu C. (2025). Separator
Engineering for Sodium Metal Batteries: Challenges, Rational Design
and Recent Modification Strategies. Adv. Mater..

[638] Zhao Q., Hao Z., Tang J., Xu X., Liu J., Jin Y., Zhang Q., Wang H. (2021). Cation-Selective
Separators
for Addressing the Lithium-Sulfur Battery Challenges. ChemSusChem.

[639] Liang Y., Ma D., Zhao N., Wang Y., Yang M., Ruan J., Yang G., Mi H., He C., Zhang P. (2022). Novel concept of separator design: efficient ions transport
modulator enabled by dual-interface engineering toward ultra-stable
Zn metal anodes. Adv. Funct. Mater..

[640] Zhang W., Tu Z., Qian J., Choudhury S., Archer L. A., Lu Y. (2018). Design principles of
functional polymer
separators for high-energy, metal-based batteries. Small.

[641] Zong Y., He H., Wang Y., Wu M., Ren X., Bai Z., Wang N., Ning X., Dou S. X. (2023). Functionalized
separator strategies toward advanced aqueous zinc-ion batteries. Adv. Energy Mater..

[642] Gu W., Wu K., Huang J., Yang X., Huang X., Dong Z., Shen S., Bai Y., Liu H. K., Dou S. X. (2025). The Role and Mechanism of Separators in Aqueous Zinc
Metal Batteries: a Critical Review. Adv. Energy
Mater..

[643] Thang A. Q., Tso S., Jia B. E., Tan X. Y., Dong J., Zhang M., Ng M. F., Yao G., Wong S. Y., Liu Z. (2024). Functionalizing separator
by coating a lithiophilic metal for dendrite-free anode-free lithium
metal batteries. Chem. Asian J..

[644] Hao H., Hutter T., Boyce B. L., Watt J., Liu P., Mitlin D. (2022). Review of multifunctional
separators: Stabilizing the
cathode and the anode for alkali (Li, Na, and K) metal-sulfur and
selenium batteries. Chem. Rev..

[645] Huang X., He R., Li M., Chee M. O. L., Dong P., Lu J. (2020). Functionalized
separator for next-generation
batteries. Mater. Today.

[646] Ji Y., Yang C., Han J., He W. (2024). Functional Separators
for Modulating Li-Ion Flux Toward Uniform Li Deposition: A Review. Adv. Energy Mater..

[647] Bai P., Guo J., Wang M., Kushima A., Su L., Li J., Brushett F. R., Bazant M. Z. (2018). Interactions between lithium growths
and nanoporous ceramic separators. Joule.

[648] Hao Z., Zhao Q., Tang J., Zhang Q., Liu J., Jin Y., Wang H. (2021). Functional separators towards the suppression of lithium
dendrites for rechargeable high-energy batteries. Mater. Horiz..

[649] Wang H., Zhao Y., Huang J., Wang L., Li C., Chen Y. (2025). Ultra-Stable Anode-Free Na Metal Batteries Enabled
by Al2O3-Functionalized Separators. Batteries.

[650] Muche Z. B., Nikodimos Y., Tekaligne T. M., Merso S. K., Agnihotri T., Serbessa G. G., Wu S.-H., Su W.-N., Hwang B. J. (2023). Thermally
stable 3D cross-linked
fluorinated polyimide/PVDF-HFP hybrid separator for lithium battery
applications. Chem. Eng. J..

[651] Ni Q., Kim B., Wu C., Kang K. (2022). Non-electrode
components
for rechargeable aqueous zinc batteries: Electrolytes, solid-electrolyte-interphase,
current collectors, binders, and separators. Adv. Mater..

[652] Li G. (2021). Regulating mass transport
behavior for high-performance lithium metal
batteries and fast-charging lithium-ion batteries. Adv. Energy Mater..

[653] Lei H., Jiao S., Tu J., Song W.-L., Zhang X., Wang M., Li S., Chen H., Fang D. (2020). Modified separators
for rechargeable high-capacity selenium-aluminium batteries. Chem. Eng. J..

[654] Ji Y., Liu-Théato X., Xiu Y., Indris S., Njel C., Maibach J., Ehrenberg H., Fichtner M., Zhao-Karger Z. (2021). Polyoxometalate modified separator
for performance enhancement of magnesium-sulfur batteries. Adv. Funct. Mater..

[655] Lin G., Meng T., Peng Y., Li P., Hu X. (2025). Janus-Architectured
Lithium Replenishment Separators Boosting Longevity of Anode-Free
Lithium Metal Batteries. Small Methods.

[656] Zhang F., Lu Q., Li J., Zhang Q., Yu H., Wang Y., Li J., Ren H., Liang H., Shen F. (2025). Dual-Strategy Design
Based on Polymer-Matrix Composite
Cathode and Coated Separator for High-Performance Lithium-Iron Disulfide
Batteries. Materials.

[657] Lee F., Tsai M.-C., Lin M.-H., Ni’mah Y. L., Hy S., Kuo C.-Y., Cheng J.-H., Rick J., Su W.-N., Hwang B.-J. (2017). Capacity retention
of lithium sulfur batteries enhanced
with nano-sized TiO 2-embedded polyethylene oxide. J. Mater. Chem. A.

[658] Dai L., Yi Z., Xie L., Su F., Guo X., Wang Z., Cheng J., Chen C. (2024). Hierarchically
Porous
Carbon Microspheres Coated with MnO2 Nanosheets as the Sulfur Host
for High-Loading Lithium-Sulfur Batteries. Molecules.

[659] Bosubabu D., Sivaraj J., Gurunathan P., Ramesha K. (2020). Hollow Co3O4 microspheres grafted with nitrogen-doped
carbon nanotubes as efficient sulfur host for high performing lithium-sulfur
batteries. Energy Fuels.

[660] Wang J., Li J. (2022). C@ MoS2 modified separator
as efficient
trapper and catalysis for promoting polysulfide conversion in Li-S
battery. J. Colloid Interface Sci..

[661] Huang Z., Liang Y., Wu Z., Kong Y., Bai M., Li M., Hong B., Huang T., Huang S., Chen H. (2025). Multifunctional
Ultrathin Ti3C2Tx MXene@ CuCo2O4/PE
Separator for Ultra-High-Energy-Density and Large-Capacity Lithium-Sulfur
Pouch Cells. Adv. Mater..

[662] Huang J.-Q., Zhang Q., Wei F. (2015). Multi-functional
separator/interlayer
system for high-stable lithium-sulfur batteries: Progress and prospects. Energy Storage Mater..

[663] Zhao C., Gao Y., Chen Y., Yin X., Liu H., Li W., Hu X., Wang J., Xi K., Ding S. (2025). Enhancing Li+ Kinetics Via Selective Repulsion-Adsorption
and Intermolecular Ion-Conduction Layers for High-Energy-Density Anode-Free
Lithium-Metal Batteries. Adv. Mater..

[664] Lim D.-A., Seok J.-H., Lim S.-J., Kim D.-W. (2025). Multi-scavenging
functional separator ensuring the cycling stability of high energy
density lithium-ion batteries. J. Power Sources.

[665] Davino S., Mustarelli P., Callegari D., Quartarone E. (2025). Multifunctional scavengers in an
MOF-Al 2 O 3-based
Janus separator for high-voltage lithium batteries. EES Batteries.

[666] Zhou L., Tian S., Du X., Liu T., Zhang H., Zhang J., Hu S., Chen Z., Zhang J., Cui G. (2023). Suppressing hydrogen evolution in
aqueous lithium-ion batteries with double-site hydrogen bonding. ACS Energy Lett..

[667] Du H., Yi Z., Li H., Lv W., Hu N., Zhang X., Chen W., Wei Z., Shen F., He H. (2024). Separator design strategies to advance
rechargeable aqueous zinc
ion batteries. Chem. Eur. J..

[668] Nian Q., Yang X., Hong H., Chen P., Zhao Y., Lv H., Zhi C. (2025). Advancements
in Separator
Materials for Aqueous Zinc Batteries. Nanoscale
Horizons.

[669] Kumar J., Kichambare P., Rai A. K., Bhattacharya R., Rodrigues S., Subramanyam G. (2016). A high performance ceramic-polymer
separator for lithium batteries. J. Power Sources.

[670] Kim K. M., Latifatu M., Lee Y.-G., Ko J. M., Kim J. H., Cho W. I. (2014). Effect of ceramic
filler-containing
polymer hydrogel electrolytes coated on the polyolefin separator on
the electrochemical properties of activated carbon supercapacitor. J. Electroceram..

[671] Muche Z. B., Nikodimos Y., Chang C.-Y., Merso S. K., Tekaligne T. M., Shitaw K. N., Serbessa G. G., Hagos T. M., Taklu B. W., Agnihotri T. (2025). Integrating electrodes
and PVDF-HFP/Kevlar nanofiber separator for multi-functions using
electrospinning. Chem. Eng. J..

[672] Wei Z., Ren Y., Sokolowski J., Zhu X., Wu G. (2020). Mechanistic
understanding of the role separators playing in advanced lithium-sulfur
batteries. InfoMat.

[673] Cavers H., Molaiyan P., Abdollahifar M., Lassi U., Kwade A. (2022). Perspectives on improving the safety
and sustainability of high voltage lithium-ion batteries through the
electrolyte and separator region. Adv. Energy
Mater..

[674] Yao N., Liu F., Shao A., Xue R., Jia Q., Liu Y., Wang H., Wang X., Zhang Y., Zhang M. (2024). Upcycling the Spent Graphite Anode Into the Prelithiation Catalyst:
A Separator Strategy Toward Anode-Free Cell Prototyping. Adv. Mater..

[675] Yuan H., Wen K., Guan S., Liang Y., Wu Y.-H., Liu S., Yu D., Li L., Nan C.-W. (2024). In-plane isotropic separator-induced highly efficient
sodium plating for unlocking the fast-charging capability of anode-free
sodium battery at practical conditions. J. Materiomics.

[676] Hu Z., Liu L., Wang X., Zheng Q., Lu H., Tang Z., Han C., Li W. (2025). Enhancing low-temperature
durability and sodium-ion transport of anode-free sodium metal batteries
through utilization of a solvent adsorption separator. Energy Environ. Sci..

[677] Redda H. G., Nikodimos Y., Su W.-N., Chen R.-S., Jiang S.-K., Abrha L. H., Hagos T. M., Bezabh H. K., Weldeyohannes H. H., Hwang B. J. (2021). Enhancing the electrochemical performance
of a flexible solid-state supercapacitor using a gel polymer electrolyte. Mater. Today Commun..

[678] Nikodimos Y., Su W. N., Hwang B. J. (2023). Halide solid-state
electrolytes: stability and application for high voltage all-solid-state
Li batteries. Adv. Energy Mater..

[679] Nikodimos Y., Huang C.-J., Taklu B. W., Su W.-N., Hwang B. J. (2022). Chemical Stability of Sulfide Solid-state
Electrolytes:
Stability Toward Humid Air and Compatibility with Solvents and Binders. Energy Environ. Sci..

[680] Nikodimos Y., Tsai M.-C., Abrha L. H., Weldeyohannis H. H., Chiu S.-F., Bezabh H. K., Shitaw K. N., Fenta F. W., Wu S.-H., Su W.-N., Yang C.-C., Hwang B. J. (2020). Al-Sc Dual
Doped LiGe2 (PO4) 3-a NASICON-Type Solid Electrolyte with Improved
Ionic Conductivity. J. mater. chem. A.

[681] Nikodimos Y., Taklu B. W., Chang C.-Y., Shitaw K. N., Hagos T. M., Muche Z. B., Chaudhary S. K., Serbessa G. G., Yeh T.-I., Merso S. K. (2025). Unveiling
the Chemical Stability and Solvent Compatibility of Halide Solid-State
Electrolytes: Insights from Isothermal Calorimetric Titration and
Synchrotron Spectroscopy. Chem. Mater..

[682] Temesgen N. T., Bezabh H. K., Weret M. A., Shitaw K. N., Nikodimos Y., Taklu B. W., Lakshmanan K., Yang S.-C., Jiang S.-K., Huang C.-J., Wu S.-H., Su W.-N., Hwang B. J. (2023). Solvent-free design of argyrodite
sulfide composite solid electrolyte with superb interface and moisture
stability in anode-free lithium metal batteries. J. Power Sources.

[683] Zegeye T. A., Su W.-N., Fenta F. W., Zeleke T. S., Jiang S.-K., Hwang B. J. (2020). Ultrathin Li6. 75La3Zr1. 75Ta0. 25O12-Based
Composite Solid Electrolytes Laminated on Anode and Cathode Surfaces
for Anode-free Lithium Metal Batteries. ACS
Appl. Energy Mater..

[684] Zhao Q., Stalin S., Zhao C.-Z., Archer L. A. (2020). Designing
solid-state electrolytes for safe, energy-dense batteries. Nat. Rev. Mater..

[685] Kong L., Li Y., Feng W. (2021). Strategies
to solve
lithium battery thermal runaway: from mechanism to modification. Electrochem. Energy Rev..

[686] Taklu B. W., Su W.-N., Nikodimos Y., Lakshmanan K., Temesgen N. T., Lin P.-X., Jiang S.-K., Huang C.-J., Wang D.-Y., Sheu H.-S., Wu S.-H., Hwang B. J. (2021). Dual CuCl doped argyrodite superconductor to boost
the interfacial compatibility and air stability for all solid-state
lithium metal batteries. Nano Energy.

[687] Shitaw K. N., Yang S. C., Jiang S. K., Huang C. J., Sahalie N. A., Nikodimos Y., Weldeyohannes H. H., Wang C. H., Wu S. H., Su W. N., Hwang B. J. (2021). Decoupling
Interfacial Reactions at Anode and Cathode by Combining Online Electrochemical
Mass Spectroscopy with Anode-Free Li-Metal Battery. Adv. Funct. Mater..

[688] Taklu B. W., Chia P. H., Liu C. L., Nikodimos Y., Serbessa G. G., Kitaba L. T., Shitaw K. N., Yeh T.-I., Lin L.-Y., Chang J.-H. (2025). Reaction-induced heat
evolution and interface stabilization from bifunctional chlorine-rich
anode for sulfide solid-state batteries. Energy
Storage Materials.

[689] Kazyak E., Wang M. J., Lee K., Yadavalli S., Sanchez A. J., Thouless M., Sakamoto J., Dasgupta N. P. (2022). Understanding
the electro-chemo-mechanics of Li plating in anode-free solid-state
batteries with operando 3D microscopy. Matter.

[690] Lee Y.-G., Fujiki S., Jung C., Suzuki N., Yashiro N., Omoda R., Ko D.-S., Shiratsuchi T., Sugimoto T., Ryu S. (2020). High-energy long-cycling
all-solid-state lithium metal batteries enabled by silver-carbon composite
anodes. Nat. Energy.

[691] Ye L., Lu Y., Wang Y., Li J., Li X. (2024). Fast cycling
of lithium metal in solid-state batteries by constriction-susceptible
anode materials. Nat. Mater..

[692] Cui C., Yang H., Zeng C., Gui S., Liang J., Xiao P., Wang S., Huang G., Hu M., Zhai T. (2022). Unlocking the in situ Li plating dynamics and
evolution
mediated by diverse metallic substrates in all-solid-state batteries. Sci. Adv..

[693] Ko D.-S., Kim S., Lee S., Yoon G., Kim D., Shin C., Kim D., Lee J., Sul S., Yun D.-J. (2025). Mechanism of stable
lithium plating and stripping in
a metal-interlayer-inserted anode-less solid-state lithium metal battery. Nat. Commun..

[694] Vishnugopi B. S., Naik K. G., Kawakami H., Ikeda N., Mizuno Y., Iwamura R., Kotaka T., Aotani K., Tabuchi Y., Mukherjee P. P. (2023). Asymmetric
contact loss dynamics
during plating and stripping in solid-state batteries. Adv. Energy Mater..

[695] Shen Z. Z., Zhang X. S., Wan J., Liu G. X., Tian J. X., Liu B., Guo Y. G., Wen R. (2024). Nanoscale
Visualization of Lithium Plating/Stripping Tuned by On-site Formed
Solid Electrolyte Interphase in All-Solid-State Lithium-Metal Batteries. Angew. Chem..

[696] Yu H., Wang L., Zhang Z., Li Y., Yang S., He X. (2024). Insight Understanding of External
Pressure on Lithium Plating in
Commercial Lithium-Ion Batteries. Adv. Funct.
Mater..

[697] Nikodimos Y., Jiang S.-K., Huang S.-J., Taklu B. W., Huang W.-H., Desta G. B., Tekaligne T. M., Muche Z. B., Lakshmanan K., Chang C.-Y. (2024). Moisture
robustness of Li6PS5Cl argyrodite sulfide solid electrolyte improved
by nano-level treatment with lewis acid additives. ACS Energy Lett..

[698] Abrha L. H., Hagos T. T., Nikodimos Y., Bezabh H. K., Berhe G. B., Hagos T. M., Huang C.-J., Addis Tegegne W., Jiang S.-K., Weldeyohannes H. H., Wu S.-H., Su W.-N., Hwang B. J. (2020). Dual-Doped Cubic
Garnet Solid Electrolyte with Superior Air Stability. ACS Appl. Mater. Interfaces.

[699] Nikodimos Y., Su W.-N., Bezabh H., Tsai M.-C., Yang C.-C., Hwang B. (2022). Effect of selected
dopants on conductivity
and moisture stability of Li3PS4 sulfide solid electrolyte: a first-principles
study. Mater. Today Chem..

[700] Hwang, B.-J. ; Yang, S.-C. ; Wu, S.-H. ; Su, W.-N. ; Asgedom, Y. N. , Additive Containing Sulfide-Based Solid Electrolyte. U.S. Patents: 2023; p US20230343995A1.

[701] Jiang S.-K., Yang S.-C., Nikodimos Y., Huang S.-J., Lin K.-Y., Kuo Y.-H., Tsai B.-Y., Li J.-N., Lin S. D., Jiang J.-C., Wu S.-H., Su W.-N., Hwang B. J. (2023). Lewis Acid Probe for Basicity of
Sulfide Electrolytes Investigated by 11B Solid-State NMR. JACS Au.

[702] Asgedom, Y. N. ; Su, W.-N. ; Hwang, B. J. In Enhancing Moisture Tolerance of Sulfide Solid Electrolytes through Nano-Level Treatment Utilizing Lewis Acid Additives; Electrochemical Society Meeting Abstracts 245; The Electrochemical Society, Inc., 2024; pp 115–115.

[703] Taklu B. W., Nikodimos Y., Bezabh H. K., Lakshmanan K., Hagos T. M., Nigatu T. A., Merso S. K., Sung H.-Y., Yang S.-C., Wu S.-H. (2023). Air-stable iodized-oxychloride
argyrodite sulfide and anionic swap on the practical potential window
for all-solid-state lithium-metal batteries. Nano Energy.

[704] Nikodimos Y., Abrha L. H., Weldeyohannes H. H., Shitaw K. N., Temesgen N. T., Olbasa B. W., Huang C.-J., Jiang S.-K., Wang C.-H., Sheu H.-S., Wu S.-H., Su W.-N., Yang C.-C., Hwang B. J. (2020). A new high-Li+-conductivity
Mg-doped Li1.5Al0.5Ge1.5­(PO4)­3 solid electrolyte with enhanced electrochemical
performance for solid-state lithium metal batteries. J. Mater. Chem. A.

[705] Nikodimos Y., Su W.-N., Taklu B. W., Merso S. K., Hagos T. M., Huang C.-J., Redda H. G., Wang C.-H., Wu S.-H., Yang C.-C., Hwang B. J. (2022). Resolving anodic
and cathodic interface-incompatibility in solid-state lithium metal
battery via interface infiltration of designed liquid electrolytes. J. Power Sources.

[706] Zhu Y., He X., Mo Y. (2015). Origin of outstanding stability in
the lithium solid electrolyte materials: insights from thermodynamic
analyses based on first-principles calculations. ACS Appl. Mater. Interfaces.

[707] Ding J.-F., Xu R., Yan C., Li B.-Q., Yuan H., Huang J.-Q. (2021). A review on the
failure and regulation
of solid electrolyte interphase in lithium batteries. J. Energy Chem..

[708] Cheng X.-B., Yan C., Zhang X.-Q., Liu H., Zhang Q. (2018). Electronic and ionic channels in working interfaces of lithium metal
anodes. ACS Energy Lett..

[709] Mo Y., Ong S. P., Ceder G. (2012). First principles
study of the Li10GeP2S12
lithium super ionic conductor material. Chem.
Mater..

[710] Wenzel S., Randau S., Leichtweiß T., Weber D. A., Sann J., Zeier W. G., Janek J. r. (2016). Direct
observation of the interfacial instability of the fast ionic conductor
Li10GeP2S12 at the lithium metal anode. Chem.
Mater..

[711] Hartmann P., Leichtweiss T., Busche M. R., Schneider M., Reich M., Sann J., Adelhelm P., Janek J. (2013). Degradation
of NASICON-type materials in contact with lithium metal: formation
of mixed conducting interphases (MCI) on solid electrolytes. J. Phys. Chem. C.

[712] Wang C., Zhang H., Li J., Chai J., Dong S., Cui G. (2018). The interfacial evolution
between
polycarbonate-based polymer electrolyte and Li-metal anode. J. Power Sources.

[713] Shen F., Wang K., Yin Y., Shi L., Zeng D., Han X. (2020). PAN/PI functional double-layer coating
for dendrite-free lithium metal anodes. J. Mater.
Chem. A.

[714] Marchiori C. F., Carvalho R. P., Ebadi M., Brandell D., Araujo C. M. (2020). Understanding
the electrochemical stability window
of polymer electrolytes in solid-state batteries from atomic-scale
modeling: the role of Li-ion salts. Chem. Mater..

[715] Su S., Ma J., Zhao L., Lin K., Li Q., Lv S., Kang F., He Y. B. (2021). Progress and perspective of the cathode/electrolyte
interface construction in all-solid-state lithium batteries. Carbon Energy.

[716] Wang M. J., Carmona E., Gupta A., Albertus P., Sakamoto J. (2020). Enabling “lithium-free”
manufacturing
of pure lithium metal solid-state batteries through in situ plating. Nat. Commun..

[717] Davis A. L., Garcia-Mendez R., Wood K. N., Kazyak E., Chen K.-H., Teeter G., Sakamoto J., Dasgupta N. P. (2020). Electro-chemo-mechanical
evolution of sulfide solid electrolyte/Li metal interfaces: operando
analysis and ALD interlayer effects. J. Mater.
Chem. A.

[718] Krauskopf T., Dippel R., Hartmann H., Peppler K., Mogwitz B., Richter F. H., Zeier W. G., Janek J. (2019). Lithium-metal
growth kinetics on LLZO garnet-type solid electrolytes. Joule.

[719] Fenta A. T., Nikodimos Y., Merso S. K., Meniber A., Weret M. A., Shitaw K. N., Taklu B. W., Valencia F., Yeh T.-I., Hsieh C. L. (2025). Boosting anode interfacial
stability in All-Solid-State lithium hybrid batteries with MCMB-Modified
stainless steel current collector. Chem. Eng.
J..

[720] Ning Z., Jolly D. S., Li G., De Meyere R., Pu S. D., Chen Y., Kasemchainan J., Ihli J., Gong C., Liu B. (2021). Visualizing
plating-induced cracking in lithium-anode solid-electrolyte cells. Nat. Mater..

[721] Dandena B. D., Wu L.-T., Yeh T.-I., Chang C.-Y., Jiang J.-C., Tsai D.-S., Ihrig M., Serbessa G. G., Yang S.-C., Lakshmanan K. (2025). Intrinsically-ductile
argyrodite solid electrolytes to improve the performance of all-solid-state
lithium batteries. Chem. Eng. J..

[722] Kitaba L. T., Nikodimos Y., Merso S. K., Taklu B. W., Serbessa G. G., Dilebo W. B., Yeh T.-I., Iskandar J. A., Valencia F., Chang C.-Y. (2025). Overcoming Chemo-Mechanical
Instability at Silicon-Solid Electrolyte Interfaces in Solid-State
Batteries. ACS Appl. Mater. Interfaces.

[723] Sun F., Dong K., Osenberg M., Hilger A., Risse S., Lu Y., Kamm P. H., Klaus M., Markötter H., García-Moreno F. (2018). Visualizing the morphological and compositional
evolution of the interface of InLi-anode| thio-LISION electrolyte
in an all-solid-state Li-S cell by in operando synchrotron X-ray tomography
and energy dispersive diffraction. J. Mater.
Chem. A.

[724] Gunnarsdóttir A. B., Amanchukwu C. V., Menkin S., Grey C. P. (2020). Noninvasive in situ
NMR study of
“dead lithium” formation and lithium corrosion in full-cell
lithium metal batteries. J. Am. Chem. Soc..

[725] Slade, P. G. Electrical contacts: principles and applications; CRC Press, 2017.

[726] Vetter K.
J., Barnatt S. (1968). Electrochemical
kinetics: theoretical
aspects. J. Electrochem. Soc..

[727] Kim S., Jung C., Kim H., Thomas-Alyea K. E., Yoon G., Kim B., Badding M. E., Song Z., Chang J., Kim J. (2020). The role
of interlayer
chemistry in Li-metal growth through a garnet-type solid electrolyte. Adv. Energy Mater..

[728] Krauskopf T., Richter F. H., Zeier W. G., Janek J. r. (2020). Physicochemical
concepts of the lithium metal anode in solid-state batteries. Chem. Rev..

[729] Liu X., Garcia-Mendez R., Lupini A. R., Cheng Y., Hood Z. D., Han F., Sharafi A., Idrobo J. C., Dudney N. J., Wang C. (2021). Local electronic structure
variation resulting in Li ‘filament’formation within
solid electrolytes. Nat. Mater..

[730] Han F., Westover A. S., Yue J., Fan X., Wang F., Chi M., Leonard D. N., Dudney N. J., Wang H., Wang C. (2019). High electronic
conductivity as the origin of lithium dendrite formation within solid
electrolytes. Nat. Energy.

[731] Bezabh H. K., Abrha L. H., Chiu S.-F., Nikodimos Y., Hagos T. M., Tsai M.-C., Su W.-N., Hwang B. J. (2025). Enhancing
ionic conductivity and air stability of Li7La3Zr2O12 garnet-based
electrolyte through dual-dopant strategy. J.
Alloys Compd..

[732] Yang C., Zhang L., Liu B., Xu S., Hamann T., McOwen D., Dai J., Luo W., Gong Y., Wachsman E. D. (2018). Continuous plating/stripping
behavior of solid-state lithium metal anode in a 3D ion-conductive
framework. Proc. Natl. Acad. Sci. U. S. A..

[733] Xu R., Liu F., Ye Y., Chen H., Yang R. R., Ma Y., Huang W., Wan J., Cui Y. (2021). A morphologically stable
Li/electrolyte interface for all-solid-state batteries enabled by
3D-micropatterned garnet. Adv. Mater..

[734] Huang Y., Zhang Y., Wu R., Shao B., Deng R., Das R., Han F. (2024). Hydride-Based
Interlayer
for Solid-State Anode-Free Battery. ACS Energy
Lett..

[735] Serbessa G. G., Taklu B. W., Nikodimos Y., Temesgen N. T., Muche Z. B., Merso S. K., Yeh T.-I., Liu Y.-J., Liao W.-S., Wang C.-H. (2024). Boosting
the Interfacial Stability of the Li6PS5Cl Electrolyte with a Li Anode
via In Situ Formation of a LiF-Rich SEI Layer and a Ductile Sulfide
Composite Solid Electrolyte. ACS Appl. Mater.
Interfaces.

[736] Dandena B. D., Su W. N., Tsai D. S., Nikodimos Y., Taklu B. W., Bezabh H. K., Desta G. B., Yang S. C., Lakshmanan K., Sheu H. S. (2025). Li-Sb Alloy Formation
Strategy to Improve Interfacial Stability of All-Solid-State Lithium
Batteries. Small Methods.

[737] Serbessa G. G., Nikodimos Y., Taklu B. W., Merso S. K., Muche Z. B., Dandena B. D., Vallal S. A., Yeh T.-I., Valencia F., Hung Y.-F. (2025). Stabilizing the interface
between Li6PS5Cl argyrodite sulfide solid electrolyte and Li via in
situ formed LiF-Li3 Bi lithiophobic-lithiophilic bifunctional layer. Energy Storage Mater..

[738] Lee J. H., Oh S.-H., Yim H., Lee H.-J., Kwon E., Yu S., Kim J. S., Song J., Koo J., Cho J. (2024). Interfacial stabilization strategy via In-doped
Ag metal coating enables a high cycle life of anode-free solid-state
Li batteries. Energy Storage Mater..

[739] Jiang S.-K., Yang S.-C., Huang W.-H., Sung H.-Y., Lin R.-Y., Li J.-N., Tsai B.-Y., Agnihotri T., Nikodimos Y., Wang C.-H. (2023). Enhancing
the interfacial
stability between argyrodite sulfide-based solid electrolytes and
lithium electrodes through CO 2 adsorption. J. Mater. Chem. A.

[740] Wen J., Wang T., Wang C., Dai Y., Song Z., Liu X., Yu Q., Zheng X., Ma J., Luo W. (2024). A Tailored Interface Design for Anode-Free
Solid-State Batteries. Adv. Mater..

[741] Kim K. H., Lee M.-J., Ryu M., Liu T.-K., Lee J. H., Jung C., Kim J.-S., Park J. H. (2024). Near-strain-free
anode architecture enabled by interfacial diffusion creep for initial-anode-free
quasi-solid-state batteries. Nat. commun..

[742] Nikodimos Y., Ihrig M., Taklu B. W., Su W.-N., Hwang B. J. (2023). Solvent-Free Fabrication of Freestanding
Inorganic
Solid Electrolyte Membranes: Challenges, Progress, and Perspectives. Energy Storage Mater..

[743] Oh J., Choi S. H., Kim J. Y., Lee J., Lee T., Lee N., Lee T., Sohn Y., Chung W. J., Bae K. Y. (2023). Anode-less
all-solid-state batteries operating at room temperature
and low pressure. Adv. Energy Mater..

[744] Dandena B. D., Tsai D.-S., Wu S.-H., Su W.-N., Hwang B. J. (2025). Review on interface issues of Li-argyrodite-based
solid-state
Li-metal batteries. EES Batteries.

[745] Huang W.-Z., Liu Z.-Y., Xu P., Kong W.-J., Huang X.-Y., Shi P., Wu P., Zhao C.-Z., Yuan H., Huang J.-Q. (2023). High-areal-capacity
anode-free all-solid-state lithium batteries enabled by interconnected
carbon-reinforced ionic-electronic composites. J. Mater. Chem. A.

[746] Wang Y., Liu Y., Nguyen M., Cho J., Katyal N., Vishnugopi B. S., Hao H., Fang R., Wu N., Liu P. (2023). Stable Anode-Free All-Solid-State Lithium Battery
Through Tuned Metal Wetting on the Copper Current Collector. Adv. Mater..

[747] Ding Y., Yi N., Zhang F., Li H., Zhan T., Guo Z., Zhu J., Chen J. (2025). Recent advances
in 3D Cu current collectors for Li metal anodes. Mater. Sci. Eng., B.

[748] Tian W., Zhang H., Duan X., Sun H., Shao G., Wang S. (2020). Porous carbons: structure-oriented
design and versatile applications. Adv. Funct.
Mater..

[749] Farmahini A. H., Krishnamurthy S., Friedrich D., Brandani S., Sarkisov L. (2021). Performance-based
screening of porous
materials for carbon capture. Chem. Rev..

[750] Kong C., Wang F., Liu Y., Liu Z., Liu J., Feng K., Pei Y., Wu Y., Wang G. (2024). Constructing
three-dimensional architectures to design advanced copper-based current
collector materials for alkali metal batteries: From nanoscale to
microscale. Molecules.

[751] Liu X., Wu R., Hu X., Ganose A. M., Luo J., Pinnock I., Naresh N., Zhu Y., Fan Y., Wang T. (2025). 3D Porous Zinc Scaffold
Anodes for Enhanced Stability
and Performance in Zinc-Ion Energy Storage Systems. ACS Nano.

[752] Shi S., Zhou D., Jiang Y., Cheng F., Sun J., Guo Q., Luo Y., Chen Y., Liu W. (2024). Lightweight Zn-philic
3D-Cu scaffold for customizable zinc ion batteries. Adv. Funct. Mater..

[753] Wang Y., Raj V., Naik K. G., Vishnugopi B. S., Cho J., Nguyen M., Recker E. A., Su Y., Celio H., Dolocan A. (2025). Control of Two Solid Electrolyte Interphases at the
Negative Electrode of an Anode-Free All Solid-State Battery based
on Argyrodite Electrolyte. Adv. Mater..

[754] Bazri B., Huang J.-Y., Liu C.-E., Liao S.-C., Wei D.-H., Hu S.-F., Liu R.-S. (2025). Spatially
Controlled
Lithium Utilization in Anode-Free All-Solid-State Batteries via Diffusive
Interface. Nano Energy.

[755] Huang Y., Zhang Y., Wu R., Shao B., Deng R., Das R., Han F. (2024). Hydride-Based Interlayer
for Solid-State Anode-Free Battery. ACS Energy
Lett..

[756] Choi S. H., Baek C. H., Oh J., Lee G.-J., Kim M., Lee H., Yoo D.-J., Jung Y. S., Kim K., Yu J.-S. (2025). Silver exsolution from Li-argyrodite electrolytes for
initially anode-free all-solid-state batteries. Nat. Commun..

[757] Seo D.-B., Kim D., Kim M.-R., Kwon J., Kook H. J., Kang S., Yim S., Lee S. S., Shin D. O., An K.-S. (2025). Tailoring
Artificial
Solid Electrolyte Interphase via MoS2 Sacrificial Thin Film for Li-Free
All-Solid-State Batteries. Nano-Micro Lett..

[758] Ko D. Y., Kim H. J., Park H., Kim E., Kim H. (2025). Correlation between the Microstructure of the Carbon
Protective Layer
and Cycle Performance of Anode-Free All-Solid-State Lithium-Ion Batteries. ACS Nano.

[759] Kim J., Lee S., Kim J., Park J., Lee H., Kwon J., Sun S., Choi J., Paik U., Song T. (2024). Regulating Li electrodeposition by constructing Cu-Sn nanotube thin
layer for reliable and robust anode-free all-solid-state batteries. Carbon Energy.

[760] Lee D., Kim J., Sun S., Kim J., Paik U., Song T. (2023). Sacrificial
cathode additives for enhanced cycle performance for
liquid and all-solid-state anode-free lithium secondary batteries. J. Alloys Compd..

[761] Kang J., Kim J., Kim R., Lim Y. J., Lee J.-W. (2025). Mechanistic insight
into calendar aging of anode-less
all-solid-state batteries. Energy Storage Mater..

[762] Metzler M., Doerrer C., Sun Y., Matthews G., Liotti E., Grant P. S. (2025). Effect of Silver
Particle Distribution
in a Carbon Nanocomposite Interlayer on Lithium Plating in Anode-Free
All-Solid-State Batteries. ACS Appl. Mater.
Interfaces.

[763] Deysher G., Oh J. A. S., Chen Y.-T., Sayahpour B., Ham S.-Y., Cheng D., Ridley P., Cronk A., Lin S. W.-H., Qian K. (2024). Design principles for
enabling an anode-free sodium all-solid-state battery. Nat. Energy.

[764] Feng W., Li C., Wang P., Hu J., Yu X., Zhang Y., Zhu X., Zhang D., Xia Y., Zhao Y. (2025). Highly stable anode-free solid-state sodium batteries
enabled by
electrostatic shielding and nitrogenated interphase. Chem. Eng. J..

[765] Sun C., Li Y., Sun Z., Yuan X., Jin H., Zhao Y. (2024). Ferroelectric
interface for efficient sodium metal cycling in anode-free
solid-state batteries. Mater. Today.

[1000] Shitaw K. N., Nikodimos Y., Hagos T. M., Balajig
Tamilarasan E., Taklu B. W., Pan S.-Y., Chen Y.-N., Yang S.-C., Muche Z. B., Su H. H. (2026). Failure
mechanisms and scalability of anode-free lithium-metal and lithium–sulfur
batteries. Nat. Rev. Clean Technol..

